# Homogeneous cobalt-catalyzed reductive amination for synthesis of functionalized primary amines

**DOI:** 10.1038/s41467-019-13351-7

**Published:** 2019-11-29

**Authors:** Kathiravan Murugesan, Zhihong Wei, Vishwas G. Chandrashekhar, Helfried Neumann, Anke Spannenberg, Haijun Jiao, Matthias Beller, Rajenahally V. Jagadeesh

**Affiliations:** Leibniz-Institut für Katalyse e.V. an der Universität Rostock, Albert-Einstein Str. 29a, Rostock, D-18059 Germany

**Keywords:** Homogeneous catalysis, Synthetic chemistry methodology

## Abstract

The development of earth abundant 3d metal-based catalysts continues to be an important goal of chemical research. In particular, the design of base metal complexes for reductive amination to produce primary amines remains as challenging. Here, we report the combination of cobalt and linear-triphos (bis(2-diphenylphosphinoethyl)phenylphosphine) as the molecularly-defined non-noble metal catalyst for the synthesis of linear and branched benzylic, heterocyclic and aliphatic primary amines from carbonyl compounds, gaseous ammonia and hydrogen in good to excellent yields. Noteworthy, this cobalt catalyst exhibits high selectivity and as a result the -NH_2_ moiety is introduced in functionalized and structurally diverse molecules. An inner-sphere mechanism on the basis of the mono-cationic [triphos-CoH]^+^ complex as active catalyst is proposed and supported with density functional theory computation on the doublet state potential free energy surface and H_2_ metathesis is found as the rate-determining step.

## Abstract (web summary)

Despite their higher abundance, 3d metal-based catalysts are less investigated than their precious metal counterparts. Here, the authors report a cobalt-triphos complex as molecularly-defined non-noble metal catalyst for the reductive amination of carbonyl compounds with gaseous ammonia and hydrogen.

## Introduction

Catalysis constitutes an indispensable tool for controlling all kinds of chemical transformations^1–11^. Although catalysts are routinely employed in industrial production of fine and bulk chemicals as well as for exhaust gas decomposition in environmental technologies and enzymatic processes, there is a constant need for better and improved catalytic systems^1–11^. With respect to organometallic catalysts, their activity and selectivity are controlled to a large extent by the nature of the metal and the adjacent ligands^1,2,8–11^. In this regard, precious metals were generally believed to be crucial components^12–23^. Indeed, organometallic complexes based on palladium (coupling reactions)^12–14^, ruthenium (metathesis)^15,16^, rhodium (hydrogenations and hydroformylations)^17–19^, platinum (hydrosilylations)^20,21^, and iridium (hydrogenations)^22,23^ have revolutionized organic synthesis. Key for their success is the use of a broad variety of complexes based on certain privileged ligands^24,25^. However, the limited availability (10^−7^–10^−6^% proportion of weight in the Earth’s crust) and consequently the higher price as well as the toxicity of some derivatives of these metals^26,27^ have spurred interest towards the development of alternative earth abundant metal catalysts. Hence in recent years, 3d-metal complexes have been successfully developed for a variety of reactions including hydrogenation of carboxylic acids, esters, ketones, nitriles and olefins^28–37^. Nevertheless, more challenging reactions such as reductive amination with ammonia and hydrogen to access primary amines were scarcely explored^38–44^. In general, applying molecularly defined catalysts this transformation suffers from low selectivity to the desired product due to side reactions such as over-alkylation or reduction to the corresponding alcohols^38–44^. In addition, catalyst deactivation by ammonia constitutes another problem^45^. Thus, to the best of our knowledge no defined homogeneous catalysts based on available 3d-metals are known for this transformation and only a few Rh-, Ir-, and Ru-complexes have been reported to catalyze amination of carbonyl compounds with ammonia and molecular hydrogen^38–44^. Thus, in the past reductive aminations are mainly relied on heterogeneous catalysts of precious metals^38,39,46–50^ or Raney nickel^38,39,50,51^. However, the latter material is limited in its application due to selectivity, stability and handling problems. Notably in 2017, we reported specific supported cobalt nanoparticles derived from metal organic frameworks, which proved to be general reductive amination catalysts^52^. In addition, very recently, Kempe^53^ and our group^54^ disclosed nickel materials. Despite these notable advancements, the development of related homogeneous non-noble metal catalysts remains interesting because of the inherent advantage regarding activity—in principle, all the individual metal centers can be active here. Furthermore, compared to homogeneous catalysts the upscaling of advanced heterogeneous materials possesses additional challenges.

Here, we report that the specific combination of cobalt and bis(diphenylphosphinoethyl)phenylphosphine (so-called linear triphos) allows the reductive amination of broad variety of aldehydes and ketones with ammonia in presence of molecular hydrogen, and this enables the synthesis of a series of functionalized and structurally diverse linear and branched benzylic, heterocyclic and aliphatic amines. The resulting primary amines serve as key precursors and central intermediates for the production of advanced chemicals, pharmaceuticals, agrochemicals, biomolecules, and materials^55–57^. Our present work is complementary to the known syntheses of primary benzylic and aliphatic amines by direct catalytic amination of alcohols^58–61^ and hydroamination using ammonia^45,62–64^.

## Results

### Catalyst and reaction design

We started our investigations to identify potentially active non-noble metal complexes based on iron, manganese and cobalt for the reaction of 4-methylbenzaldehyde with ammonia and molecular hydrogen. In general, the presence of strongly coordinating anions (e.g., halides) is inferior for hydrogen catalysis. Hence, Fe(BF_4_)_2_•6H_2_O and Co(BF_4_)_2_•6H_2_O were employed as metal salts. In case of manganese, the corresponding tetrafluoroborate salt is not commercially available, therefore the inexpensive manganese(II) acetate was used. To avoid the formation of well-known Werner-type ammonia complexes, selected privileged phosphine ligands (**L1-L8**) with strong coordination to the metal center were  chosen (Table 1). Testing the in situ generated respective Fe-phosphorus complexes, all ligands produced inactive catalysts except for **L7**. This Fe-**L7** system showed some activity and gave the secondary imine as the main product. However, no 4-methylbenzylamine was observed. In case of manganese, none of the ligands led to an active catalyst. Conversely, Co-complexes based on linear- and tripodal-triphos ligands (Co-**L7** and Co-**L8**) produced the desired primary amine in 96 and 93% yield, respectively. Next applying Co-**L7**, important reaction parameters such as catalyst concentration, temperature and pressure of hydrogen were tested (Supplementary Table 1, entries 2–5). Optimal results for the synthesis of 4-methylbenzylamine were achieved with a combination of 3 mol% of Co(BF_4_)_2_•6H_2_O and 4 mol% of **L7** at 40 bar of hydrogen, 5 bar of ammonia and 100 °C. Variation of different solvents revealed the importance of alcohols, with 2,2,2-trifluoroethanol (TFE) as the best one (Supplementary Table 2). Notably, this 3d-metal catalyst system allows performing reductive aminations with ammonia under milder reaction conditions compared to all previously reported precious metal complexes^40–44^.

**Table 1:**
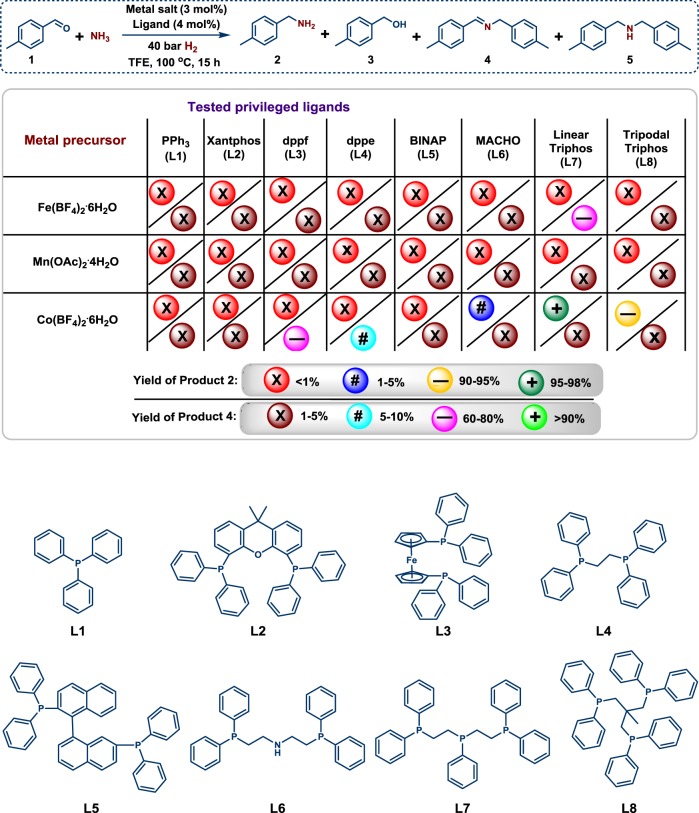
Reductive amination of 4-methylbenzaldehyde with in situ generated complexes.

Reaction conditions: ^a^0.5 mmol 4-methylbenzaldehyde, 3 mol% metal salt, 4 mol% ligand, 5 bar NH_3_, 40 bar H_2_, 2 mL TFE, 100 °C, 15 h, GC yields using n-hexadecane as standard.

To know the structure of the in situ-formed cobalt-triphos complex (C-**L7**) and to understand its mode of reactivity, the molecularly defined Co-**L7** complex **A** was prepared (Methods). Surprisingly, the single crystal analysis of complex **A** revealed the coordination of two phosphine ligands (1:2 ratio of Co:**L7**) to the Co center, in which one ligand coordinates via three phosphorus atoms and the second ligand via two phosphorus atoms (Supplementary methods, complex **A**). Performing the crystallization attempts without vigorous exclusion of air gave the partially oxidized phosphine ligand, which forms cobalt complex **B**. Both complexes **A** and **B** were observed as cobalt (II) species consisting of a complex dication and two tetrafluoroborates. Comparing these two defined complexes in the benchmark reaction, complex **A** exhibited similar activity and selectivity to that of the in situ generated Co-triphos system, while **B** is much less active and produced only 70% of the corresponding secondary imine **4** as the sole product (Supplementary Table 1, entries 8–9). This indicates a major deactivation of the catalyst by oxidation of phosphine to phosphine oxide. poisoning experiments were performed. As expected, adding Hg or 50 mol%PPh_3_ to the reaction under standard conditions did not affect the activity or selectivity of complex **A** (Supplementary Table 4).

### Synthesis of linear primary amines from aldehydes

After having identified the active homogeneous cobalt catalyst system [L7CoH]^+^, we explored its general applicability for the preparation of primary amines.

**Fig. 1:**
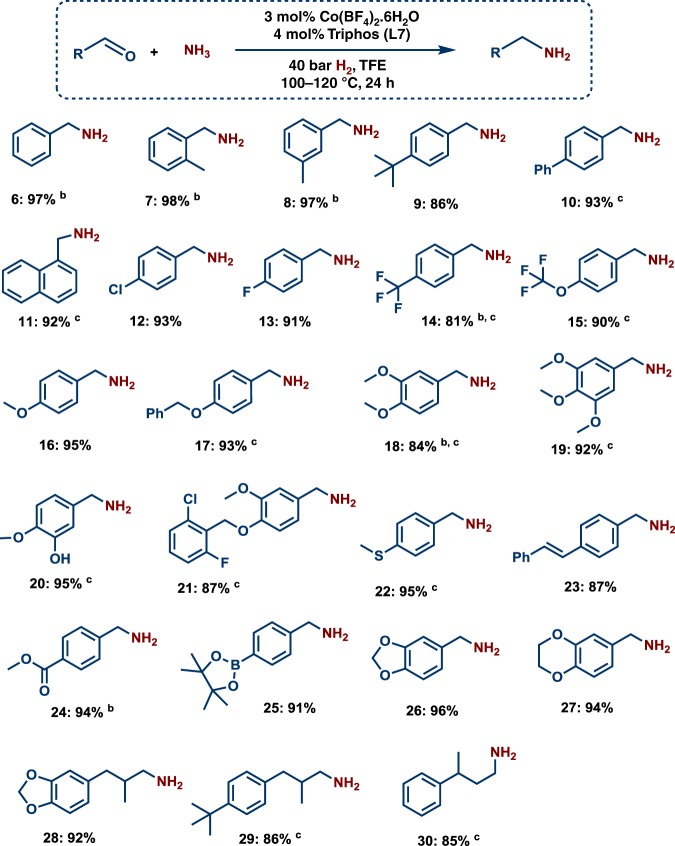
Cobalt-triphos catalyzed synthesis of primary amines from aldehydes. Reaction conditions: ^a^0.5 mmol aldehyde, 3 mol% Co(BF_4_)_2_.6H_2_O, 4 mol% triphos (**L7**), 5–7 bar NH_3_, 40 bar H_2_, 2 mL degassed TFE, 100 °C, 24 h. ^b^GC yields using n-hexadecane as standard. ^c^Same as ‘a’ at 120 °C. Isolated as free amines and converted to hydrochloride salts. Corresponding hydrochloride salts were subjected to NMR analysis.

As shown in Fig. 1, substituted, functionalized and structurally diverse aldehydes underwent amination to produce linear primary amines in good to excellent yields at 100–120 °C. Substrates containing either electron-donating or electron-withdrawing groups were successfully reacted and gave the desired products. The tested halogenated aldehydes were well tolerated and produced corresponding amines without significant dehalogenations (<5%) (Fig. 1, products **12**–**15** and **21**). For any catalyst applicable in organic synthesis as well as drug discovery, achieving a high degree of chemoselectivity is important, yet challenging. To showcase this aspect, reductive aminations of various functionalized aldehyde were performed. Reducible groups such as C–C double bonds and esters remained untouched. In addition, thioethers and boronic ester groups are well tolerated (Fig. 1; products **22**–**25**). Primary amines of 3,4-methylenedioxy and benzo-1,4-dioxane, which represent versatile motifs in many drugs and natural products, were prepared in up to 96 % yield (products **26**–**28**). Finally, also aliphatic aldehydes produced the corresponding amines (Fig. 1, products **28**–**30**).

### Synthesis of branched primary amines from ketones

**Fig. 2:**
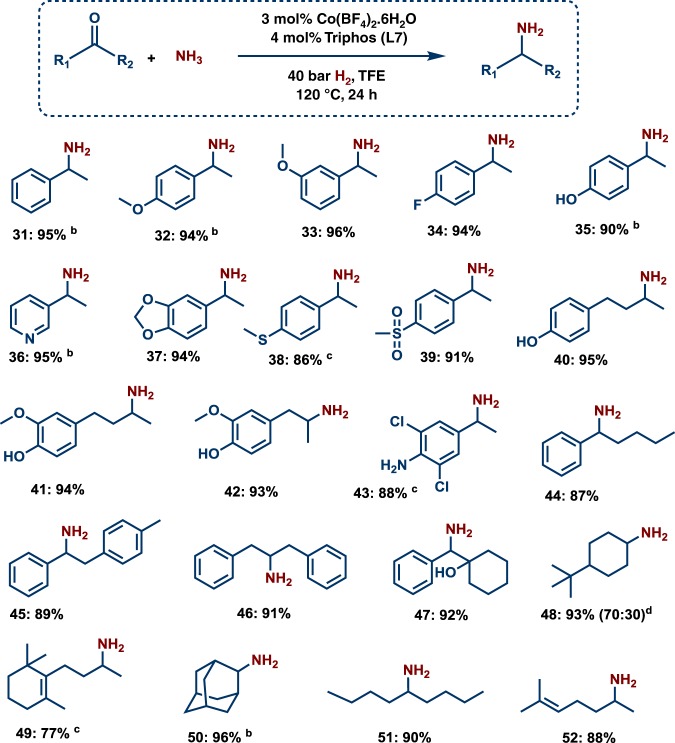
Synthesis of primary amines from ketones using cobalt-triphos catalyst. Reaction conditions: ^a^0.5 mmol ketone, 3 mol% Co(BF_4_)_2_⋅6H_2_O, 4 mol% triphos (**L7**), 5–7 bar NH_3_, 40 bar H_2_, 2 mL degassed TFE, 120 °C, 24 h. ^b^GC yields using n-hexadecane as standard. ^c^Same as ‘**a**’ with 50 bar H_2_. ^d^Diastereomeric ratio. Isolated as free amines and converted to hydrochloride salts. Corresponding hydrochloride salts were subjected to NMR analysis.

**Fig. 3:**
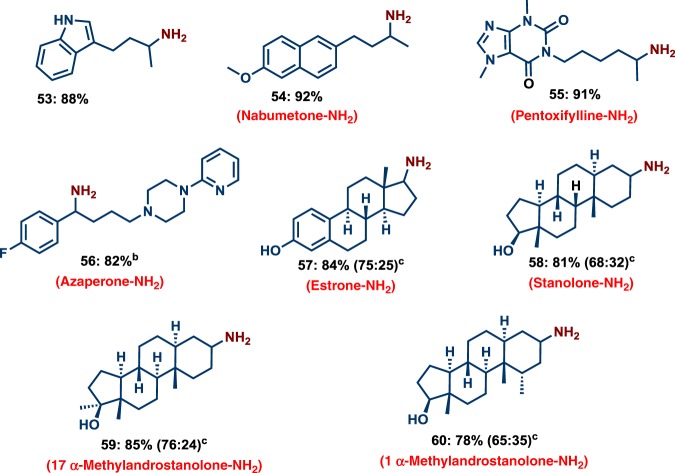
Co-catalyzed amination of bioactive compounds. Reaction conditions: ^a^0.5 mmol substrate, 3 mol% Co(BF_4_)_2_⋅6H_2_O, 4 mol% triphos (**L7**), 5–7 bar NH_3_, 40 bar H_2_, 2 mL degassed TFE, 120 °C, 24 h. ^b^Same as ‘a’ with 50 bar H_2_. ^c^Diastereomeric ratio. Isolated as free amines and converted to hydrochloride salts. Corresponding hydrochloride salts were subjected to NMR analysis.

Compared to aldehydes, amination of ketones is more challenging, because the hydrogenation of the sterically hindered imine is more difficult. Nevertheless, this Co-triphos catalyst system is active and selective for the reductive amination of ketones, too (Figs. 2, 3). As a result, 30 ketones were efficiently aminated to produce branched primary amines in high yields. Substrates bearing easily coordinating groups to the metal such as –NH_2_, –OH and phenolic groups as well as pyridines gave 90–95% yield of corresponding amines (Fig. 2; products **35**–**36** and **40**–**43**). Further aliphatic ketones smoothly gave the branched amines in up to 93% yield (Fig. 2; products **48**–**52**).

### Amination of life science molecules and steroid-derivatives

For any potential catalyst, its utility for the refinement of complex molecules is of central importance. In order to prove the general applicability of our catalytic system, we performed the amination of structurally complex ketones, including existing drug and steroid-based molecules (Fig. 3). Gratifyingly, cobalt-triphos is highly efficient and selective for the amination of drugs such as Nabumetone, Pentoxifylline, and Azaperone as well as Estrone and Stanolone-based steroid derivatives (Fig. 3). This methodology offers many opportunities for late stage functionalization of life science and bioactive molecules.

## Discussion

**Fig. 4:**
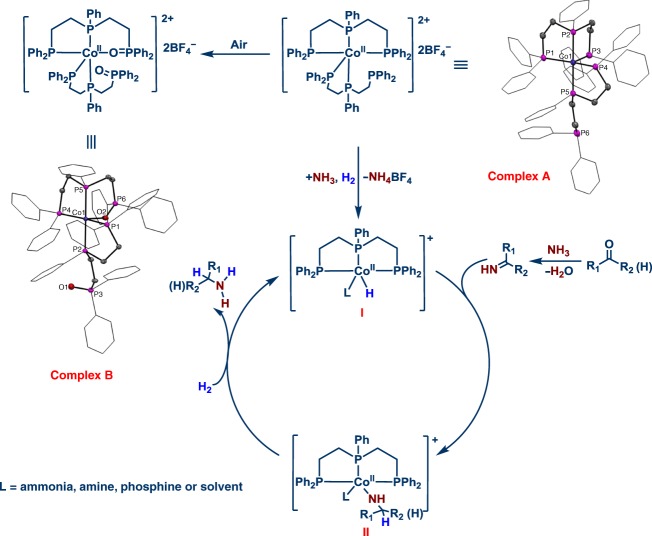
Proposed reaction mechanism for the Co-triphos catalysed reductive amination. Molecular structure of the cation of complexes A and B. Displacement ellipsoids are drawn at the 30% probability level; phenyl rings are shown as wireframe; hydrogen atoms are omitted for clarity.

Since Co^II^(BF_4_)_2_•6H_2_O is the most active cobalt salt for the reductive amination of 4-methylbenzaldehyde (Supplementary Table 3), the mono-cationic hydride [L7CoH]^+^ (**I**) complex is proposed as the active catalyst along the catalytic cycle (Fig. 4) on the basis of our results as well as those of the previously reported cobalt/phosphine-catalyzed hydrogenation reactions^31^^,^^65–68^, Starting from the cationic complex **A** [(L7)_2_Co^II^]^2+^, the first step is the dissociation of one L7 ligand and the formation of the active cobalt hydride catalyst [L7CoH]^+^ (**I**) in the presence of ammonia and H_2_. Without ammonia present, no hydrogenation of 4-methylbenzaldehyde to the corresponding alcohol occurred (Supplementary Table 1, entries 6–7).

Next, the primary imine formed from **1** and ammonia generates complex **II**. In agreement with previous work using ruthenium complexes and based on our DFT calculations, we propose first substrate coordination and then beta hydride addition. Finally, coordination of H_2_ followed by hydrogenolysis releases the primary amine and regenerates catalytically active species **I**.

**Fig. 5:**
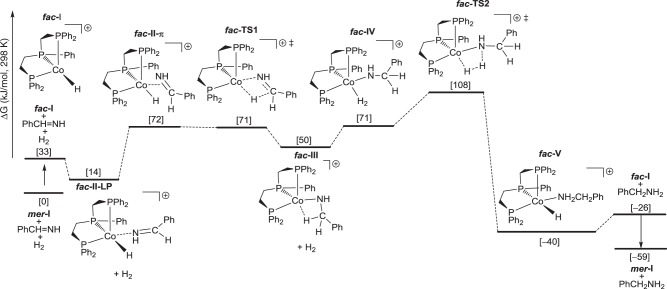
Gibbs free energy surface for Ph−CH=NH hydrogenation in TFE solvation. Ph−CH=NH hydrogenation by using mono-cationic [triphos-CoH]^+^ as active catalyst.

To understand the detailed mechanism, we carried out B3PW91 density functional theory computations for the hydrogenation of phenylmethanimine (Ph-CH = NH) generated from benzaldehyde and NH_3_. In our calculations we used the real-size complexes and substrate and calculated the catalytic cycle in the gas phase as well as in a solution of 2,2,2-trifluoroethanol (dielectric constant = 26.69) without and with van de Waals dispersion correction. All these data are listed in Supplementary Information. The potential energy surfaces show the same trend and shape but differ quantitatively. In the gas phase and in a solution, the apparent barriers are close (96 vs. 108 kJ/mol), while that in a solution with dispersion correction is highly underestimated (29 kJ/mol). In addition to the mono-cationic catalyst [L7CoH]^+^, we included the di-cationic catalyst [L7Co]^2+^, and the apparent barriers are much too high (128 kJ/mol in the gas phase and 212 kJ/mol in a solution, 122 kJ/mol in a solution with dispersion correction, Supplementary Figs. 8–10) and this catalytic cycle can be discarded. We therefore discussed our results of the mono-cationic complex [L7CoH]^+^ (**I**) in solvation. Since complex **I** has *fac* and *mer* conformations under equilibrium, we computed both catalytic cycles and found that ***mer*-I** is more stable than ***fac*-I** by 33 kJ/mol, while ***fac*-I** based catalytic cycle has lower apparent barrier than that of ***mer*-I** (108 vs. 140 kJ/mol, Supplementary Fig. 6). On the basis of Curtin–Hammett principle, the ***fac*-I** based catalytic cycle is more preferred kinetically (Fig. 5). Starting from the ***mer*-I**, the coordination of Ph−CH = NH to form ***fac*-II-LP** is endergonic by 14 kJ/mol, and the formation of π-coordinated ***fac*-II-**π is endergonic by 72 kJ/mol. The Gibbs free energy barrier of Ph−CH = NH insertion into ***fac*-I** is 71 kJ/mol. The formation of intermediate ***fac*-III** with agostic interaction is endergonic by 50 kJ/mol. In the second step, H_2_ coordination to form ***fac*-IV** is endergonic by 71 kJ/mol. The metathesis step has Gibbs free energy barrier of 108 kJ/mol for ***fac*-IV**. The formation of ***fac*-V** is exergonic by 40 kJ/mol. The release of amine from complex ***fac*-V** with the regeneration of ***mer*-I** is exergonic 59 kJ/mol. The transition state of H_2_ metathesis represents the highest point on the Gibbs free energy surface and is the rate-determining transition state; and the apparent Gibbs free energy barrier is 108 kJ/mol from ***mer*-I**. On the basis of our reaction conditions (100–120 °C, 40 bar H_2_ and 15–24 h reaction time), the barrier is reasonable. This catalytic cycle is similar with that proposed by Hanson and Jones. It is noted that the result with GD3BJ correction (Supplementary Figs. 9 and 12) has extremely low apparent barrier (29 kJ/mol).

In conclusion, we demonstrate that reductive aminations for the preparation of primary amines can be easily performed using non-noble metal-based homogeneous catalysts. Key to success for this achievement is the use of a specific metal ligand system (cobalt-triphos), which enables the synthesis of a broad variety of linear and branched benzylic, heterocyclic, and aliphatic amines starting from inexpensive and easily accessible carbonyl compounds, gaseous ammonia and hydrogen. Remarkably, this cobalt-triphos system works under milder reaction conditions compared to the previously reported precious homogeneous catalysts for reductive amination with ammonia. Isolation of an active pre-catalyst revealed the fast oxidation of phosphine moiety as a potential deactivation pathway of the catalyst. Density functional theory computations verified the proposed inner-sphere mechanism with the H_2_ metathesis step as the rate-determining step.

## Methods

### General considerations

Unless specified, all substrates were obtained commercially from various chemical companies and their purity has been checked before use. Unless otherwise stated, all commercial reagents were used as received without purification. All catalytic reactions were carried out in 300 mL and 100 mL autoclaves (PARR Instrument Company). In order to avoid unspecific reactions, catalytic reactions were carried out either in glass vials, which were placed inside the autoclave, or glass/Teflon vessel fitted autoclaves. GC conversion and yields were determined by GC-FID, HP6890 with FID detector, column HP530 m × 250 mm × 0.25 μm. ^1^H, ^13^C, ^19^F NMR data were recorded on a Bruker ARX 300 and Bruker ARX 400 spectrometers using DMSO-d_6_, CD_3_OD and CDCl_3_ solvents. HRMS data were recorded on (1) ESI-HRMS: HPLC System 1200 /ESI-TOF-MS 6210 (Agilent).

X-ray crystal structure analysis of Complex **A** and Complex **B**: Data were collected on a Bruker Kappa APEX II Duo diffractometer. The structures were solved by direct methods (SHELXS-97: Sheldrick, G. M. *Acta Cryst*. **2008**, *A*64, 112.) and refined by full-matrix least-squares procedures on *F*^2^ (SHELXL-2014: Sheldrick, G. M. *Acta Cryst*. **2015**, *C*71, 3.). XP (Bruker AXS) and Mercury (Macrae, C. F., Edgington, P. R., McCabe, P., Pidcock, E., Shields, G. P., Taylor, R., Towler, M., van de Streek, J. *J. Appl. Cryst*. **2006**, *39*, 453.) were used for graphical representations.

### Synthesis of cobalt (II) complexes A and B

In 100 mL dried schlenk tube, 340.63 mg of Co(BF_4_)_2_.6H_2_O (1.0 mmol) was stirred in 40 mL of THF (dry and degassed) for 5 min under argon to dissolve metal salt completely to give pink colored solution. Then, 535.55 mg of triphos ((phenylphosphanediyl) bis(ethane-2,1-diyl)) bis (diphenylphosphane) (1.0 mmol) was added to the solution of metal precursor. Upon adding the ligand, the pink colored catalyst precursor solution was turned to brown colored solution. To this, another 20 mL of THF (dry and degassed) was added and stirring was continued for 2 h at RT. In 10 min of stirring time, the ligand was completely dissolved. After 1 h of stirring, the brown color solid was started to form along with some brown color crystal type material. After the completion of the reaction, the reaction mixture was stored at −30 °C for overnight. The THF solution was removed by using syringe and the complex formed was washed with 10 mL of THF (dry and degassed). Further, it was washed with dry and degassed hexane (2 × 10 mL) and then dried under high vacuum for 6 h, to get a brown color solid in 48–50% yield. The dark brown crystals were observed along with brown solid. These crystals were separated carefully and recrystallized with dry and degassed ethanol under argon. The crystals obtained were suitable for X-ray analysis. The oxide species of complex **B (**yellow crystals) were formed during the crystallization, when we carried out in normal solvent without dry and degas.

### General procedure for synthesis of primary amines

The magnetic stirring bar and Co(BF_4_)_2_.6H_2_O (3 mol%) and linear triphos ((phenylphosphanediyl)bis(ethane-2,1-diyl))bis(diphenylphosphane) (4 mol%) were transferred to 8 mL glass vial and then 2 mL degassed (degassed under argon for 15 minutes before adding) trifluoroethanol (TFE) solvent was added. The colorless solution turned in to pale yellow first and then finally to brown color by stirring under argon for 15 min. Then, 0.5 mmol corresponding carbonyl compound was added to the reaction vial. The vial was fitted with septum, cap, and needle. The reaction vials (8 vials with different substrates at a time) were placed into a 300 mL autoclave. The autoclave was flushed with hydrogen twice at 30 bar pressure and then it was pressurized with 5–7 bar ammonia gas and 40 bar hydrogen. The autoclave was placed into an aluminum block preheated at 130 °C (placed 30 minutes before counting the reaction time in ordered to attain reaction temperature) and the reactions were stirred for a required time. During the reaction, the inside temperature of the autoclave was measured to be 120 °C and this temperature was used as the reaction temperature. After the completion of the reactions, the autoclave was cooled to room temperature. The remaining ammonia and hydrogen were discharged and the vials containing reaction products were removed from the autoclave. The reaction mixture was filtered off and washed thoroughly with ethyl acetate. The reaction products were analyzed by GC-MS. The crude product was purified by flash column chromatography. The corresponding primary amines were converted to their respective hydrochloride salt and characterized by NMR and GC-MS analysis. For converting into hydrochloride salt of amine, 1–2 mL methanolic HCl (0.5 M HCl in methanol) was added to the ether solution of respective amine and stirred at room temperature for 4–5 h. Then, the solvent was removed and the resulted hydrochloride salt of amine is dried under high vacuum. The yields were determined by GC for the selected amines: After completion of the reaction, n-hexadecane (100 µL) as standard was added to the reaction vials and the reaction products were diluted with ethyl acetate followed by filtration using a plug of silica and then analyzed by GC.

Note: Dry and degassed solvent should necessary for this transformation to achieve high yield and reactivity. Similarly, dry ligand and metal salts have been employed. And also well-defined complex-A is more active than in situ prepared system and Complex-A should be stored at −30 °C for maintaining its stability for a longer time.

### Computational methods and models

All calculations were carried out with Gaussian 09 program^69^. Geometry optimization was carried out in gas phase at the B3PW91^70^ level with the TZVP^71^ basis set. All optimized structures were further characterized either as energy minimums without imaginary frequencies or transition states with only one imaginary frequency by frequency calculations, which provided zero-point vibrational energies and thermodynamic corrections to enthalpy and Gibbs free energy at 298.15 K under 1 atmosphere. On the basis of B3PW91/TZVP geometries in gas phase, two types single-point energies were calculated, one including solvation effect of 2,2,2-trifluoroethanol (TFE) as solvent (dielectric constant ε = 26.69^72^) based on solute electron density (SMD) at the B3PW91/Def2-TZVP^73^ level (B3PW91-SMD) and one including solvation^73^ and van der Waals dispersion^74^ correction for the effect of phenyl substitution (B3PW91-SMD-D3). The Gibbs free energies were further corrected to standard state in solution with a standard concentration of 1 mol/L (*p* = 24.5 atm) from standard state in gas phase (*p* = 1 atm). Both mono-cationic hydride [L7CoH]^+^ (L7 = triphos) and di-cationic [L7Co(H)_2_]^2+^ complexes are proposed as potential active catalysts (Table 1).

## Supplementary information

Peer Review File

Supplementary Information

Description of Additional Supplementary Files

Supplementary Data 1

## Data Availability

The data that support the findings of this study are available from the corresponding authors (M.B. and R.V.J.) upon reasonable request. The X-ray crystallographic coordinates for structures reported in this study have been deposited at the Cambridge Crystallographic Data Centre (CCDC), under deposition numbers CCDC 1897492-1897493. These data can be obtained free of charge from The Cambridge Crystallographic Data Centre via www.ccdc.cam.ac.uk/data_request/cif.

## References

[1] Beller, M. & Bolm, C. *Transition Metals for Organic Synthesis* (Wiley-VCH, NewYork, 2008).

[2] Negishi E-i (2011). Magical power of transition metals: past, present, and future (Nobel lecture). Angew. Chem. Int. Ed..

[3] Smith, G. V., Notheisz, F. *Heterogeneous Catalysis in Organic Chemistry* (Academic Press, San Diego, 1999).

[4] Ertl, G., Knözinger, H., Weitkamp, J. *Environmental Catalysis* (WILEY‐VCH, 2008).

[5] Drauz, K., Gröger, H., May, O. *Enzyme Catalysis in Organic Synthesis* (Wiley‐VCH, 2012).

[6] Boersma AJ, Megens RP, Feringa BL, Roelfes G (2010). DNA-based asymmetric catalysis. Chem. Soc. Rev..

[7] James T, van Gemmeren M, List B (2015). Development and applications of disulfonimides in enantioselective organocatalysis. Chem. Rev..

[8] Leeuwen, P. W. N. M. V. *Homogeneous Catalysis: Understanding the Art* (Kluwer Academic Publishers, Boston, 2004).

[9] Cornils, B. & Herrmann, W. A. *Applied homogeneous Catalysis with Organometallic Compounds* (Wiley-VCH, Weinheim, 1996).

[10] Alig L, Fritz M, Schneider S (2019). First-row transition metal (de)hydrogenation catalysis based on functional pincer ligands. Chem. Rev..

[11] O’Reilly ME, Veige AS (2014). Trianionic pincer and pincer-type metal complexes and catalysts. Chem. Soc. Rev..

[12] de Meijere, A. & Diederich, F. *Metal-Catalyzed Cross-Coupling Reactions* (Wiley-VCH, ed. 2, Weinheim, 2004).

[13] Ruiz-Castillo P, Buchwald SL (2016). Applications of palladium-catalyzed C–N cross-coupling reactions. Chem. Rev..

[14] Hartwig JF (2008). Evolution of a fourth generation catalyst for the amination and thioetherification of aryl halides. Acc. Chem. Res..

[15] Alcaide B, Almendros P, Luna A (2009). Grubbs’ ruthenium-carbenes beyond the metathesis reaction: less conventional non-metathetic utility. Chem. Rev..

[16] Ogba OM, Warner NC, O’Leary DJ, Grubbs RH (2018). Recent advances in ruthenium-based olefin metathesis. Chem. Soc. Rev..

[17] Franke R, Selent D, Börner A (2012). Applied hydroformylation. Chem. Rev..

[18] Leeuwen, P. W. N. M. V. & Claver, C. *Rhodium Catalyzed Hydroformylation* (Springer Netherlands, 2002).

[19] Etayo P, Vidal-Ferran A (2013). Rhodium-catalysed asymmetric hydrogenation as a valuable synthetic tool for the preparation of chiral drugs. Chem. Soc. Rev..

[20] Troegel D, Stohrer J (2011). Recent advances and actual challenges in late transition metal catalyzed hydrosilylation of olefins from an industrial point of view. Coord. Chem. Rev..

[21] Marciniec, B., Maciejewski, H., Pietraszuk, C., Pawluc, P. *Hydrosilylation: A Comprehensive Review on Recent Advances* (Springer: London, 2009).

[22] Roseblade SJ, Pfaltz A (2007). Iridium-catalyzed asymmetric hydrogenation of olefins. Acc. Chem. Res..

[23] Hopmann KH, Bayer A (2014). Enantioselective imine hydrogenation with iridium-catalysts: reactions, mechanisms and stereocontrol. Coord. Chem. Rev..

[24] Zhou, Q.-L. *Privileged Chiral Ligands and Catalysts* (Wiley‐VCH, Weinheim, 2011).

[25] Privileged ligands. https://www.sigmaaldrich.com/content/dam/sigmaaldrich/docs/Aldrich/Brochure/al_chemfile_v6_n8.pdf (2006).

[26] Precious metals management (pmm). www.platinum.matthey.com. (2019).

[27] Metals market prices, forecasts & analysis. www.metalprices.com. (2019).

[28] Gebbink, R. J. M. K. & Moret, M.-E. *Non-Noble Metal Catalysis Molecular Approaches and Reactions* (Wiley-VCH, 2019).

[29] Beller M (2019). Introduction: first row metals and catalysis. Chem. Rev..

[30] Irrgang T, Kempe R (2019). 3d-Metal catalyzed N- and C-alkylation reactions via borrowing hydrogen or hydrogen autotransfer. Chem. Rev..

[31] Korstanje TJ, van der Vlugt Ivar, Elsevier J, de Bruin CJ (2015). Hydrogenation of carboxylic acids with a homogeneous cobalt catalyst. Science.

[32] Ai W, Zhong R, Liu X, Liu Q (2019). Hydride transfer reactions catalyzed by cobalt complexes. Chem. Rev..

[33] Jerphagnon T, Pizzuti MG, Minnaard AJ, Feringa BL (2009). Recent advances in enantioselective copper-catalyzed 1,4-addition. Chem. Soc. Rev..

[34] Wei D, Darcel C (2019). Iron catalysis in reduction and hydrometalation reactions. Chem. Rev..

[35] Liu W, Sahoo B, Junge K, Beller M (2018). Cobalt complexes as an emerging class of catalysts for homogeneous hydrogenations. Acc. Chem. Res..

[36] Cahiez G, Moyeux A (2010). Cobalt-catalyzed cross-coupling reactions. Chem. Rev..

[37] Chirik P, Morris R (2015). Getting down to earth: the renaissance of catalysis with abundant metals. Acc. Chem. Res..

[38] Gomez S, Peters JA, Maschmeyer T (2002). The reductive amination of aldehydes and ketones and the hydrogenation of nitriles: mechanistic aspects and selectivity control. Adv. Synth. Catal..

[39] Alinezhad H, Yavari H, Salehian F (2015). Recent advances in reductive amination catalysis and its applications. Curr. Org. Chem..

[40] Gross T, Seayad AM, Ahmad M, Beller M (2002). Synthesis of primary amines: first homogeneously catalyzed reductive amination with ammonia. Org. Lett..

[41] Gallardo-Donaire J, Ernst M, Trapp O, Schaub T (2016). Direct synthesis of primary amines via ruthenium‐catalysed amination of ketones with ammonia and hydrogen. Adv. Synth. Catal..

[42] Gallardo-Donaire J (2018). Direct asymmetric ruthenium-catalyzed reductive amination of alkyl–aryl ketones with ammonia and hydrogen. J. Am. Chm. Soc..

[43] Senthamarai T (2018). Simple ruthenium-catalyzed reductive amination enables the synthesis of a broad range of primary amines. Nat. Commun..

[44] Tan X (2018). Asymmetric synthesis of chiral primary amines by ruthenium-catalyzed direct reductive amination of alkyl aryl ketones with ammonium salts and molecular H2. J. Am. Chm. Soc..

[45] Klinkenberg JL, Hartwig JF (2011). Catalytic organometallic reactions of ammonia. Angew. Chem., Int. Ed..

[46] Nakamura Y, Kon K, Touchy AS, Shimizu K-i, Ueda W (2015). Selective synthesis of primary amines by reductive amination of ketones with ammonia over supported Pt catalysts. ChemCatChem.

[47] Liang G (2017). Angew. Chem. Int. Ed..

[48] Komanoya T, Kinemura T, Kita Y, Kamata YK, Hara M (2017). J. Am. Chem. Soc..

[49] Chatterjee M, Ishizaka T, Kawanami H (2016). Reductive amination of furfural to furfurylamine using aqueous ammonia solution and molecular hydrogen: an environmentally friendly approach. Green. Chem..

[50] Reductive amination review. https://erowid.org/archive/rhodium/chemistry/reductive.amination.html (2004).

[51] Hintermann, L. Mignonac Reaction in Comprehensive Organic Name Reactions and Reagents (Wiley, 2010).

[52] Jagadeesh RV (2017). MOF-derived cobalt nanoparticles catalyze a general synthesis of amines. Science.

[53] Hahn G, Kunnas P, de Jonge N, Kempe R (2019). General synthesis of primary amines via reductive amination employing a reusable nickel catalyst. Nat. Catal..

[54] Murugesan K, Beller M, Jagadeesh RV (2019). Reusable nickel nanoparticles-catalyzed reductive amination for selective synthesis of primary amines. Angew. Chem. Int. Ed..

[55] Lawrence, S. A. *Amines: Synthesis, Properties and Applications* (Cambridge University Press, 2004).

[56] Ricci, A. *Amino Group Chemistry: From Synthesis to the Life Sciences* (Wiley-VCH, 2008).

[57] Smith, D. T., Delost, M. D., Qureshi, H. & Njarðarson, J. T. Top 200 Pharmaceutical Products by Retail Sales in 2016. https://njardarson.lab.arizona.edu/sites/njardarson.lab.arizona.edu/files/2016Top200PharmaceuticalRetailSalesPosterLowResV3_0.pdf (2017).

[58] Gunanathan C, Milstein D (2008). Selective synthesis of primary amines directly from alcohols and ammonia. Angew. Chem. Int. Ed..

[59] Imm S, Bähn S, Neubert L, Neumann H, Beller M (2010). An efficient and general synthesis of primary amines by ruthenium‐catalyzed amination of secondary alcohols with ammonia. Angew. Chem., Int. Ed..

[60] Pingen D, Müller C, Vogt D (2010). Direct amination of secondary alcohols using ammonia. Angew. Chem., Int. Ed..

[61] Bähn S (2011). The catalytic amination of alcohols. ChemCatChem.

[62] Müller TE, Beller M (1998). Metal-initiated amination of alkenes and alkynes. Chem. Rev..

[63] Pohlki F, Doye S (2003). The catalytic hydroamination of alkynes. Chem. Soc. Rev..

[64] Müller TE, Hultzsch KC, Yus M, Foubelo F, Tada M (2008). Hydroamination: direct addition of amines to alkenes and alkynes. Chem. Rev..

[65] Ma X, Lei M (2017). Mechanistic insights into the directed hydrogenation of hydroxylated alkene catalyzed by bis(phosphine)cobalt dialkyl complexes. J. Org. Chem..

[66] Yuwen J, Chakraborty S, Brennessel WW, Jones WD (2017). Additive-free cobalt-catalyzed hydrogenation of esters to alcohols. ACS Catal..

[67] Zhang G, Scott BL, Hanson SK (2012). Mild and homogeneous cobalt-catalyzed hydrogenation of C-C, C-O, and C-N bonds. Angew. Chem. Int. Ed..

[68] Zhang G, Vasudevan KV, Scott BL, Hanson SK (2013). Understanding the mechanisms of cobalt-catalyzed hydrogenation and dehydrogenation reactions. J. Am. Chem. Soc..

[69] Frisch, M. J. et al. *Gaussian software, version 09 revision D01*. (Gaussian Inc.: Wallingford, CT, USA, 2009).

[70] Becke AD (1993). Density‐functional thermochemistry. III. The role of exact exchange. J. Chem. Phys..

[71] Schäfer A, Huber C, Ahlrichs R (1994). Fully optimized contracted Gaussian basis sets of triple zeta valence quality for atoms Li to Kr. J. Chem. Phys..

[72] Wohlfarth, C. *Static Dielectric Constants of Pure Liquids and Binary Liquid Mixtures* 17 (Springer, 2015).

[73] Marenich AV, Cramer CJ, Truhlar DG (2009). Universal solvation model based on solute electron density and on a continuum model of the solvent defined by the bulk dielectric constant and atomic surface tensions. J. Phys. Chem. B.

[74] Grimme S, Ehrlich S, Goerigk L (2011). Effect of the damping function in dispersion corrected density functional theory. J. Comput. Chem..

