# Supplementary material for: Homogeneous cobalt-catalyzed reductive amination for synthesis of functionalized primary amines
Source: Nat Commun. 2019 Nov 29;10:5443. doi: 10.1038/s41467-019-13351-7 (PMC6884468; doi:10.1038/s41467-019-13351-7)
Supplement: Supplementary file 2 — Supplementary Information [file 41467_2019_13351_MOESM2_ESM.pdf]

Supplementary Information

Homogeneous cobalt-catalyzed reductive amination for synthesis of functionalized primary amines

Murugesan et al

Supplementary methods

Crystal data for complex A and complex B

Crystal data for complex A: $C_{68}H_{66}B_2CoF_8P_6$, M = 1301.57, monoclinic, space group $C2/c$, a = 27.9413(8), b = 21.6226(6), c = 25.4121(6) Å, β = 114.9250(14)°, V = 13923.1(7) Å^3, T = 150(2) K, Z = 8, 101327 reflections measured, 12330 independent reflections (R_{int} = 0.1008), final R values ($I > 2\sigma(I)$): R_1 = 0.0483, wR_2 = 0.1182, final R values (all data): R_1 = 0.0656, wR_2 = 0.1291, 658 parameters. Contributions of co-crystallized solvent molecules were removed from the diffraction data with PLATON/ SQUEEZE (Spek, A. L. *Acta Cryst.* **2015**, *C*71, 9).

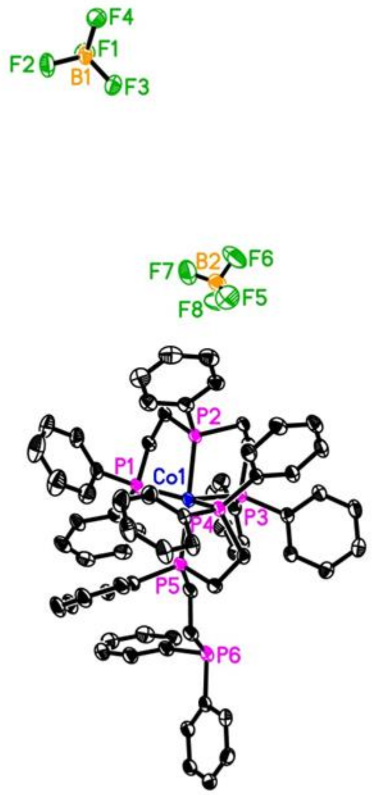

ORTEP representation of complex A.

Displacement ellipsoids corresponds to 30% probability. Hydrogen atoms are omitted for clarity. Selected bond lengths [Å] and angles [°]: Co1-P2-2.2477(9), Co1-P1 2.2672(8), Co1-P4 2.2776(8), Co1-P5 2.2789(9), Co1-P3 2.4808(9); P2-Co1-P1 83.64(3), P2-Co1-P4 93.34(3), P1-Co1-P4 152.13(4), P2-Co1-P5 175.34(4), P1-Co1-P5 97.91(3), P4-Co1-P5 83.23(3), P2-Co1-P3 83.38(3), P1-Co1-P3 102.31(3), P4-Co1-P3 104.86(3), P5-Co1-P3 100.52(3).

HRMS for Complex A: HRMS (ESI-TOF, m/z): Calcd for C68H66CoP6 $[M]^{2+}$ 563.6466; found 563.6453.

ESI-TOF Accurate Mass Report Page 1

Results file: E:\Projects\1902.PRO\SampleDB\1902.rpt
Last modified: Tuesday, February 26, 2019 15:04:18

Sample Summary:

Sample	File	Sample Name	User	Target	Formula	Expected Mass	Observed Mass	Error PPM	Error mDa	
245	19022608	KM22-446A	Kathir	1127.2922	C68H66CoP6	563.6466	563.6453	-2.3	-1.3	M+2

ESI-TOF Accurate Mass Report Page 2

File:19022608
Vial:1:C,3
Description:MeOH/0.1%HCOOH in H2O 9:1

Sample Name:KM22-446A
Date:26-Feb-2019

UserName:Kathir
Time:14:57:27

Sample Report:

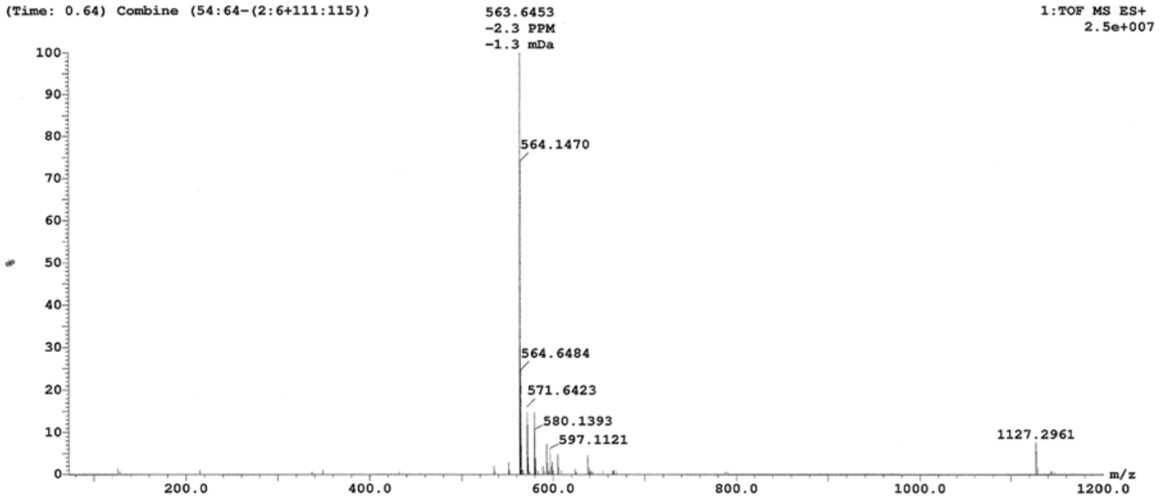

Supplementary Figure 1. HRMS of paramagnetic complex-A

190531.f324.10.fid
Kathir KM22-446
PROTON CDCl3 {C:\Bruker\TopSpin3.6.0} 1905 24

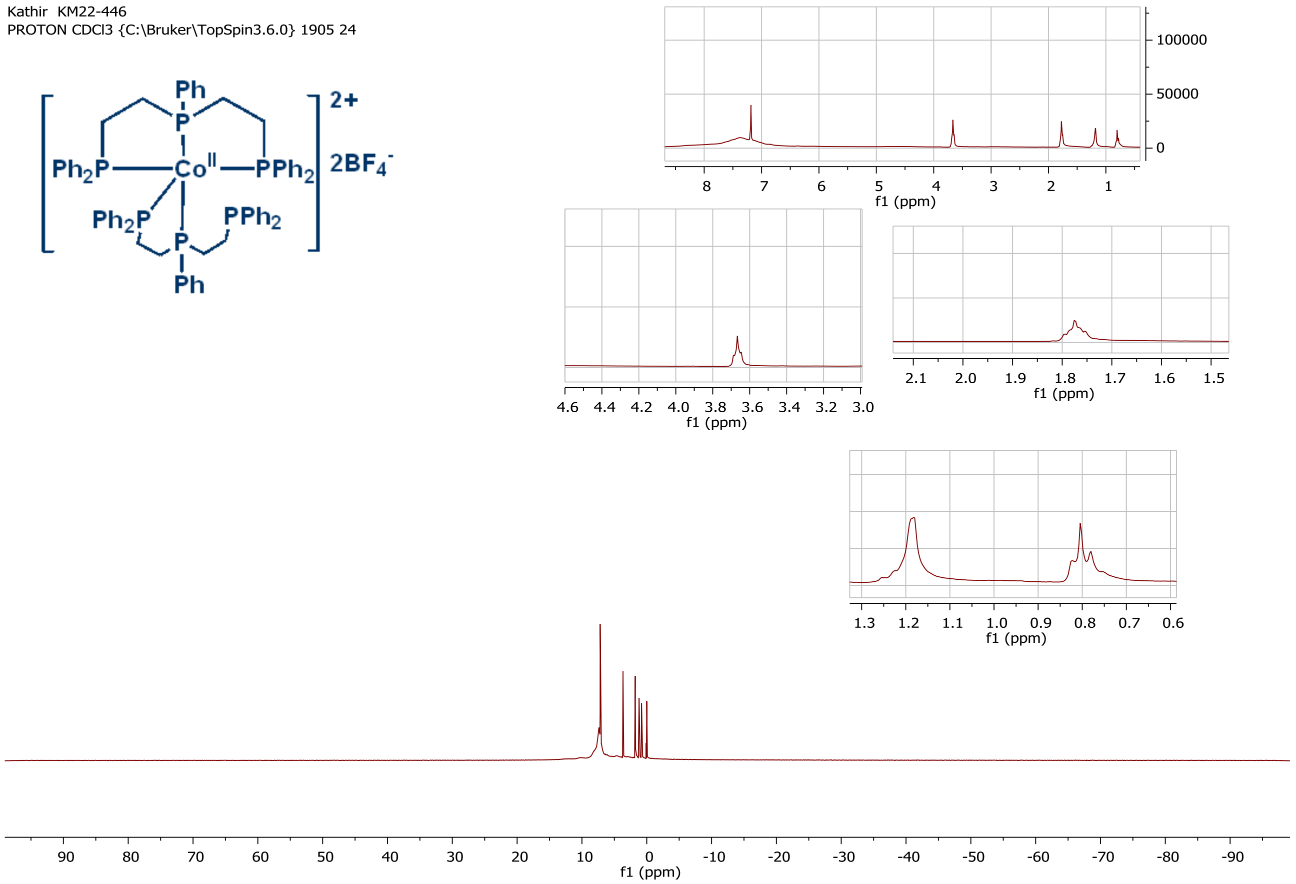

Supplementary Figure 2. ^{1}H NMR (**300 MHz, Chloroform-*d***) of paramagnetic complex-A

190606.40a.3.fid
KM 22-446 // Co complex detail

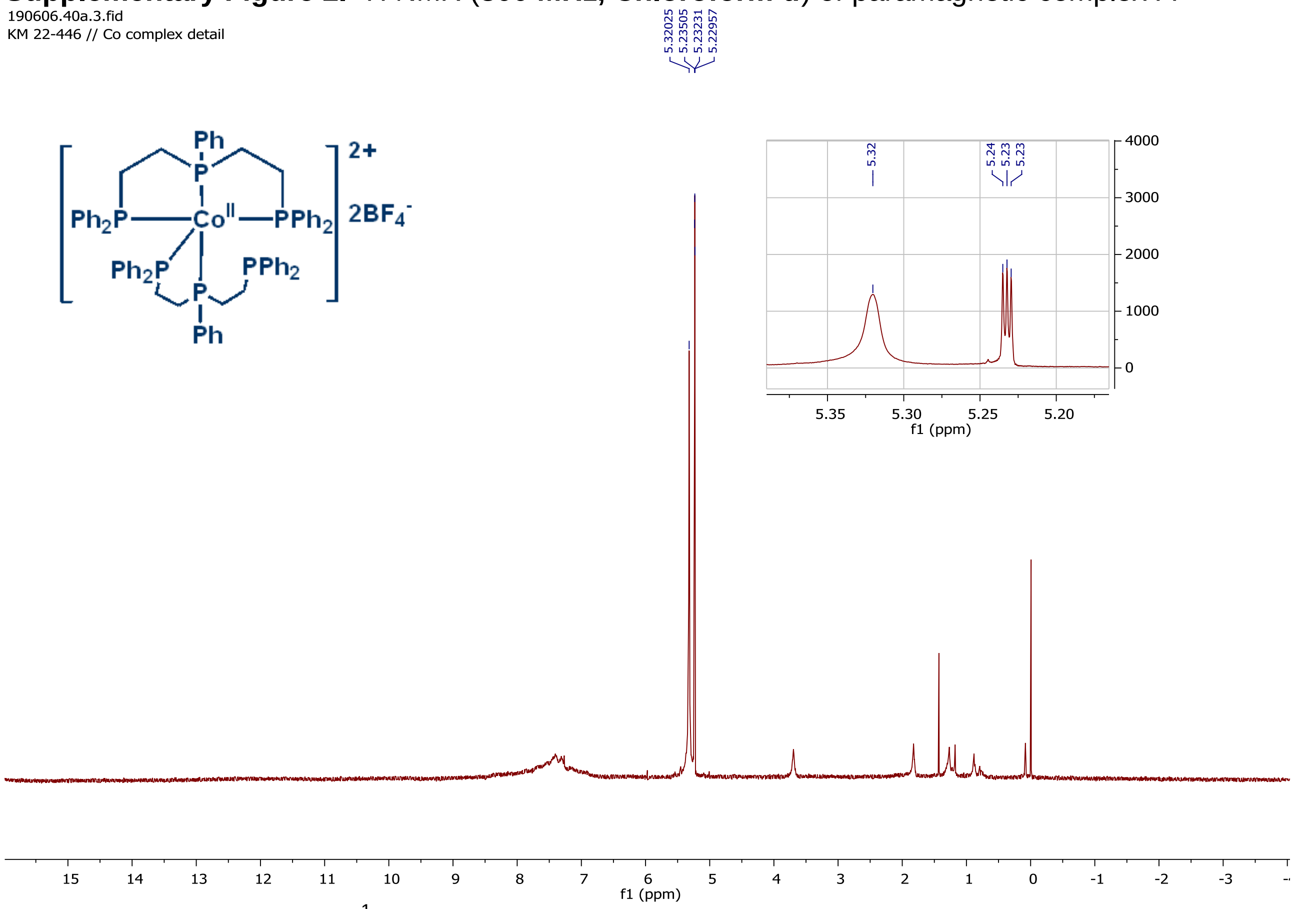

Supplementary Figure 3. ^{1}H NMR (**400 MHz, Dichloromethane-*d*$_2$**) of complex-A by The Evans Method

Paramagnetic susceptibility measurement preliminary results:

Entry	Measurement	Data
1	Observed paramagnetic shift	0,088 ppm (^{1}H)
2	Mass susceptibility χ_m	8,92·10^{-10} m^3/kg
3	Molar susceptibility χ_M	1,16·10^{-9} m^3/mol
4	Number of unpaired electron	1

Crystal data for complex B: $C_{70}H_{72}B_2CoF_8O_3P_6$, M = 1379.64, triclinic, space group $P\bar{1}$, a = 10.8798(2), b = 14.9770(3), c = 21.8175(5) Å, α = 102.2273(9), β = 92.1113(9), γ = 107.3687(9)°, V = 3296.88(12) Å^3, T = 150(2) K, Z = 2, 39417 reflections measured, 11604 independent reflections (R_{int} = 0.0409), final R values ($I > 2\sigma(I)$): R_1 = 0.0554, wR_2 = 0.1488, final R values (all data): R_1 = 0.0631, wR_2 = 0.1559, 742 parameters.

CCDC 1897492-1897493 contain the supplementary crystallographic data for this paper. These data are provided free of charge by The Cambridge Crystallographic Data Centre.

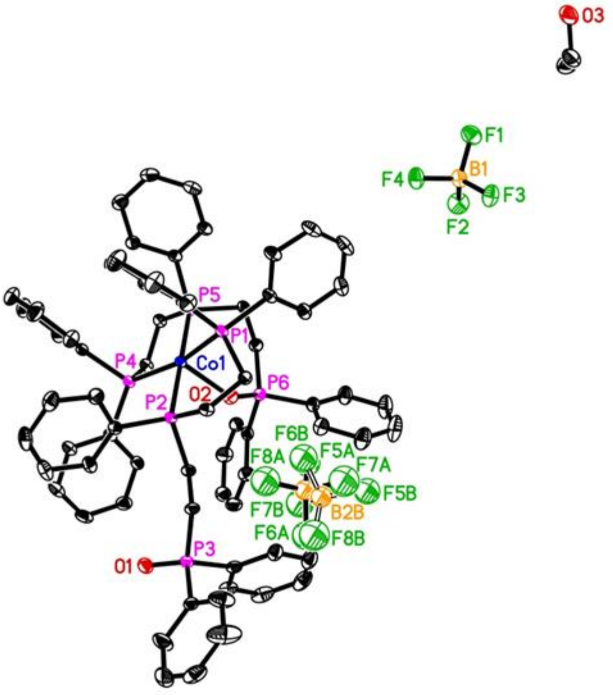

ORTEP representation of complex B.

Displacement ellipsoids corresponds to 30% probability. Hydrogen atoms are omitted for clarity. One BF_4 anion is disordered over two sites with occupancies of 0.608(5) : 0.392(5). Lower occupancy sites are depicted with open bonds. Selected bond lengths [Å] and angles [°]: Co1-O2 2.137(2), Co1-P4 2.2507(9), Co1-P5 2.2509(8), Co1-P1 2.2732(9), Co1-P2 2.2811(8), P6-O2 1.507(2), P3-O1 1.485(3); O2-Co1-P4 97.44(8), O2-Co1-P5 90.71(6), P4-Co1-P5 82.86(3), O2-Co1-P1 96.58(7), P4-Co1-P1 165.98(4), P5-Co1-P1 97.01(3), O2-Co1-P2 89.18(6), P4-Co1-P2 96.71(3), P5-Co1-P2 179.54(3), P1-Co1-P2 83.44(3).

HRMS for Complex B: HRMS (ESI-TOF, m/z): Calcd for C68H66CoP6O2 $[M]^{2+}$ 579.6415; found 579.6400.

ESI-TOF Accurate Mass Report Page 1

Results file: E:\Projects\1902.PRO\SampleDB\1902.rpt
Last modified: Tuesday, February 26, 2019 14:52:06

Sample Summary:

Sample	File	Sample Name	User	Target	Formula	Expected Mass	Observed Mass	Error PPM	Error mDa	
250	19022607	KM22-112A	Kathir	1127.2922	C68H66CoP6	563.6466				
				1159.2821	C68H66CoP6O2	579.6415	579.6400	-2.6	-1.5	M2+

ESI-TOF Accurate Mass Report Page 2

File:19022607
Vial:1:C,1
Description:MeOH/0.1%HCOOH in H2O 9:1

Sample Name:KM22-112A
Date:26-Feb-2019

UserName:Kathir
Time:14:47:44

Sample Report:

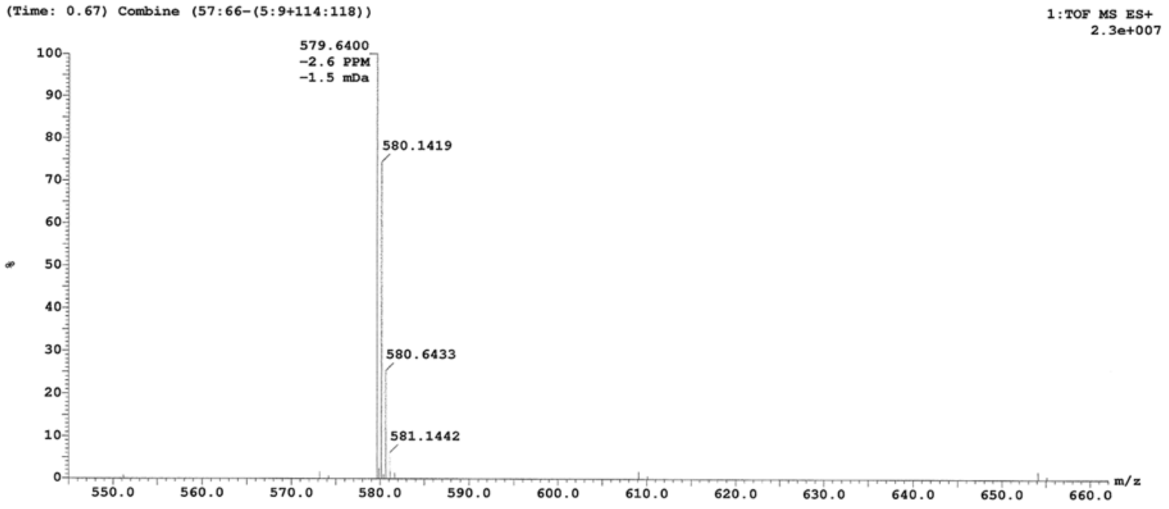

Supplementary Figure 4. HRMS of complex-B

Supplementary Tables

Supplementary Table 1. Reductive amination of 4-methylbenzaldehyde with cobalt-phosphine complexes.

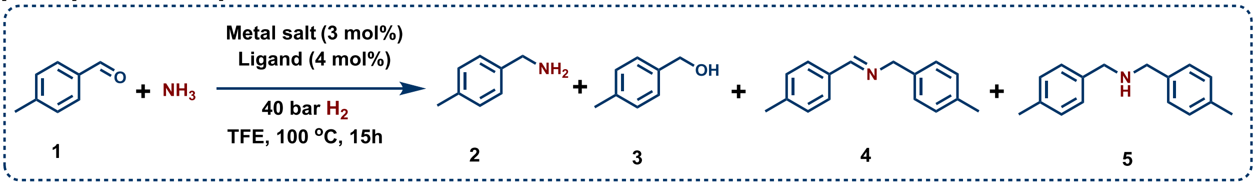

Entry	Ligand	Conv. (%)	Yield of 2 (%)	Yield of 3 (%)	Yield of 4 (%)	Yield of 5 (%)
1[a]	L7	>99	96	-	2	-
2[b]	L7	60	10	-	48	-
3[c]	L7	90	60	-	28	-
4[d]	L7	95	88	-	5	-
5[e]	-	20	-	-	18	-
6[f]	L7	-	-	-	-	-
7[g]	L7	>99	-	98	-	-
8[h]	-	>99	96	-	2	-
9[i]	-	72	-	-	70	-

Reaction conditions: [a]0.5 mmol 4-methylbenzaldehyde, 3 mol% $Co(BF_4)_2 \cdot 6H_2O$, 4 mol% triphos (**L7**), 5-7 bar NH_3, 40 bar H_2, 2 mL trifluoroethanol (TFE), 100 °C, 15 h, GC yields using n-hexadecane as standard. [b]Same as ‘a’ with 20 bar H_2. [c]Same as ‘a’ at 80 °C. [d]Same as ‘a’ with 2 mol% of catalyst. [e]Same as ‘a’ without ligand (**L7**). [f]Same as ‘a’ without ammonia. [g]Same as ‘a’ without ammonia and using 10 mol% of *t*-BuOK. [h]Same as ‘a’ with 3 mol% of complex **A** instead of $Co(BF_4)_2 \cdot 6H_2O$ /triphos (**L7**). [i]Same as ‘a’ with 3 mol% of complex **B** instead of $Co(BF_4)_2 \cdot 6H_2O$ /triphos (**L7**).

Supplementary Table 2. Influence of solvents in reductive amination of 4-methylbenzaldehyde.

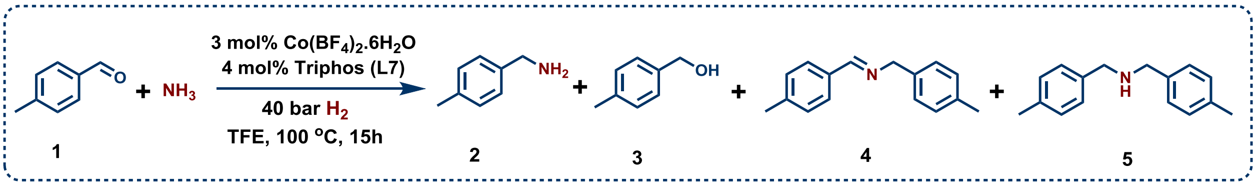

Entry	Solvent	Conv. (%)	Yield of 2 (%)	Yield of 3 (%)	Yield of 4 (%)	Yield of 5 (%)
1	Toluene	10	-	-	8	-
2	THF	20	-	-	18	-
3	*t*-Amyl alcohol	40	-	-	35	-
4	*t*-BuOH	2	-	-	1	-
5	IPA	80	60	-	18	-
6	MeOH	90	80	-	8	-
7	Trifluoroethanol	>99	96	-	2	-

Reaction conditions: 0.5 mmol 4-methylbenzaldehyde, 3 mol% $Co(BF_4)_2{\cdot}6H_2O$, 4 mol% linear triphos (**L7**), 5-7 bar NH_3, 40 bar H_2, 2 mL solvent, 100 °C, 15 h, GC yields using n-hexadecane as standard.

Supplementary Table 3. Reductive amination of 4-methylbenzaldehyde with Co- salts.

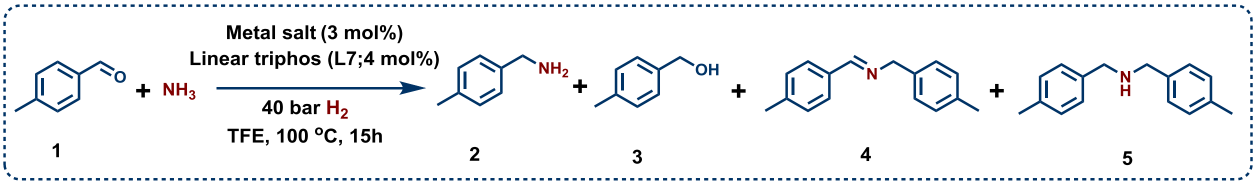

Entry	Cobalt salt	Ligand	Conv. (%)	Yield of 2 (%)	Yield of 3 (%)	Yield of 4 (%)	Yield of 5 (%)
1	$Co_2(CO)_8$	L7	90	68	-	20	-
2	$Co(PPh_3)_3Cl$	L7	95	88	-	5	-
3	$Co(BF_4)_2{\cdot}6H_2O$	L7	>99	96	-	2	-
4	$Co(acac)_3$	L7	70	-	-	68	-
5	$Co[(NH_3)6]Cl_3$ Werner complex	L7	10	-	-	8	-

Reaction conditions: 0.5 mmol 4-methylbenzaldehyde, 3 mol% Cobalt salt, 4 mol% linear triphos (**L7**), 5-7 bar NH_3, 40 bar H_2, 2 mL trifluoroethanol (TFE), 100 °C, 15 h, GC yields using n-hexadecane as standard.

Catalytic poisoning test

Supplementary Table 4. Catalyst poisoning experiments using Hg and PPh_3.

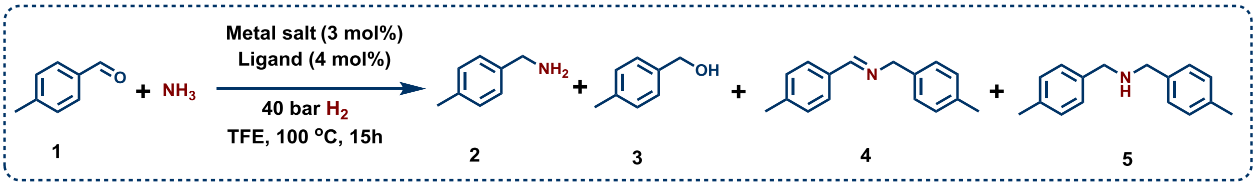

Entry	Catalytic poison	Conv. (%)	Yield of 2 (%)	Yield of 3 (%)	Yield of 4 (%)	Yield of 5 (%)
1[a]	Hg (2 eq)	>99	94	-	4	-
2[a]	PPh_3 (50 mol%)	>99	93	-	5	-
3[b]	Hg (2 eq)	>99	96	-	2	-
4[b]	PPh_3 (50 mol%)	>99	95	-	3	-

Reaction conditions: [a]0.5 mmol 4-methylbenzaldehyde, 3 mol% complex **A**, 5-7 bar NH_3, 40 bar H_2 2 mL trifluoroethanol (TFE), 100 °C, 15 h, GC yields using n-hexadecane as standard. [b]same as 'a' using 120°C for 24 h.

Comparison of activities and selective of different catalysts

Comparison reactivity and selectivity of Co-triphos system with previously reported Co-nanoparticles and Ru-based complexes for the reductive amination to prepare primary amines

A) This work with Co-triphos catalyst

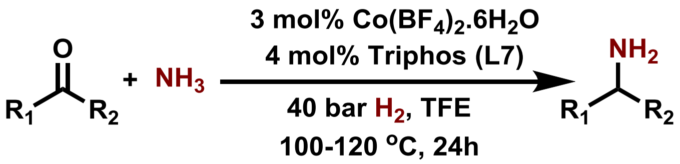

B) Using previously reported Co-nanoparticles (ref: *Science,* 2017, 358, 326-332)

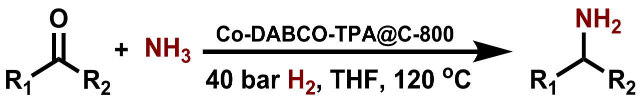

B) Using Ru and Rh-based homogeneous catalysts

1	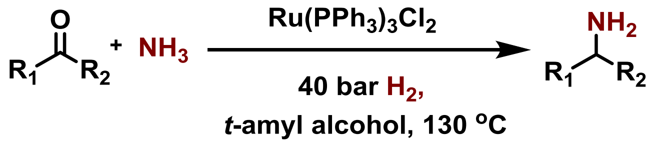	***Nature Commun.*, 2018, *9*, 4123.**
2	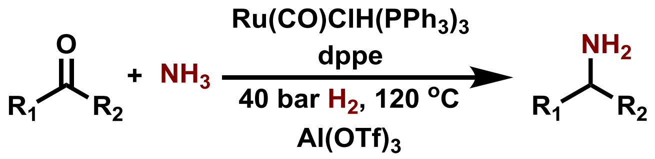	***Adv. Synth. Catal*. 2016, *358*, 358.**
3	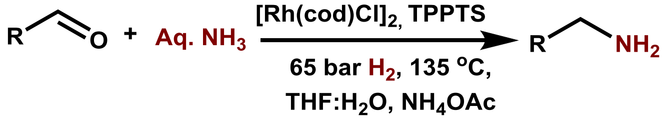	***Org. Lett.*, 2002, *12*, 2055.**

Supplementary Table 5. Comparison of Co-triphos with previously reported Ru-complexes.

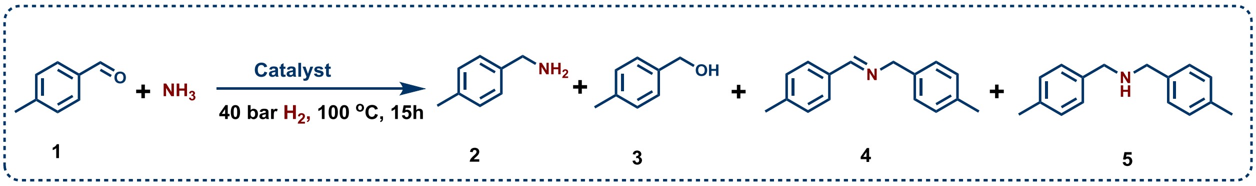

Entry	Catalyst	Conv. (%)	Yield of 2 (%)	Yield of 3 (%)	Yield of 4 (%)	Yield of 5 (%)
1[A]	Co-triphos(2019) This work	>99	96	-	2	-
2 [B]	Ru (2018) *Nature Commun.*, **2018**, 9, 4123	72	-	-	70	-
3 [C]	Ru (2016) *Adv. Synth. Catal.* **2016,** *358, 358*	>99	30	-	68	-

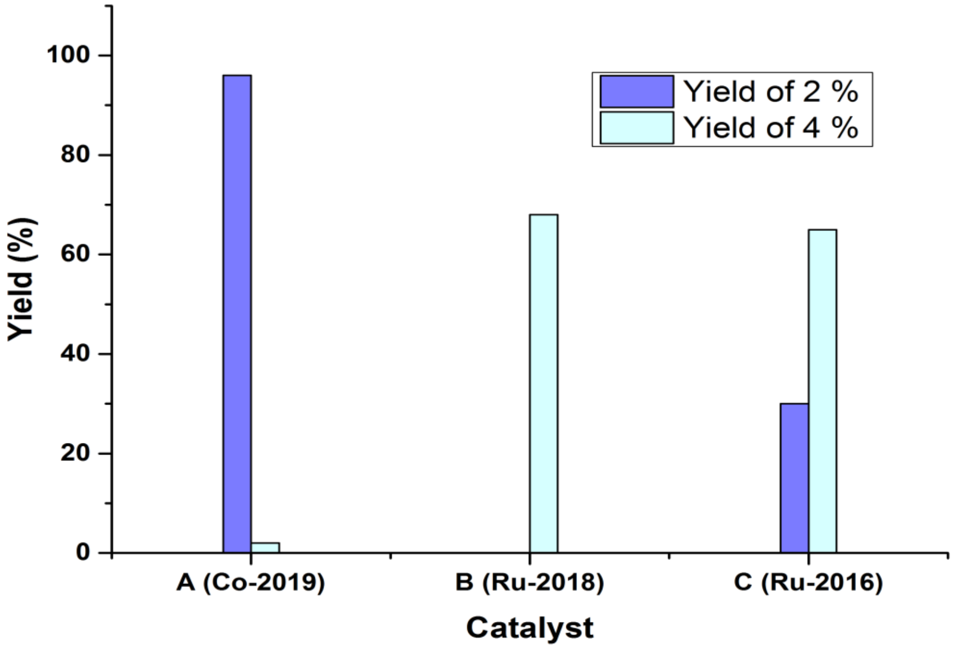

Reaction conditions: [A]0.5 mmol 4-methyl benzaldehyde, 3 mol% $Co(BF_4)_2 \cdot 6H_2O$, 4 mol% triphos (L7), 5-7 bar NH_3, 40 bar H_2, 2 mL trifluoroethanol (TFE), 100 ^oC, 15 h. [B]0.5 mmol 4-methyl benzaldehyde, 2 mol% $RuCl_2(PPh_3)_3$, 5-7 bar NH_3, 40 bar H_2, 2 mL *t*-amyl alcohol, 100 ^oC, 15 h. [C]0.5 mmol 4-methyl benzaldehyde, 1 mol% $Ru(Co)ClH(PPh_3)_3$, 1.1 mol% dppe (L4), $Al(OTf)_3$10 mol%, 5-7 bar NH_3, 40 bar H_2, 2 mL Toluene, 100 ^oC, 15 h GC yields using n-hexadecane as standard. Products 3 and 5 are not detected.

DFT calculation

Cationic hydride $[L7CoH]^+$ as active catalyst (Supplementary Figure 5): For Ph-CH=NH hydrogenation, the hydride mechanism starting with $[L7CoH]^+$ species (**I**) in gas phase, in TFE solution and in TFE solution including GD3BJ dispersion was computed for comparison.

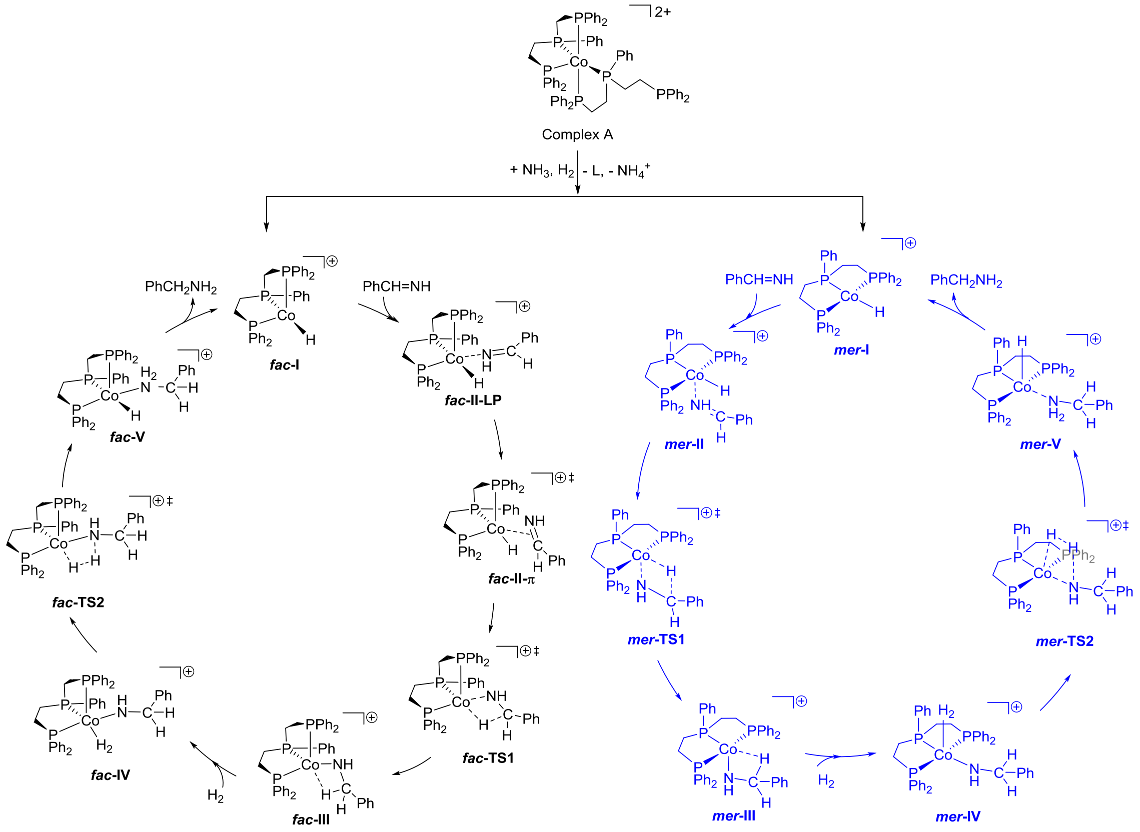

Supplementary Figure 5. Proposed mechanism for Ph−CH=NH hydrogenation by using $[L7CoH]^+$ as active catalyst through the *fac*- and *mer*-route.

Cationic hydride $[L7CoH]^+$ catalyzed cycle in gas phase (Supplementary Figure 6): As shown in Supplementary Figure 5, the proposed active catalyst has two conformations, i.e.; ***mer*-I** and ***fac*-I**; and therefore we computed the reaction mechanisms using both conformers. In gas phase, complex ***mer*-I** is more stable than complex ***fac*-I** by 31 kJ/mol. Starting from the ***mer*-I**, the coordination of Ph−CH=NH to form the σ-complex is endergonic by 1 kJ/mol for ***mer*-II-LP**, while exergonic by 4 kJ/mol for ***fac*-II-LP**. The formation of π-complex ***fac*-II-π** is endergonic by 40 kJ/mol, while ***mer*-II-π** could not be located. The Gibbs free energy barrier of Ph−CH=NH insertion into **I** is 112 and 39 kJ/mol for ***mer*-TS1** and ***fac*-TS1**, respectively. Thus, the Ph−CH=NH insertion into **I** from π-complex ***fac*-II-π** is barrier-less. The formation of intermediate **III** with agostic interaction is endergonic by 108 and 24 kJ/mol for ***mer*-III** and ***fac*-III**, respectively.

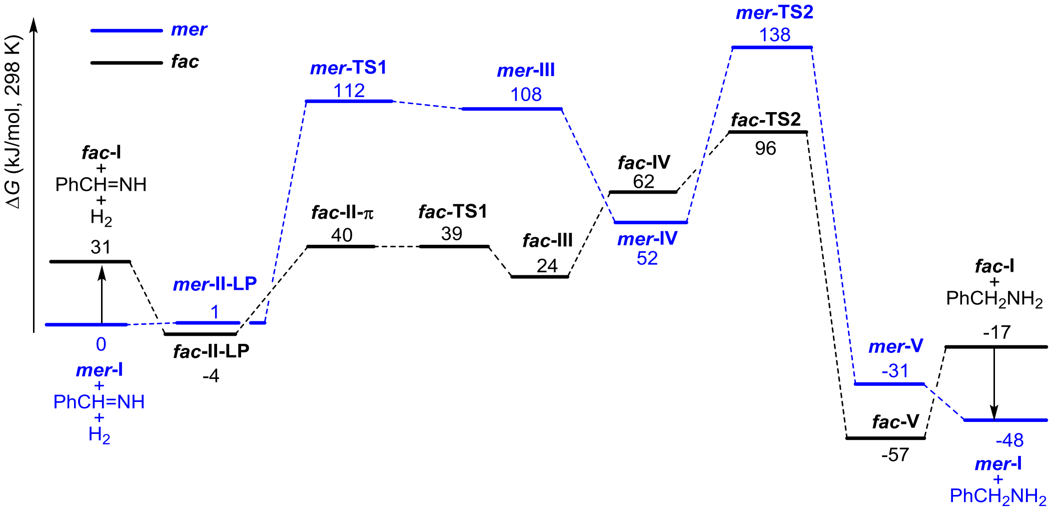

Supplementary Figure 6. Gibbs free energy surface for Ph−CH=NH hydrogenation by using $[L7CoH]^+$ as catalyst through ***fac-*** and ***mer-***route in gas phase at B3PW91/TZVP level.

In the second step, H_2 coordination to form **IV** is endergonic by 52 and 62 kJ/mol for ***mer*-IV** and ***fac*-IV**, respectively. The final step of H_2 metathesis has Gibbs free energy barrier of 138 and 96 kJ/mol for ***mer*-TS2** and ***fac*-TS2**, respectively. The formation of **V** is exergonic by 31 and 57 kJ/mol for ***mer*-V** and ***fac*-V**, respectively. The release of amine from complex ***mer*-V** and ***fac*-V** with the regeneration of ***mer*-I** is exergonic by 48 kJ/mol. The transition state of H_2 metathesis represents the highest point on the Gibbs free energy surface and is therefore rate-determining transition state. On the basis of whole Gibbs free energy surface, the apparent Gibbs free energy barrier is 96 kJ/mol and 138 kJ/mol for ***fac-*** and ***mer***-route, respectively. Under the consideration of the isomerization of both conformers and the Curtin–Hammett principle which states that for a reaction with two active intermediate under rapid equilibrium interconversion with low barrier and each intermediate has its own rate-determining transition state, the energy difference of the two rate-determining transition states rather than the equilibrium distribution of the two intermediates determines the product distribution (or selectivity), it is therefore to conclude that the ***fac***-route with lower apparent barrier is more preferred kinetically.

Cationic hydride [L7CoH]$^+$ catalyzed cycle in TFE solution (Supplementary Figure 7): As shown in Supplementary Figure 7, the potential free energy surfaces in gas phase and in solution are very similar. In TFE solution, complex ***mer*-I** is more stable than complex ***fac*-I** by 33 kJ/mol. Starting from the ***mer*-I**, the coordination of Ph−CH=NH to form the σ-complex is endergonic by 28 and 32 kJ/mol for ***mer*-II-LP** and ***fac*-II-LP**, respectively. The formation of π-complex ***fac*-II-π** is endergonic by 72 kJ/mol. The Gibbs free energy barrier of Ph−CH=NH insertion into **I** is 130 and 71 kJ/mol for ***mer*-TS1** and ***fac*-TS1**, respectively. The formation of intermediate **III** with agostic interaction is endergonic by 121 and 50 kJ/mol for ***mer*-III** and ***fac*-III**, respectively.

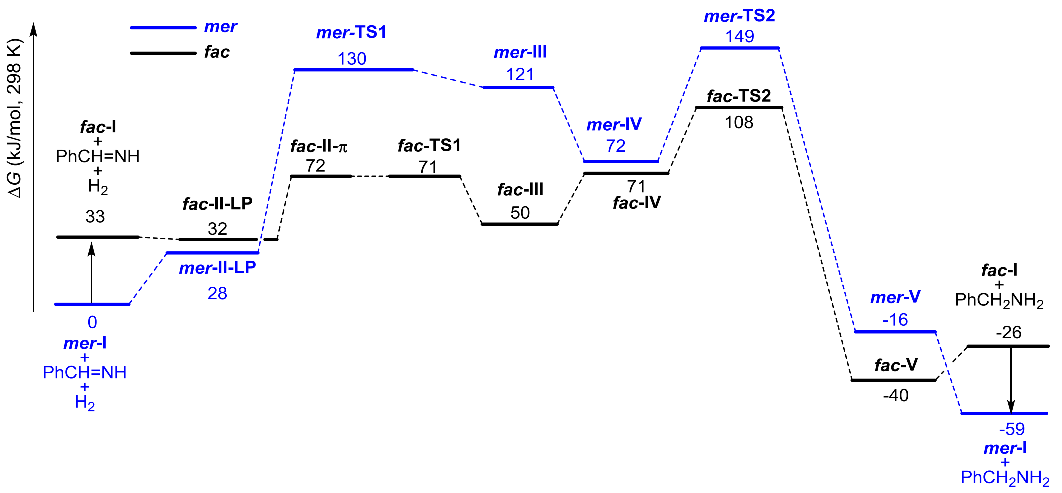

Supplementary Figure 7. Gibbs free energy surface for Ph−CH=NH hydrogenation by using [L7CoH]$^+$ as catalyst through ***fac*-** and ***mer*-route** in TFE at B3PW91-SMD/Def2-TZVP//B3PW91/TZVP level.

In the second step, H_2 coordination to form **IV** is exergonic by 72 and 71 kJ/mol for ***mer*-IV** and ***fac*-IV**, respectively. The final step of H2 metathsis has Gibbs free energy barrier of 149 and 108 kJ/mol for ***mer*-TS2** and ***fac*-TS2**, respectively. The formation of **V** is exergonic by 16 and 40 kJ/mol for ***mer*-V** and ***fac*-V**, respectively. The release of amine from complex ***mer*-V** and ***fac*-V** with the regeneration of ***mer*-I** is exergonic 59 kJ/mol. Similar as in gas phase, the transition state of H_2 metathesis represents the highest point on the Gibbs free energy surface and is therefore rate-determining transition state. On the basis of whole Gibbs free energy surface, the apparent Gibbs free energy barrier is 108 and 149 kJ/mol for ***fac*-** and ***mer*-route**, respectively. Therefore, the ***fac*-route** is more favored kinetically.

Cationic hydride [L7CoH]$^+$ catalyzed cycle in TFE solution with dispersion (Supplementary Figure 8): Furthermore, the dispersion energies have been included by single point calculation. As shown in Supplementary Figure 8, the potential free energy surfaces in solution including dispersion correction shows the same trend and shape as found in gas phase and in solution, but they differ quantitatively in numbers.

It is found that the ***mer*-I** is more stable than the ***fac*-I** by 22 kJ/mol, and the ***fac***-route has lower apparent barrier than ***mer***-route (29 vs. 65 kJ/mol). This agrees qualitatively with the results in Supplementary Figure 6; but they differ quantitatively, especially in the rate-determining energy, i.e.; from 108 kJ/mol without dispersion correction to 29 kJ/mol with dispersion correction. On the basis of the applied reaction conditions (100-120°C, 40 bar H_2 and 15-24 h reaction time), GD3BJ correction underestimates extremely the barriers and that without dispersion correction is very reasonably.

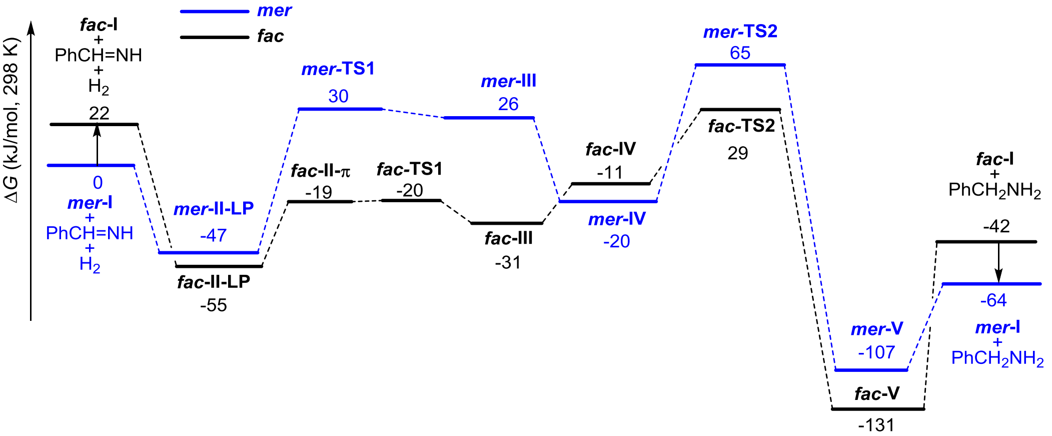

Supplementary Figure 8. Gibbs free energy surface for Ph−CH=NH hydrogenation by using $[L7CoH]^+$ as catalyst through ***fac-*** and ***mer***-route in TFE with GD3BJ dispersion at B3PW91-SMD-D3/Def2-TZVP//B3PW91/TZVP level.

Di-cationic $[L7Co(H)_2]^{2+}$ as active catalyst: Alternatively, we computed the di-cationic complex from the dissociation of one ligand and the addition of one H_2 in gas phase, in TFE solution with and without GD3BJ dispersion. As shown in Supplementary Figure 9, the proposed active catalyst has two conformations, i.e.; ***mer*-I** and ***fac*-I**; and therefore we computed the reaction mechanisms using both conformers.

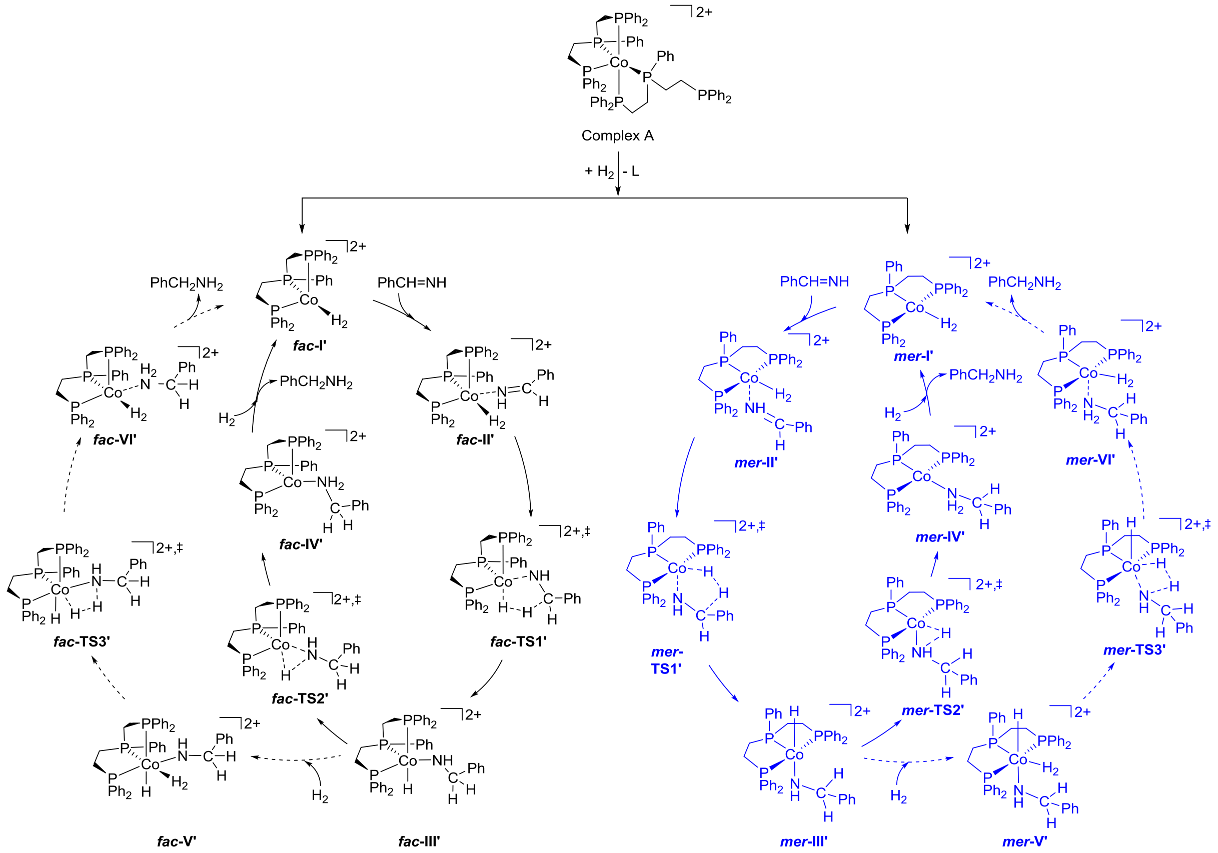

Supplementary Figure 9. Proposed reaction mechanism for for Ph−CH=NH hydrogenation by using $[L7Co(H_2)]^{2+}$ as active catalyst through the *fac* and *mer* routes.

Di-cationic $[L7Co(H)_2]^{2+}$ catalyzed cycle in gas phase (Supplementary Figure 10): Starting from ***mer*-[L7Co]$^{2+}$**, H_2 coordination to form di-cationic $[L7Co(H)_2]^{2+}$ **I'** complex is exergonic by 24 kJ/mol for ***mer*-I'**, while endergonic by 23 kJ/mol for ***fac*-I'**. Therefore, we used ***mer*-I'** as reference and both ***fac*-I'** and ***mer*-I'** based catalytic cycles were calculated.

Starting from the ***mer*-I'**, the coordination of Ph−CH=NH to form the σ-complex **II'** is exergonic by 10 and 42 kJ/mol for ***mer*-I'** and ***fac*-I'**, respectively. The Gibbs free energy barrier of Ph−CH=NH insertion into **II'** is 170 and 128 kJ/mol for ***mer-*** and ***fac*-**route, respectively. The formation of intermediate **III'** is endergonic by 76 and 104 kJ/mol for ***mer*-III'** and ***fac*-III'**, respectively.

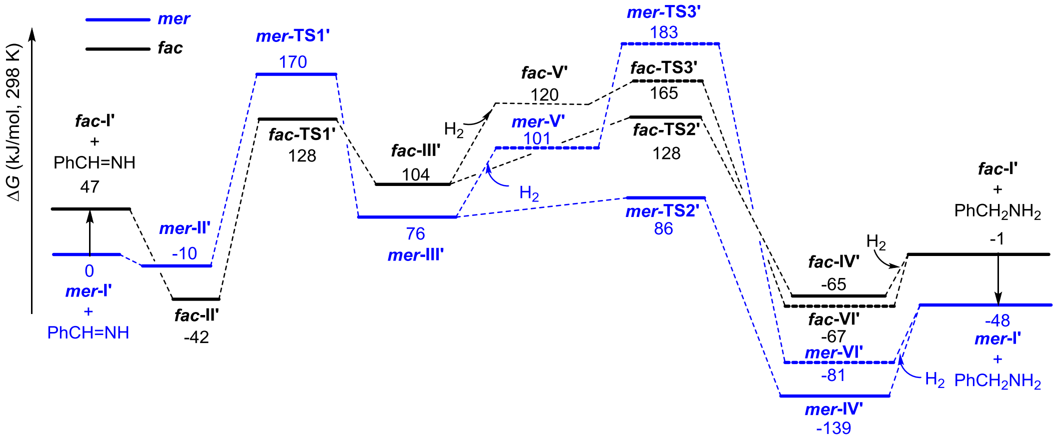

Supplementary Figure 10. Gibbs free energy surface for Ph−CH=NH hydrogenation by using $[L7CoH_2]^{2+}$ as catalyst through *fac*- and *mer*-route in gas phase at B3PW91/TZVP level

In the second step, the $PhCH_2NH_2$ can be generated through either the direct route of reductive elimination or the metathesis of the second molecular of H_2. For the direct reductive elimination route, the reductive elimination through transition state of **TS2'** has an energy barrier of 86 and 128 kJ/mol for ***mer*** and ***fac***-route, respectively. The formation of amine complex **IV'** is exergonic by 139 and 65 kJ/mol for ***mer*-IV'** and ***fac*-IV'**, respectively. The release of $PhCH_2NH_2$ from complex **IV'** and coordination of H_2 with the regeneration of ***mer*-I'** is exergonic by 48 kJ/mol.

For the H_2 metathesis route, the H_2 coordination to the amine intermediate to form **V'** is endergonic by 101 and 120 kJ/mol for ***mer*-V'** and ***fac*-V'**. The final step of H_2 metathesis has Gibbs free energy barrier of 183 and 165 kJ/mol for ***mer*-TS3'** and ***fac*-TS3'**, respectively. The formation of amine complex **VI'** is exergonic by 81 and 67 kJ/mol for ***mer*-VI'** and ***fac*-VI'**, respectively. The release of amine from complex **VI'** with the regeneration of ***mer*-I'** is endergonic by 48 kJ/mol. Therefore, the direct reductive elimination route is more favorable than the H_2 metathesis route. The transition state of the first step represents the highest point on the Gibbs free energy surface and the apparent Gibbs free energy barrier is 170 and 128 kJ/mol for ***mer*-** and ***fac***-route, respectively. It is noted that these barriers are much higher than those of the mono-cationic complex by 32 kJ/mol; and such di-cationic routes are unlikely.

Di-cationic $[L7Co(H_2)]^{2+}$ catalyzed cycle in TFE (Supplementary Figure 11): Nevertheless, the di-cationic routes in solution have been also computed for comparison. Starting from ***mer*-[L7Co]$^{2+}$**, H_2 coordination is exergonic by 5 kJ/mol for ***mer*-I'**, while endergonic by 47 kJ/mol for ***fac*-I'**. Similar as in gas phase, we also use ***mer*-I'** as reference and both ***fac*-I'** and ***mer*-I'** based catalytic cycles were calculated.

Starting from the ***mer*-I'**, the coordination of Ph−CH=NH to form the σ-complex **II'** is endergonic by 56 and 35 kJ/mol for ***mer*-II'** and ***fac*-II'**, respectively. The Gibbs free energy barrier of Ph−CH=NH insertion into **II'** is 248 and 200 kJ/mol for ***mer*-** and ***fac***-route, respectively. The formation of intermediate **III'** is endergonic by 144 and 176 kJ/mol for ***mer*-II'** and ***fac*-II'**, respectively.

In the second step, the $PhCH_2NH_2$ can be directly generated through either the reductive elimination or metathesis of the second molecular of H_2. For the direct route, the reductive elimination through transition state of **TS2'** has an energy barrier of 154 and 212 kJ/mol for ***mer*** and ***fac*** route, respectively. The formation of amine complex **IV'** is exergonic by 89 and 6 kJ/mol for ***mer*-IV'** and ***fac*-IV'**, respectively. The release of $PhCH_2NH_2$ from complex **IV'** and coordination of H_2 with the regeneration of ***mer*-I'** is exergonic by 59 kJ/mol. For the H_2 metathesis route, the H_2 coordination to the amine intermediate to form **V'** is endergonic by 158 and 180 kJ/mol for ***mer*-V'** and ***fac*-V'**, respectively. The final step of hydrogenolysis has Gibbs free energy barrier of 242 and 236 kJ/mol for ***mer*-TS3'** and ***fac*-TS3'**, respectively. The formation of amine complex **VI'** is exergonic by 29 and 23 kJ/mol for ***mer*-VI'** and ***fac*-VI'**, respectively. The release of amine from complex **VI'** with the regeneration of ***mer*-I'** is exergonic by 59 kJ/mol. In TFE solution, the direct route is more favorable than the H_2 metathesis route, and the apparent Gibbs free energy barrier is 248 and 212 kJ/mol for ***mer-*** and ***fac***-route, respectively. It is noted that these barriers are much higher than those of the mono-cationic complex by more than 100 kJ/mol and can be ruled out.

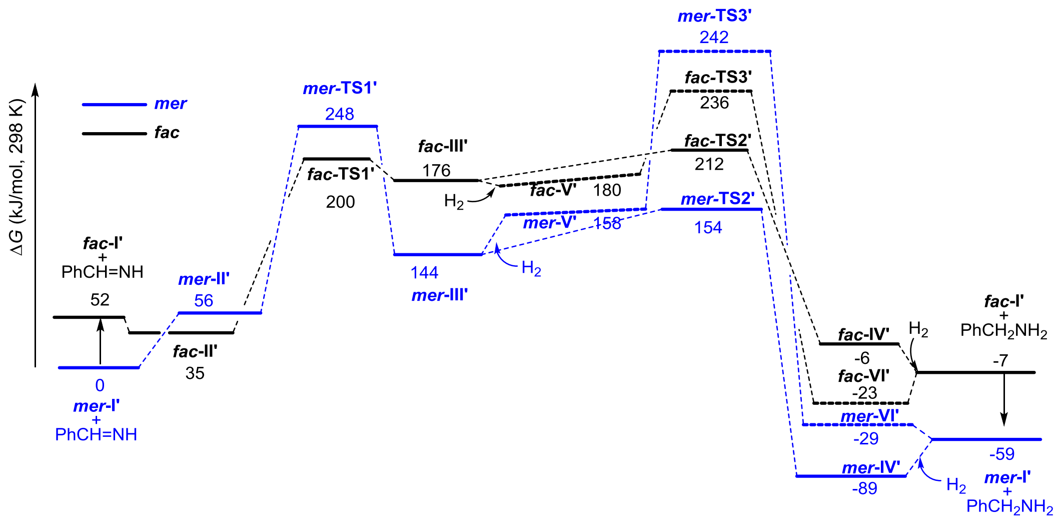

Supplementary Figure 11. Gibbs free energy surface for Ph−CH=NH hydrogenation by using $[L7Co^{II}{-}H_2]^{2+}$ as catalyst through *fac-* and *mer*-route in TFE at B3PW91-SMD/Def2-TZVP//B3PW91/TZVP level.

Di-cationic $[L7Co^{II}\text{-}H_2]^{2+}$ catalyzed cycle in TFE with GD3BJ dispersion (Supplementary Figure 12): Nevertheless, we included dispersion correction (GD3BJ) in the di-cationic cycle (Supplementary Figure 12). Although both potential energy surfaces are similar, but they differ quantitatively, especially in the apparent barriers, i.e.; dispersion correction lowers the barrier by 90 kJ/mol for ***fac***-route. However, these barriers are still much higher than that of mon-ocationic route by 93 kJ/mol for ***fac***-route.

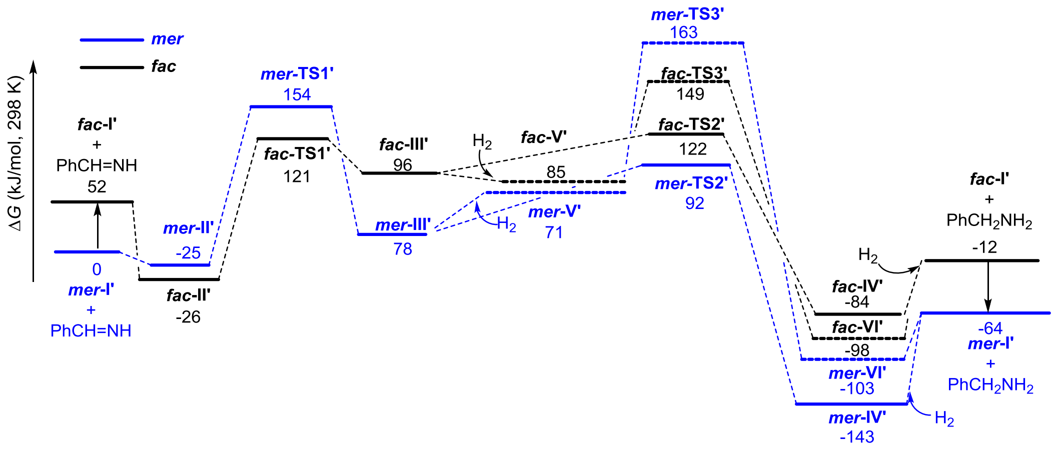

Supplementary Figure 12. Gibbs free energy surface for Ph−CH=NH hydrogenation by using $[L7Co(H_2)]^{2+}$ as catalyst through *fac*- and *mer*-route in TFE with GD3BJ dispersion at B3PW91-SMD-D3/Def2-TZVP//B3PW91/TZVP level.

All these show that the di-cationic route is unlikely and can be discarded. On the basis of the applied reaction conditions (100-120°C, 40 bar H_2 and 15-24 h reaction time), the result with GD3BJ correction is extremely underestimated, and that without dispersion correction is very reasonably.

Supplementary Table 6. Computed total energies (au), enthalpy and free energy correction (au, 298 K) in gas phase at B3PW91/TZVP level as well as single-point total energies in TFE solution without (B3PW91-SMD/Def2-TZVP//B3PW91/TZVP) and with dispersion correction (B3PW91-SMD-D3/Def2-TZVP//B3PW91/TZVP)

	E (B3PW91)	H (298.15)	G (298.15)	E (B3PW91 -SMD)	E(B3PW91-SMD-D3)
**H_2**	-1.178635 ZEP=0.010064	-1.165267	-1.180065	-1.1760136	-1.1761507
**PhCHNH**	-325.672112 ZPE= 0.122318	-325.542363	-325.580491	-325.6925958	-325.7188601
**$PhCH_2NH_2$**	-326.893012 ZPE= 0.145907	-326.738958	-326.778881	-326.9119766	-326.9404524
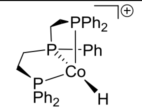 ***fac*-I**	-3722.608459 ZPE= 0.581492	-3721.989757	-3722.101071	-3722.764375	-3722.990356
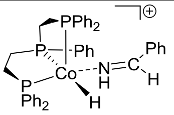 ***fac*-II**	-4048.319178 ZPE= 0.706932	-4047.56758	-4047.695044	-4048.478273	-4048.760476
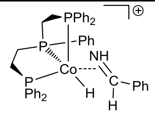 ***fac*-IIπ**	-4048.302943 ZPE= 0.706337	-4047.551964	-4047.678325	-4048.46373	-4048.747175
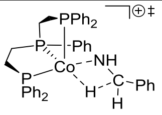 ***fac*-TS1**	-4048.302818 NImag = 1 (-269.2732) ZPE= 0.705561	-4047.553118	-4047.678577	-4048.46391	-4048.747293
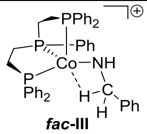 ***fac*-III**	-4048.310216 ZPE= 0.708453	-4047.557395	-4047.684535	-4048.473294	-4048.753119
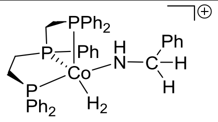 ***fac*-IV**	-4049.493227 ZPE= 0.726734	-4048.721026	-4048.849953	-4049.657233	-4049.937379
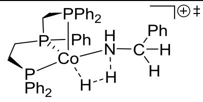 ***fac*-TS2**	-4049.476194 NImag = 1 (-1074.276) ZPE= 0.724539	-4048.706423	-4048.83689	-4049.639005	-4049.918162
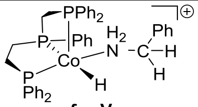 ***fac*-V**	-4049.539322 ZPE= 0.730649	-4048.763122	-4048.895347	-4049.700409	-4049.983921
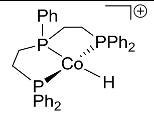 ***mer*-I**	-3722.621223 ZPE= 0.581333	-3722.003702	-3722.112995	-3722.776849	-3722.999503
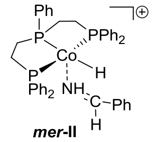 ***mer*-II**	-4048.313283 ZPE= 0.705403	-4047.562847	-4047.693005	-4048.476241	-4048.753658

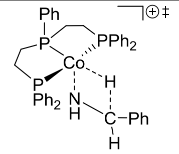 ***mer*-TS1**	-4048.276617 NImag = 1 (-550.8865) ZPE= 0.70575	-4047.527011	-4047.650746	-4048.442854	-4048.72985
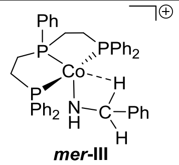 ***mer*-III**	-4048.278815 ZPE= 0.707909	-4047.526748	-4047.652172	-4048.446966	-4048.73243
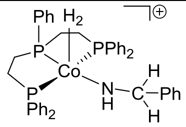 ***mer*-IV**	-4049.495158 ZPE= 0.726195	-4048.723408	-4048.853718	-4049.655023	-4049.939281
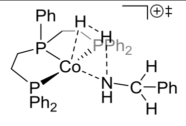 ***mer*-TS2**	-4049.46281 NImag = 1 (-428.622) ZPE= 0.725517	-4048.69229	-4048.821171	-4049.62604	-4049.906983
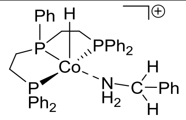 ***mer*-V**	-4049.531299 ZPE= 0.730363	-4048.755594	-4048.885368	-4049.692908	-4049.976893
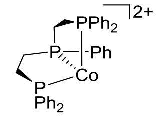 ***fac*-L**	-3721.687621 ZPE= 0.573398	-3721.078652	-3721.185945	-3722.016732	-3722.237035
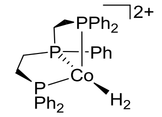 ***fac*-I'**	-3722.893052 ZPE= 0.590774	-3722.26431	-3722.37703	-3723.207984	-3723.438305
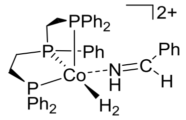 ***fac*-II'**	-4048.622782 ZPE= 0.716541	-4047.860737	-4047.991438	-4048.927496	-4049.207308
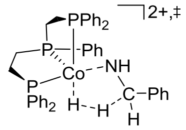 ***fac*-TS1'**	-4048.558113 NImag = 1 (-783.6629) ZPE= 0.712849	-4047.80057	-4047.926839	-4048.864612	-4049.151449
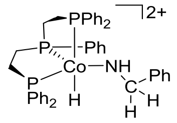 ***fac*-III'**	-4048.571601 ZPE= 0.718514	-4047.80831	-4047.935861	-4048.878149	-4049.165413
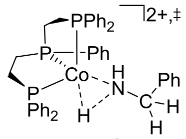 ***fac*-TS2'**	-4048.55908 NImag = 1 (-1039.2811) ZPE= 0.716868	-4047.797497	-4047.926664	-4048.861183	-4049.152144
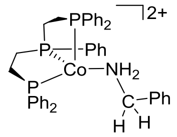 ***fac*-IV'**	-4048.640422 ZPE= 0.723941	-4047.871356	-4048.000142	-4048.952259	-4049.238638

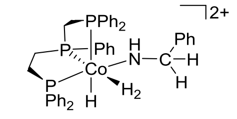 ***fac*-V'**	-4049.765492<br>ZPE= 0.737105	-4048.983446	-4049.10977	-4050.071271	-4050.364214
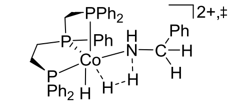 ***fac*-TS3'**	-4049.74176<br>NImag = 1<br>(-1631.6038)<br>ZPE= 0.733016	-4048.96368	-4049.092576	-4050.043258	-4050.333326
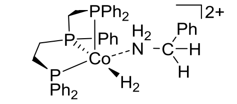 ***fac*-VI'**	-4049.837067<br>ZPE= 0.740871	-4049.050299	-4049.181257	-4050.148618	-4050.434024
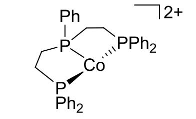 ***mer*-L**	-3721.70554<br>ZPE= 0.574096	-3721.094497	-3721.205948	-3722.034983	-3722.258357
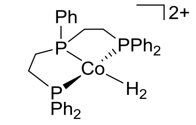 ***mer*-I'**	-3722.912552<br>ZPE= 0.591451	-3722.283629	-3722.39503	-3723.229184	-3723.459504
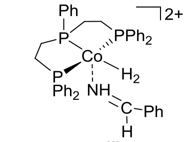 ***mer*-II'**	-4048.613953<br>ZPE= 0.716863	-4047.851941	-4047.979482	-4048.922719	-4049.210212
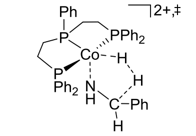 ***mer*-TS1'**	-4048.542301<br>NImag = 1<br>(-936.241)<br>ZPE= 0.713021	-4047.784879	-4047.910597	-4048.846926	-4049.139242
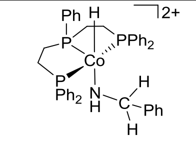 ***mer*-III'**	-4048.581321<br>ZPE= 0.718141	-4047.818248	-4047.946711	-4048.889566	-4049.170999
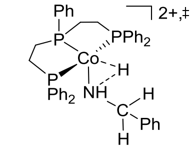 ***mer*-TS2'**	-4048.576581<br>NImag = 1<br>(-743.6983)<br>ZPE= 0.716974	-4047.814912	-4047.942871	-4048.88478	-4049.164912
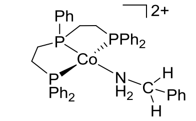 ***mer*-IV'**	-4048.667635<br>ZPE= 0.724234	-4047.898377	-4048.028576	-4048.982473	-4049.259786
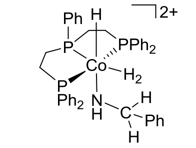 ***mer*-V'**	-4049.767609<br>ZPE= 0.73514	-4048.986855	-4049.117084	-4050.074248	-4050.364338
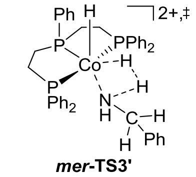 ***mer*-TS3'**	-4049.736521<br>NImag = 1<br>(-1680.3716)<br>ZPE= 0.733173	-4048.958439	-4049.085808	-4050.042746	-4050.329564

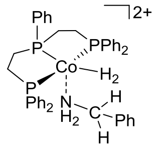 ***mer*-VI'**	-4049.84419<br>ZPE= 0.74083	-4049.057604	-4049.186566	-4050.152587	-4050.437748

NMR Data

p-tolylmethanamine hydrochloride

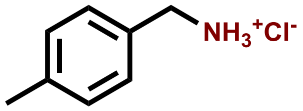

^{1}H NMR (400 MHz, DMSO-*d*$_6$) δ 8.61 (br s, 3H), 7.40 (d, *J* = 8.2 Hz, 2H), 7.20 (d, *J* = 7.6 Hz, 2H), 3.94 (s, 2H), 2.30 (s, 3H). ^{13}C NMR (101 MHz, DMSO-*d*$_6$) δ 138.11 , 131.54 , 129.47 , 129.43 , 42.30 , 21.22 . **White solid.**

(4-(tert-butyl)phenyl)methanamine hydrochloride

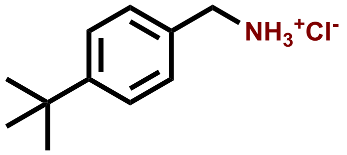

^{1}H NMR (300 MHz, DMSO-*d*$_6$) δ 8.60 (br s, 3H), 7.52 – 7.30 (m, 4H), 3.95 (s, 2H), 1.27 (s, 9H). ^{13}C NMR (75 MHz, DMSO-*d*$_6$) δ 151.32 , 131.63 , 129.25 , 125.72 , 42.22 , 34.79 , 31.54 . **White solid.**

(4-methoxyphenyl)methanamine hydrochloride

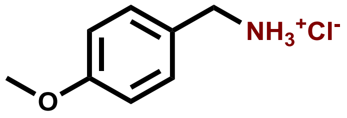

^{1}H NMR (400 MHz, DMSO-*d*$_6$) δ 8.56 (br s, 3H), 7.45 (d, *J* = 8.8 Hz, 2H), 6.95 (d, *J* = 8.7 Hz, 2H), 3.92 (s, 2H), 3.75 (s, 3H). ^{13}C NMR (101 MHz, DMSO-d6) δ 159.74 , 131.03 , 126.47 , 114.31 , 55.65 , 42.04 . **White solid.**

(3,4,5-trimethoxyphenyl)methanamine hydrochloride

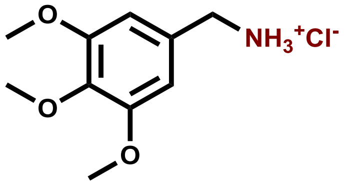

^{1}H NMR (400 MHz, DMSO-*d*$_6$) δ 8.68 (br s, 3H), 6.97 (s, 2H), 3.93 (s, 2H), 3.78 (s, 6H), 3.65 (s, 3H). ^{13}C NMR (101 MHz, DMSO-*d*$_6$) δ 153.22 , 137.70 , 130.08 , 107.04 , 60.46 , 56.48 , 42.83 . **Off white solid.**

(4-fluorophenyl)methanamine hydrochloride

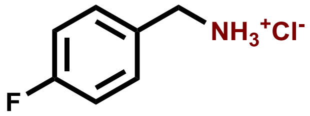

^{1}H NMR (400 MHz, DMSO-*d*$_6$) δ 8.68 (br s, 3H), 7.75 – 7.44 (m, 2H), 7.41 – 7.01 (m, 2H), 3.99 (s, 2H). ^{13}C NMR (101 MHz, DMSO-*d*$_6$) δ 162.49 (d, *J* = 244.5 Hz), 131.87 (d, *J* = 8.4 Hz), 130.88 (d,

J = 3.1 Hz), 115.75 (d, J = 21.5 Hz), 41.78 . **^{19}F NMR (282 MHz, DMSO-d_6)** δ -113.77 . **White solid.**

(4-(trifluoromethyl)phenyl)methanamine hydrochloride

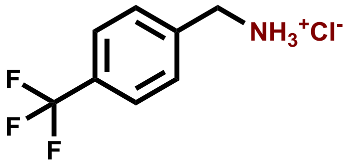

^{1}H NMR (400 MHz, DMSO-d_6) δ 8.81 (br s, 3H), 7.96 – 7.56 (m, 4H), 4.13 (s, 2H).**^{13}C NMR (101 MHz, DMSO-d_6)** δ 139.30 , 130.27 , 129.26 (q, J = 31.9 Hz), 125.74 (q, J = 3.7 Hz),124.58 (q, J = 272.2 Hz), 41.99 . **^{19}F NMR (282 MHz, DMSO-d_6)** δ -61.14 .**White solid.**

(4-chlorophenyl)methanamine hydrochloride

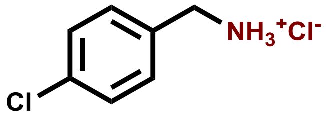

^{1}H NMR (400 MHz, DMSO-d_6) δ 8.72 (br s, 3H), 7.60 – 7.53 (m, 2H), 7.51 – 7.45 (m, 2H), 4.01 (s, 2H). **^{13}C NMR (101 MHz, DMSO-d_6)** δ 133.59 , 133.52 , 131.50 , 128.90 , 41.81 . **White solid.**

(4-(trifluoromethoxy)phenyl)methanamine hydrochloride

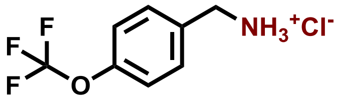

^{1}H NMR (300 MHz, DMSO-d_6) δ 8.70 (br s, 3H), 7.69 (d, J = 8.7 Hz, 2H), 7.41 (d, J = 7.8 Hz, 2H), 4.05 (s, 2H). **^{13}C NMR (75 MHz, DMSO-d_6)** δ 148.70 , 134.07 , 131.67 , 121.52 , 120.48 (q, J = 256.3 Hz), 41.76 . **^{19}F NMR (282 MHz, DMSO-d_6)** δ -56.91 . **Off white solid.**

naphthalen-1-ylmethanamine hydrochloride

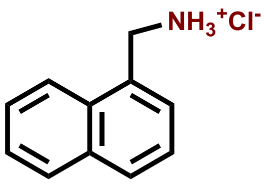

^{1}H NMR (400 MHz, DMSO-d_6) δ 8.79 (br s, 3H), 8.26 – 8.09 (m, 1H), 8.07 – 7.90 (m, 2H), 7.77 – 7.48 (m, 4H), 4.51 (s, 2H). **^{13}C NMR (101 MHz, DMSO-d_6)** δ 133.65 , 131.12 , 130.46 , 129.43 , 129.08 , 127.72 , 127.19 , 126.66 , 125.81 , 123.95 , 40.61 . **Pale brown solid.**

(3-hydroxy-4-methoxyphenyl)methanamine hydrochloride

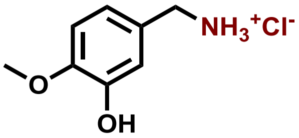

^{1}H NMR (400 MHz, DMSO-d_6) δ 8.49 (br s, 3H), 7.21 – 6.63 (m, 3H), 3.90 (s, 2H), 3.81 (s, 3H). **^{13}C NMR (101 MHz, DMSO-d_6)** δ 148.27 , 146.87 , 126.82 , 120.45 , 116.84 , 112.51 , 56.15 , 42.32 . **Brown solid.**

(4-(methylthio)phenyl)methanamine hydrochloride

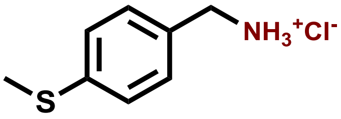

^{1}H NMR (400 MHz, DMSO-d_6) δ 8.03 (br s, 3H), 7.46 (d, J = 8.3 Hz, 2H), 7.27 (d, J = 8.3 Hz, 2H), 3.95 (s, 2H), 2.46 (s, 3H). **^{13}C NMR (101 MHz, DMSO-d_6)** δ 138.95 , 130.92 , 130.17 , 126.21 , 42.11 , 15.13 . **Brown solid.**

(E)-(4-styrylphenyl)methanamine hydrochloride

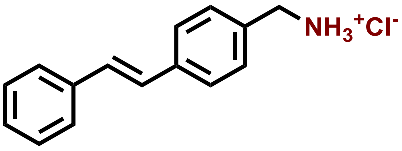

^{1}H NMR (400 MHz, DMSO-d_6) δ 8.62 (br s, 3H), 7.72 – 7.57 (m, 4H), 7.53 (d, J = 7.9 Hz, 2H), 7.45 – 7.32 (m, 3H), 7.33 – 7.20 (m, 2H), 4.00 (s, 2H). **^{13}C NMR (101 MHz, DMSO-d_6)** δ 137.59, 137.31, 133.76 , 129.86 , 129.45 , 129.40 , 129.20 , 128.26 , 128.22 , 127.00 , 42.37 . **Yellow solid.**

(4-(benzyloxy)phenyl)methanamine hydrochloride

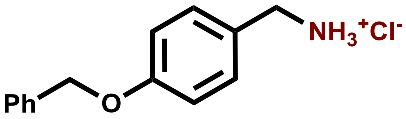

^{1}H NMR (300 MHz, DMSO-d_6) δ 8.58 (br s, 3H), 7.48 – 7.27 (m, 7H), 7.02 (d, J = 8.7 Hz, 2H), 5.12 (s, 2H), 3.91 (s, 2H). **^{13}C NMR (75 MHz, DMSO-d_6)** δ 158.74 , 137.41 , 131.06 , 128.90 , 128.29 , 128.08 , 126.70 , 115.23 , 69.61 , 42.03 . **Off white solid.**

(4-(4,4,5,5-tetramethyl-1,3,2-dioxaborolan-2-yl)phenyl)methanamine hydrochloride

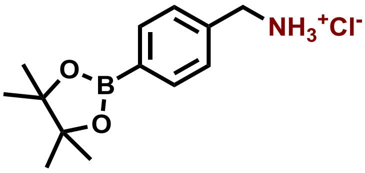

^{1}H NMR (400 MHz, DMSO-d_6) δ 8.61 (br s, 3H), 7.69 (d, J = 7.9 Hz, 2H), 7.52 (d, J = 8.0 Hz, 2H), 4.02 (s, 2H), 1.30 (s, 12H). **^{13}C NMR (101 MHz, DMSO-d_6)** δ 137.85 , 134.96 , 129.42 , 128.77 , 84.22 , 42.50 , 25.13 . **White solid**.

benzo[d][1,3]dioxol-5-ylmethanamine hydrochloride

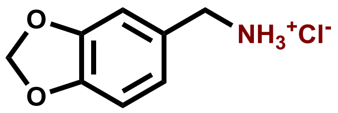

^{1}H NMR (400 MHz, DMSO-d_6) δ 8.57 (s, 3H), 7.16 (dd, J = 1.7, 0.5 Hz, 1H), 6.98 (dd, J = 8.0, 1.7 Hz, 1H), 6.93 (dd, J = 8.0, 0.4 Hz, 1H), 6.03 (s, 2H), 3.90 (s, 2H).
^{13}C NMR (101 MHz, DMSO-d_6) δ 147.69 , 147.67 , 128.17 , 123.35 , 109.99 , 108.65 , 101.63 , 42.37 . **Off white solid.**

(2,3-dihydrobenzo[b][1,4]dioxin-6-yl)methanamine hydrochloride

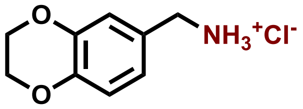

^{1}H NMR (400 MHz, DMSO-d_6) δ 8.29 (br s, 3H), 7.07 (d, J = 2.1 Hz, 1H), 6.96 (dd, J = 8.3, 2.1 Hz, 1H), 6.85 (d, J = 8.2 Hz, 1H), 4.23 (s, 4H), 3.86 (s, 2H). ^{13}C NMR (101 MHz, DMSO-d_6) δ 143.91 , 143.57 , 127.45 , 122.54 , 118.40 , 117.45 , 64.56 , 64.52 , 42.05 . **Off white solid.**

(benzo[d][1,3]dioxol-5-yl)-2-methylpropan-1-amine hydrochloride

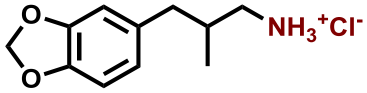

^{1}H NMR (400 MHz, DMSO-d_6) δ 8.30 (br s, 3H), 6.88 – 6.72 (m, 2H), 6.63 (dd, J = 8.0, 1.6 Hz, 1H), 5.95 (s, 2H), 2.77 – 2.54 (m, 3H), 2.28 (dd, J = 13.5, 8.3 Hz, 1H), 2.08 – 1.96 (m, 1H), 0.85 (d, J = 6.6 Hz, 3H). ^{13}C NMR (101 MHz, DMSO-d_6) δ 147.58 , 145.86 , 133.85 , 122.38 , 109.74 , 108.43 , 101.13 , 44.25 , 39.72 , 33.72 , 17.31 . **Pale brown solid.**

(4-((2-chloro-6-fluorobenzyl)oxy)-3-methoxyphenyl)methanamine hydrochloride

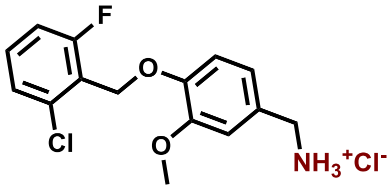

^{1}H NMR (400 MHz, DMSO-d_6) δ 8.55 (br s, 3H), 7.56 – 7.46 (m, 1H), 7.42 (dd, J = 8.1, 1.2 Hz, 1H), 7.35 – 7.27 (m, 2H), 7.13 (d, J = 8.2 Hz, 1H), 7.03 (dd, J = 8.2, 2.0 Hz, 1H), 5.13 (s, 2H), 3.95 (s, 2H), 3.75 (s, 3H). ^{13}C NMR (101 MHz, DMSO-d_6) δ 161.94 (d, J = 250.0 Hz), 149.53 , 148.10 , 136.00 (d, J = 5.2 Hz), 132.29 (d, J = 9.9 Hz), 127.92 , 126.18 (d, J = 3.2 Hz), 122.49 (d, J = 17.9 Hz), 121.85 , 115.23 (d, J = 22.4 Hz), 114.14, 113.97, 62.16 , 56.08 , 42.47 . ^{19}F NMR (282 MHz, DMSO-d_6) δ -113.48 . **Off white solid.**

[1,1'-biphenyl]-4-ylmethanamine hydrochloride

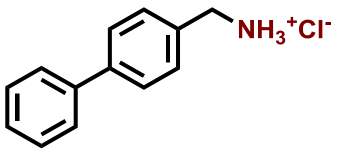

^{1}H NMR (400 MHz, DMSO-*d_6*) δ 8.75 (br s, 3H), 7.74 – 7.61 (m, 6H), 7.50 – 7.43 (m, 2H), 7.41 – 7.33 (m, 1H), 4.06 (s, 2H). **^{13}C NMR (101 MHz, DMSO-*d_6*)** δ 140.59 , 139.99 , 133.74 , 130.12 , 129.45 , 128.12 , 127.19 , 127.14 , 42.26 . **Off white solid.**

3-(4-(tert-butyl)phenyl)-2-methylpropan-1-amine hydrochloride

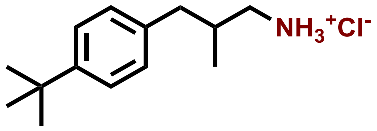

^{1}H NMR (300 MHz, DMSO-*d_6*) δ 8.36 (br s, 3H), 7.34 (d, *J* = 8.2 Hz, 2H), 7.17 (d, *J* = 8.2 Hz, 2H), 2.85 – 2.60 (m, 3H), 2.39 (dd, *J* = 13.4, 8.3 Hz, 1H), 2.20 – 2.03 (m, 1H), 1.29 (s, 9H), 0.92 (d, *J* = 6.6 Hz, 3H). **^{13}C NMR (75 MHz, DMSO-*d_6*)** δ 148.65 , 136.95 , 129.16 , 125.39 , 49.06 , 44.38 , 34.50 , 33.54 , 31.65 , 17.46 . **Pale brown solid.**

2-phenylpropan-1-amine hydrochloride

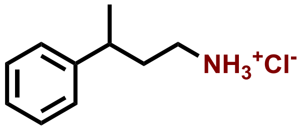

^{1}H NMR (300 MHz, DMSO-*d_6*) δ 8.17 (br s, 3H), 7.47 – 7.03 (m, 5H), 3.71 – 3.62 (m, 1H) 2.88 – 2.74 (m, 1H), 2.74 – 2.62 (m, 1H), 1.86 (q, *J* = 7.7 Hz, 2H), 1.18 (d, *J* = 6.9 Hz, 3H). **^{13}C NMR (75 MHz, DMSO-*d_6*)** δ 146.28 , 128.93 , 127.24 , 126.69 , 37.79 , 36.84 , 35.44 , 22.49 . **Off white solid.**

1-(3-methoxyphenyl)ethan-1- amine hydrochloride

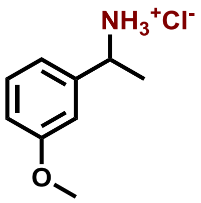

^{1}H NMR (300 MHz, DMSO-*d_6*) δ 8.35 (br s, 3H), 7.37 – 7.25 (m, 1H), 7.23 (dd, *J* = 2.7, 1.6 Hz, 1H), 7.14 – 7.04 (m, 1H), 6.91 (ddd, *J* = 8.3, 2.6, 0.9 Hz, 1H), 4.33 (q, *J* = 6.8 Hz, 1H), 3.77 (s, 3H), 1.52 (d, *J* = 6.7 Hz, 3H). **^{13}C NMR (75 MHz, DMSO-*d_6*)** δ 159.85 , 141.55 , 130.18 , 119.34 , 114.19 , 113.05 , 55.70 , 50.48 , 21.37 . **White solid.**

1-(4-fluorophenyl)ethan-1- amine hydrochloride

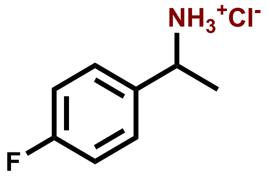

^{1}**H NMR (400 MHz, DMSO-d_6)** δ 8.75 (br s, 3H), 7.77 – 7.47 (m, 2H), 7.37 – 7.01 (m, 2H), 4.46 – 4.35 (m, 1H), 1.52 (d, J = 6.8 Hz, 3H). ^{13}**C NMR (101 MHz, DMSO-d_6)** δ 162.34 (d, J = 244.4 Hz), 136.19 (d, J = 3.0 Hz), 129.71 (d, J = 8.3 Hz), 115.85 (d, J = 21.4 Hz), 49.80 , 21.25 . ^{19}**F NMR (282 MHz, DMSO-d_6)** δ -113.92 . **Brown solid.**

1-(benzo[d][1,3]dioxol-5-yl)ethan-1-amine hydrochloride

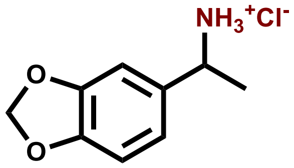

^{1}**H NMR (300 MHz, DMSO-d_6)** δ 8.66 (br s, 3H), 7.21 (d, J = 1.7 Hz, 1H), 7.00 (dd, J = 8.1, 1.7 Hz, 1H), 6.91 (d, J = 8.0 Hz, 1H), 6.02 (s, 2H), 4.50 – 4.09 (m, 1H), 1.49 (d, J = 6.7 Hz, 3H). ^{13}**C NMR (75 MHz, DMSO-d_6)** δ 147.82 , 147.53 , 133.69 , 121.11 , 108.65 , 107.85 , 101.64 , 50.35 , 21.29 . **Off white solid.**

1-(4-(methylthio)phenyl)ethan-1-amine hydrochloride

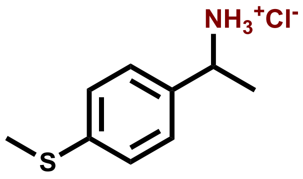

^{1}**H NMR (400 MHz, DMSO-d_6)** δ 8.61 (br s, 3H), 7.49 (d, J = 8.0 Hz, 2H), 7.29 (d, J = 8.0 Hz, 2H), 4.53 – 4.20 (m, 1H), 2.47 (s, 3H), 1.51 (d, J = 6.5 Hz, 3H). ^{13}**C NMR (101 MHz, DMSO-d_6)** δ 138.87 , 136.37 , 128.06 , 126.44 , 50.09 , 21.25 , 15.28 . **Brown solid.**

1-(4-(methylsulfonyl)phenyl)ethan-1-amine hydrochloride

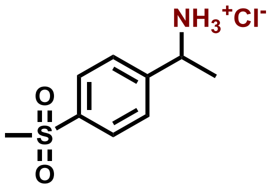

^{1}**H NMR (300 MHz, DMSO-d_6)** δ 8.87 (br s, 3H), 7.98 (d, J = 8.5 Hz, 2H), 7.84 (d, J = 8.4 Hz, 2H), 4.67 – 4.28 (m, 1H), 3.24 (s, 3H), 1.55 (d, J = 6.8 Hz, 3H). ^{13}**C NMR (75 MHz, DMSO-d_6)** δ 145.49 , 141.07 , 128.45 , 127.74 , 50.04 , 43.86 , 21.12 . **Pale brown solid.**

4-(3-aminobutyl)phenol

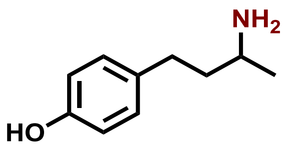

^{1}**H NMR (400 MHz, DMSO-d_6)** δ 6.98 (d, J = 8.4 Hz, 2H), 6.68 (d, J = 8.4 Hz, 2H), 6.56 – 6.30 (m, 3H), 2.97 – 2.82 (m, 1H), 2.52 – 2.49 (m, 2H), 1.81 – 1.40 (m, 2H), 1.10 (d, J = 6.4 Hz, 3H). ^{13}**C NMR (101 MHz, DMSO-d_6)** δ 155.87 , 132.04 , 129.44 , 115.57 , 46.57 , 39.51 , 31.07 , 21.40 . **Brown solid.**

4-(4-hydroxy-3-methoxyphenyl)butan-2-amine hydrochloride

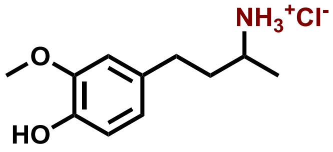

^{1}**H NMR (300 MHz, DMSO-d_6)** δ 8.27 (br s, 3H), 6.84 (d, J = 1.9 Hz, 1H), 6.78 (d, J = 8.0 Hz, 1H), 6.64 (dd, J = 8.0, 1.9 Hz, 1H), 3.79 (s, 3H), 3.26 – 2.95 (m, 1H), 2.65 – 2.57 (m, 2H), 2.07 – 1.87 (m, 1H), 1.87 – 1.60 (m, 1H), 1.28 (d, J = 6.5 Hz, 3H). ^{13}**C NMR (75 MHz, DMSO-d_6)** δ 147.91 , 145.12 , 132.11 , 120.71 , 115.84 , 112.93 , 56.04 , 46.87 , 36.50 , 30.86 , 18.47 . **Pale yellow solid.**

1-(4-hydroxy-3-methoxyphenyl)propan-2- amine hydrochloride

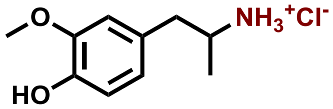

^{1}**H NMR (400 MHz, DMSO-d_6)** δ 8.19 (br s, 3H), 6.80 (d, J = 2.0 Hz, 1H), 6.74 (d, J = 8.0 Hz, 1H), 6.60 (dd, J = 8.1, 2.0 Hz, 1H), 3.75 (s, 3H), 3.40 – 3.25 (m, 1H), 2.92 (dd, J = 13.4, 5.3 Hz, 1H), 2.57 (dd, J = 13.5, 8.8 Hz, 1H), 1.12 (d, J = 6.5 Hz, 3H). ^{13}**C NMR (101 MHz, DMSO-d_6)** δ 147.98 , 145.81 , 127.83 , 121.92 , 115.94 , 113.74 , 56.01 , 48.66 , 31.76 , 18.05 . **Brown solid.**

1-(4-amino-3,5-dichlorophenyl)ethan-1- amine hydrochloride

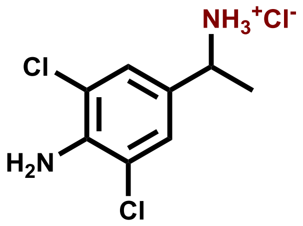

^{1}**H NMR (400 MHz, DMSO-d_6)** δ 8.58 (br s, 3H), 7.46 (s, 2H), 4.45 – 4.09 (m, 1H), 3.83 (br s, 2H), 1.47 (d, J = 6.9 Hz, 3H). ^{13}**C NMR (101 MHz, DMSO-d_6)** δ 141.52 , 128.24 , 127.38 , 118.20 , 49.31 , 20.76 . **Off white solid.**

1-phenylpentan-1- amine hydrochloride

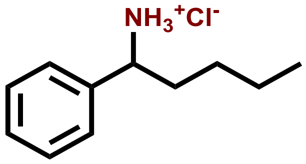

^{1}H NMR (400 MHz, DMSO-d_6) δ 8.57 (br s, 3H), 7.57 – 7.47 (m, 2H), 7.46 – 7.31 (m, 3H), 4.15 (dd, *J* = 9.5, 5.3 Hz, 1H), 2.03 – 1.92 (m, 1H), 1.87 – 1.71 (m, 1H), 1.32 – 1.12 (m, 3H), 1.05 – 0.94 (m, 1H), 0.80 (t, *J* = 7.1 Hz, 3H). **^{13}C NMR (101 MHz, DMSO-d_6)** δ 138.41 , 129.15 , 128.94 , 127.91 , 54.94 , 34.37 , 27.65 , 22.10 , 14.18 . **Pale brown solid.**

1-phenyl-2-(p-tolyl)ethan-1-amine hydrochloride

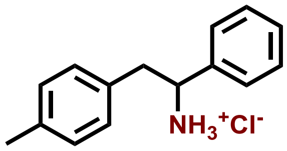

^{1}H NMR (300 MHz, DMSO-d_6) δ 8.89 (br s, 3H), 7.49 – 7.40 (m, 2H), 7.37 – 7.27 (m, 3H), 7.03 – 6.88 (m, 4H), 4.59 – 4.26 (m, 1H), 3.42 (dd, *J* = 13.5, 5.1 Hz, 1H), 3.09 (dd, *J* = 13.4, 10.2 Hz, 1H), 2.18 (s, 3H). **^{13}C NMR (75 MHz, DMSO-d_6)** δ 137.57 , 135.97 , 133.59 , 129.56 , 129.30 , 128.88 , 128.86 , 128.39 , 56.44 , 40.25 , 21.07 . **White solid.**

1,3-diphenylpropan-2- amine hydrochloride

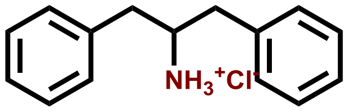

^{1}H NMR (400 MHz, DMSO-d_6) δ 8.38 (br s, 3H), 7.44 – 7.08 (m, 10H), 3.73 – 3.51 (m, 1H), 3.04 (dd, *J* = 13.9, 6.3 Hz, 2H), 2.79 (dd, *J* = 13.9, 6.9 Hz, 2H). **^{13}C NMR (101 MHz, DMSO-d_6)** δ 136.95, 129.83 , 129.06 , 127.25 , 53.63 , 38.14 . **Brown solid.**

(1-hydroxycyclohexyl)(phenyl)methanamine hydrochloride

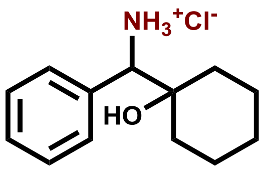

^{1}H NMR (400 MHz, DMSO-d_6) δ 8.52 (br s, 3H), 7.52 – 7.45 (m, 2H), 7.41 – 7.34 (m, 3H), 5.04 (s, 1H), 4.11 (s, 1H), 1.87 – 0.88 (m, 10H). **^{13}C NMR (101 MHz, DMSO-d_6)** δ 135.85 , 129.40 , 128.62 , 128.37 , 71.19 , 63.23 , 34.87 , 33.02 , 25.47 , 21.47 , 21.12 . **Off white solid.**

4-(tert-butyl)cyclohexan-1- amine hydrochloride (diastereomeric mixture)

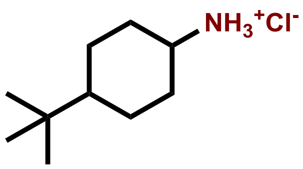

^{1}H NMR (400 MHz, DMSO-d_6) δ 7.73 (br s, 3H), 3.42 – 2.66 (m, 1H), 2.17 – 1.20 (m, 7H), 1.05 – 0.87 (m, 2H), 0.79 (s, 9H). **^{13}C NMR (101 MHz, DMSO-d_6)** δ 49.96 , 47.52 , 46.74 , 46.03 , 32.75 , 32.47 , 30.89 , 28.93 , 27.94 , 27.82 , 25.39 , 20.72 . **Off white solid.**

4-(2,6,6-trimethylcyclohex-1-en-1-yl)butan-2-amine hydrochloride

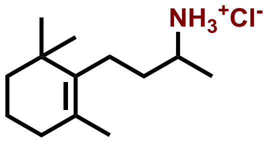

^{1}H NMR (300 MHz, DMSO-d_6) δ 8.11 (br s, 3H), 3.24 – 2.97 (m, 1H), 2.06 – 1.92 (m, 2H), 1.87 (t, *J* = 6.2 Hz, 2H), 1.75 – 1.60 (m, 1H), 1.56 (s, 3H), 1.54 – 1.44 (m, 3H), 1.42 – 1.33 (m, 2H), 1.22 (d, *J* = 6.5 Hz, 3H), 0.97 (s, 3H), 0.97 (s, 3H). **^{13}C NMR (75 MHz, DMSO-d_6)** δ 136.48 , 127.33 , 47.71 , 39.77 , 35.20 , 35.05 , 32.69 , 28.84 , 24.59 , 20.07 , 19.48 , 18.44 . **Brown solid.**

Nonan-5-amine hydrochloride

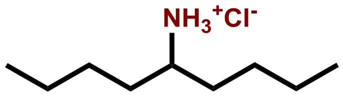

^{1}H NMR (300 MHz, DMSO-d_6) δ 8.10 (br s, 3H), 3.08 – 2.87 (m, 1H), 1.60 – 1.42 (m, 4H), 1.36 – 1.20 (m, 8H), 0.93 – 0.73 (m, 6H). **^{13}C NMR (75 MHz, DMSO-d_6)** δ 51.10 , 31.93 , 27.02 , 22.43 , 14.19 . **White solid.**

6-methylhept-5-en-2-amine hydrochloride

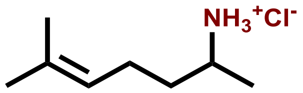

^{1}H NMR (300 MHz, DMSO-d_6) δ 6.03 (br s, 3H), 5.19 – 5.06 (m, 1H), 3.27 – 3.00 (m, 1H), 2.22 – 1.94 (m, 2H), 1.82 – 1.59 (m, 7H), 1.58 – 1.42 (m, 1H), 1.26 (d, *J* = 6.4 Hz, 3H). **^{13}C NMR (75 MHz, DMSO-d_6)** δ 132.10 , 123.65 , 46.94 , 34.88 , 25.88 , 24.09 , 18.60 , 18.04 . **Pale brown gum.**

4-(1H-indol-3-yl)butan-2-amine hydrochloride

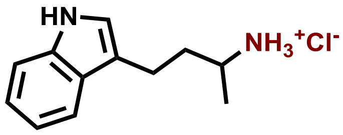

^{1}H NMR (300 MHz, DMSO-d_6) δ 11.00 (s, 1H), 8.31 (br s, 3H), 7.57 (d, *J* = 7.8 Hz, 1H), 7.39 (d, *J* = 8.0 Hz, 1H), 7.22 – 7.15 (m, 1H), 7.08 (ddd, *J* = 8.1, 7.0, 1.2 Hz, 1H), 6.99 (ddd, *J* = 7.9, 6.9, 1.1 Hz, 1H), 3.29 – 3.09 (m, 1H), 2.94 – 2.65 (m, 2H), 2.21 – 1.99 (m, 1H), 1.95 – 1.68 (m, 1H), 1.30

(d, J = 6.4 Hz, 3H). **^{13}C NMR (75 MHz, DMSO-d_6)** δ 136.82 , 127.41 , 122.81 , 121.40 , 118.85 , 118.64 , 113.60 , 111.96 , 47.14 , 35.18 , 21.27 , 18.54 . **Brown solid.**

4-(6-methoxynaphthalen-2-yl)butan-2-amine hydrochloride

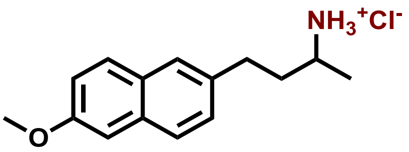

^{1}H NMR (300 MHz, DMSO-d_6) δ 8.36 (br s, 3H), 7.77 (dd, J = 8.8, 2.7 Hz, 2H), 7.70 – 7.60 (m, 1H), 7.37 (dd, J = 8.4, 1.7 Hz, 1H), 7.30 (d, J = 2.5 Hz, 1H), 7.16 (dd, J = 8.9, 2.5 Hz, 1H), 3.87 (s, 3H), 3.35 – 3.12 (m, 1H), 2.96 – 2.70 (m, 2H), 2.19 – 2.01 (m, 1H), 1.95 – 1.71 (m, 1H), 1.32 (d, J = 6.5 Hz, 3H). **^{13}C NMR (75 MHz, DMSO-d_6)** δ 157.27 , 136.55 , 133.26 , 129.25 , 129.03 , 128.02 , 127.31 , 126.41 , 119.00 , 106.25 , 55.60 , 46.98 , 36.21 , 31.28 , 18.51 . **Off white solid.**

6-(3,7-dimethyl-2,6-dioxo-2,3,6,7-tetrahydro-1H-purin-1-yl)hexan-2-amine hydrochloride

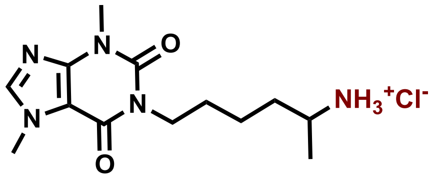

^{1}H NMR (300 MHz, Methanol-d_4) δ 7.91 (s, 1H), 4.83 (br s, 3H), 3.89 (s, 3H), 3.87 – 3.81 (m, 2H), 3.40 (s, 3H), 3.25 – 3.16 (m, 1H), 1.70 – 1.48 (m, 4H), 1.43 – 1.29 (m, 2H), 1.24 (d, J = 6.4 Hz, 3H). **^{13}C NMR (75 MHz, Methanol-d_4)** δ 154.88 , 151.34 , 147.72 , 142.33 , 107.26 , 47.55 , 40.46 , 33.88 , 32.93 , 28.96 , 27.13 , 22.29 , 17.33 . **White solid.**

1-(4-fluorophenyl)-4-(4-(pyridin-2-yl)piperazin-1-yl)butan-1-amine

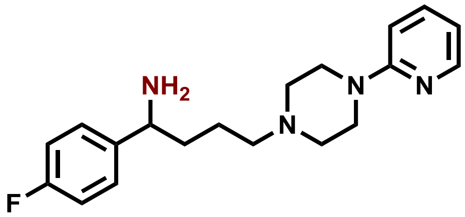

^{1}H NMR (300 MHz, Chloroform-d) δ 8.17 – 8.01 (m, 1H), 7.45 – 7.30 (m, 1H), 7.27 – 7.21 (m, 2H), 6.99 – 6.86 (m, 2H), 6.66 – 6.31 (m, 2H), 3.99 – 3.67 (m, 1H), 3.57 – 3.33 (m, 4H), 3.32 – 3.15 (m, 2H), 2.51 – 2.33 (m, 4H), 2.29 (t, J = 7.3 Hz, 2H), 1.84 – 1.06 (m, 4H). **^{13}C NMR (75 MHz, Chloroform-d)** δ 161.92 (d, J = 245.1 Hz), 159.47 , 147.90 , 140.73 , 137.43 , 128.05 (d, J = 7.9 Hz), 115.32 (d, J = 21.2 Hz), 113.30 , 107.07 , 58.35 , 55.45 , 52.97 , 45.10 , 36.98 , 23.70 . **^{19}F NMR (282 MHz, Chloroform-d)** δ -114.96 . **Brown solid.**

(8R,9S,13S,14S)-3-hydroxy-13-methyl-7,8,9,11,12,13,14,15,16,17-decahydro-6H-cyclopenta[a]phenanthren-17-amine hydrochloride (diastereomeric mixture)

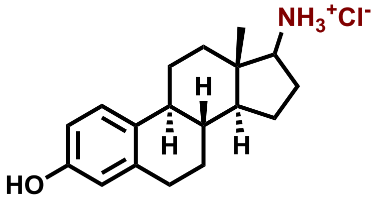

^{1}H NMR (400 MHz, DMSO-*d*$_6$) δ 9.11 (br s, 1H), 8.27 (br s, 3H), 7.03 (d, *J* = 8.7 Hz, 1H), 6.53 (dd, *J* = 8.4, 2.6 Hz, 1H), 6.46 (d, *J* = 2.7 Hz, 1H), 3.22 – 2.91 (m, 1H), 2.86 – 2.61 (m, 2H), 2.30 – 2.00 (m, 4H), 1.85 – 1.51 (m, 4H), 1.35 – 1.20 (m, 5H), 0.75 (s, 3H). **^{13}C NMR (101 MHz, DMSO-*d*$_6$)** δ 155.52 , 155.40 , 137.43 , 137.41 , 130.80 , 130.38 , 126.54 , 126.44 , 115.42 , 115.40, 113.26 , 113.21 , 59.95, 59.24, 51.27 , 49.98 , 47.93 , 43.99 , 43.86 , 43.56 , 43.27 , 39.16 , 38.66 , 36.23 , 29.64 , 29.53 , 28.38 , 28.26 , 27.50 , 27.44 , 26.71 , 26.18 , 26.09 , 23.55 , 18.35 , 12.16 . (traces of ethylacetate solvent peak was observed in the NMR spectra). **HRMS (ESI-TOF, m/z):** Calcd for C18H25NO [M+H]+ 272.2014; found 272.2013. **Off white solid.**

(5S,8R,9R,10S,13S,14S,17S)-17-hydroxy-10,13-dimethylhexadecahydro-1H-cyclopenta[a]phenanthren-3-amine hydrochloride (diastereomeric mixture)

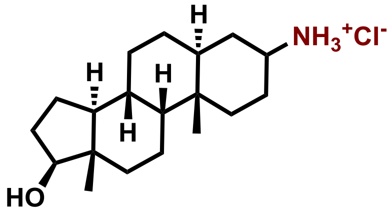

^{1}H NMR (400 MHz, DMSO-*d*$_6$) δ 8.09 (br s, 3H), 4.44 (s, 1H), 3.49 – 3.14 (m, 1H), 1.97 – 0.81 (m, 23H), 0.75 (s, 3H), 0.62 (s, 3H). **^{13}C NMR (101 MHz, DMSO-*d*$_6$)** δ 80.53, 80.48 , 54.08 , 53.70 , 52.94, 52.70, 51.20 , 51.01 , 49.90 , 46.68 , 44.57 , 44.02, 42.18, 43.02 , 38.44 , 37.11 , 37.05 , 36.49 , 36.00 , 35.56 , 35.48 , 32.77 , 31.61 , 31.26 , 30.99 , 30.30 , 28.44 , 28.21 , 26.32 , 26.27 , 24.29 , 23.51 , 20.79 , 20.73 , 20.40 , 12.29 , 11.81 , 11.56 . **HRMS (ESI-TOF, m/z):** Calcd for C19H33NO [M+H]+ 292.2640; found 292.2646. **White solid.**

(5S,8R,9S,10S,13S,14S,17S)-17-hydroxy-10,13,17-trimethylhexadecahydro-1H-cyclopenta[a]phenanthren-3- amine hydrochloride

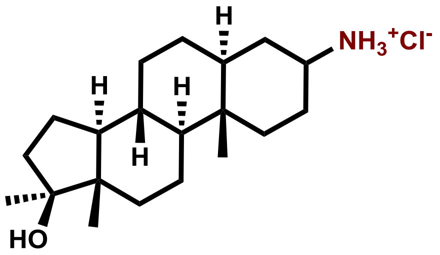

^{1}H NMR (400 MHz, DMSO-*d*$_6$) δ 8.11 (br s, 3H), 3.46 – 3.21 (m, 1H), 1.82 – 1.10 (m, 22H), 1.08 (s, 3H), 0.76 (s, 3H), 0.74 (s, 3H). **^{13}C NMR (101 MHz, DMSO-*d*$_6$)** δ 80.14 , 80.12 , 53.95 , 53.60 , 51.01 , 50.88 , 50.66 , 49.92 , 49.04 , 46.68 , 45.63 , 45.44 , 44.62 , 40.64 , 38.78 , 38.44 , 36.51 , 36.34 , 36.28 , 36.00 , 35.90 , 35.81 , 35.57 , 32.78 , 31.92 , 31.85 , 31.78 , 31.28 , 30.99 , 28.50 , 28.26 , 26.61 , 26.30 , 24.30 , 23.49 , 20.85 , 20.45 , 14.67 , 12.29 , 11.57 . (OH proton was not picked up in^{1}H NMR). **HRMS (ESI-TOF, m/z):** Calcd for C20H35NO [M+H]+ 360.2797; found 306.2792.

White solid.

(1S,5S,8R,9S,10S,13S,14S,17S)-17-hydroxy-1,10,13-trimethylhexadecahydro-1H-cyclopenta[a]phenanthren-3- amine hydrochloride

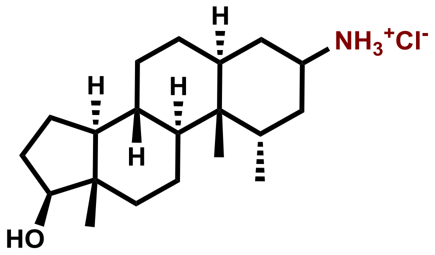

^{1}H NMR (300 MHz, DMSO-*d*$_6$) δ 7.50 (br s, 3H), 3.87 - 3.12 (m, 1H), 3.12 - 2.822 (m, 1H), 09 – 1.02 (m, 18H), 1.02 – 0.73 (m, 9H), 0.60 (s, 3H). **^{13}C NMR (75 MHz, DMSO-*d*$_6$)** δ 80.45 , 80.42 , 51.24 , 51.12 , 48.60 , 46.16 , 46.03 , 43.07 , 37.76 , 37.72 , 37.50 , 37.46 , 37.22 , 36.94 , 36.11 , 35.97 , 35.59 , 34.37 , 33.07 , 32.98 , 31.79 , 31.72 , 31.41 , 31.29 , 31.06 , 30.25 , 28.50 , 28.41 , 26.79 , 23.50 , 20.06 , 19.91 , 15.58 , 14.90 , 14.84 , 14.10 , 14.05 , 13.84 , 12.25 , 11.89 . (OH proton was not picked up in ^{1}H NMR) **HRMS (ESI-TOF, m/z):** Calcd for C20H35NO [M+H]+ 360.2797; found 306.2800. **White solid.**

NMR spectra

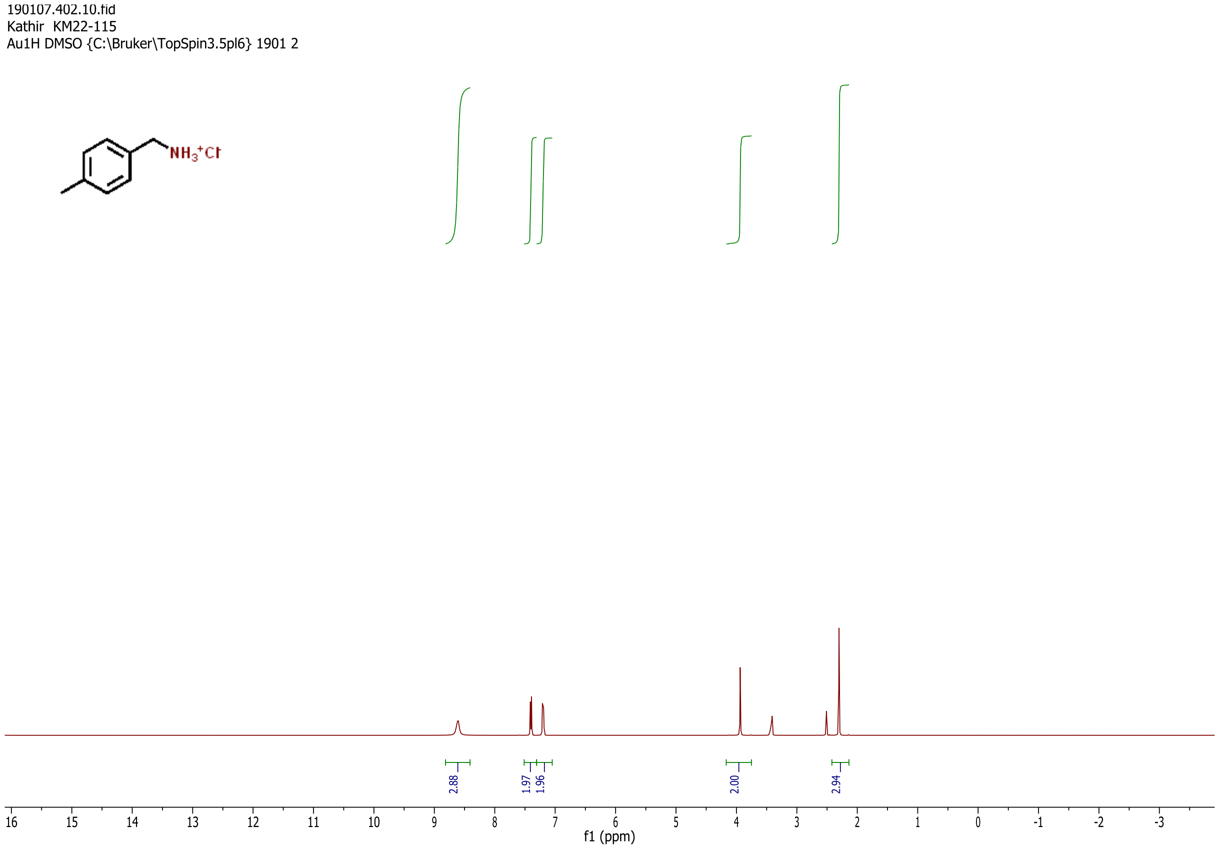

Supplementary Figure 13. ^{1}H NMR (**400 MHz, DMSO-*d*$_6$**)

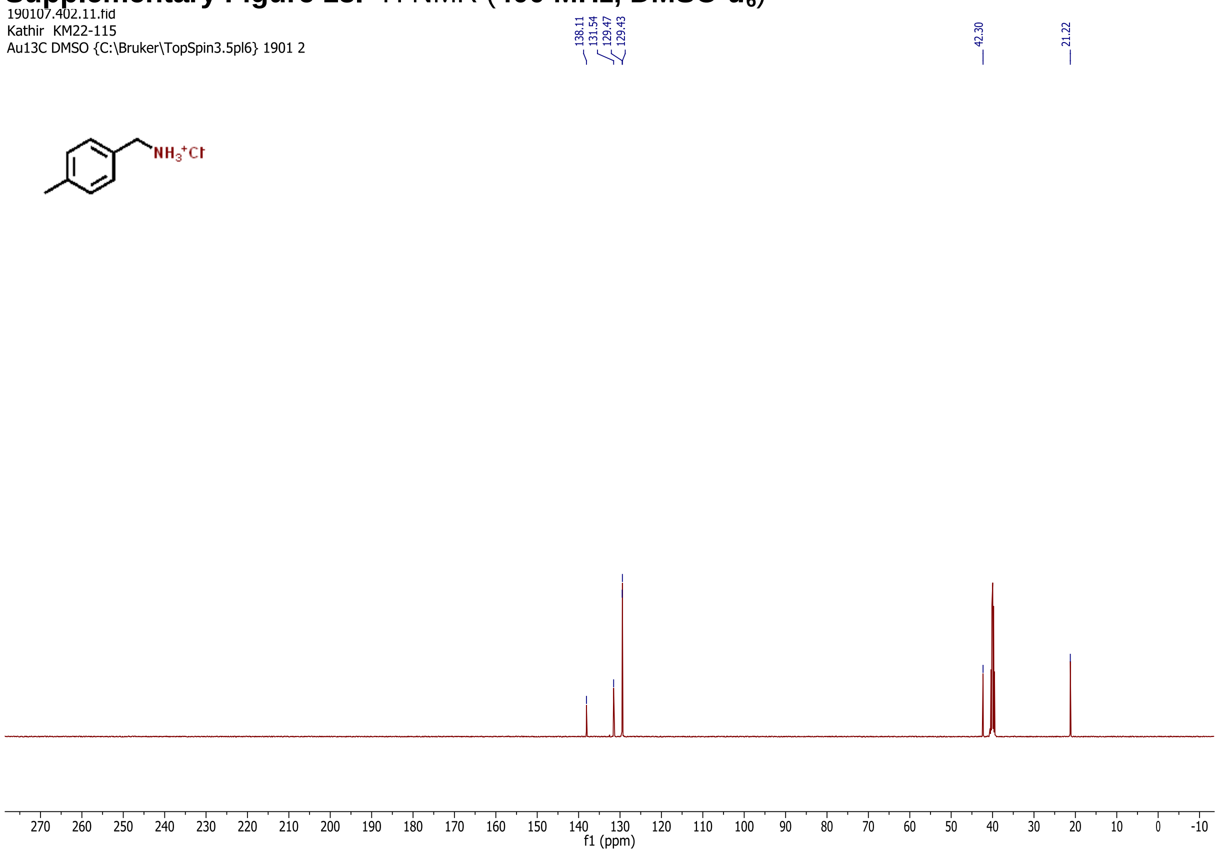

Supplementary Figure 14. ^{13}C NMR (**101 MHz, DMSO-*d*$_6$**)

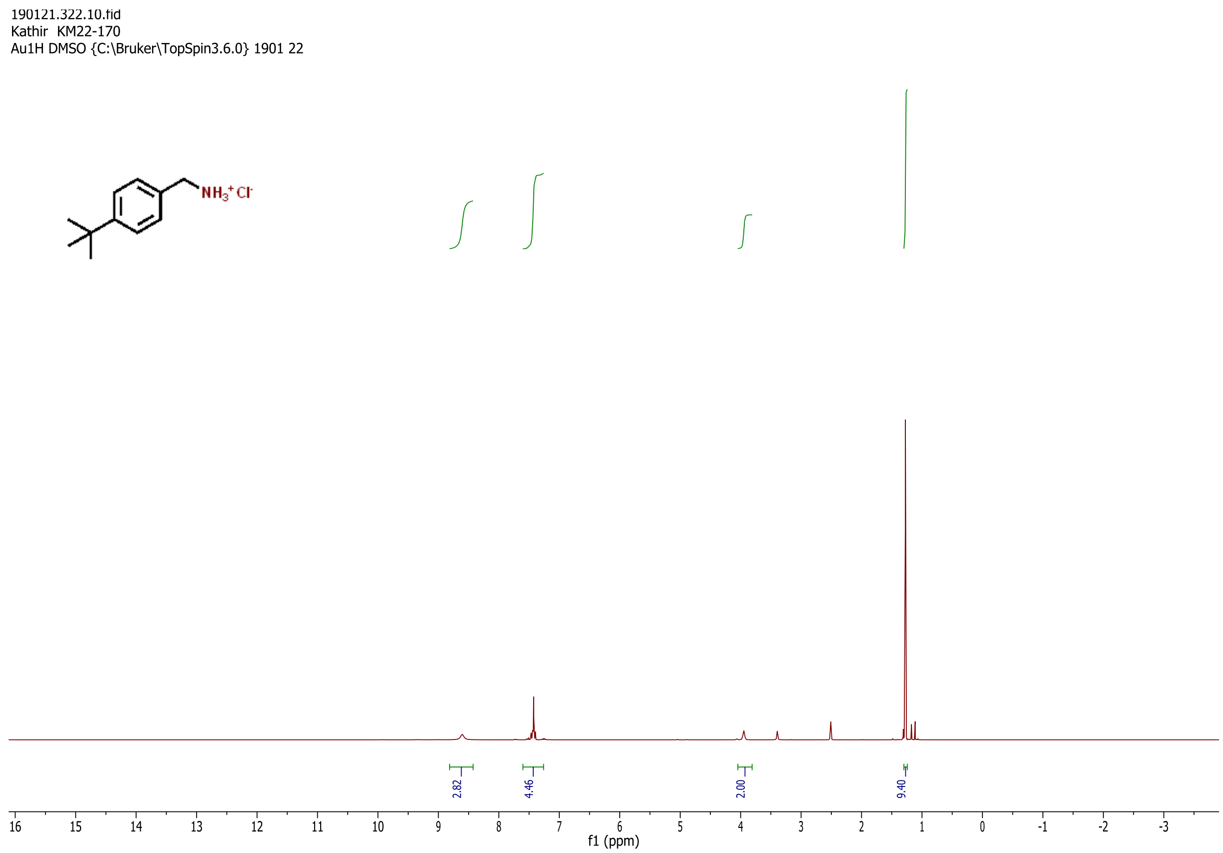

Supplementary Figure 15. ^{1}H NMR (**300 MHz, DMSO-*d*$_6$**)

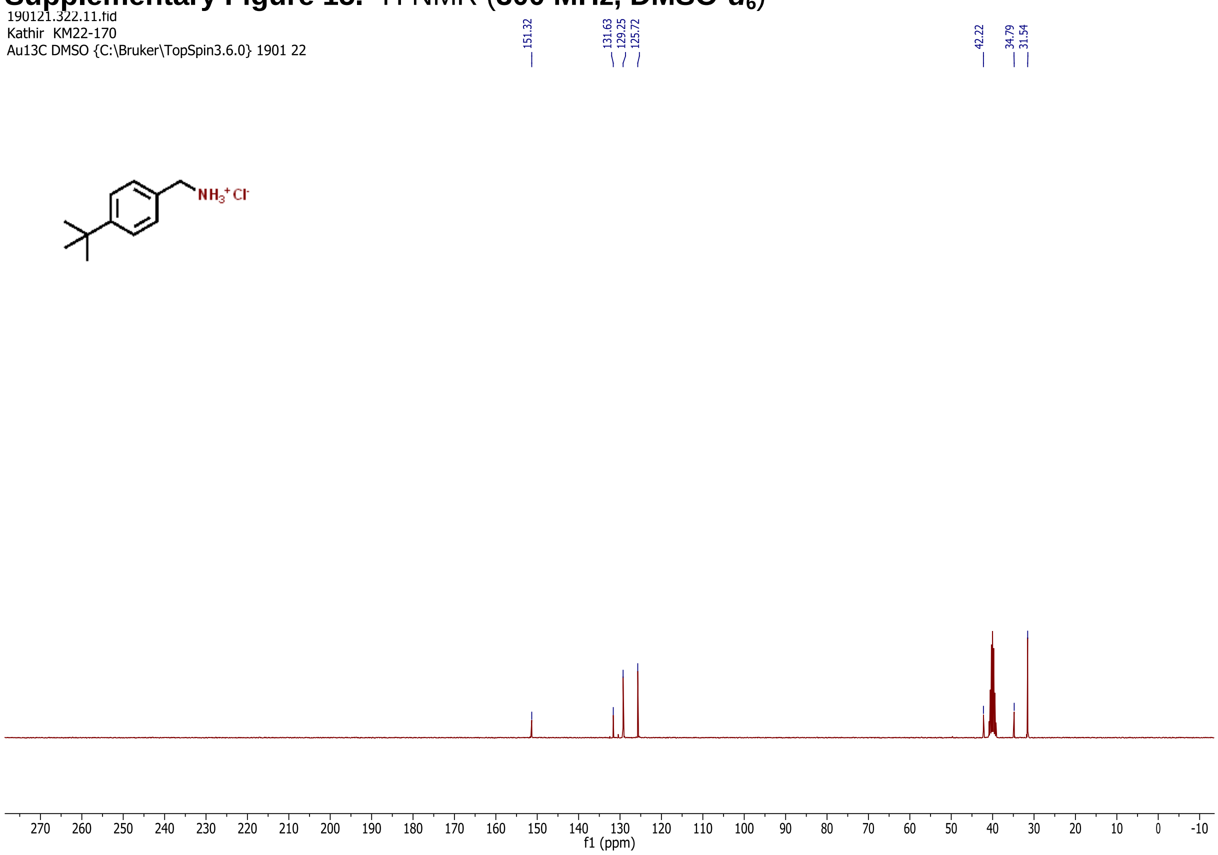

Supplementary Figure 16. ^{13}C NMR (**75 MHz, DMSO-*d*$_6$**)

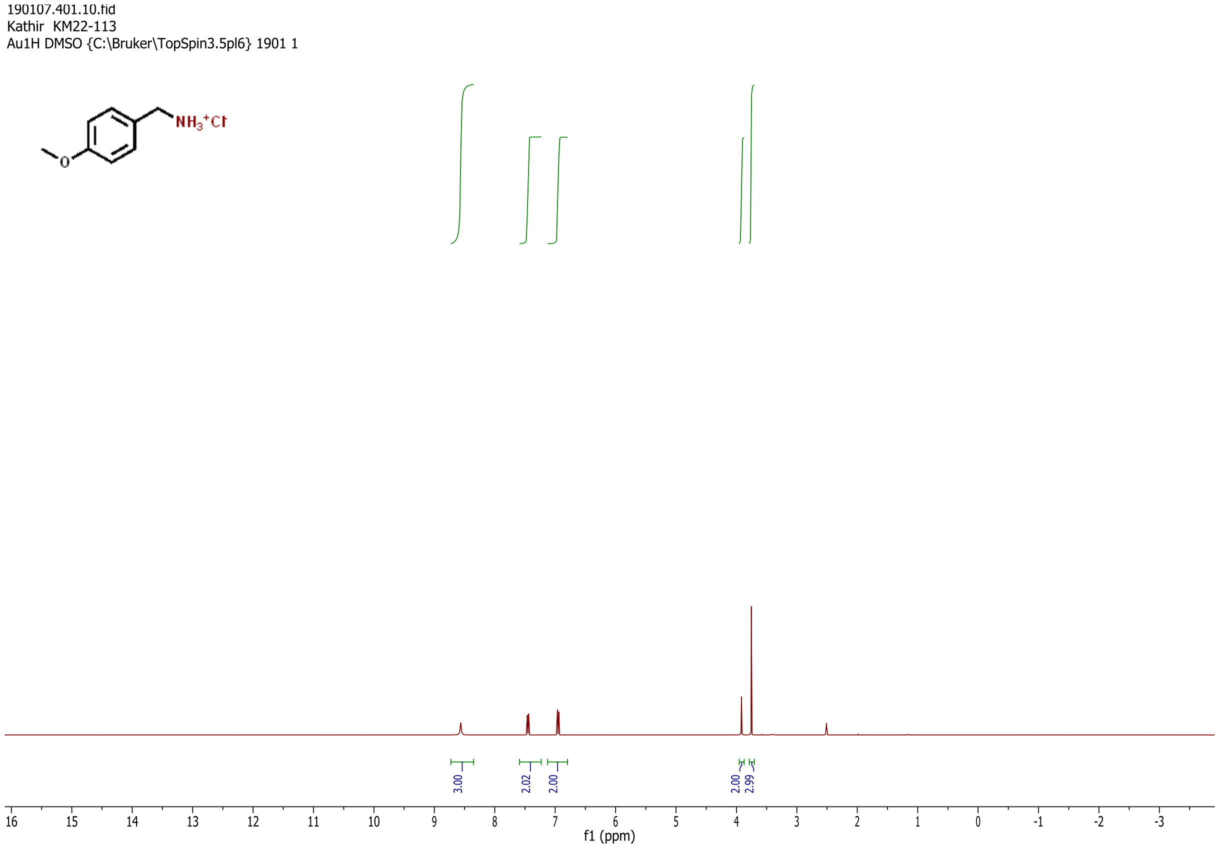

Supplementary Figure 17. ^{1}H NMR (**400 MHz, DMSO-*d*$_6$**)

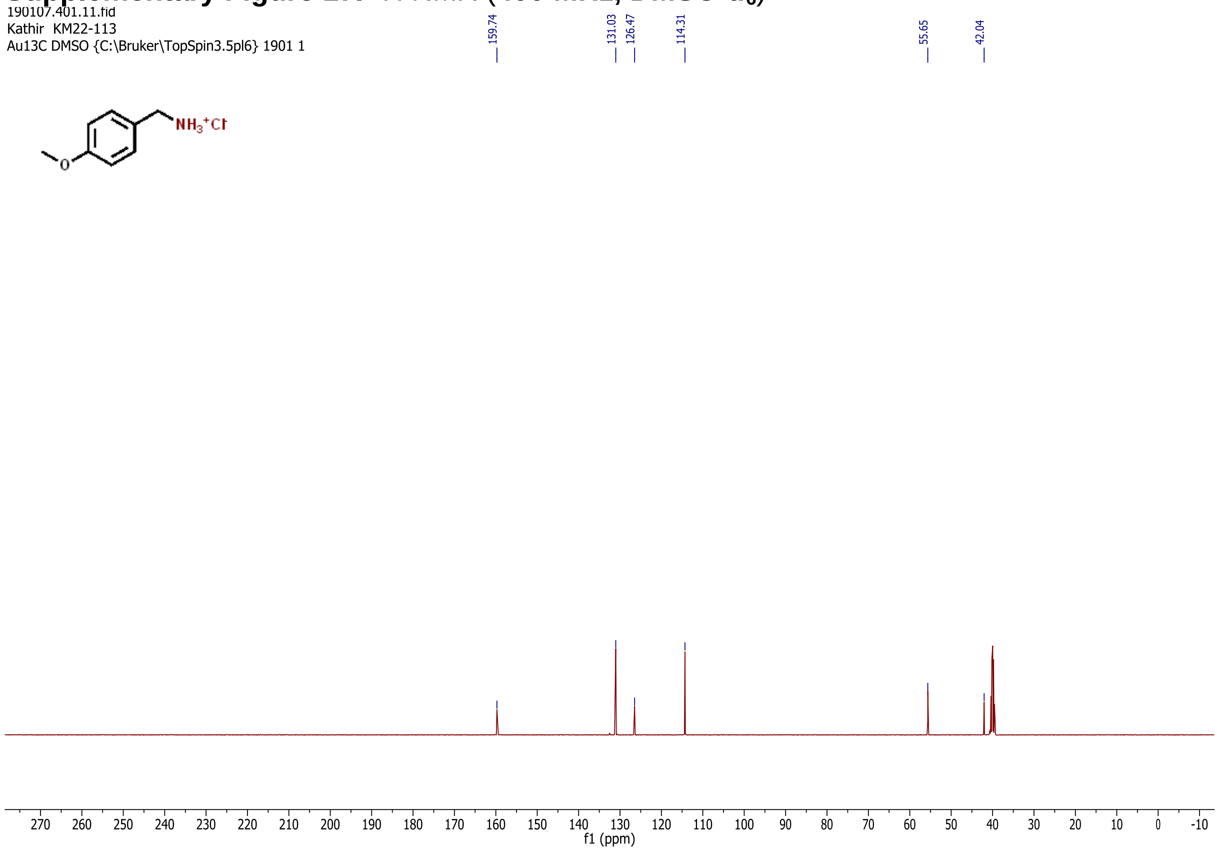

Supplementary Figure 18. ^{13}C NMR (**101 MHz, DMSO-*d*$_6$**)

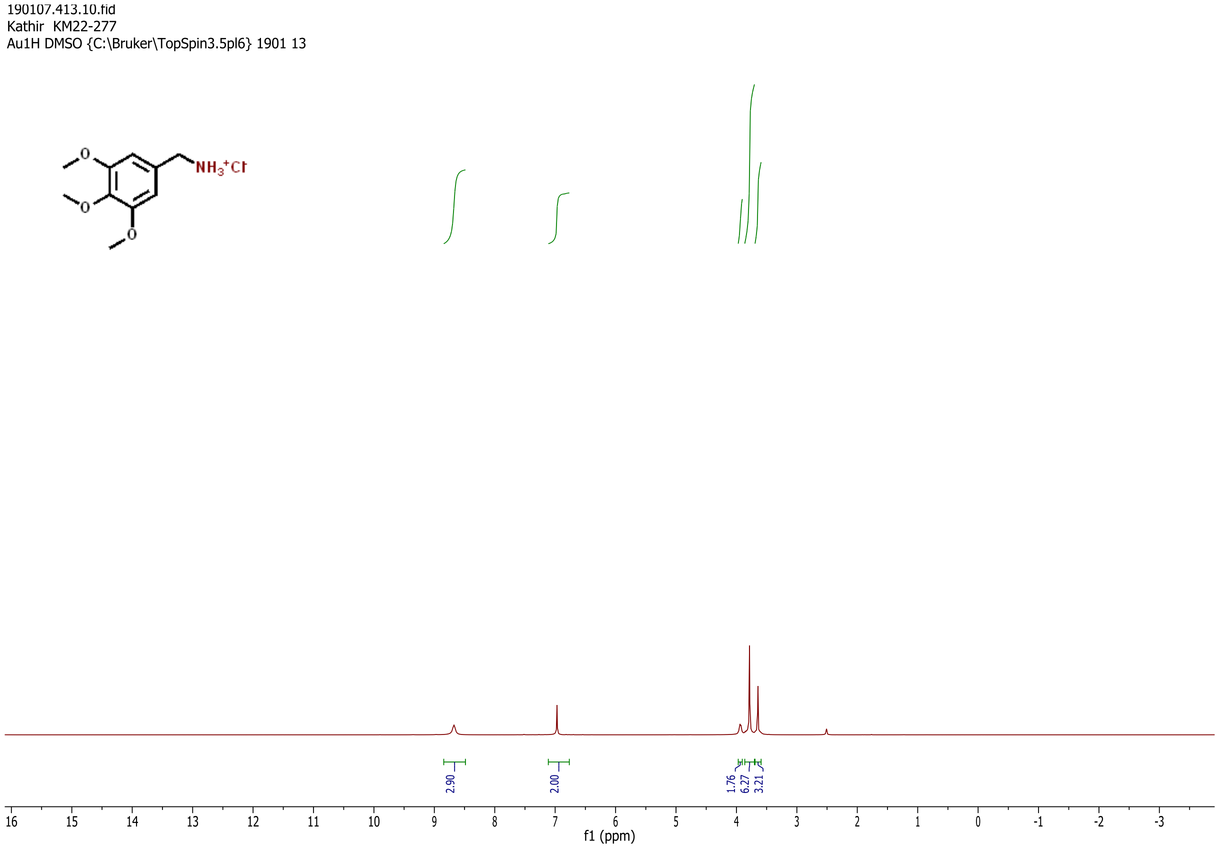

Supplementary Figure 19. ^{1}H NMR (**400 MHz, DMSO-d_6**)

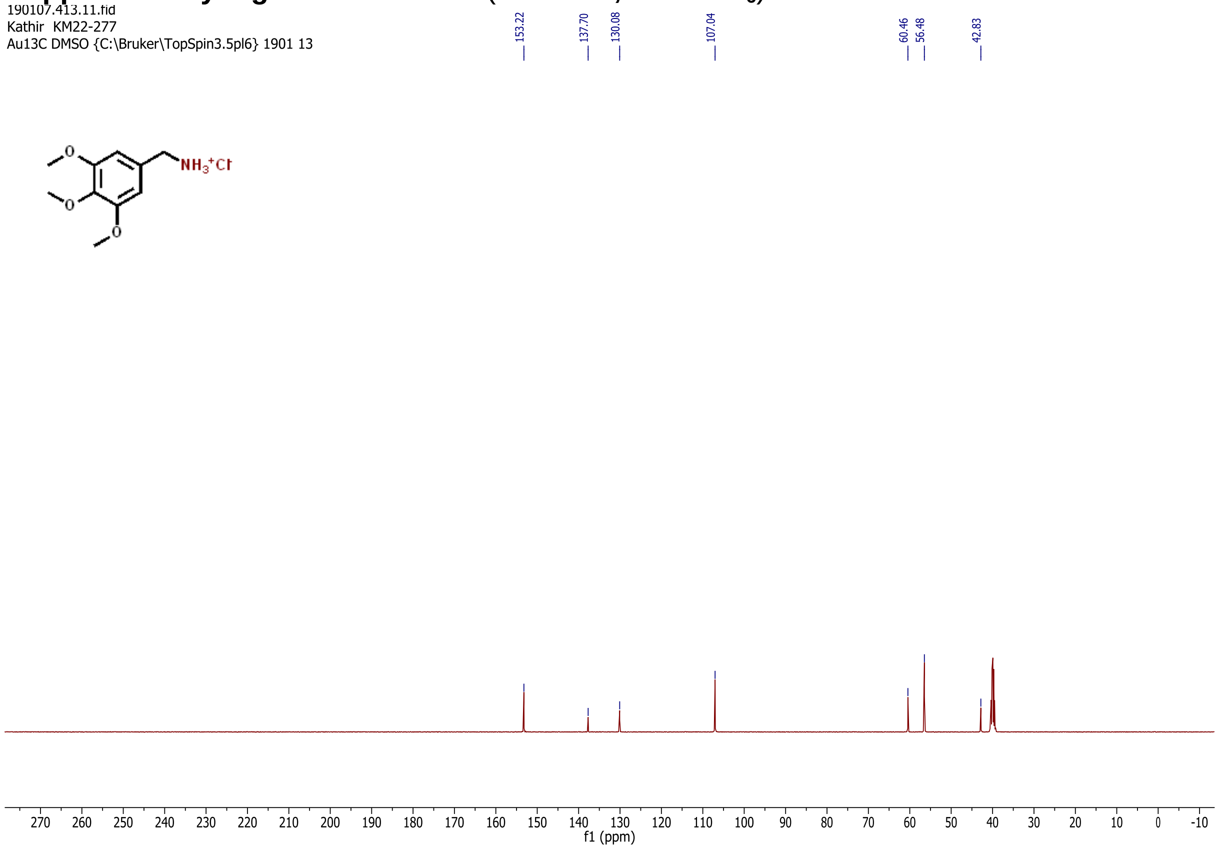

Supplementary Figure 20. ^{13}C NMR (**101 MHz, DMSO-d_6**)

190107.408.10.fid
Kathir KM22-191
Au1H DMSO {C:\Bruker\TopSpin3.5pl6} 1901 8

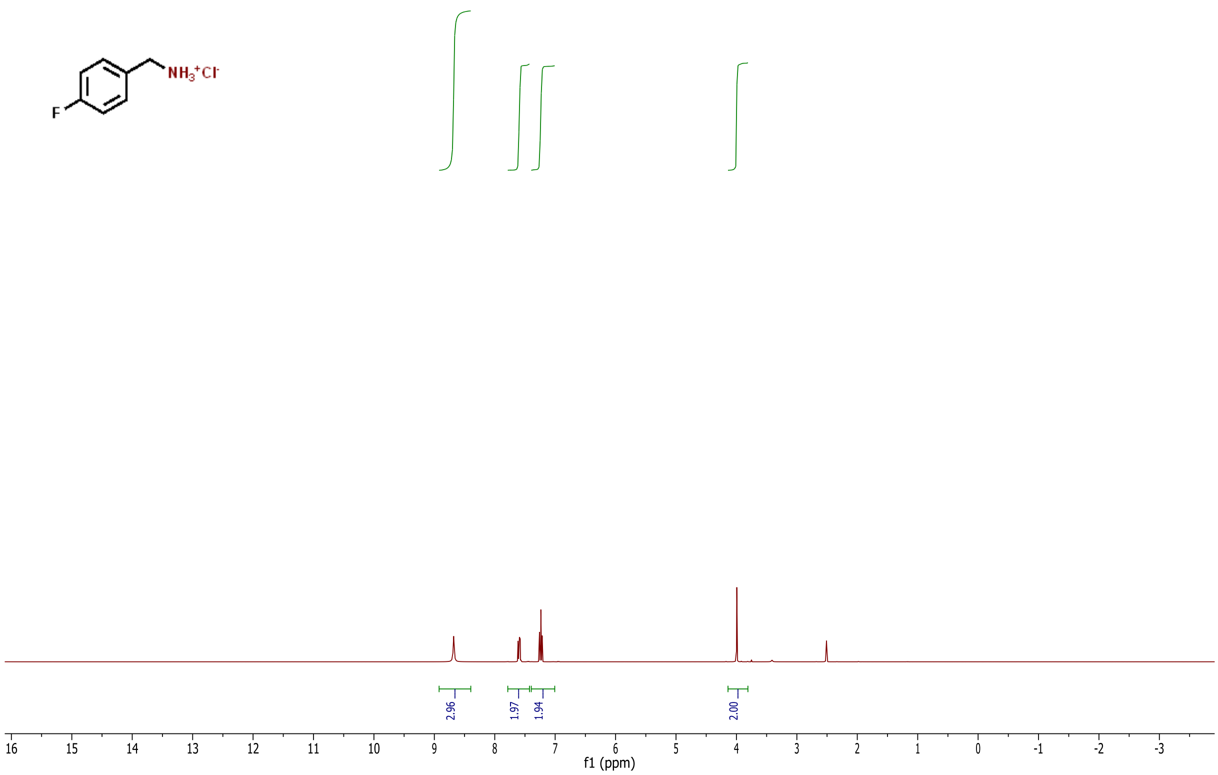

Supplementary Figure 21. ^{1}H NMR (**400 MHz, DMSO-*d*$_6$**)

190107.408.11.fid
Kathir KM22-191
Au13C DMSO {C:\Bruker\TopSpin3.5pl6} 1901 8

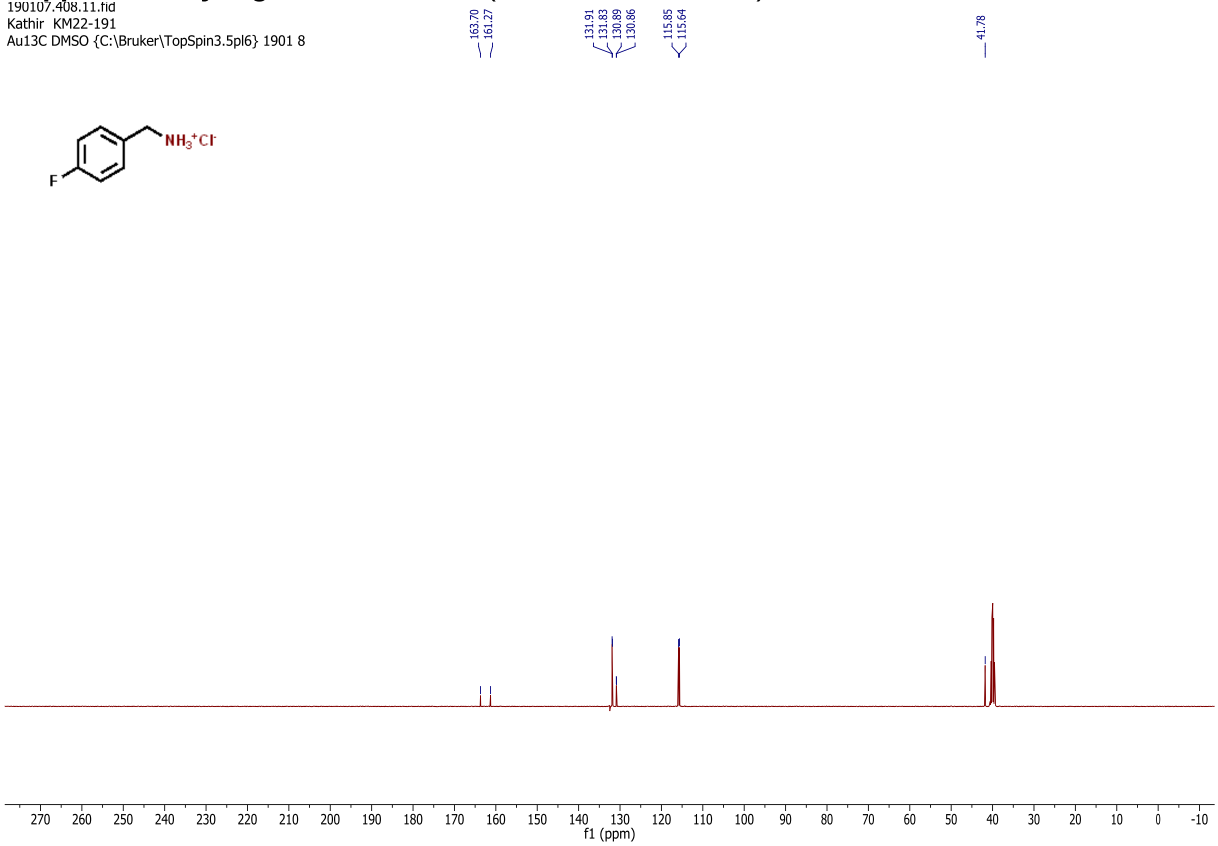

Supplementary Figure 22. ^{13}C NMR (**101 MHz, DMSO-*d*$_6$**)

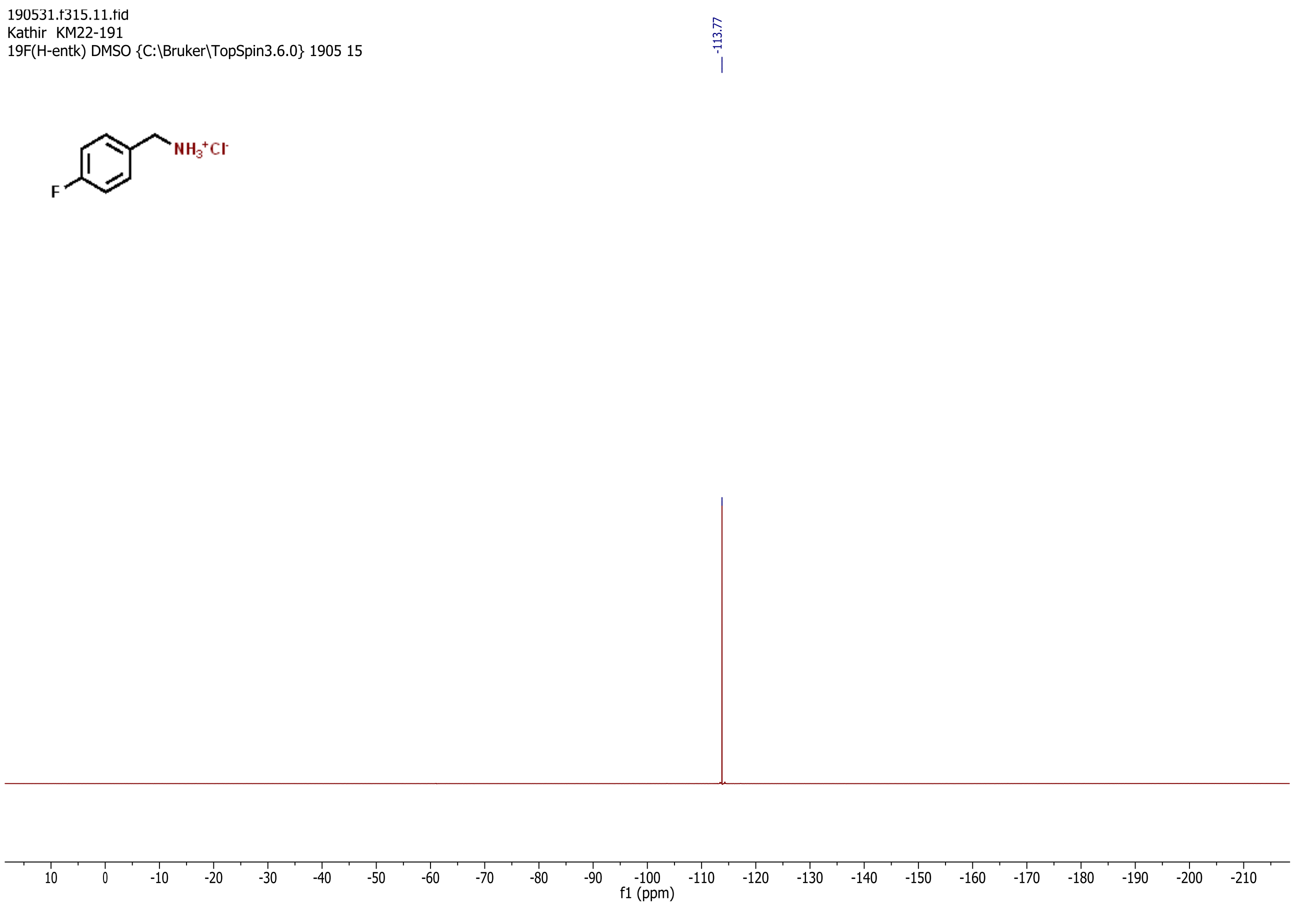

Supplementary Figure 23. ^{19}F NMR **(282 MHz, DMSO-d_6)**

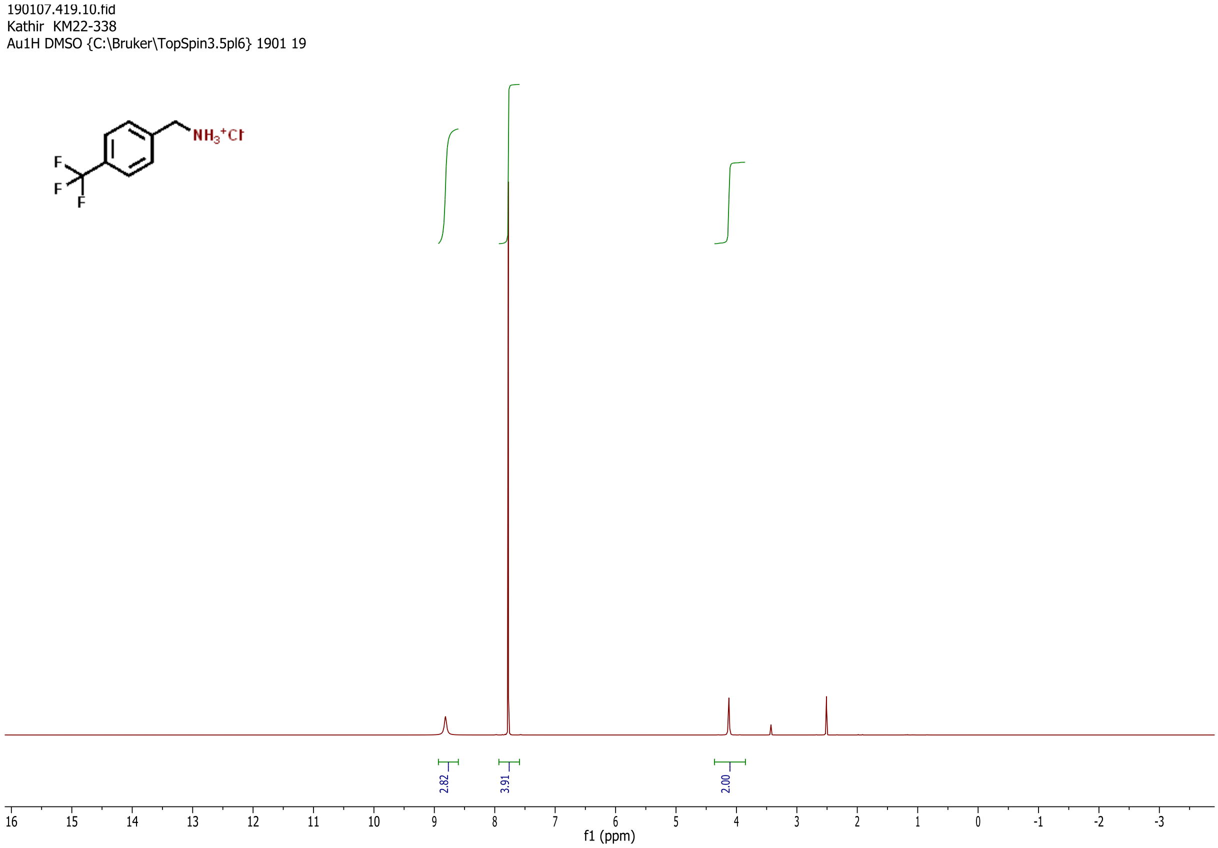

Supplementary Figure 24. ^{1}H NMR (**400 MHz, DMSO-*d*$_6$**)

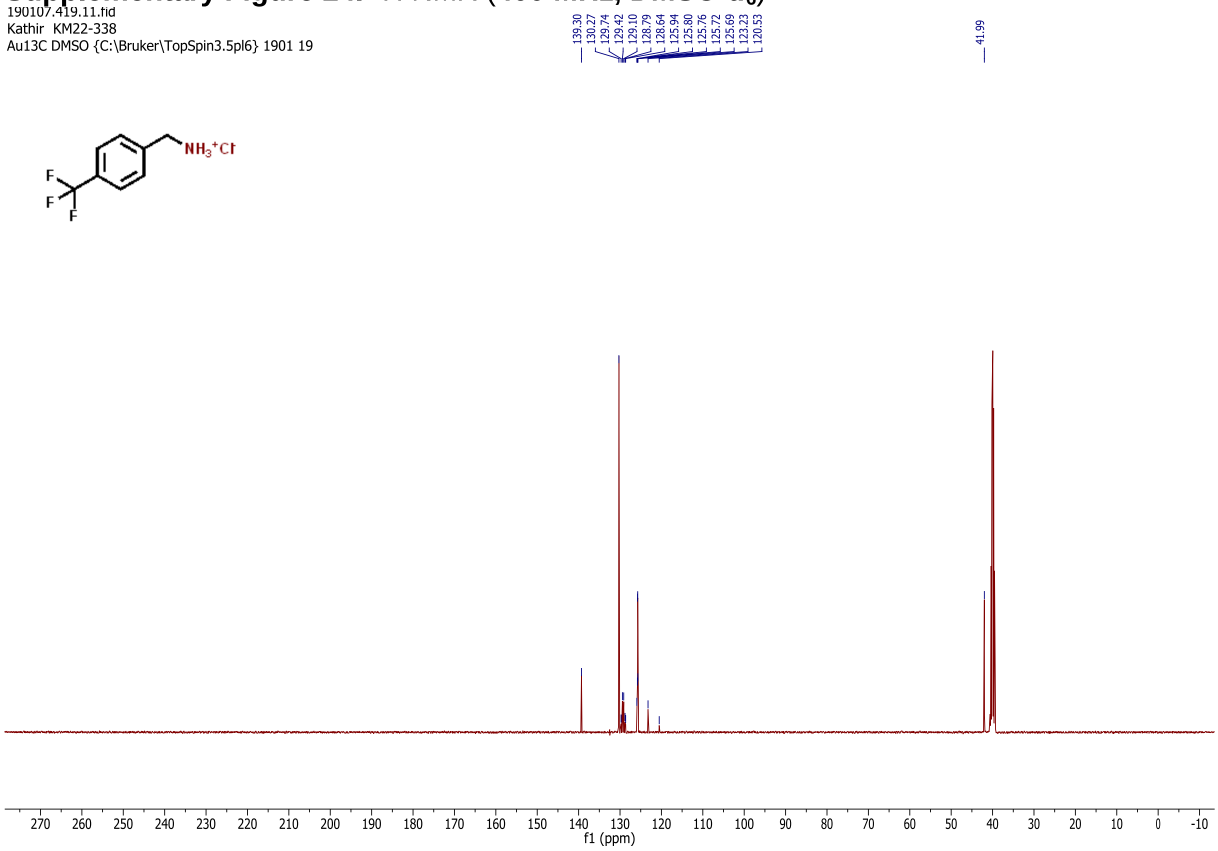

Supplementary Figure 25. ^{13}C NMR (**101 MHz, DMSO-*d*$_6$**)

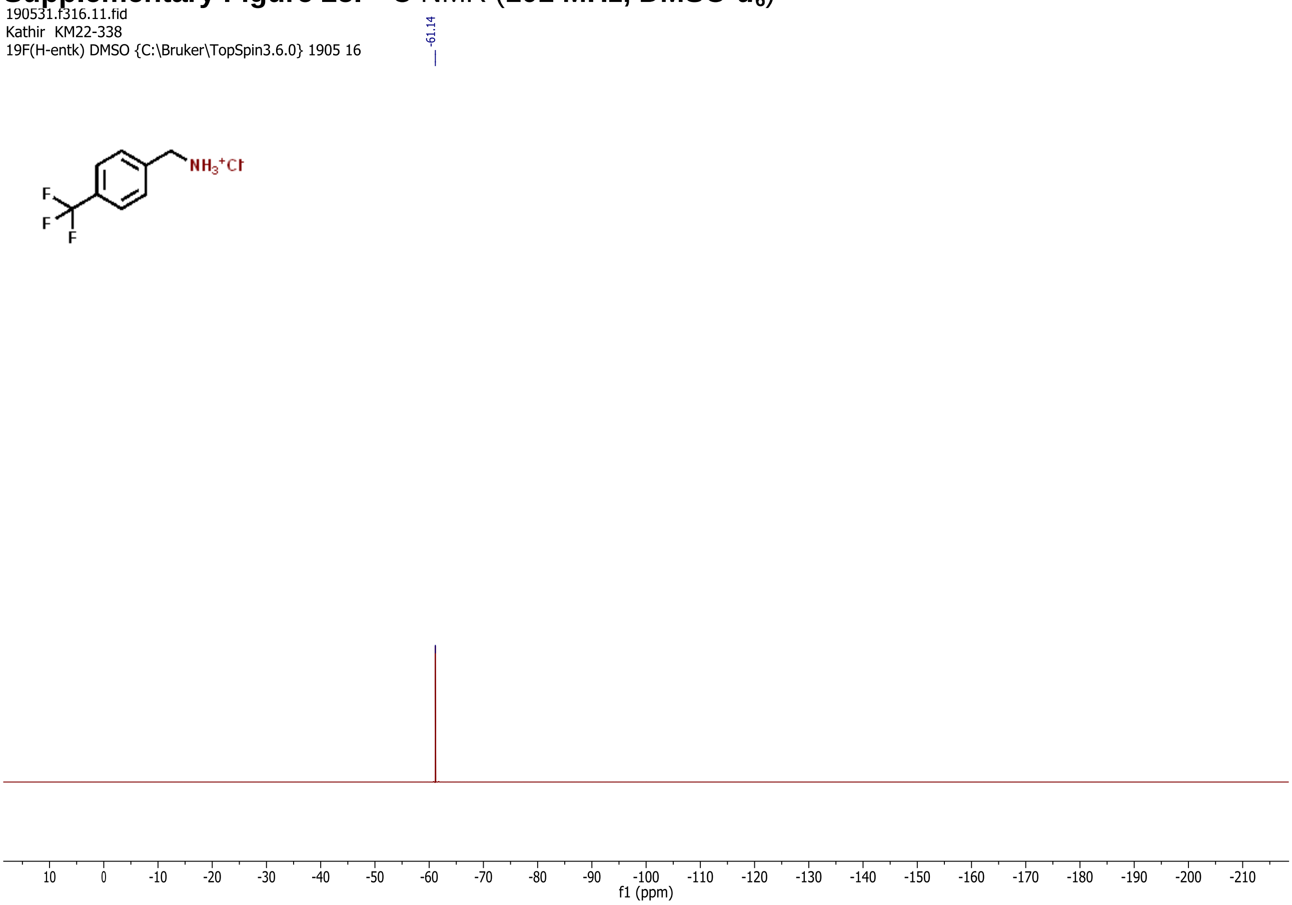

Supplementary Figure 26. ^{19}F NMR **(282 MHz, DMSO-*d*$_6$)**

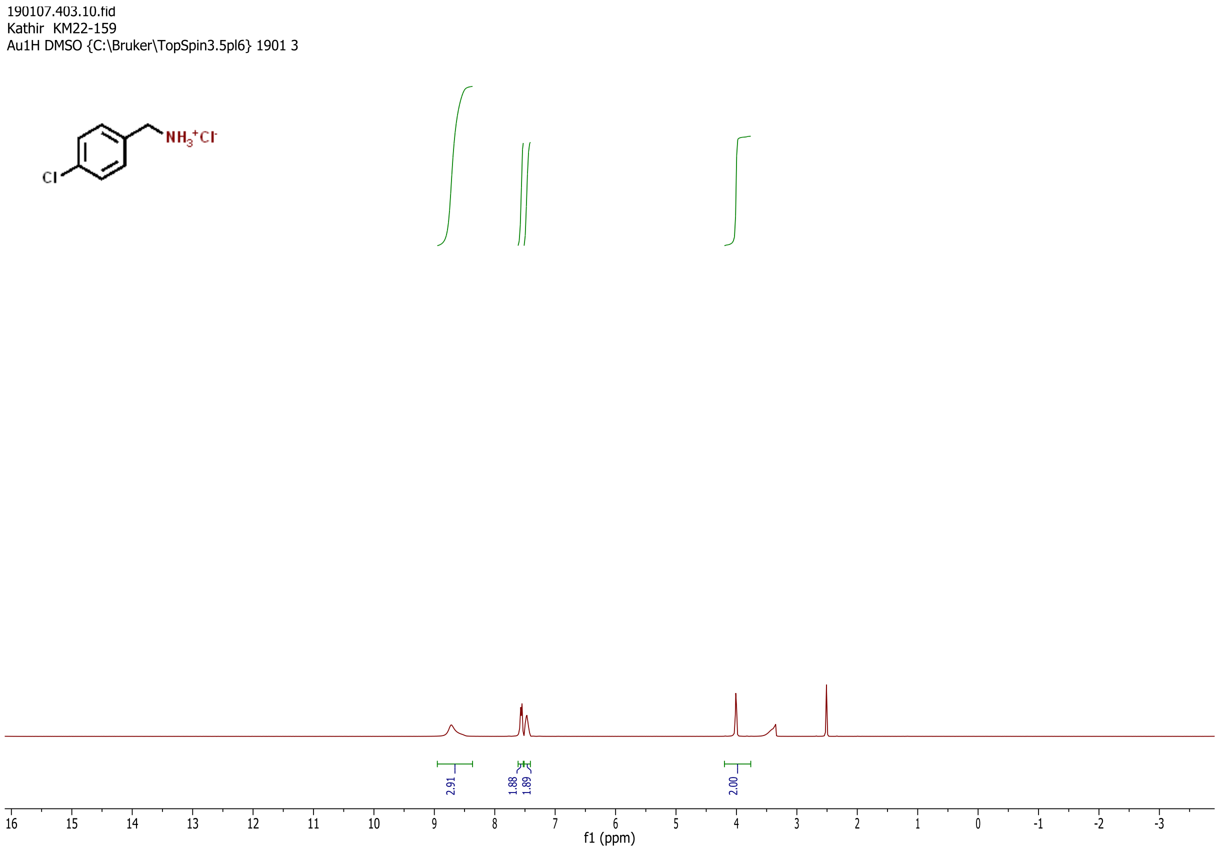

Supplementary Figure 27. ^{1}H NMR (**400 MHz, DMSO-d_6**)

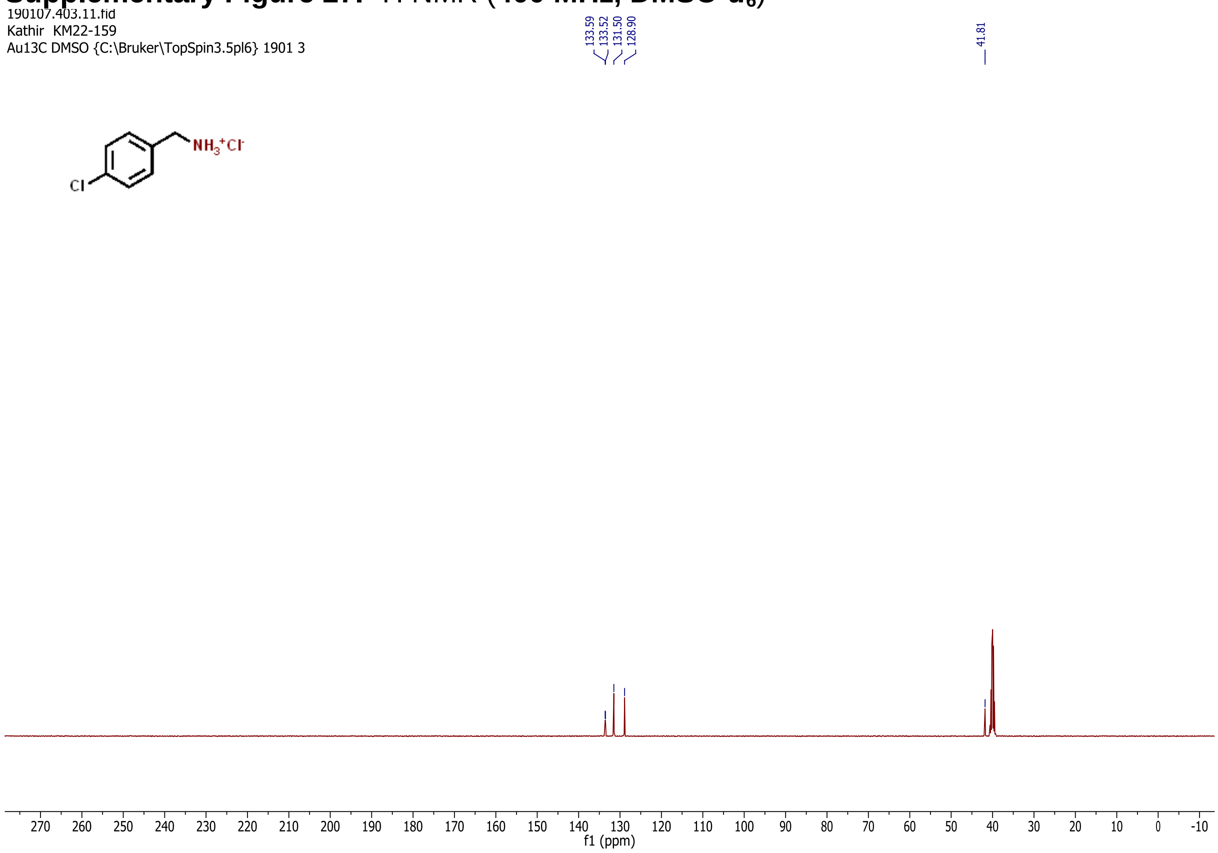

Supplementary Figure 28. ^{13}C NMR (**101 MHz, DMSO-d_6**)

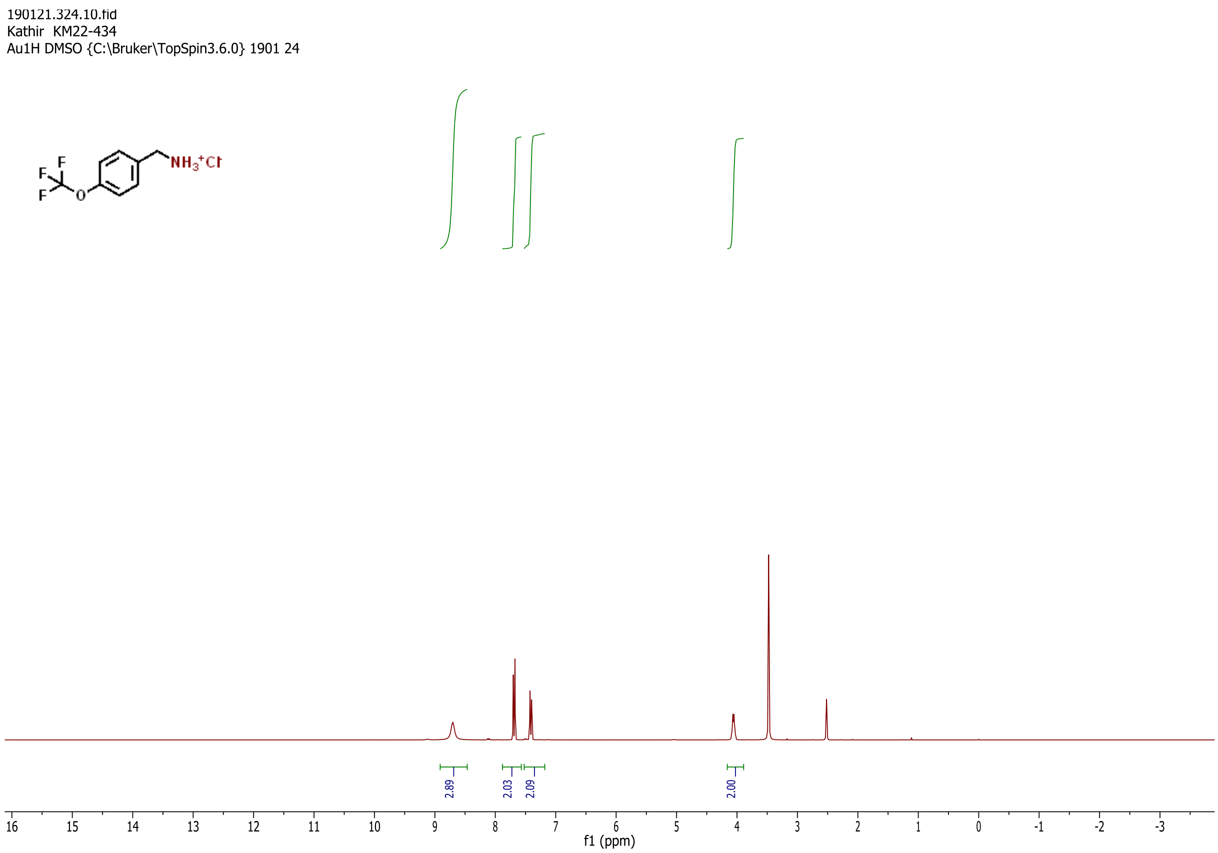

Supplementary Figure 29. ^{1}H NMR (**300 MHz, DMSO-d_6**)

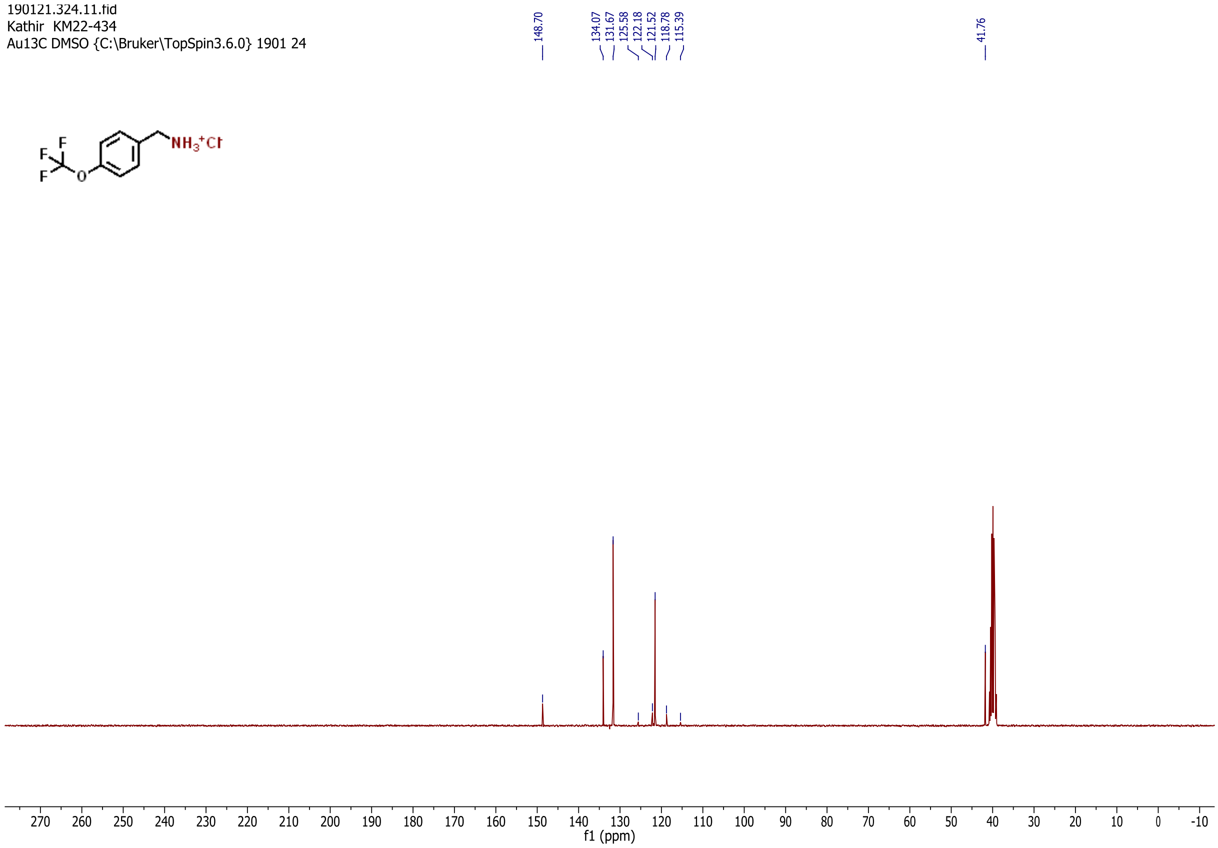

Supplementary Figure 30. ^{13}C NMR (**75 MHz, DMSO-*d*$_6$**)

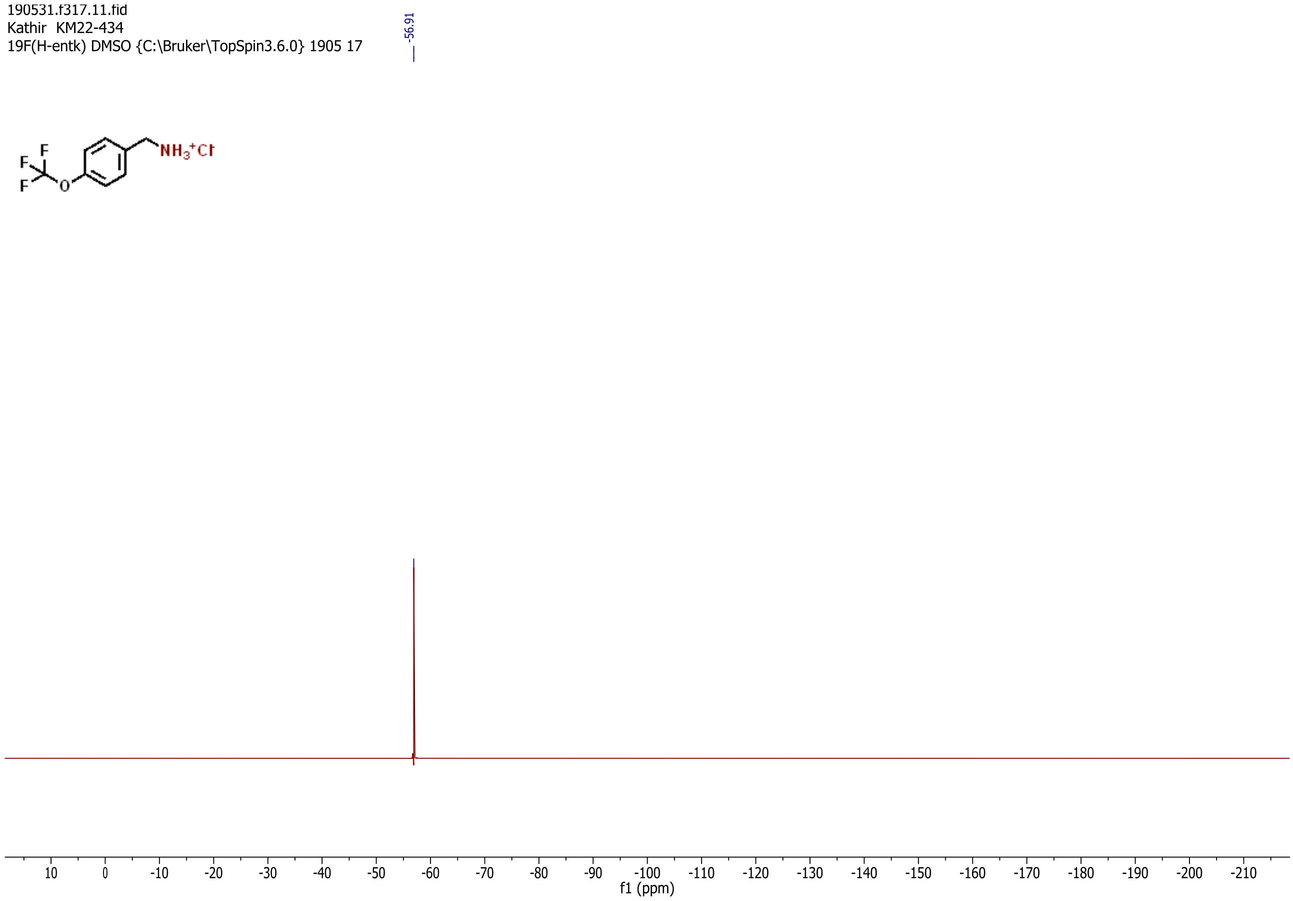

Supplementary Figure 31. ^{19}F NMR **(282 MHz, DMSO-*d*$_6$)**

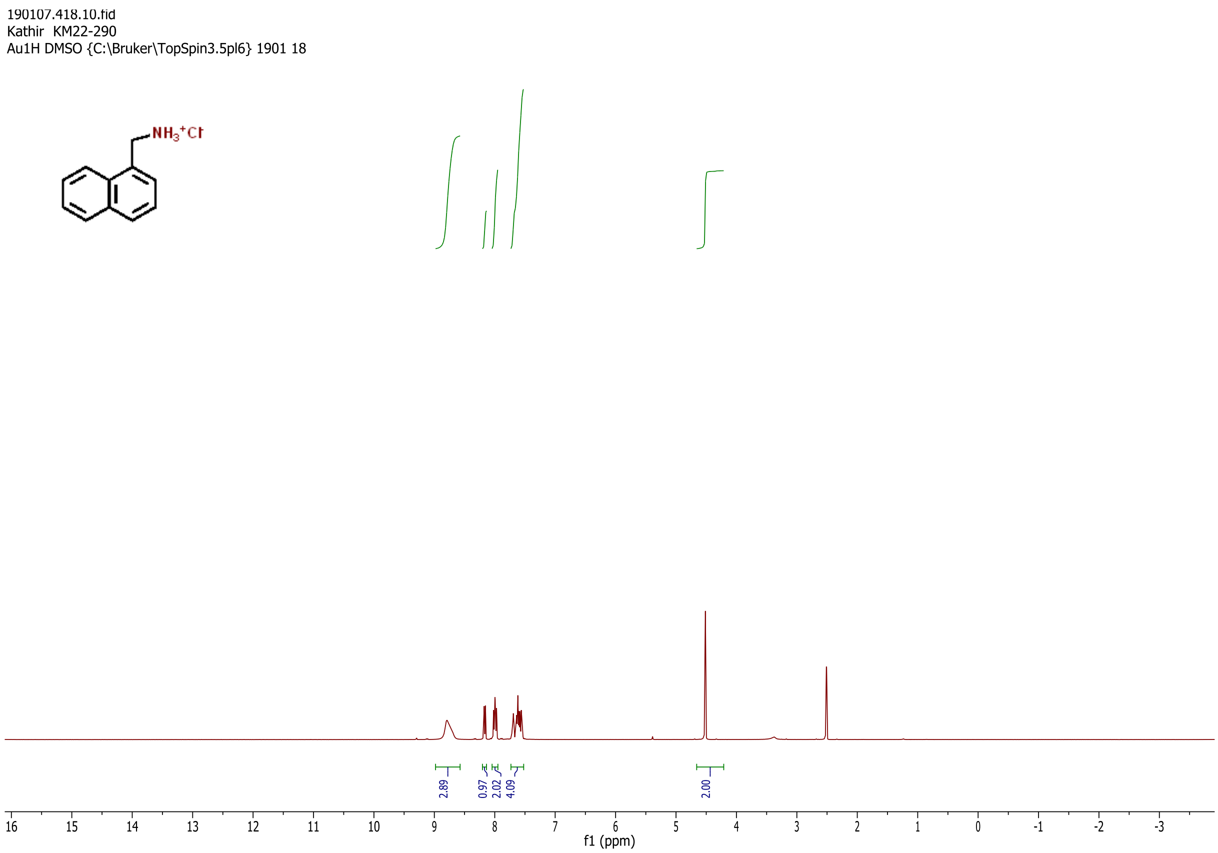

Supplementary Figure 32. ^{1}H NMR (**400 MHz, DMSO-*d*$_{6}$**)

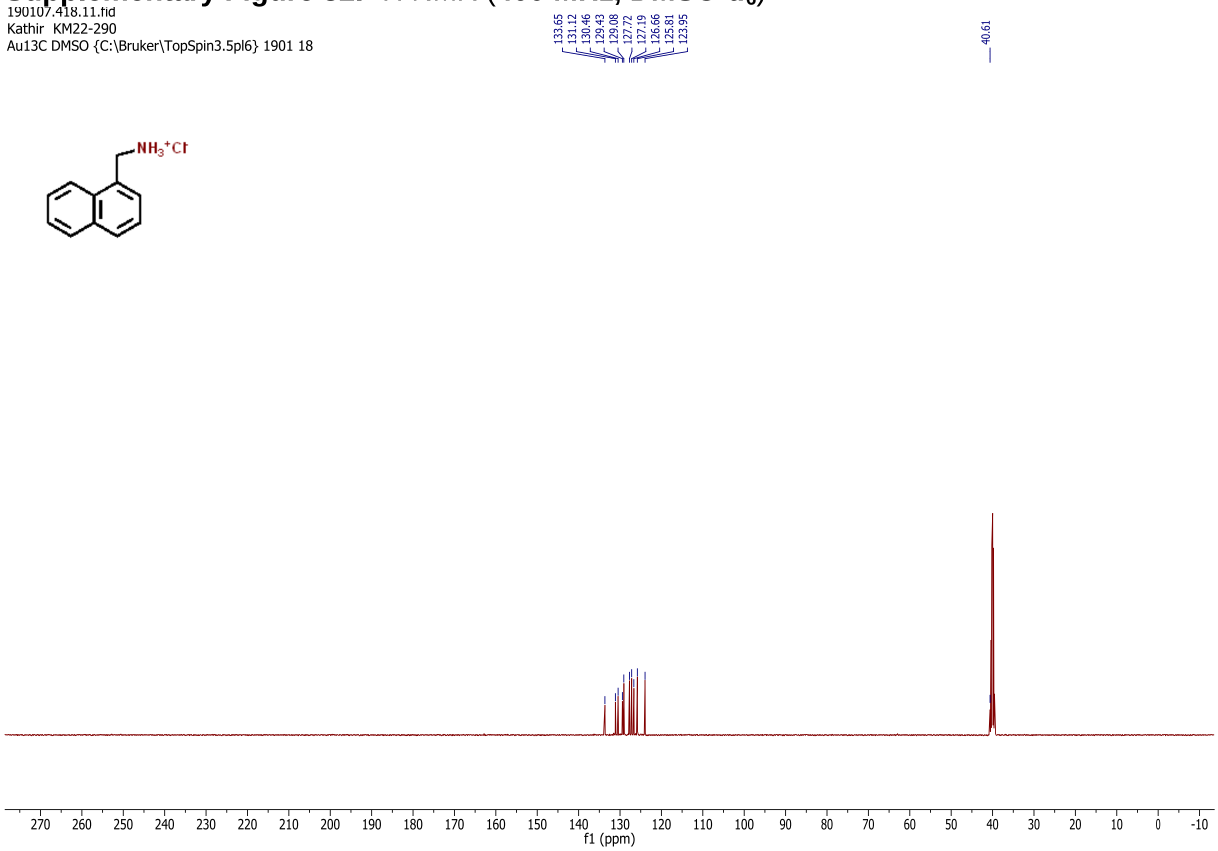

Supplementary Figure 33. ^{13}C NMR (**101 MHz, DMSO-*d*$_{6}$**)

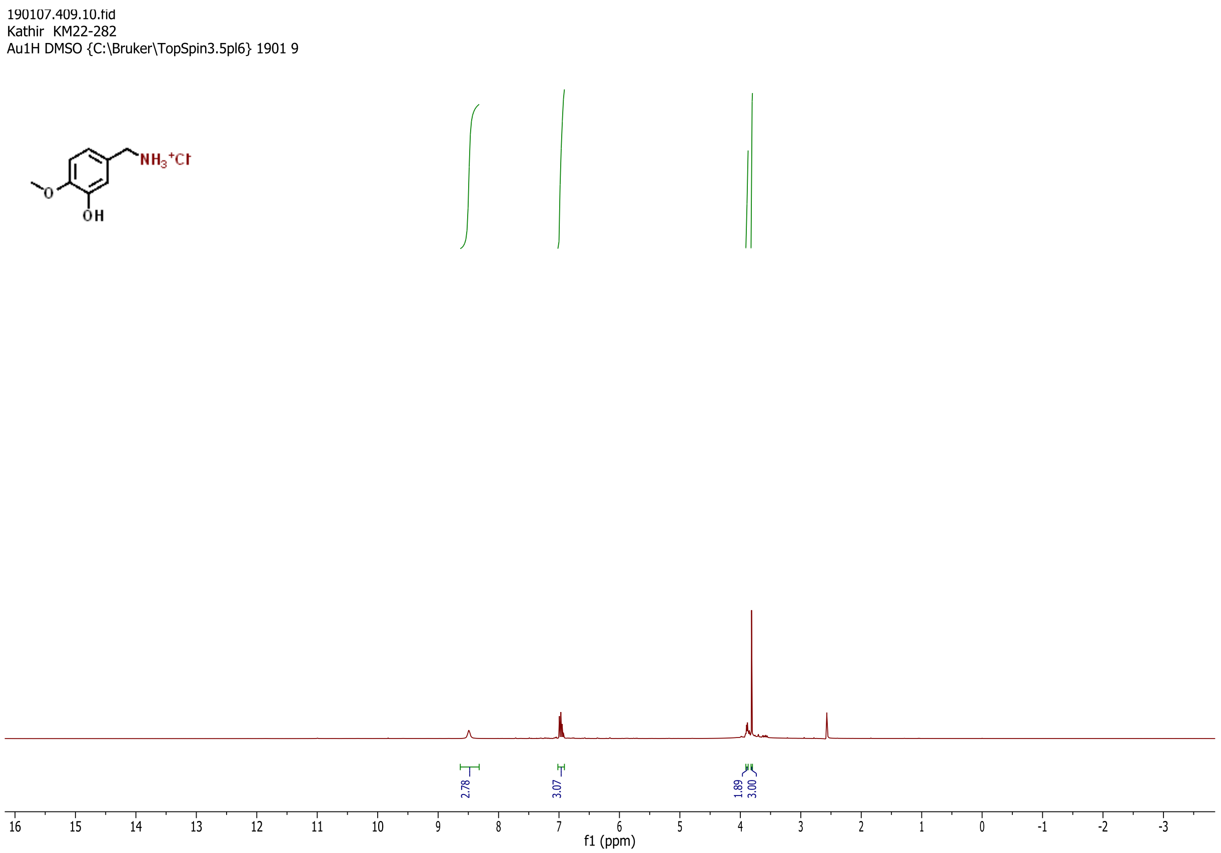

Supplementary Figure 34. ^{1}H NMR (**400 MHz, DMSO-d_6**)

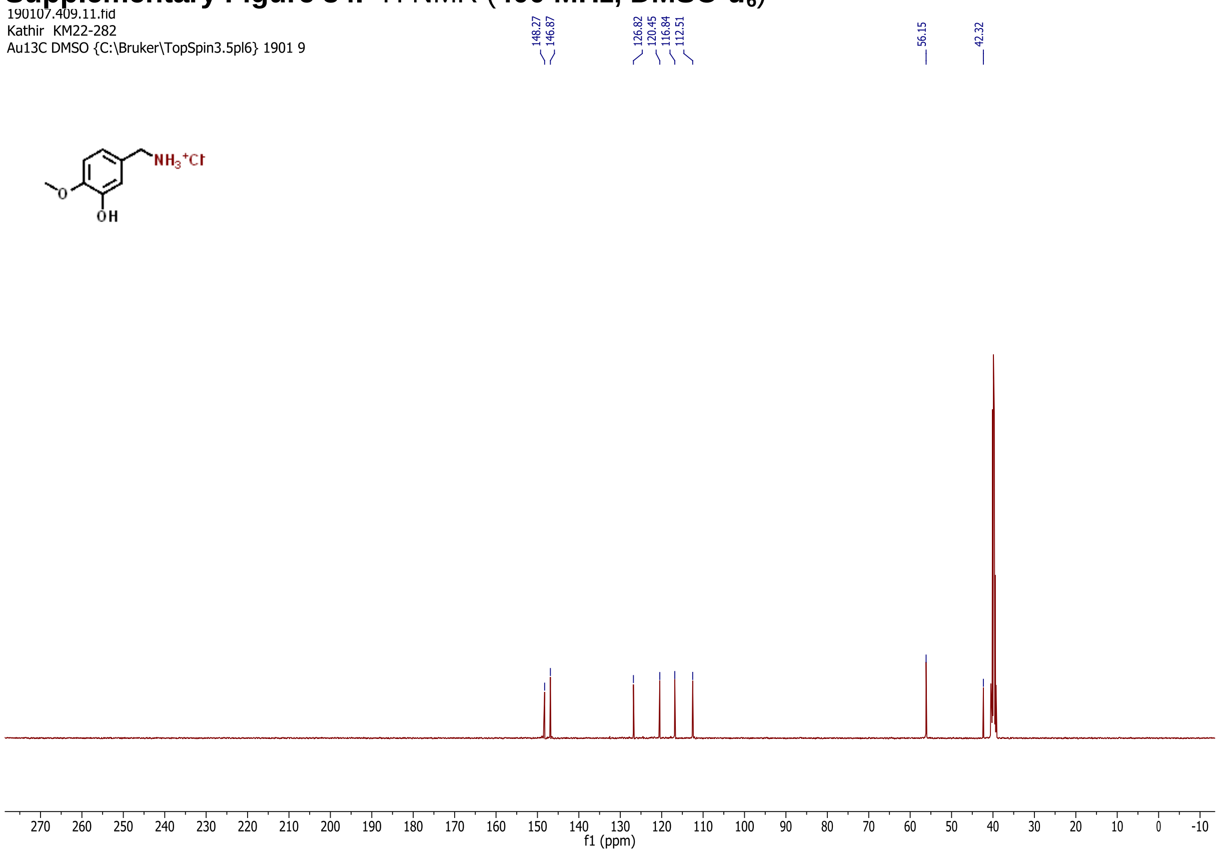

Supplementary Figure 35. ^{13}C NMR (**101 MHz, DMSO-d_6**)

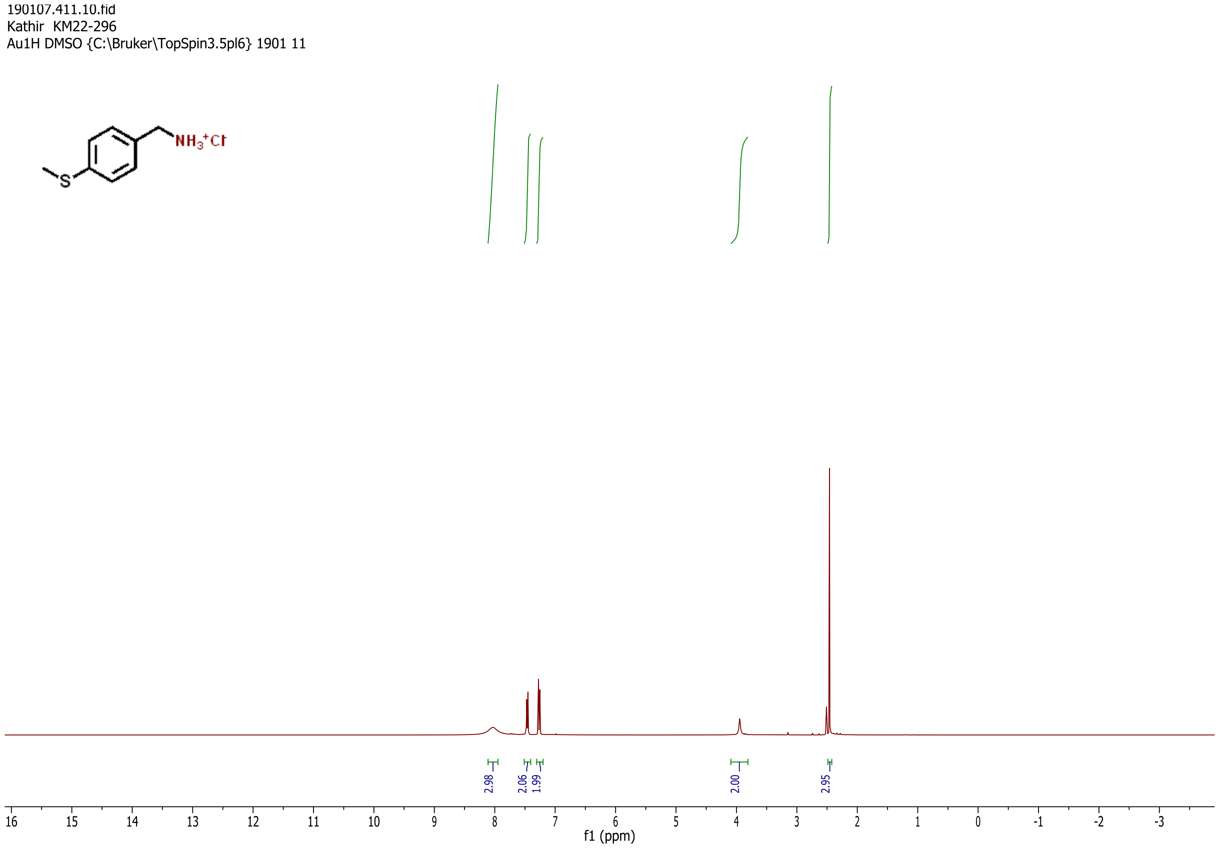

Supplementary Figure 36. ^{1}H NMR (**400 MHz, DMSO-*d*$_6$**)

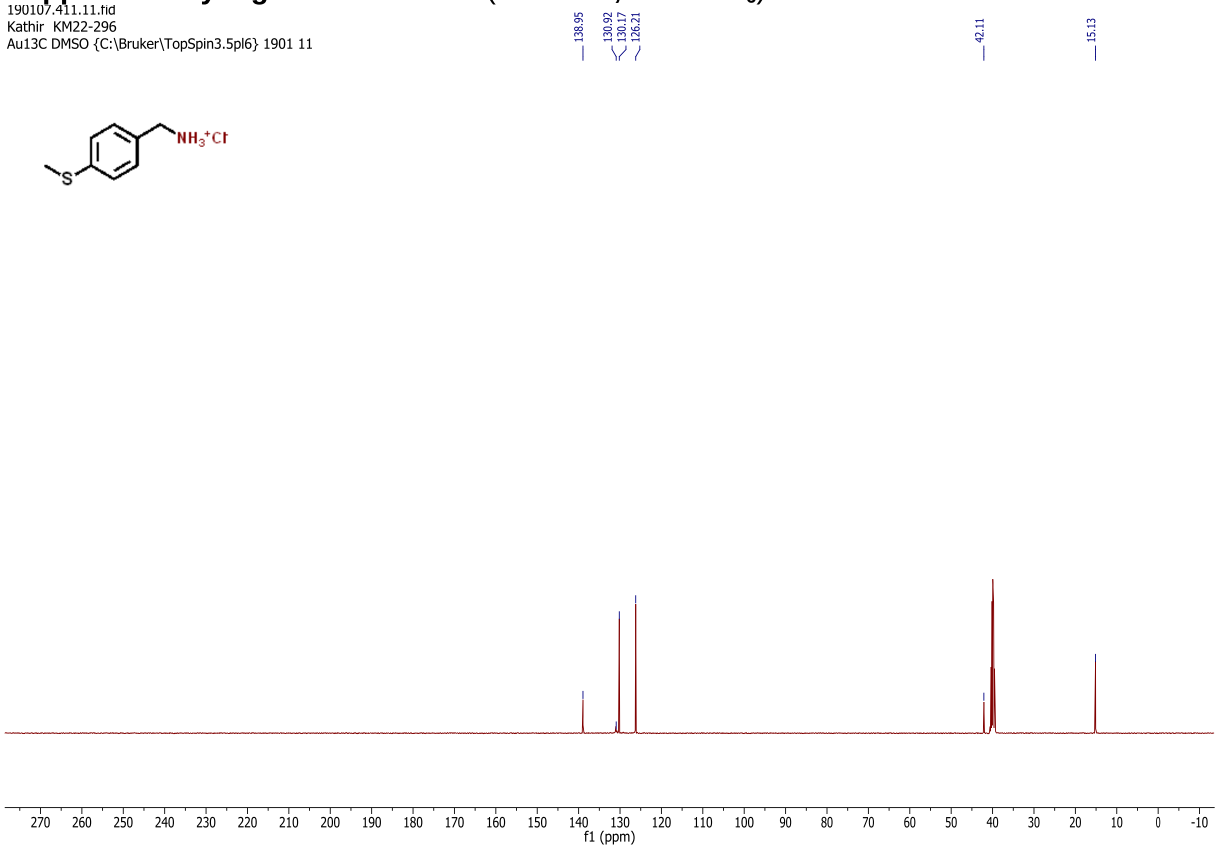

Supplementary Figure 37. ^{13}C NMR (**101 MHz, DMSO-*d*$_6$**)

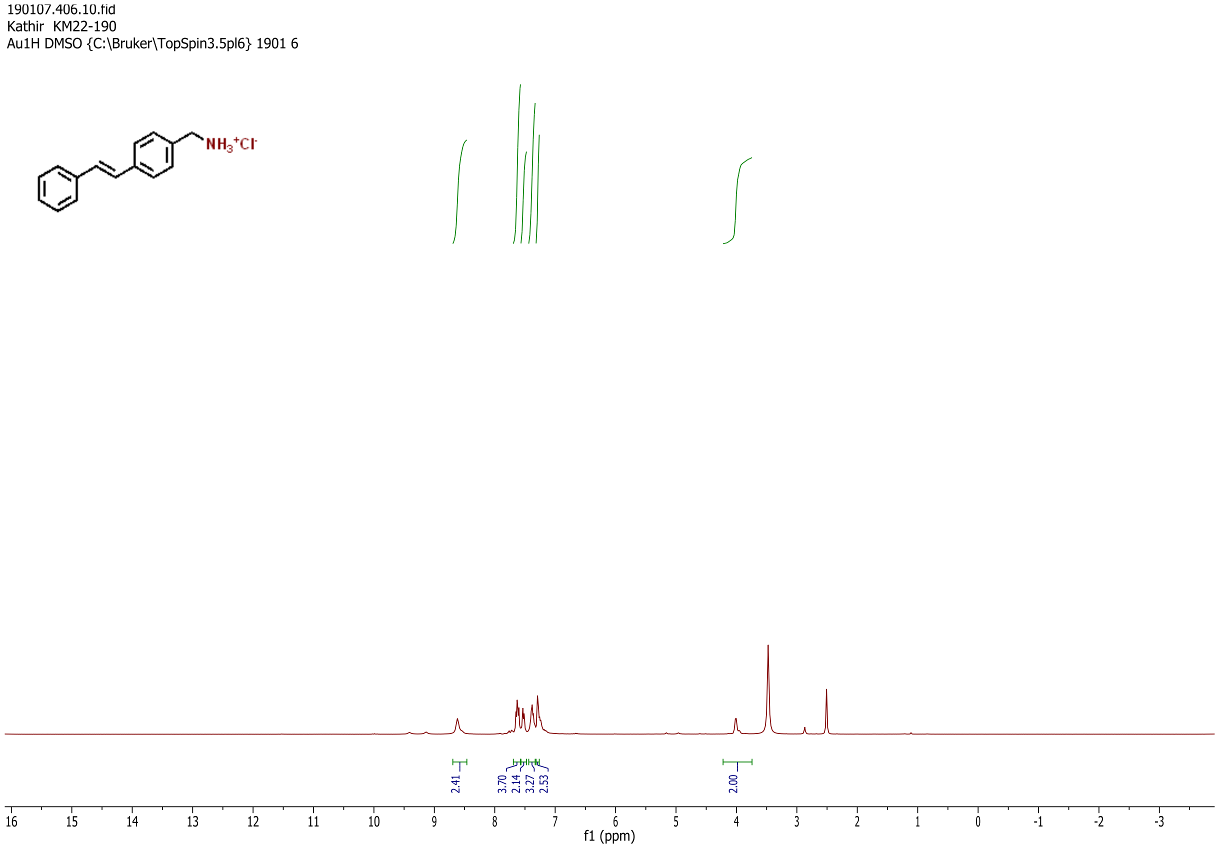

Supplementary Figure 38. ^{1}H NMR (**400 MHz, DMSO-*d*$_6$**)

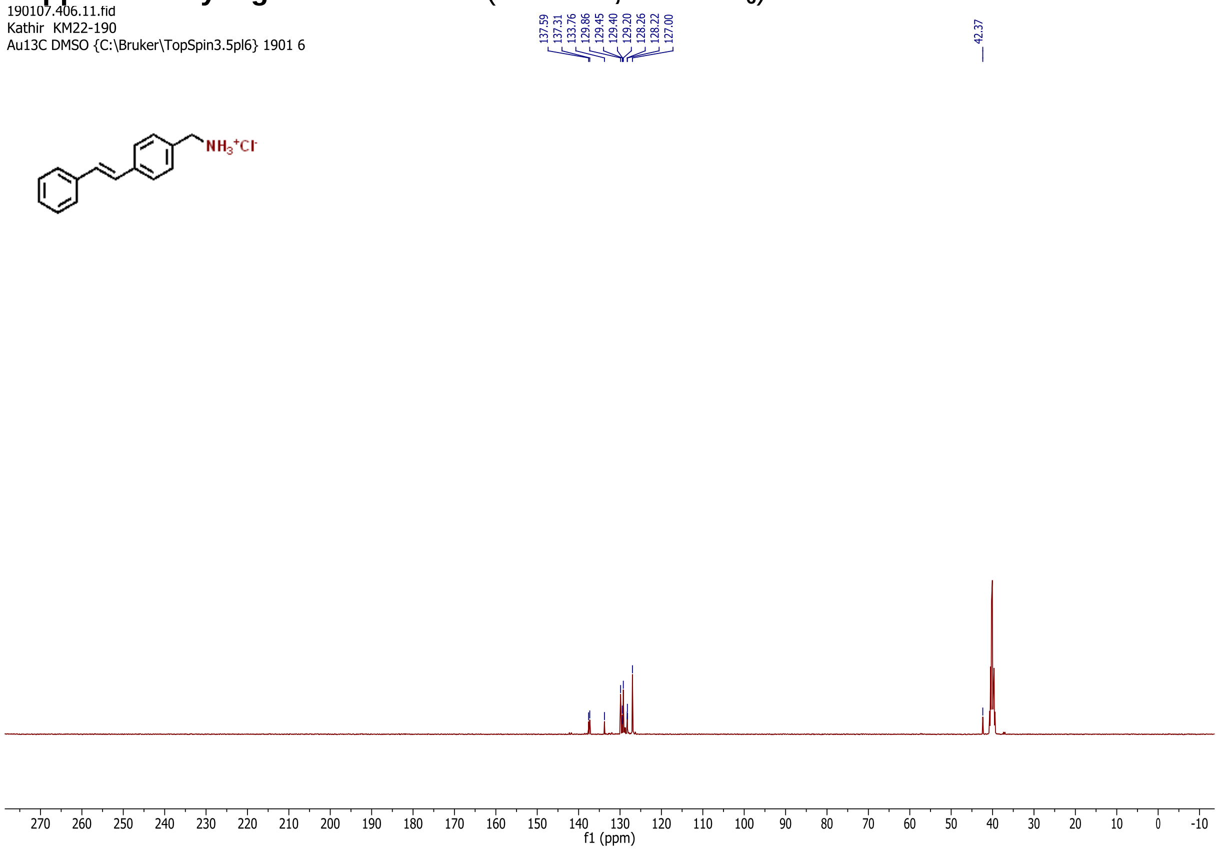

Supplementary Figure 39. ^{13}C NMR (**101 MHz, DMSO-*d*$_6$**)

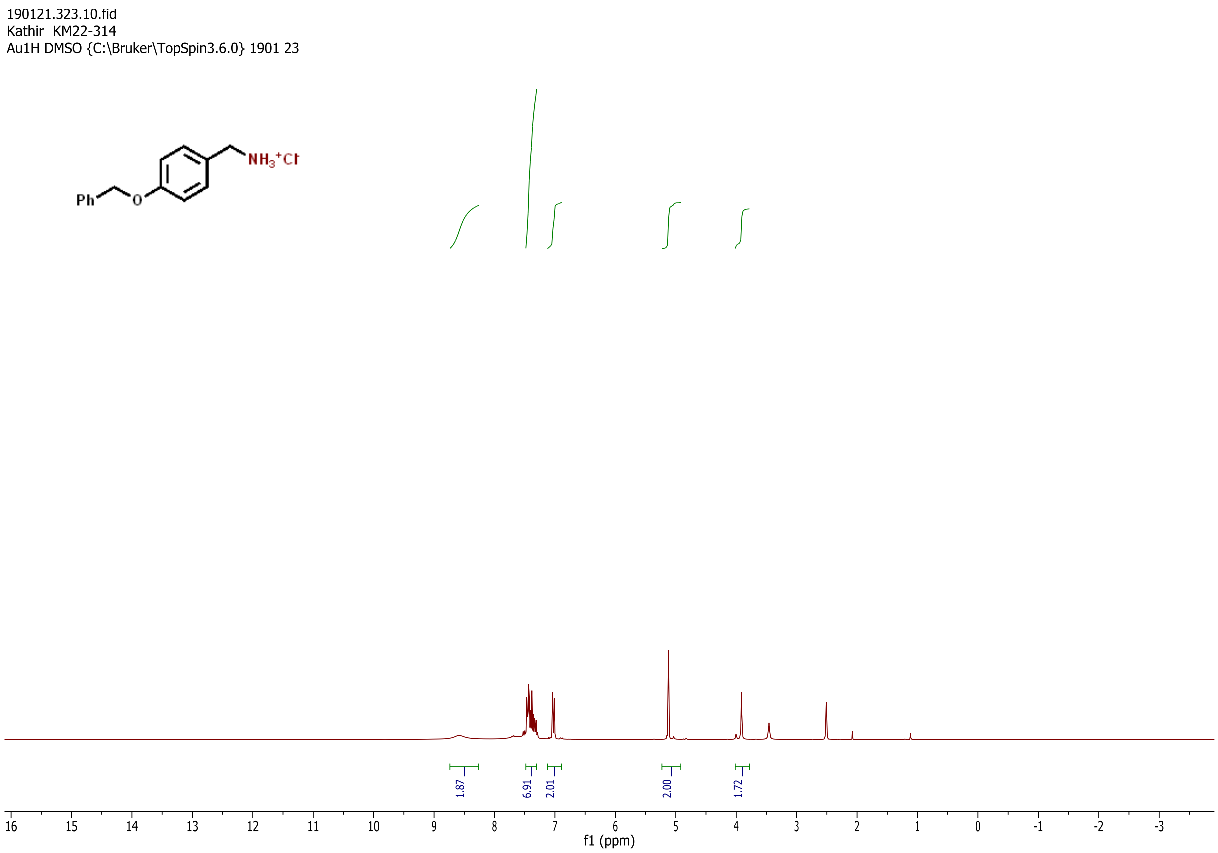

Supplementary Figure 40. ^{1}H NMR (**300 MHz, DMSO-d_6**)

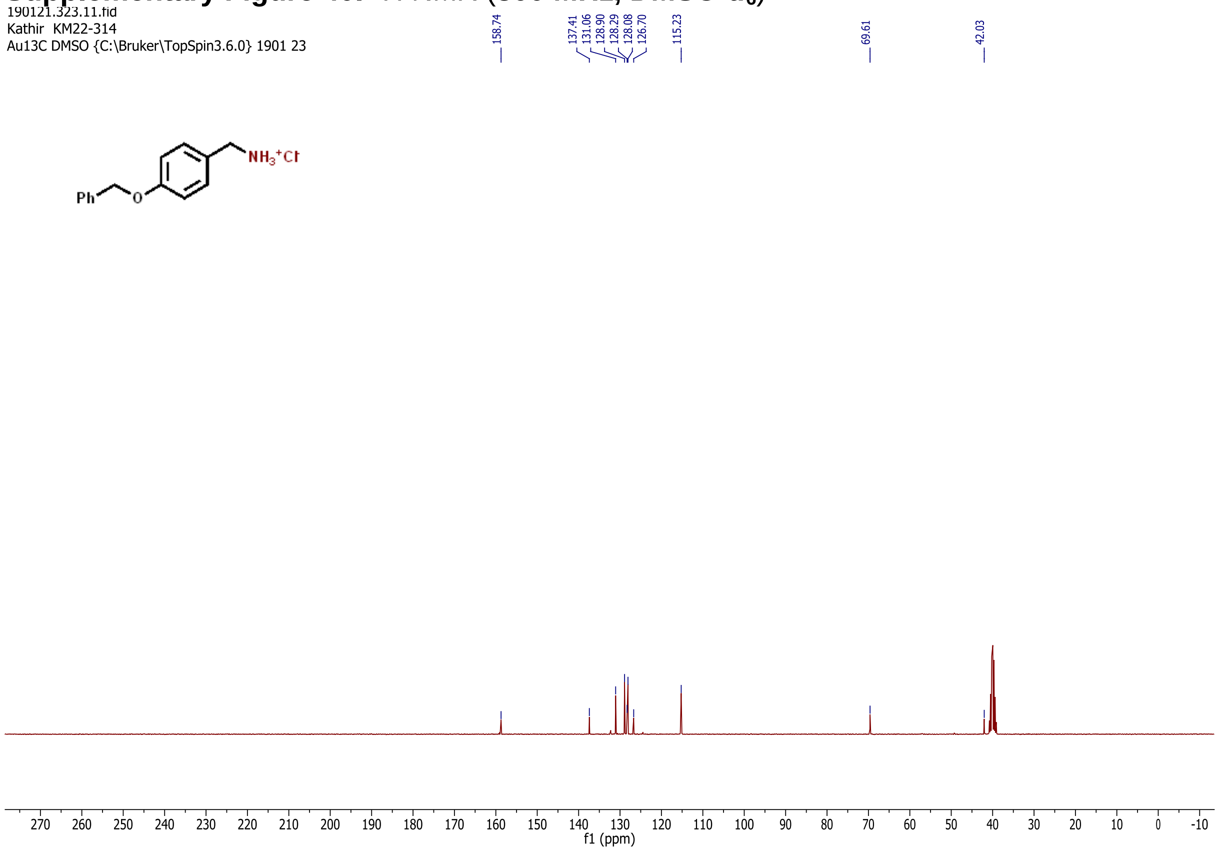

Supplementary Figure 41. ^{13}C NMR (**75 MHz, DMSO-d_6**)

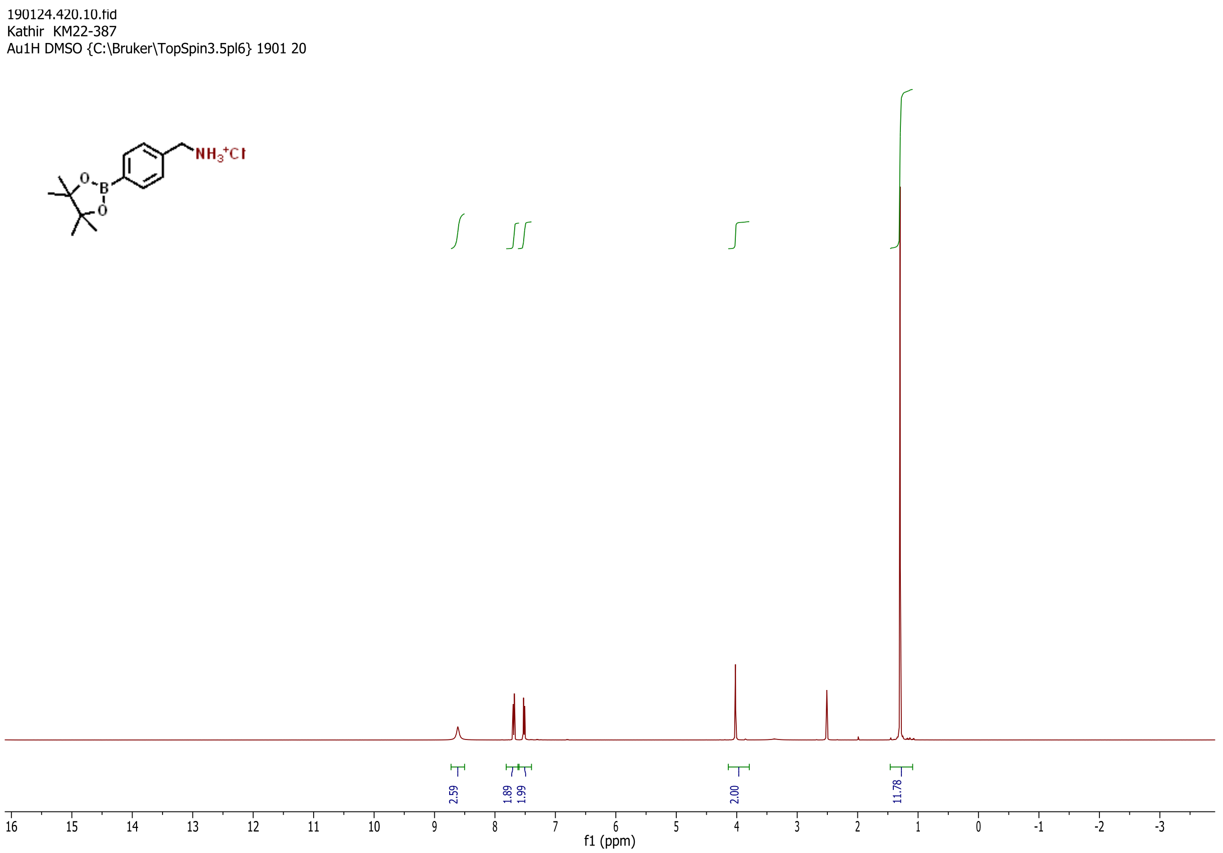

Supplementary Figure 42. ^{1}H NMR (**400 MHz, DMSO-d_6**)

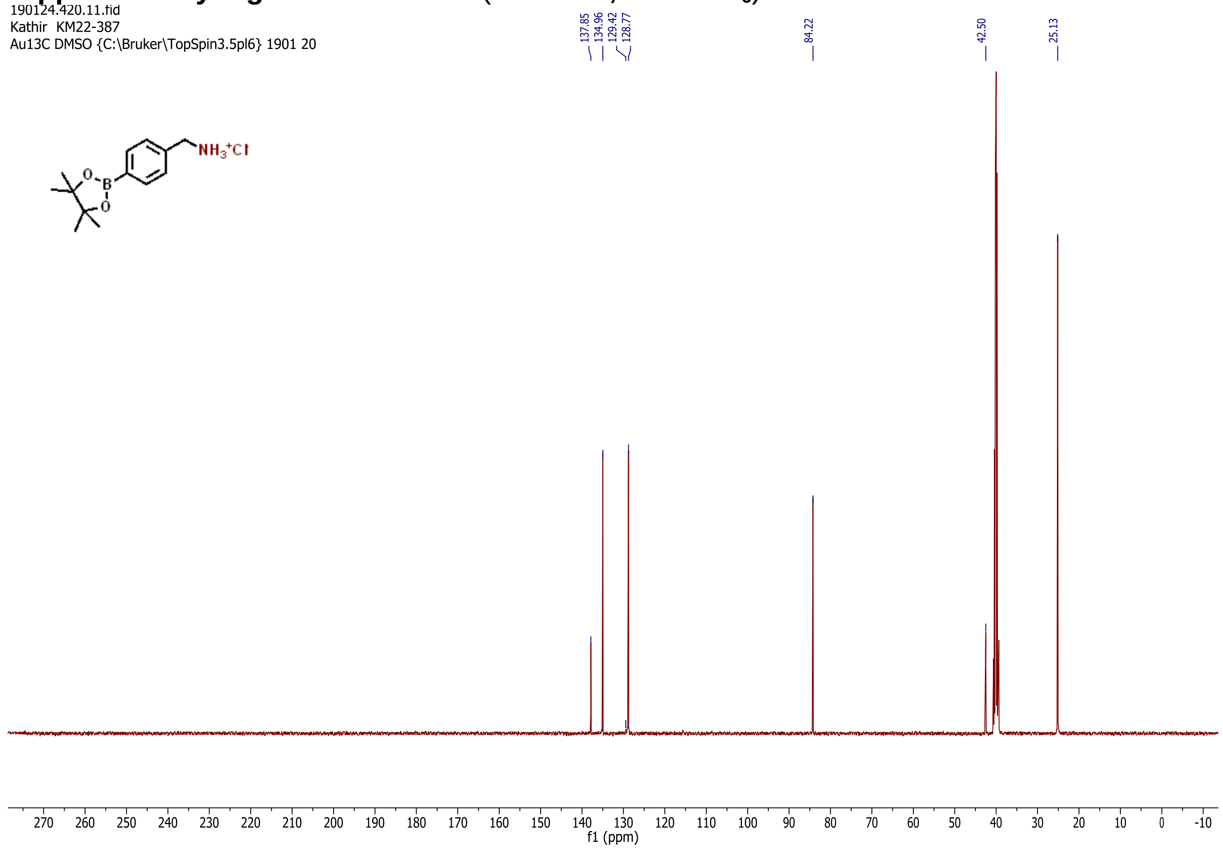

Supplementary Figure 43. ^{13}C NMR (**101 MHz, DMSO-d_6**)

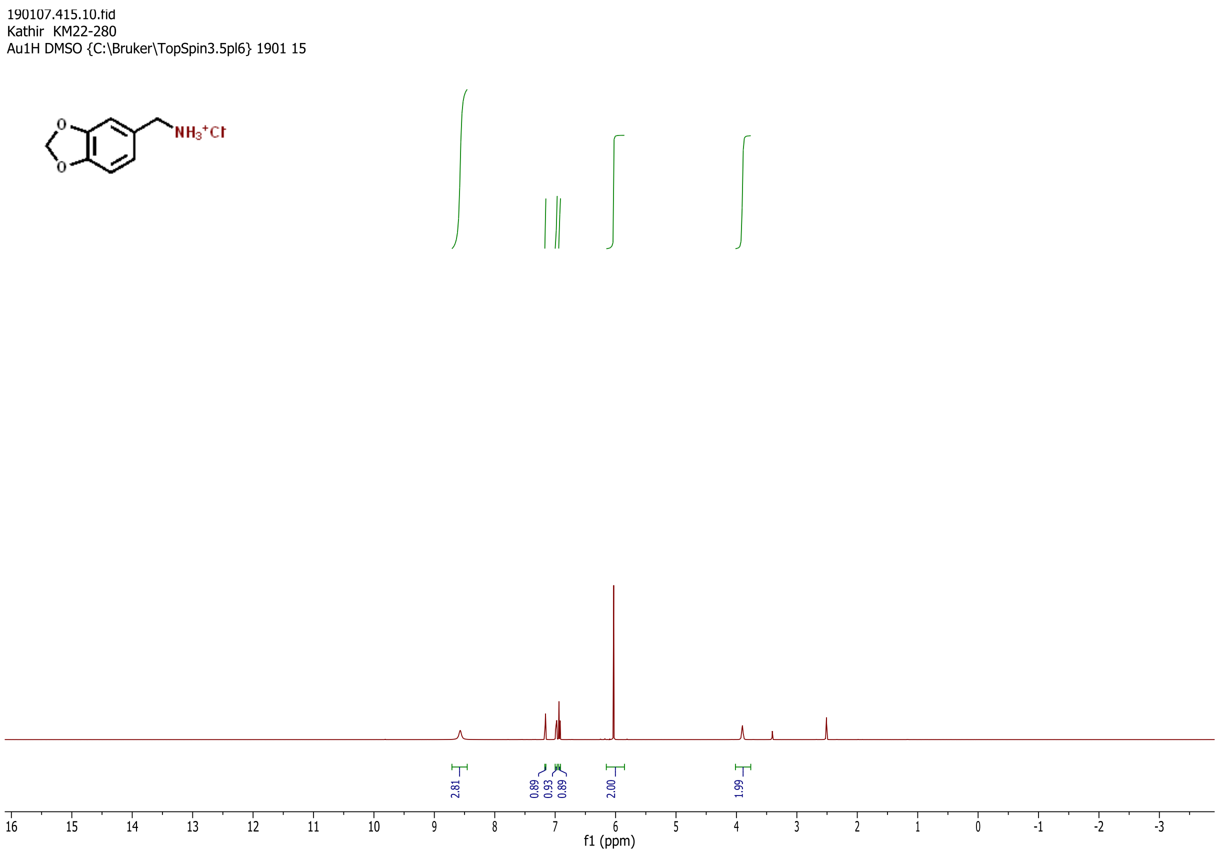

Supplementary Figure 44. ^{1}H NMR (**400 MHz, DMSO-*d*$_6$**)

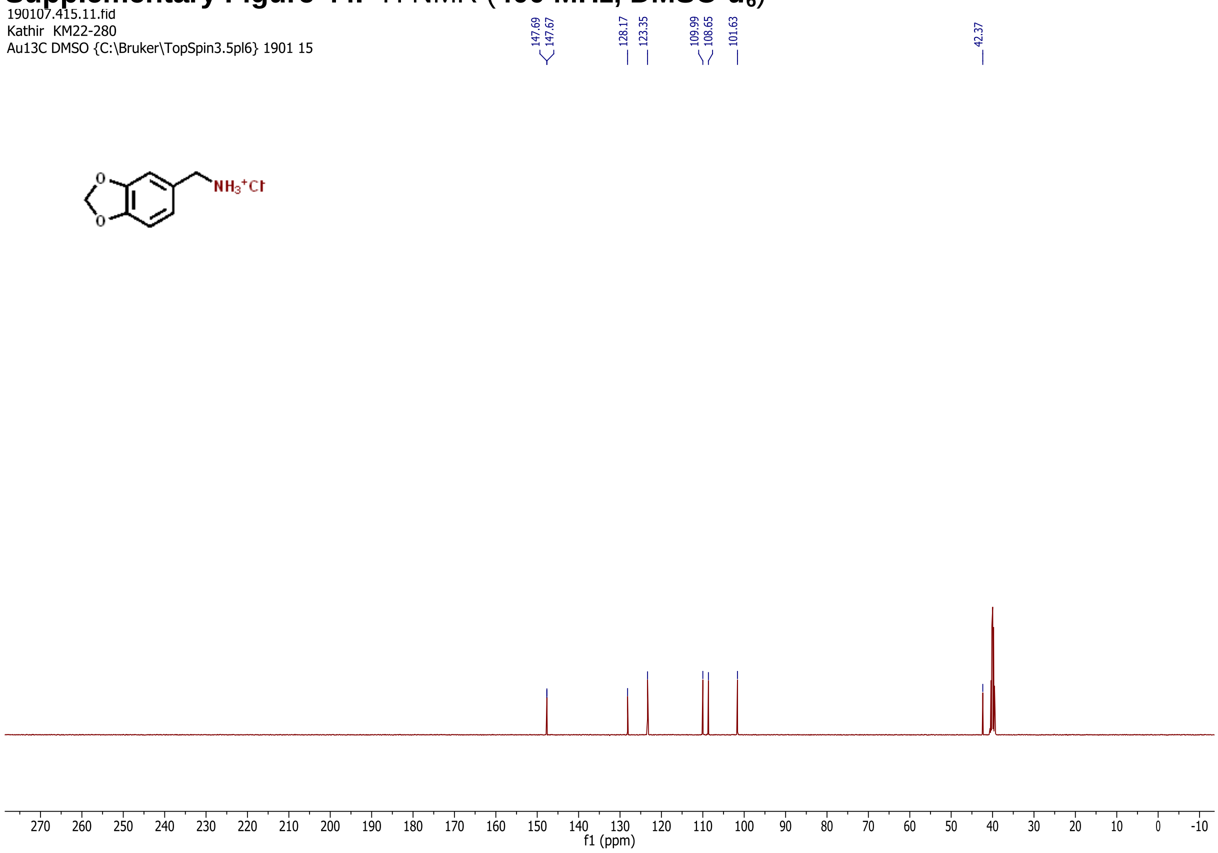

Supplementary Figure 45. ^{13}C NMR (**101 MHz, DMSO-*d*$_6$**)

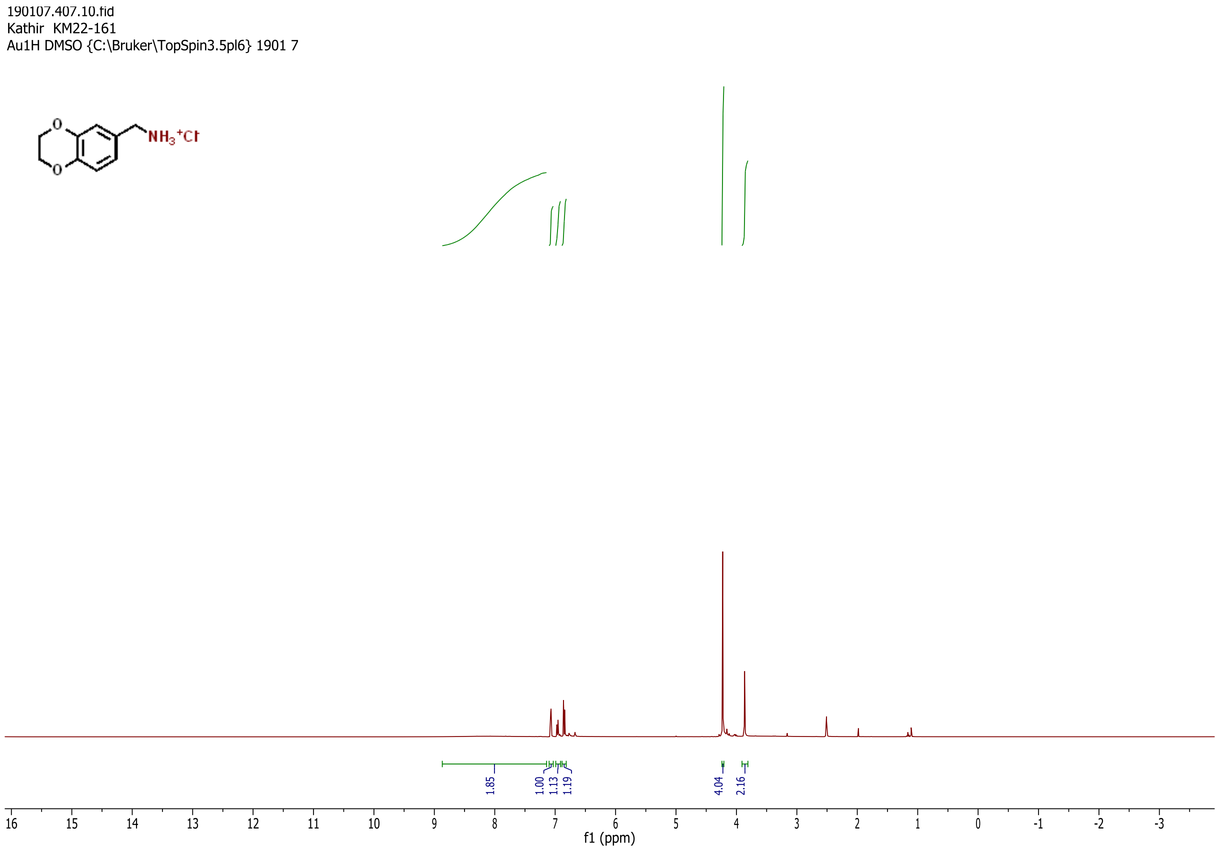

Supplementary Figure 46. ^{1}H NMR (**400 MHz, DMSO-d_6**)

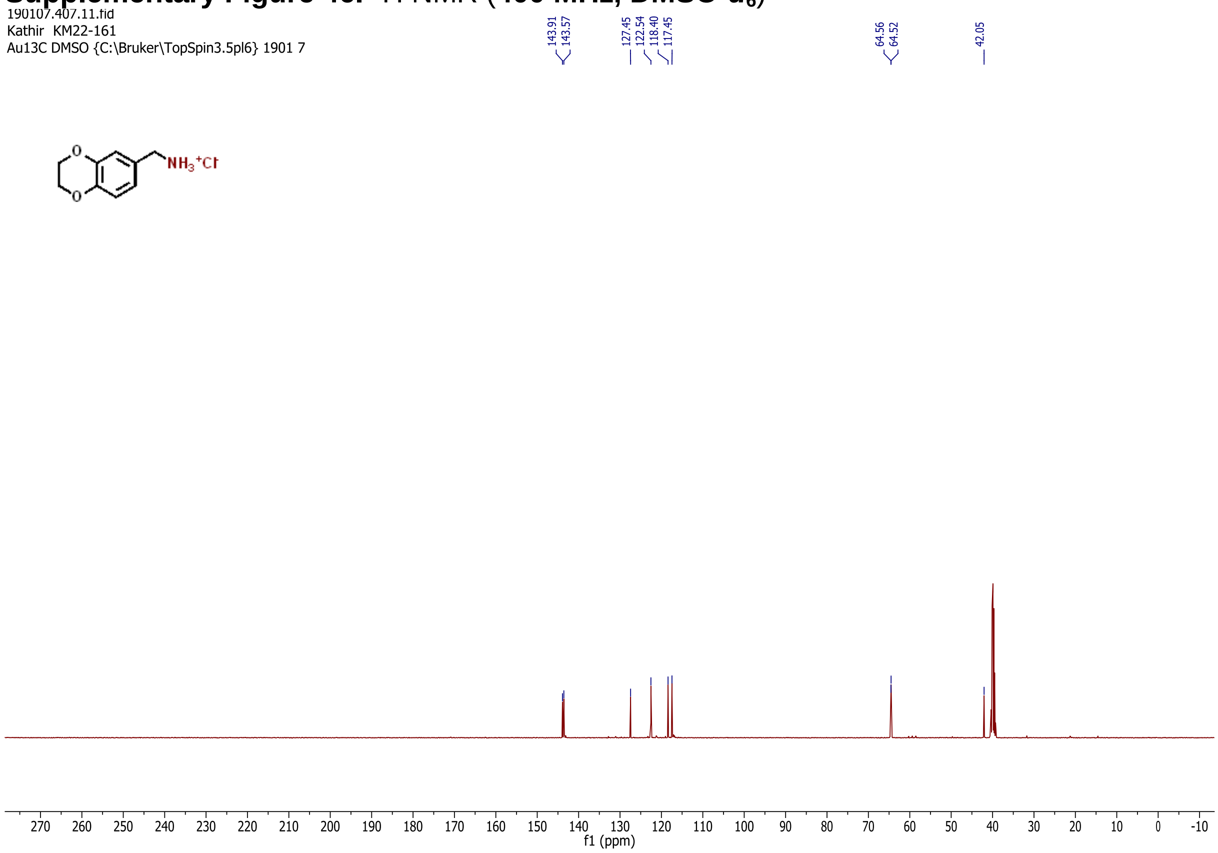

Supplementary Figure 47. ^{13}C NMR (**101 MHz, DMSO-d_6**)

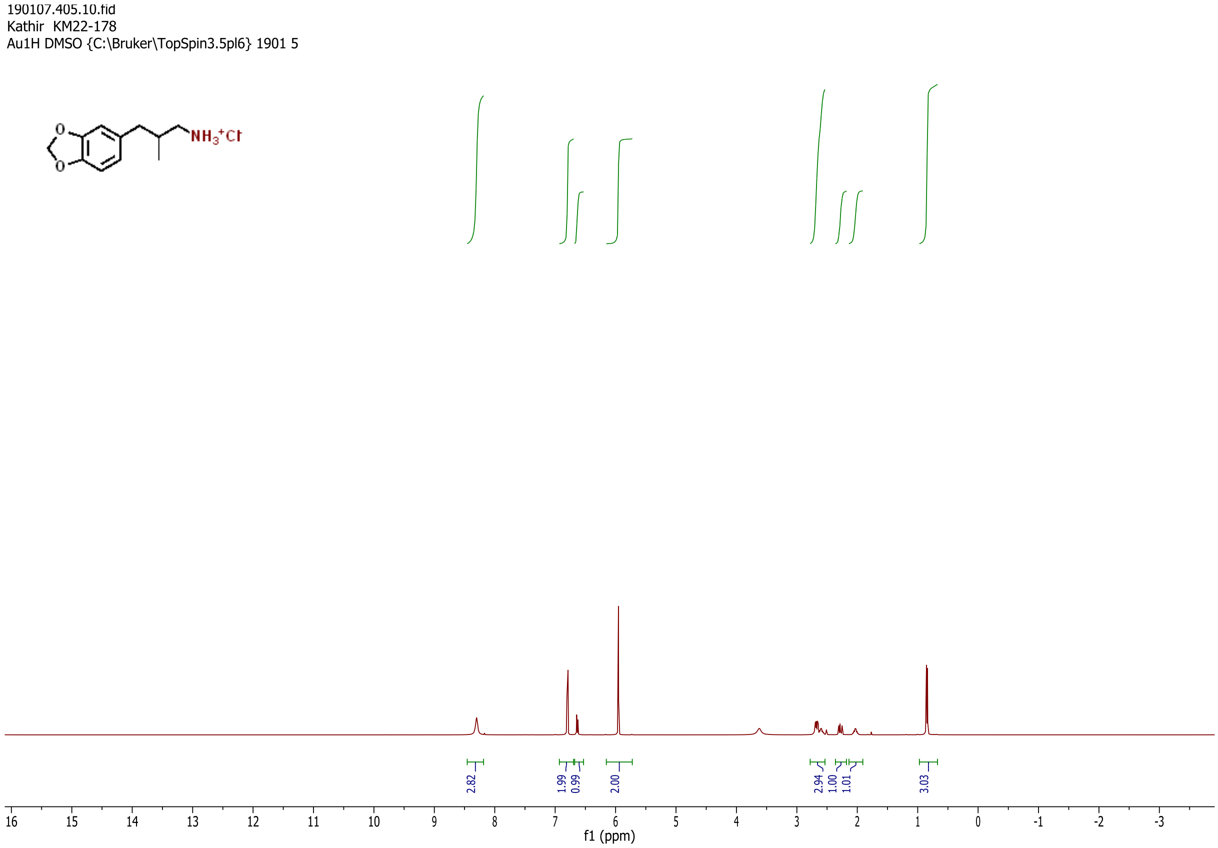

Supplementary Figure 48. ^{1}H NMR (**400 MHz, DMSO-d_6**)

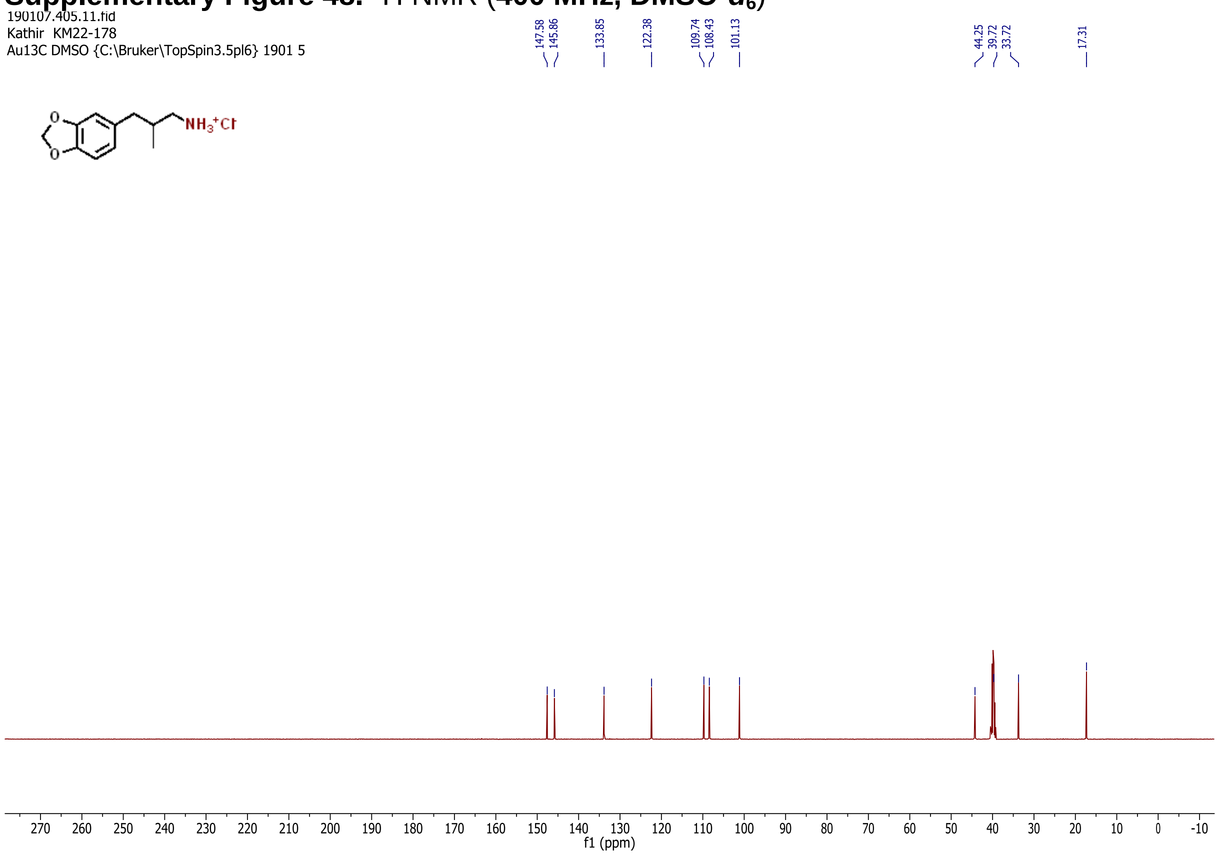

Supplementary Figure 49. ^{13}C NMR (**101 MHz, DMSO-d_6**)

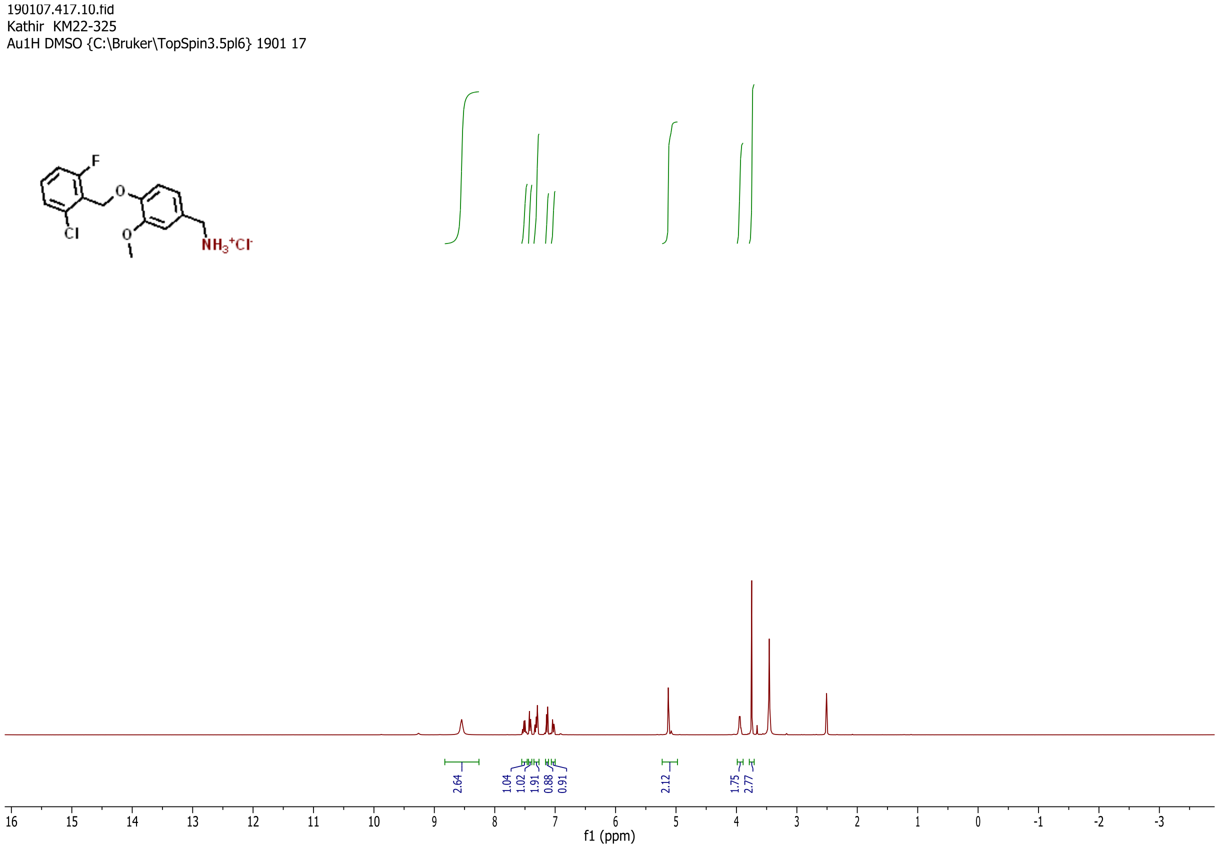

Supplementary Figure 50. ^{1}H NMR (**400 MHz, DMSO-*d*$_6$**)

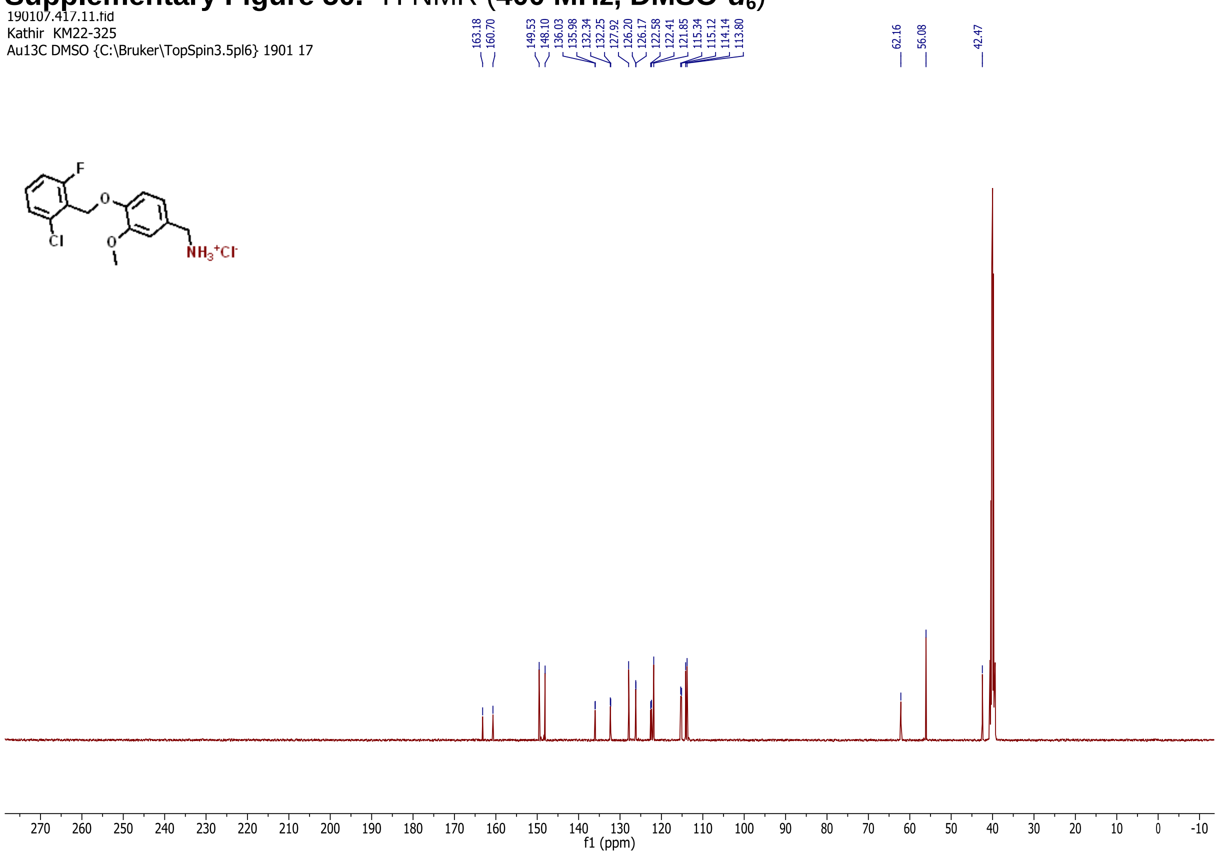

Supplementary Figure 51. ^{13}C NMR (**101 MHz, DMSO-*d*$_6$**)

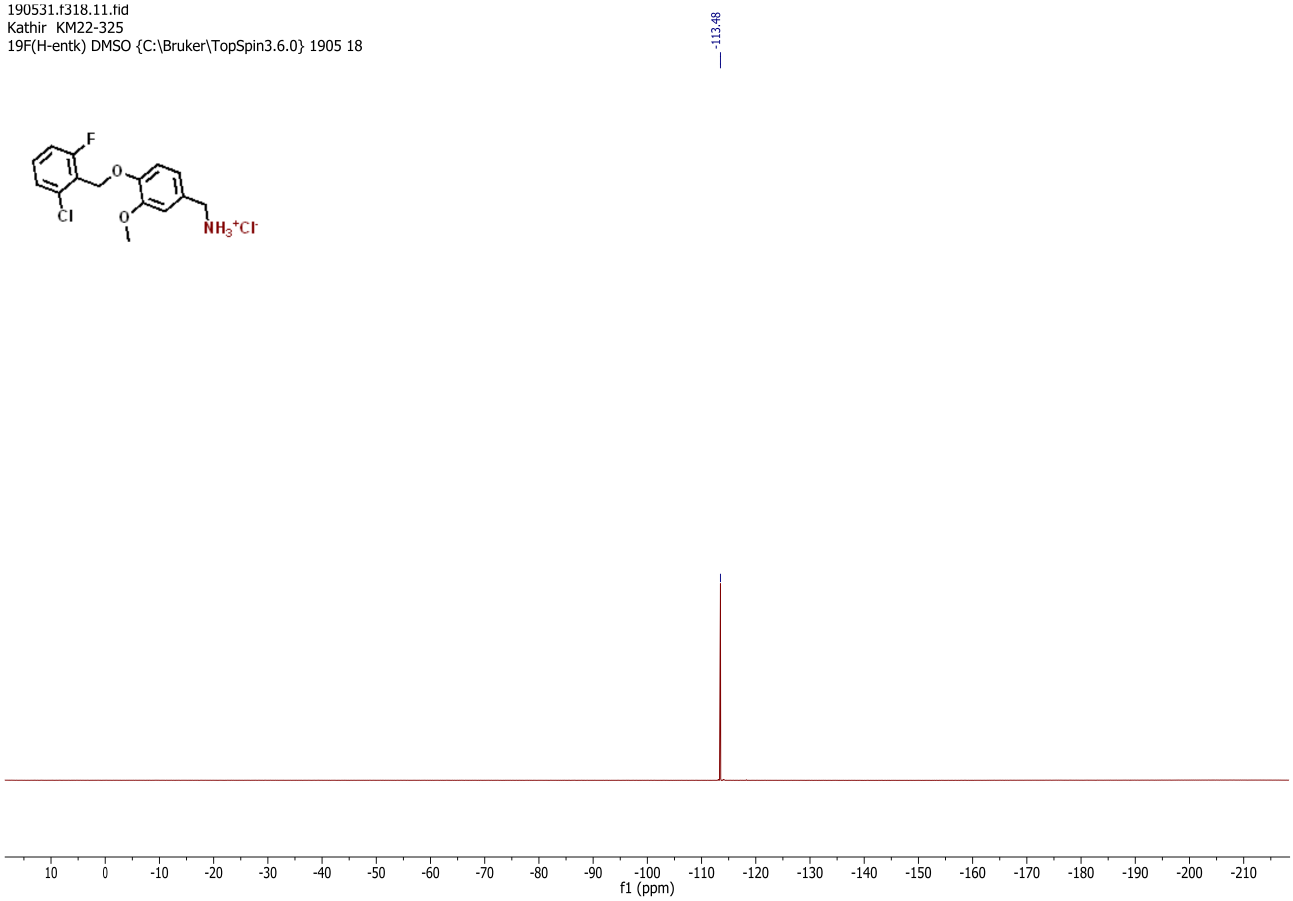

Supplementary Figure 52. ^{19}F NMR **(282 MHz, DMSO-d_6)**

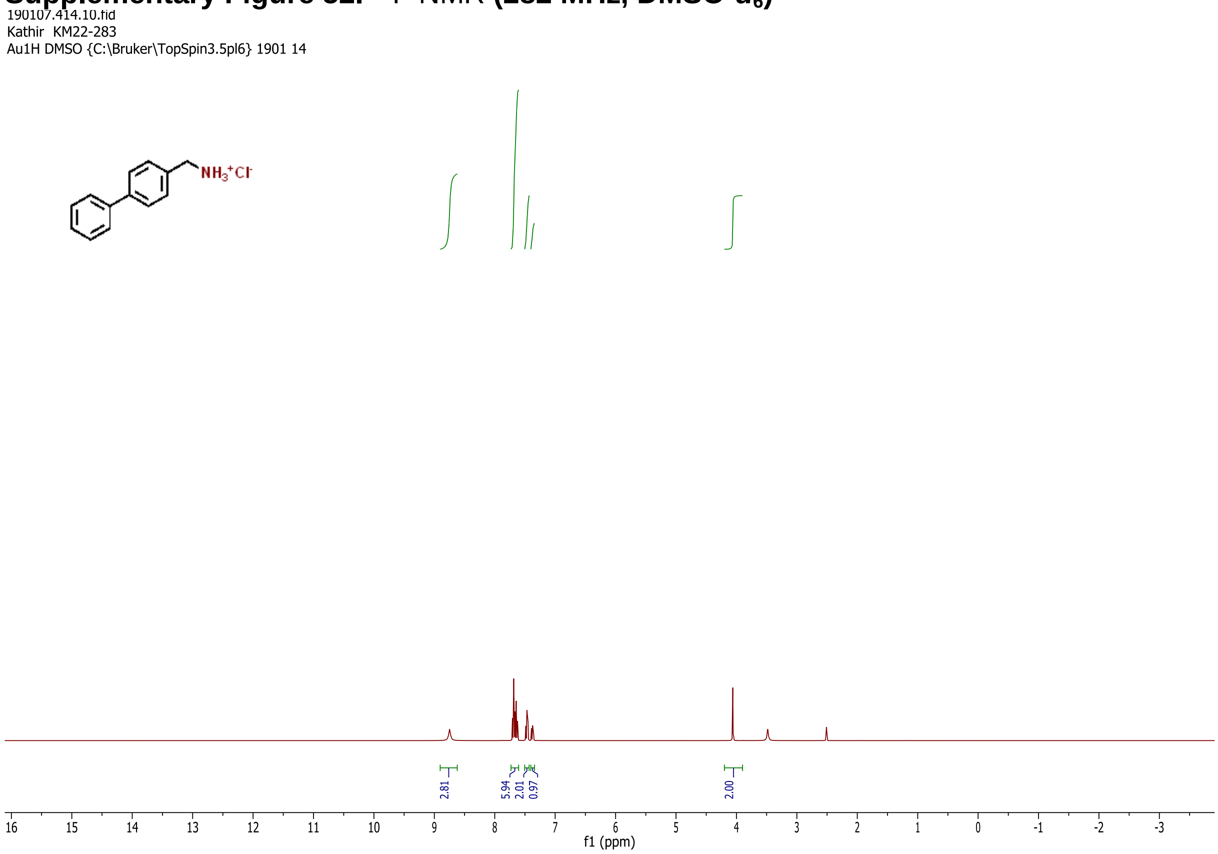

Supplementary Figure 53. ^{1}H NMR (**400 MHz, DMSO-d_6**)

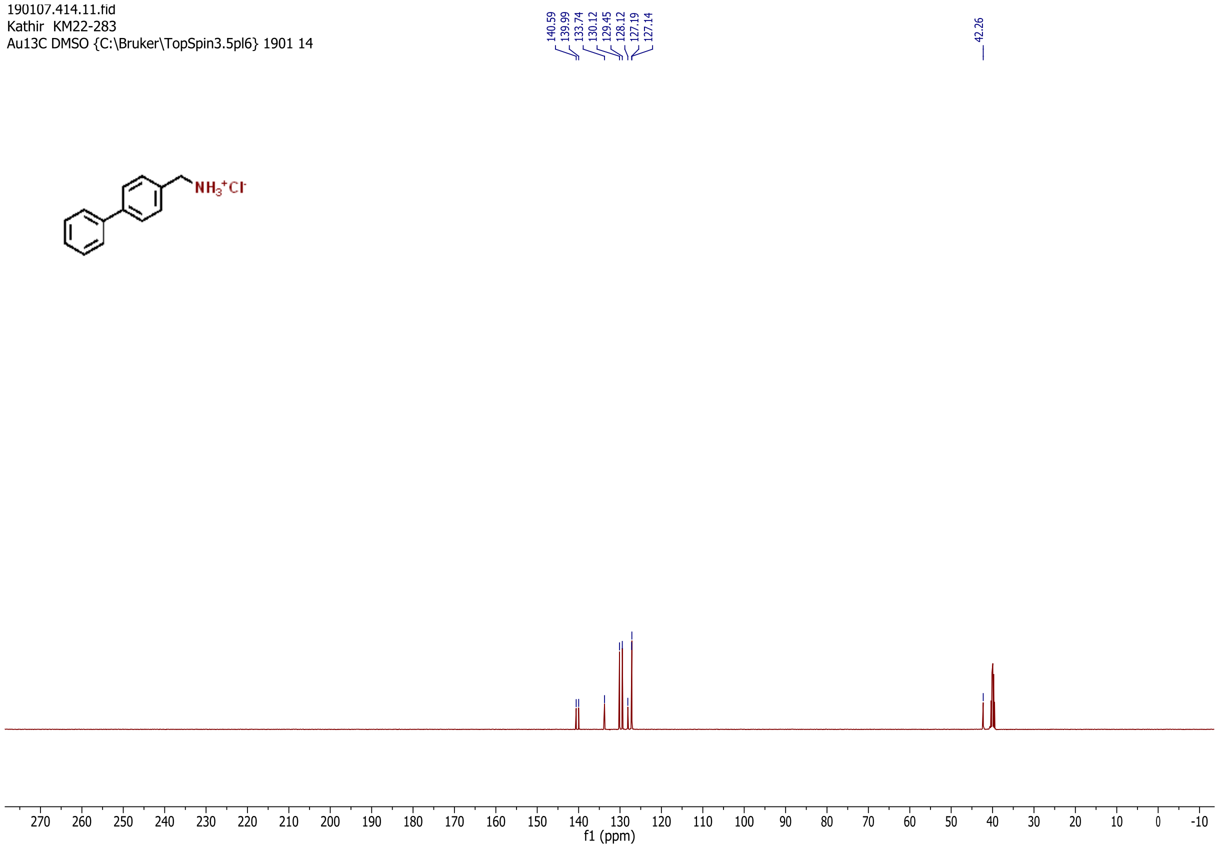

Supplementary Figure 54. ^{13}C NMR (**101 MHz, DMSO-*d*$_6$**)

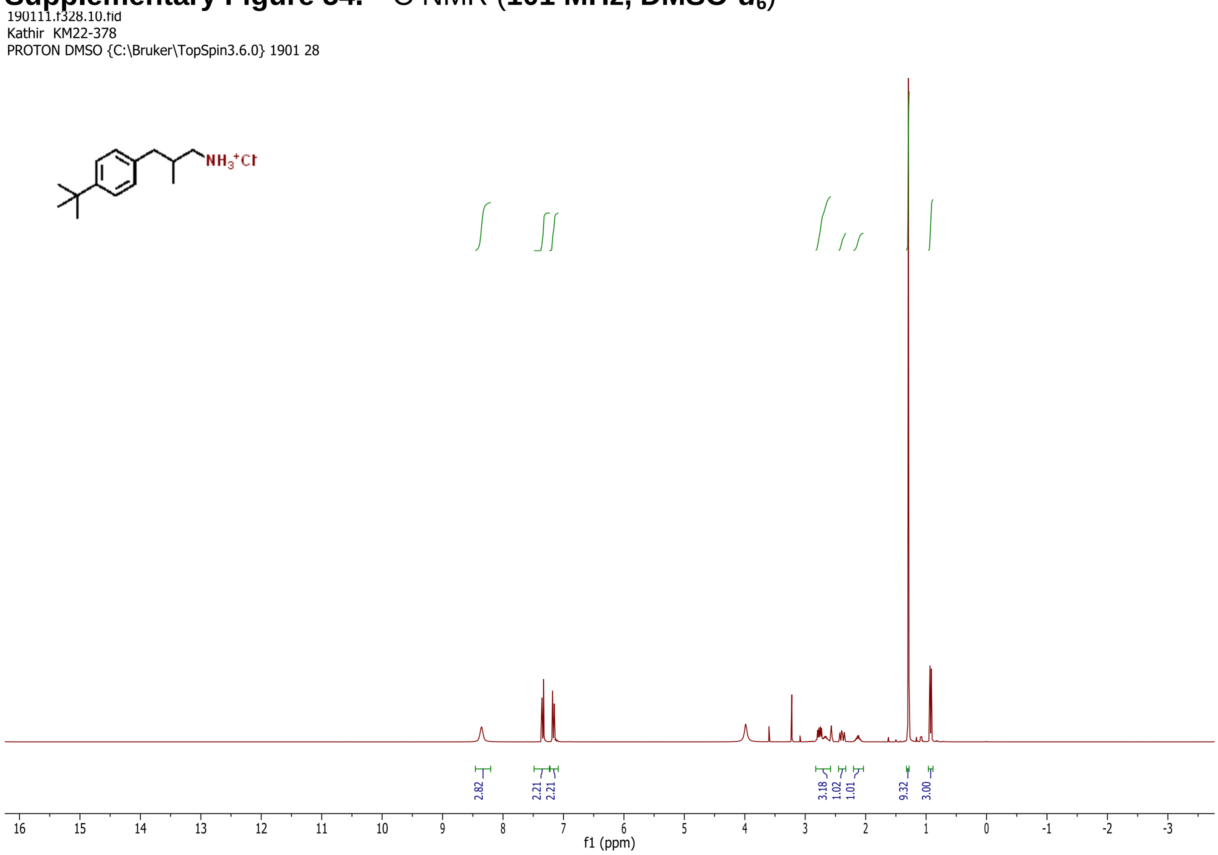

Supplementary Figure 55. ^{1}H NMR (**300 MHz, DMSO-*d*$_6$**)

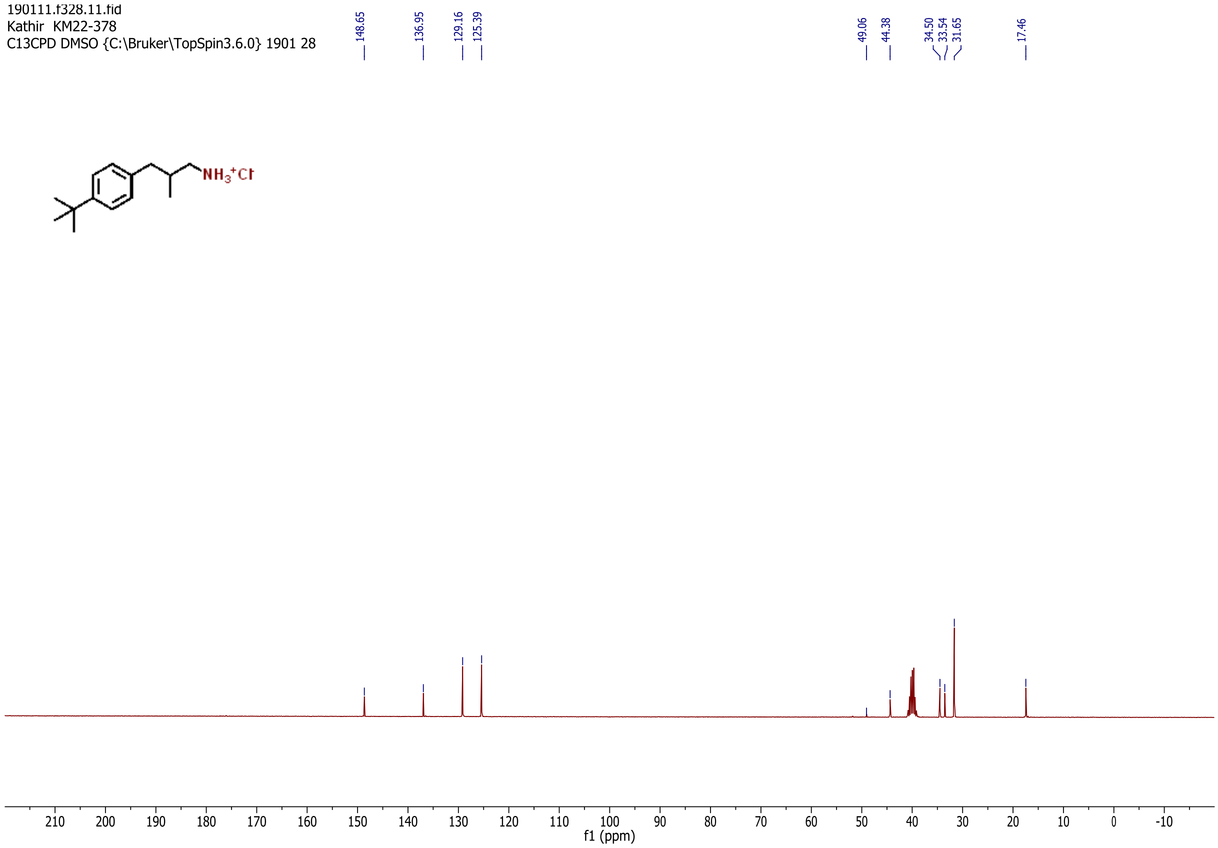

Supplementary Figure 56. ^{13}C NMR (**75 MHz, DMSO-*d*$_6$**)

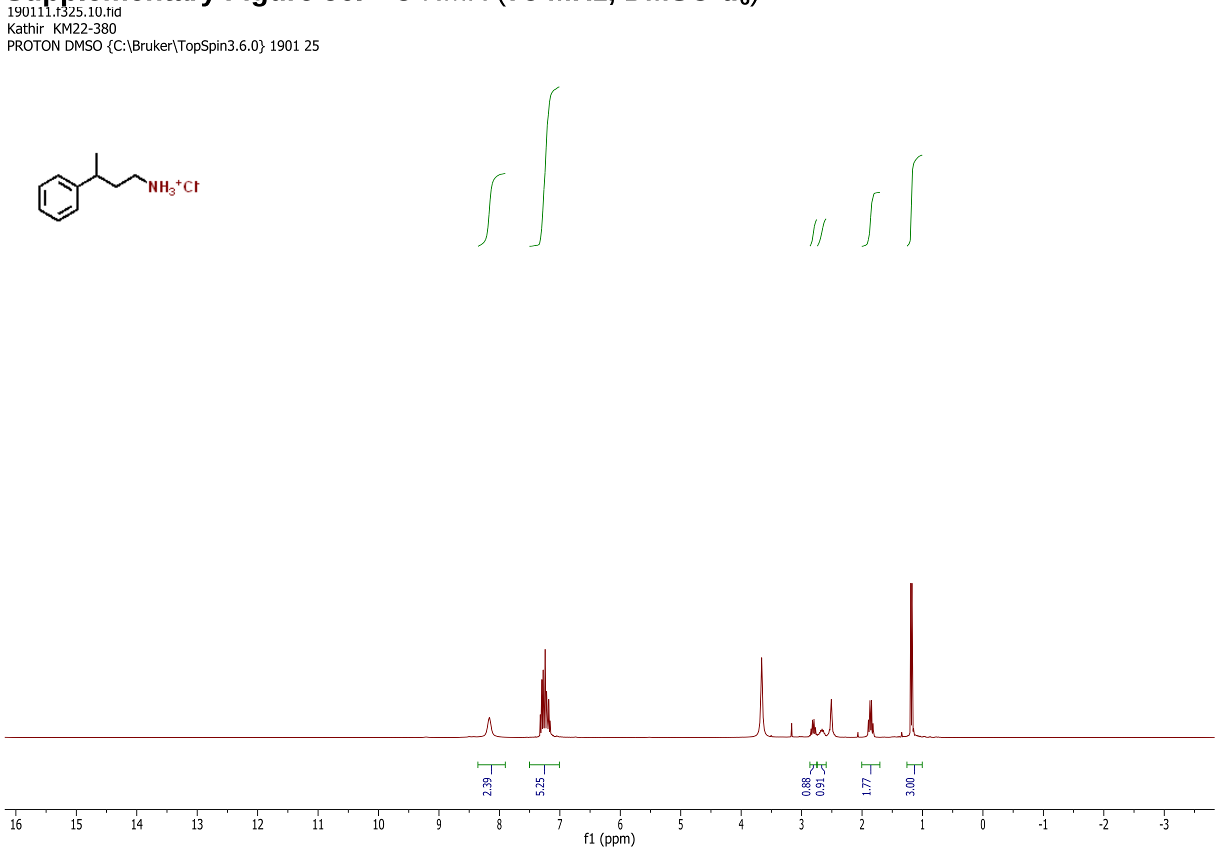

Supplementary Figure 57. ^{1}H NMR (**300 MHz, DMSO-*d*$_6$**)

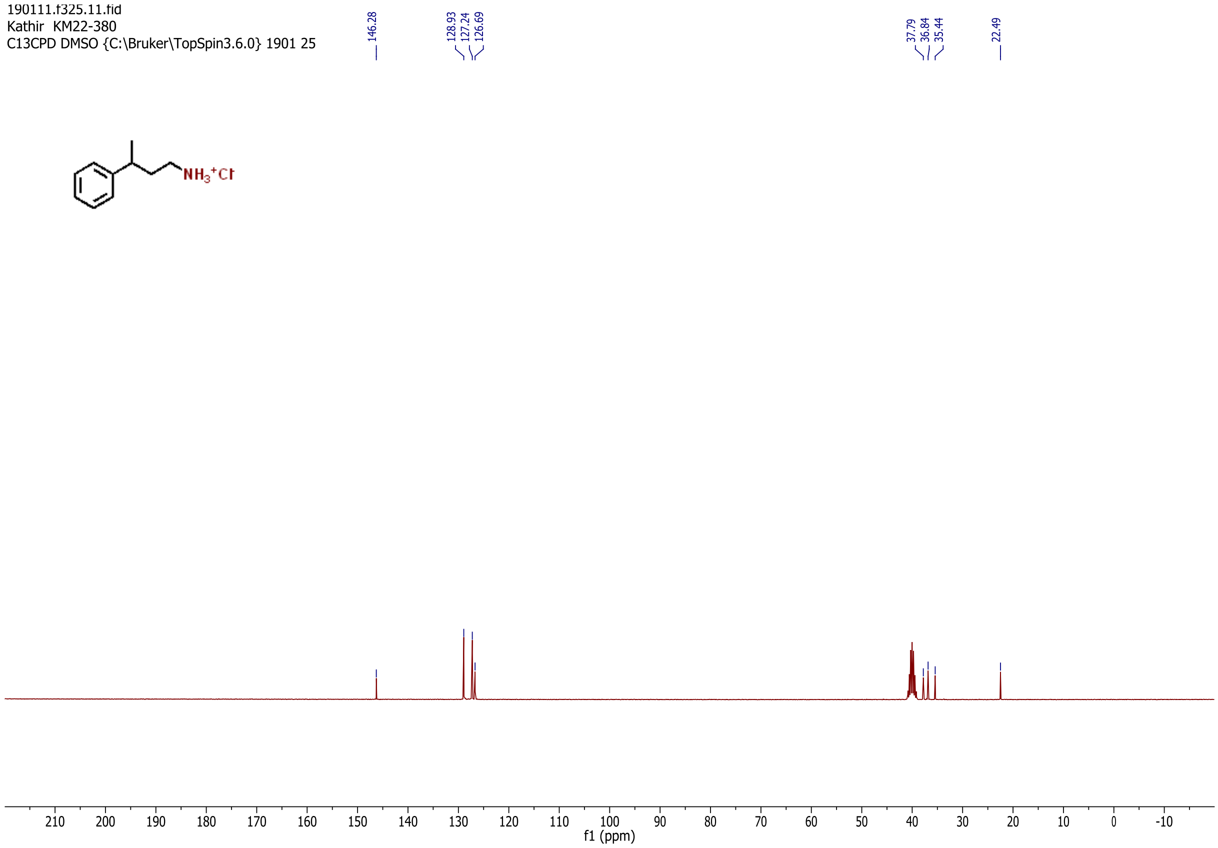

Supplementary Figure 58. ^{13}C NMR (**75 MHz, DMSO-*d*$_6$**)

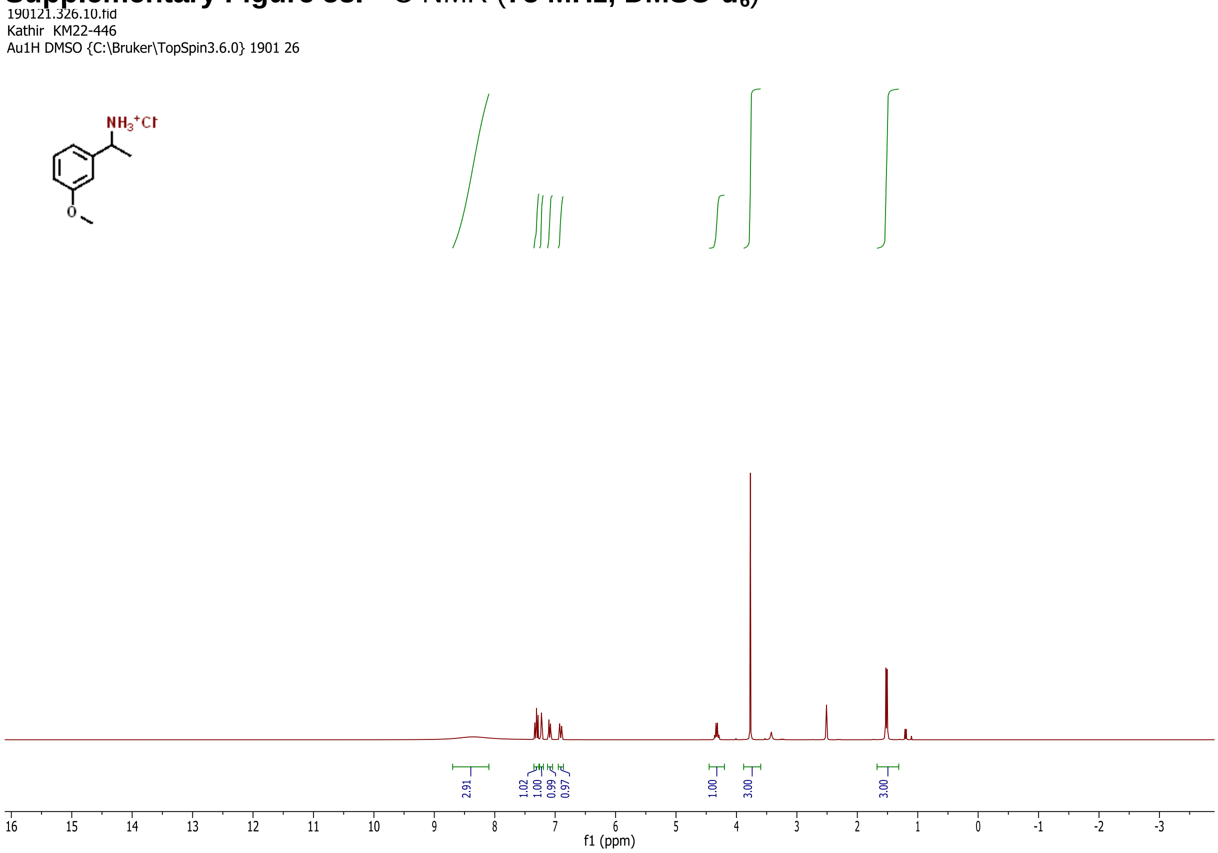

Supplementary Figure 59. ^{1}H NMR (**300 MHz, DMSO-*d*$_6$**)

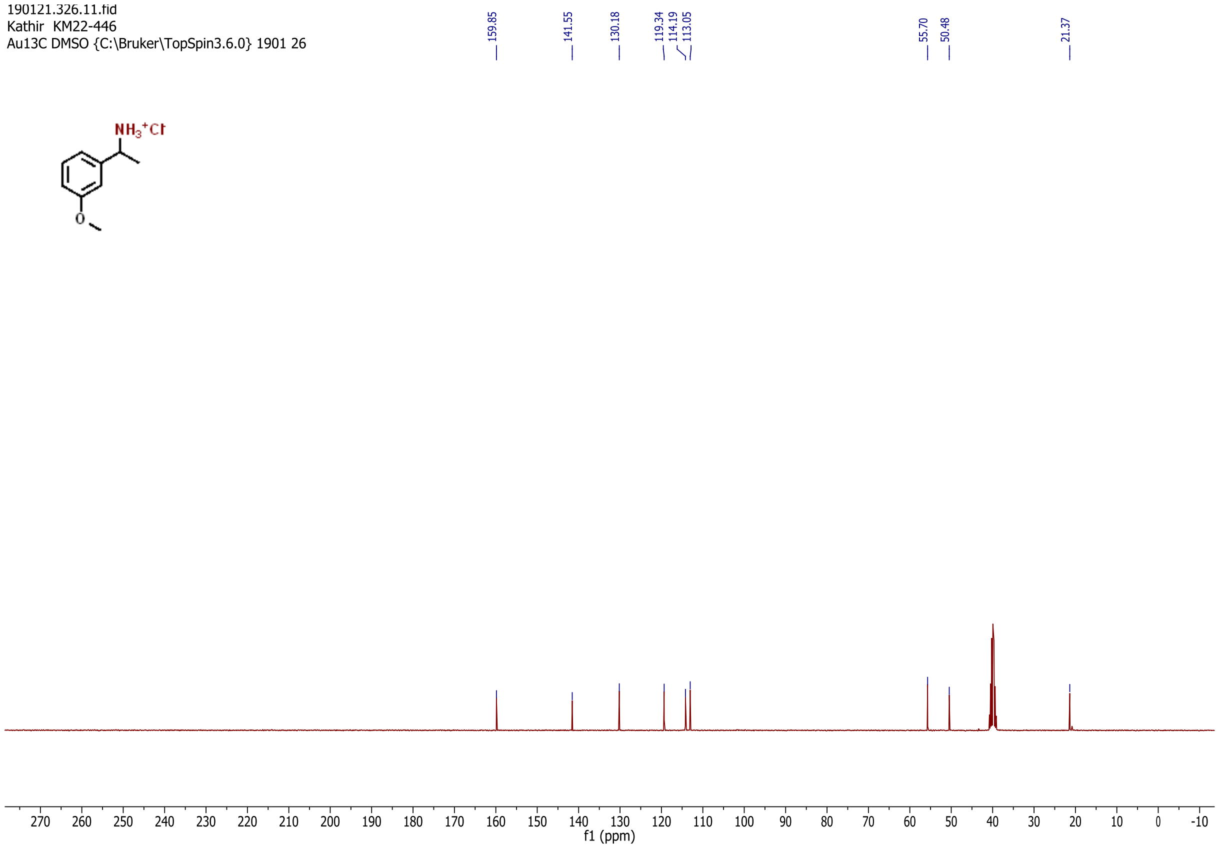

Supplementary Figure 60. ^{13}C NMR (**75 MHz, DMSO-*d*$_6$**)

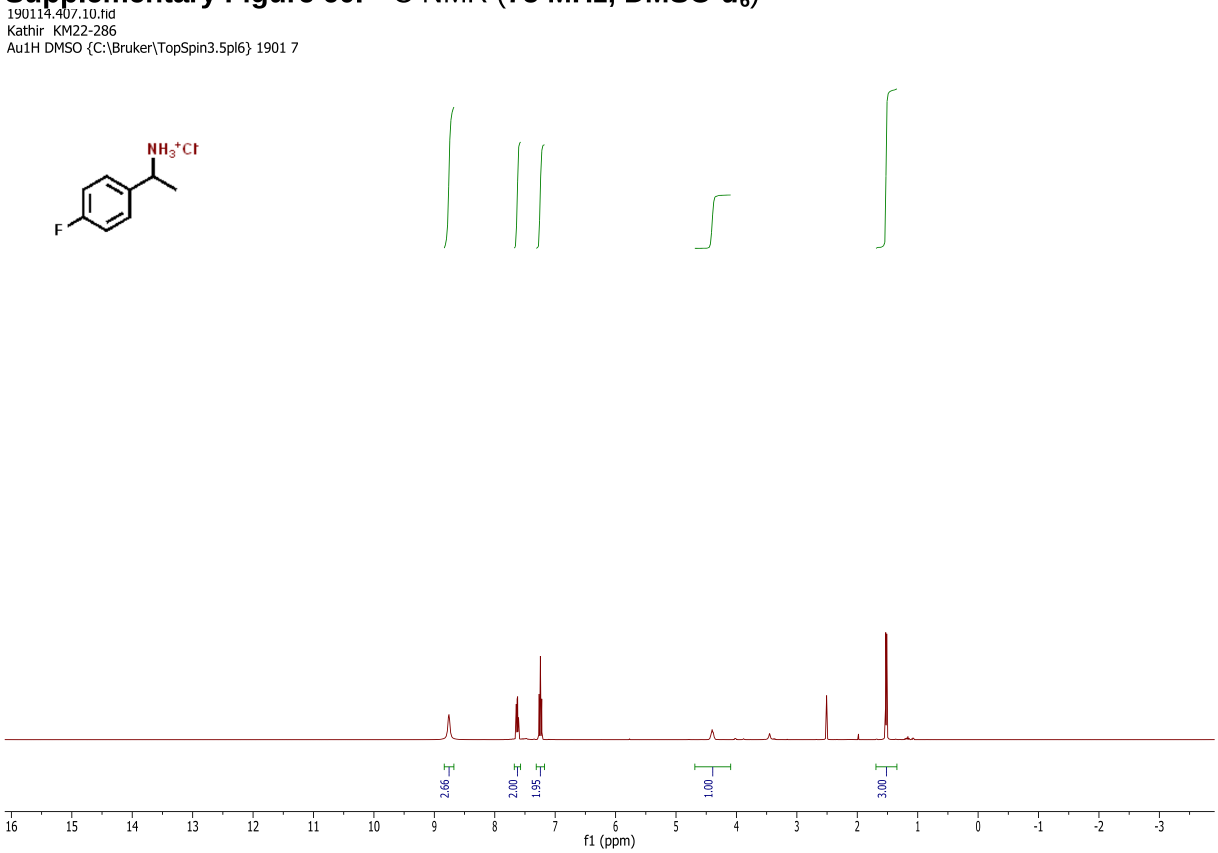

Supplementary Figure 61. ^{1}H NMR (**400 MHz, DMSO-*d*$_6$**)

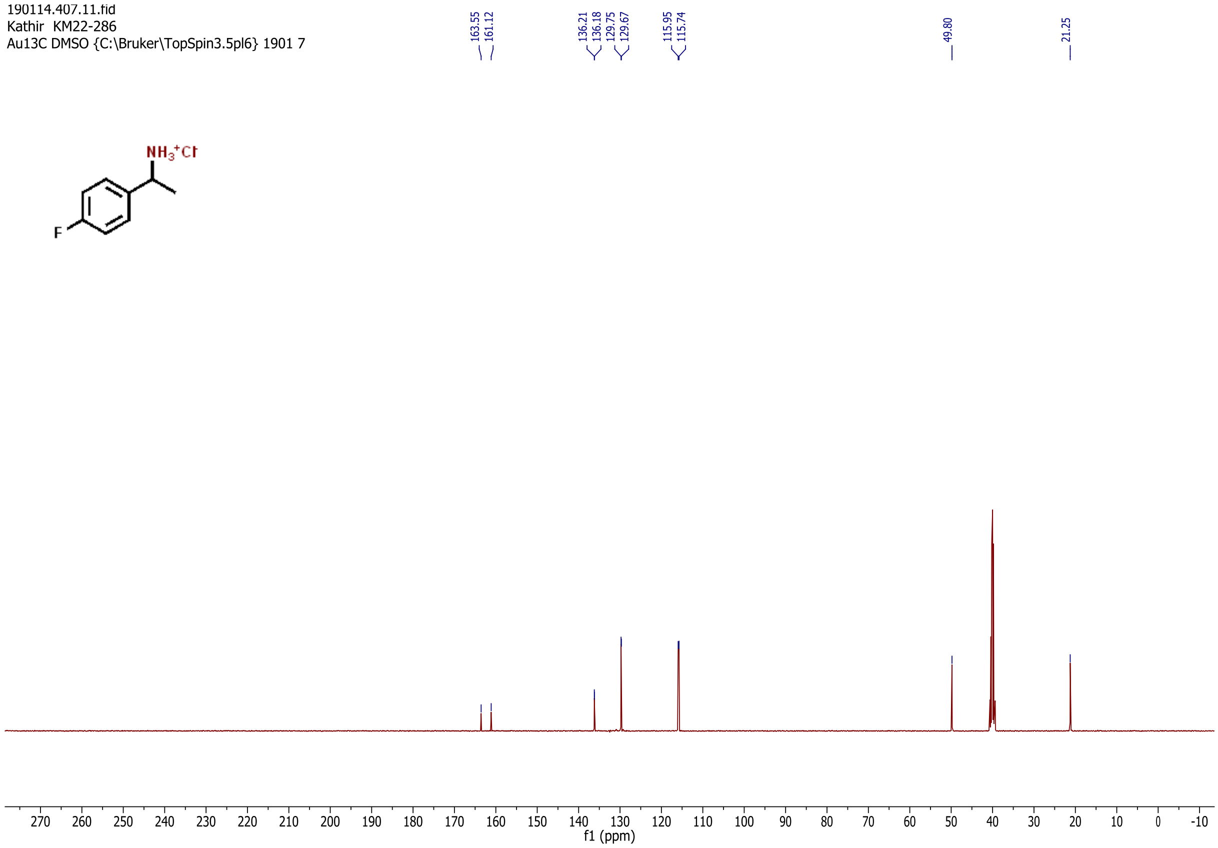

Supplementary Figure 62. ^{13}C NMR (**101 MHz, DMSO-*d*$_6$**)

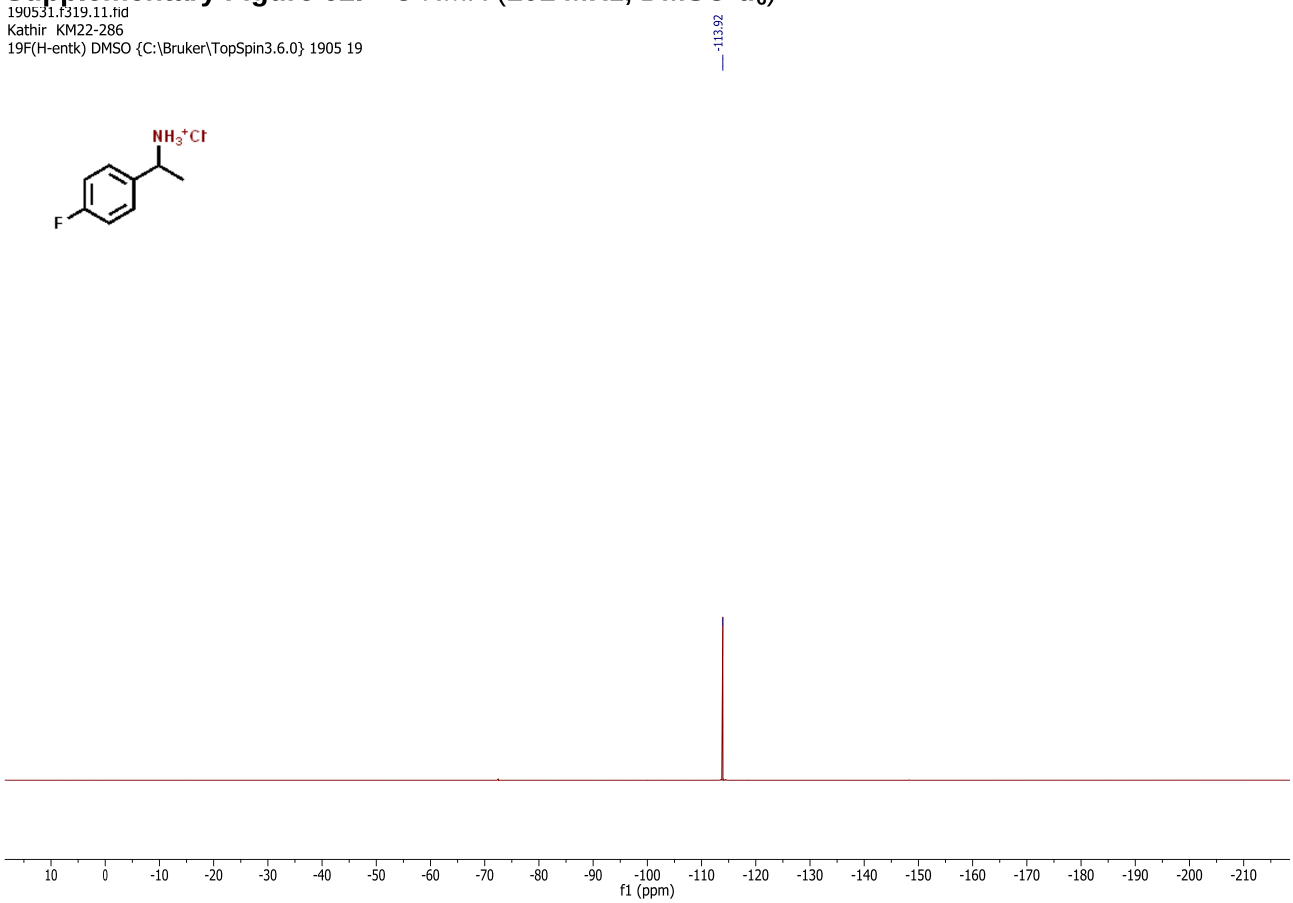

Supplementary Figure 63. ^{19}F NMR **(282 MHz, DMSO-*d*$_6$)**

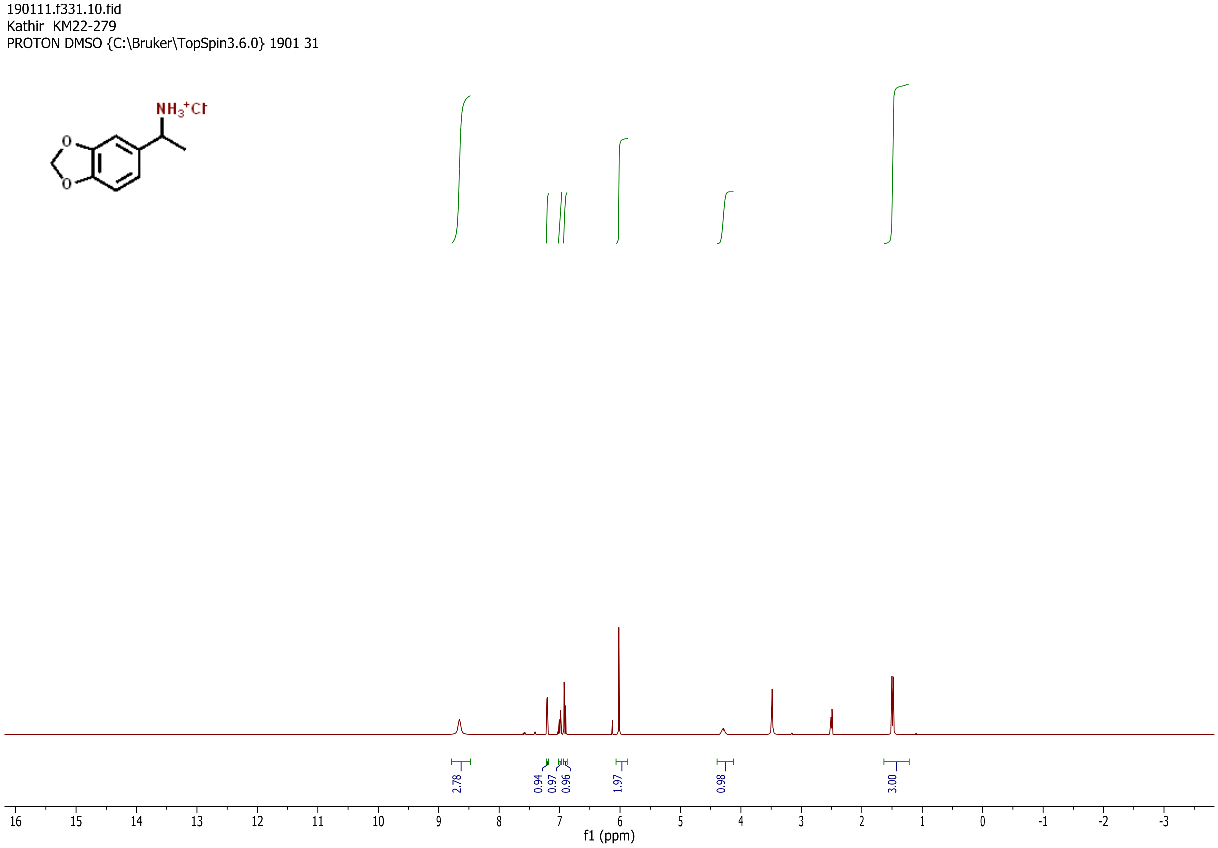

Supplementary Figure 64. ^{1}H NMR (**300 MHz, DMSO-*d*$_6$**)

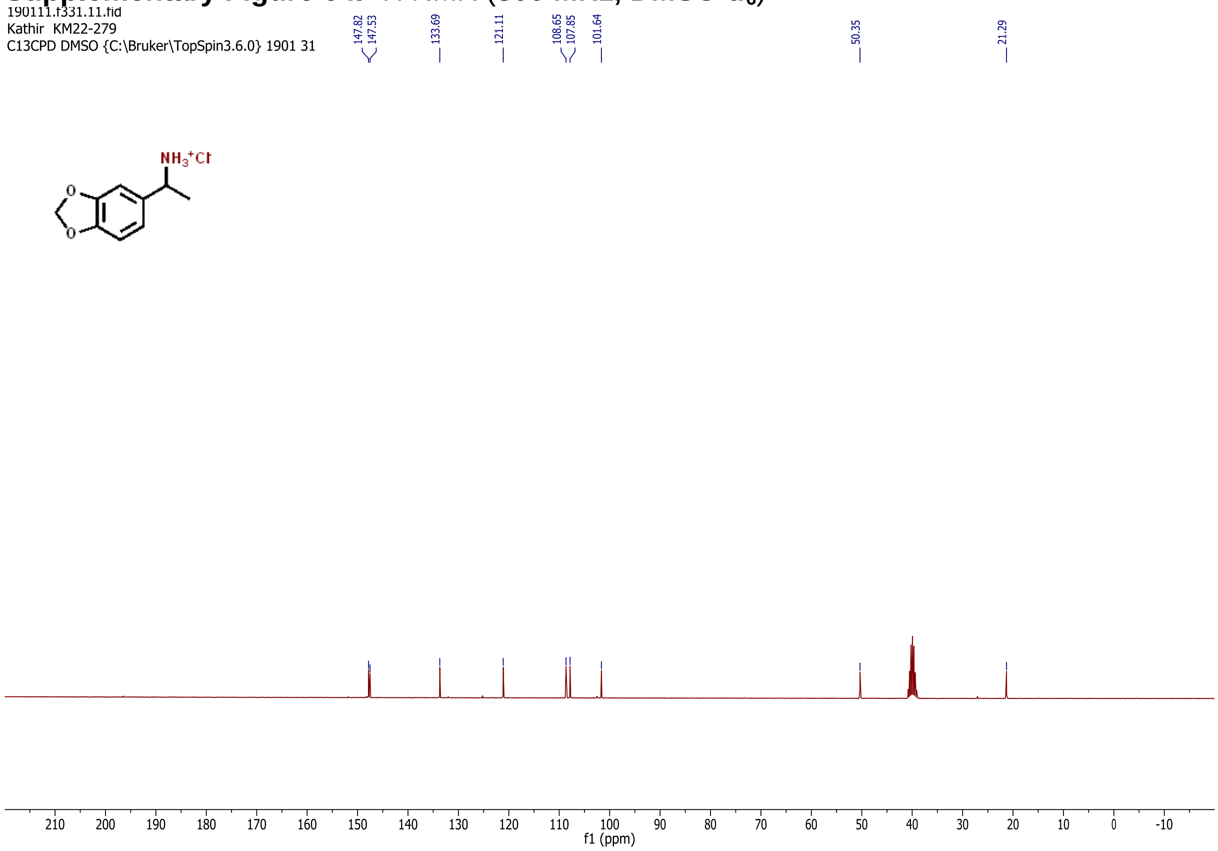

Supplementary Figure 65. ^{13}C NMR (**75 MHz, DMSO-*d*$_6$**)

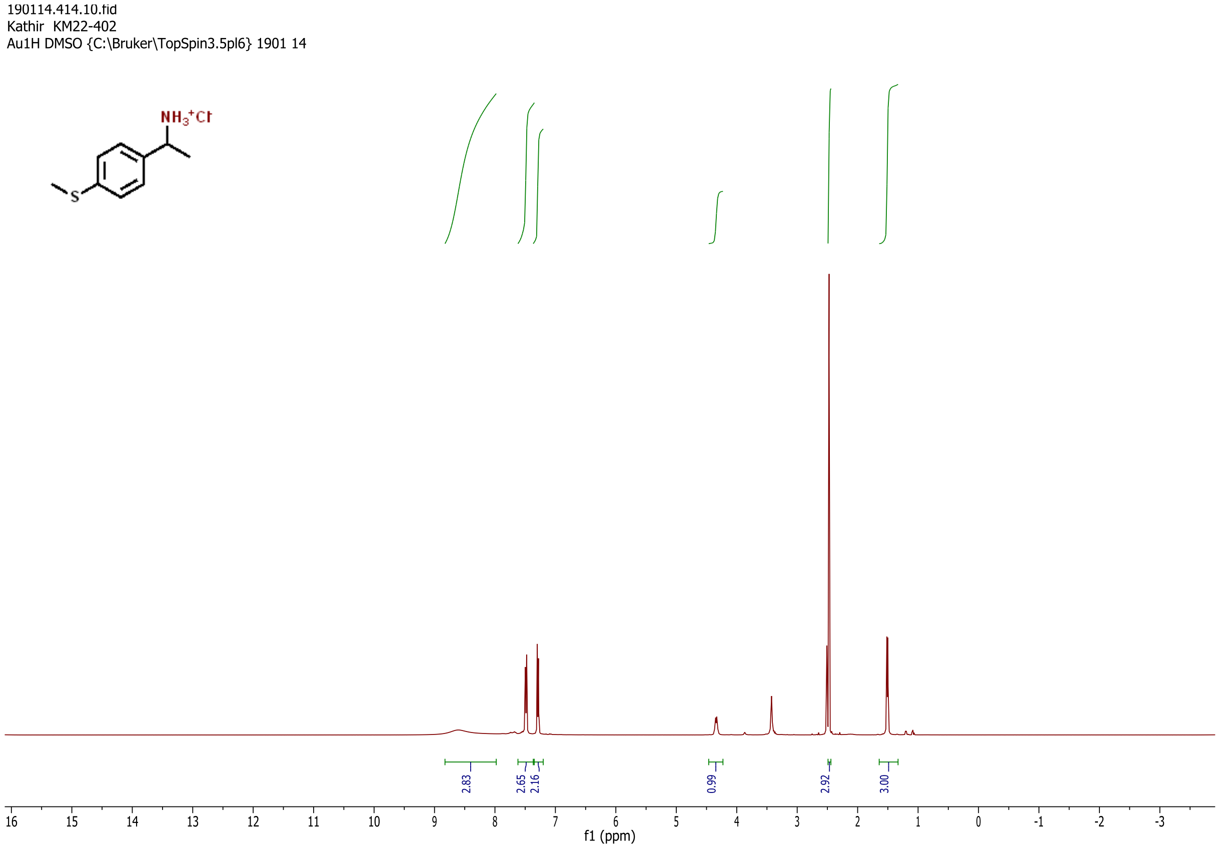

Supplementary Figure 66. ^{1}H NMR (**400 MHz, DMSO-*d*$_{6}$**)

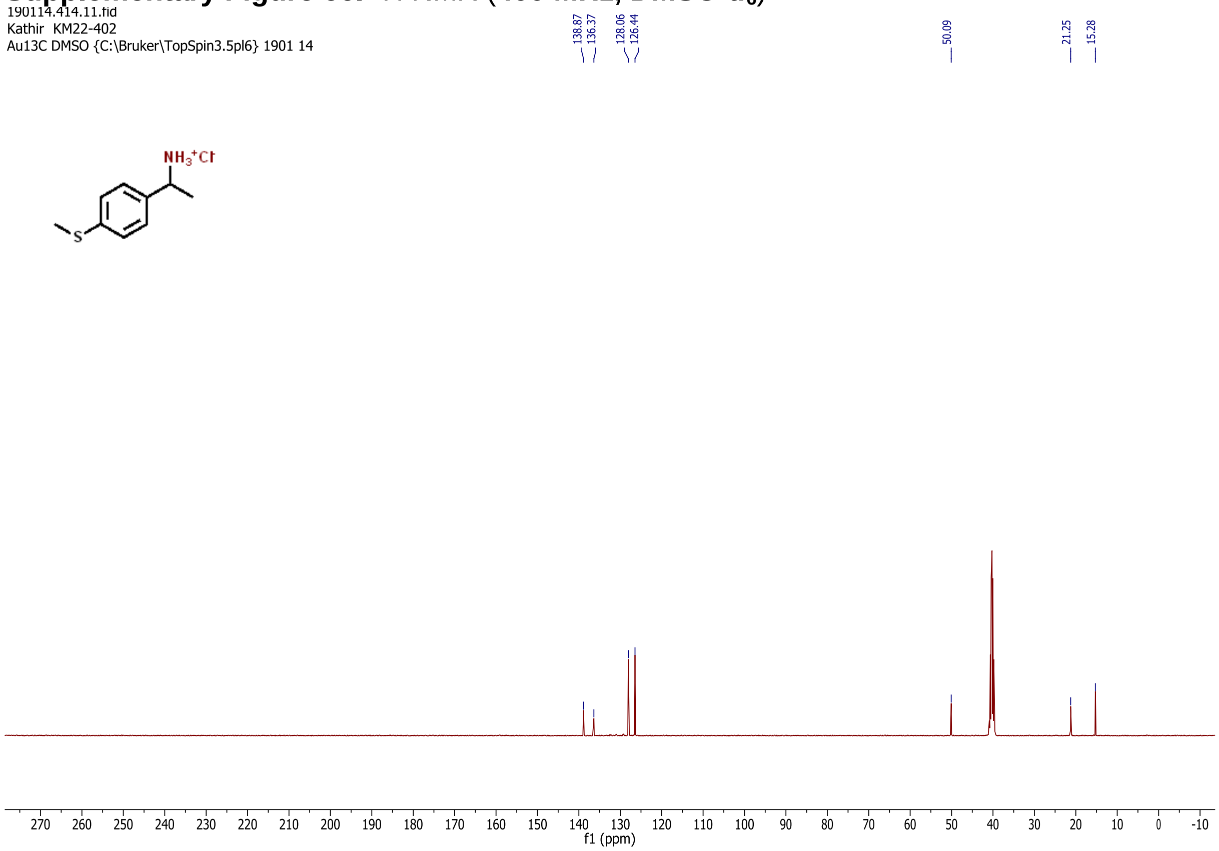

Supplementary Figure 67. ^{13}C NMR (**101 MHz, DMSO-*d*$_{6}$**)

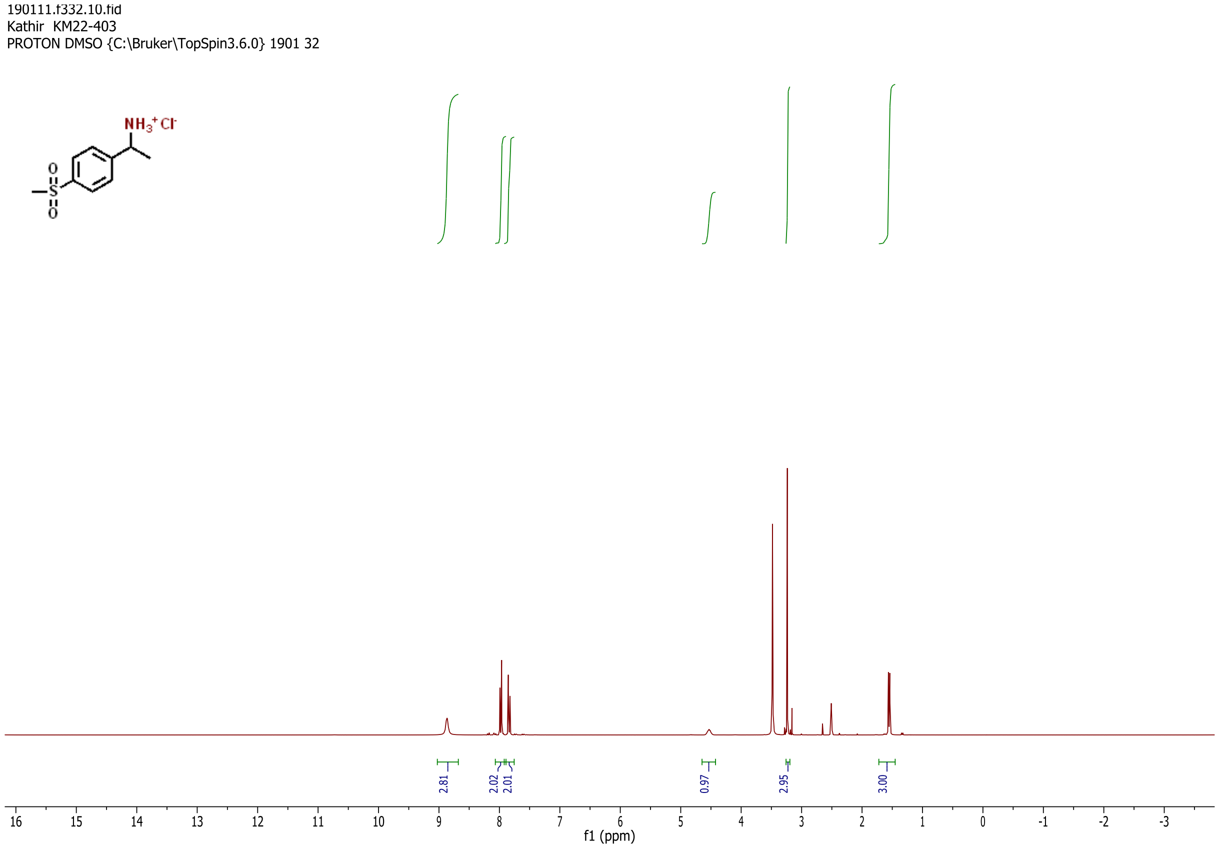

Supplementary Figure 68. ^{1}H NMR (**300 MHz, DMSO-*d*$_6$**)

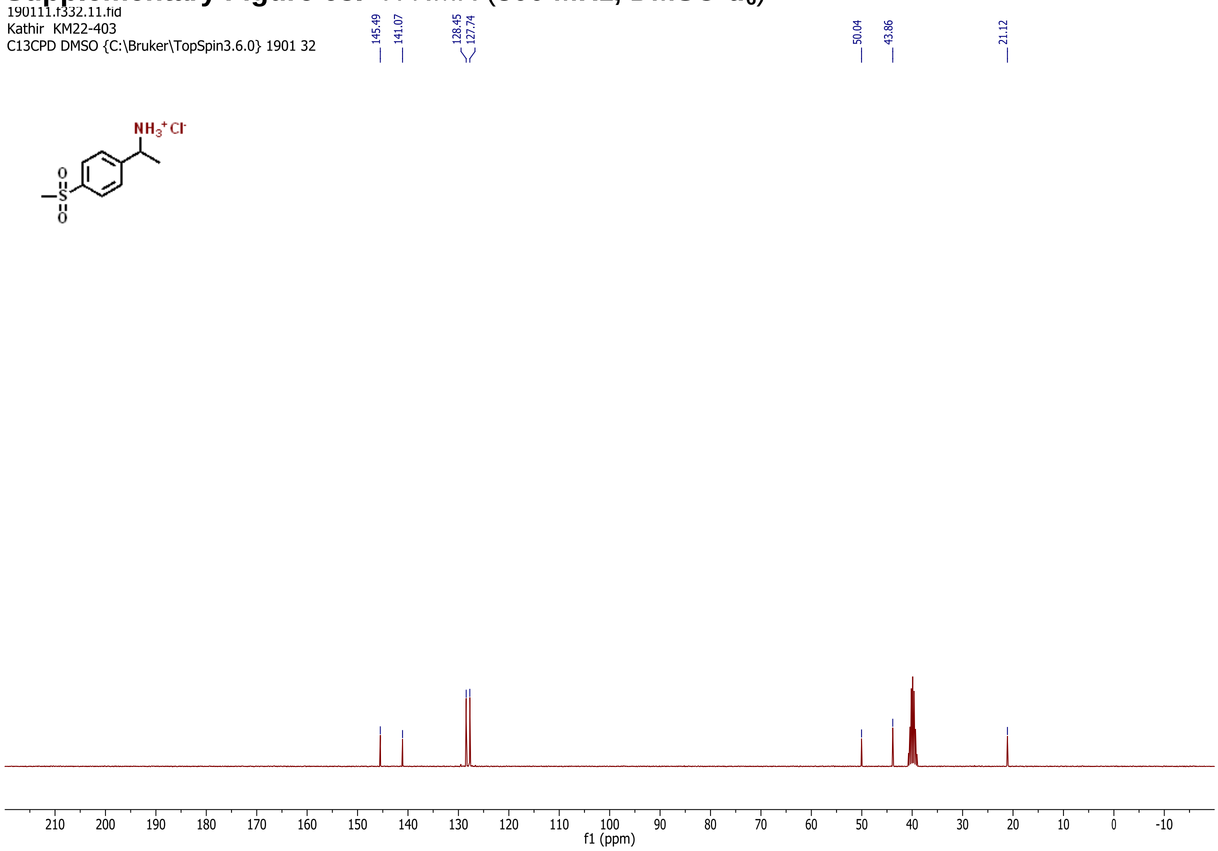

Supplementary Figure 69. ^{13}C NMR (**75 MHz, DMSO-*d*$_6$**)

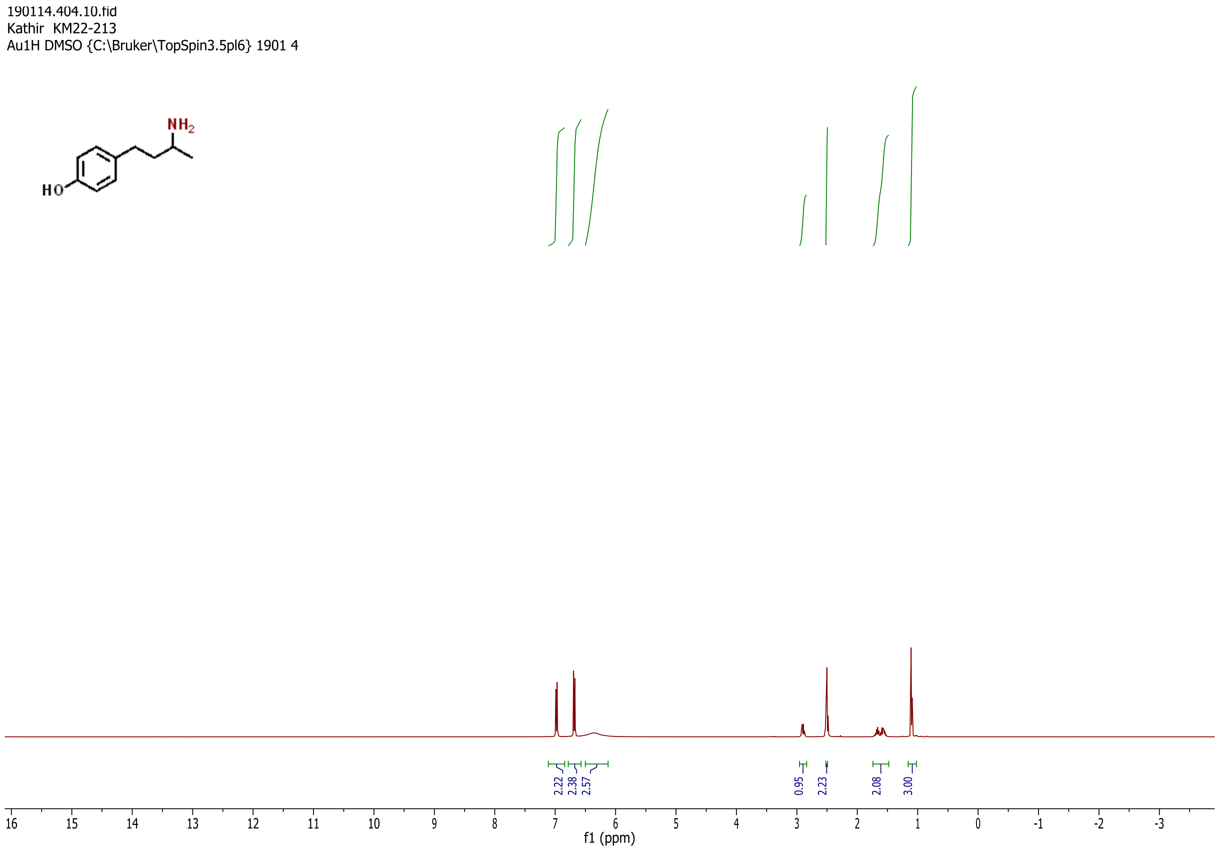

Supplementary Figure 70. ^{1}H NMR (**400 MHz, DMSO-d_6**)

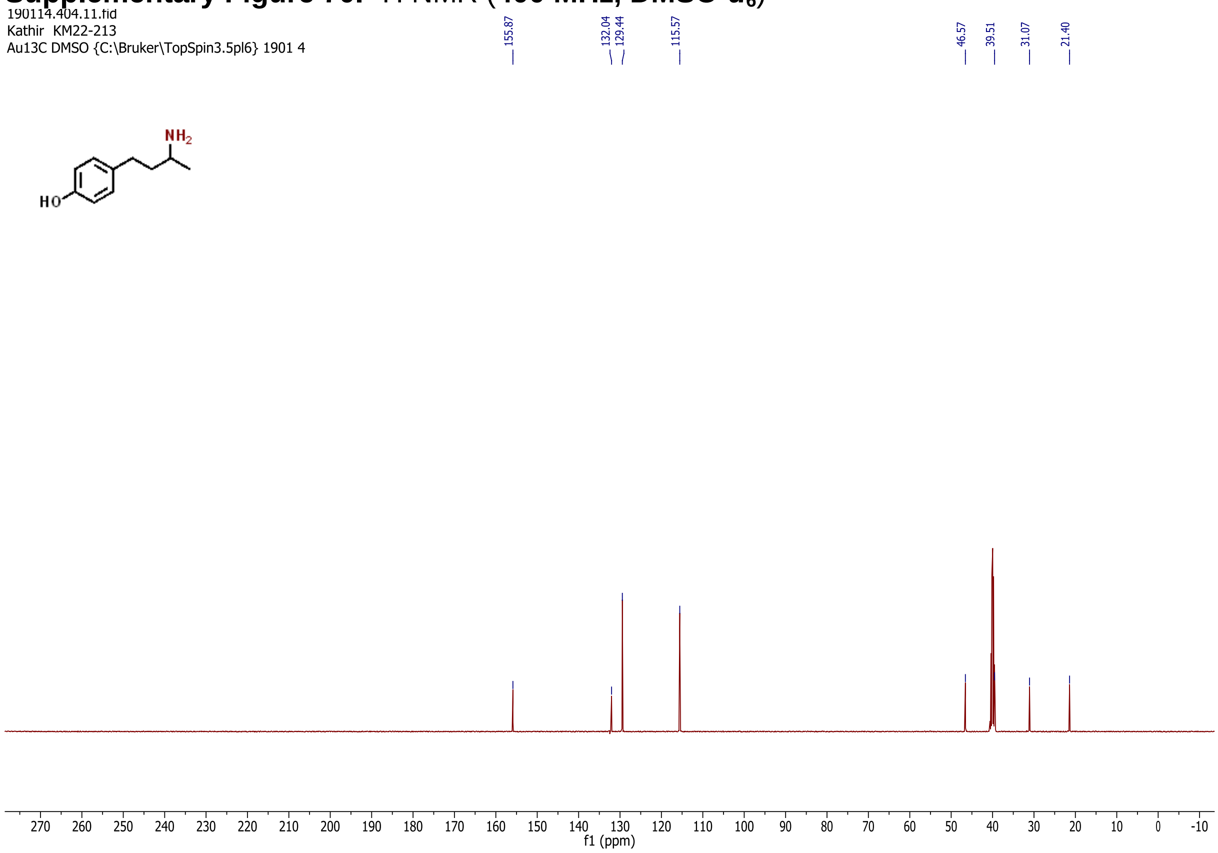

Supplementary Figure 71. ^{13}C NMR (**101 MHz, DMSO-d_6**)

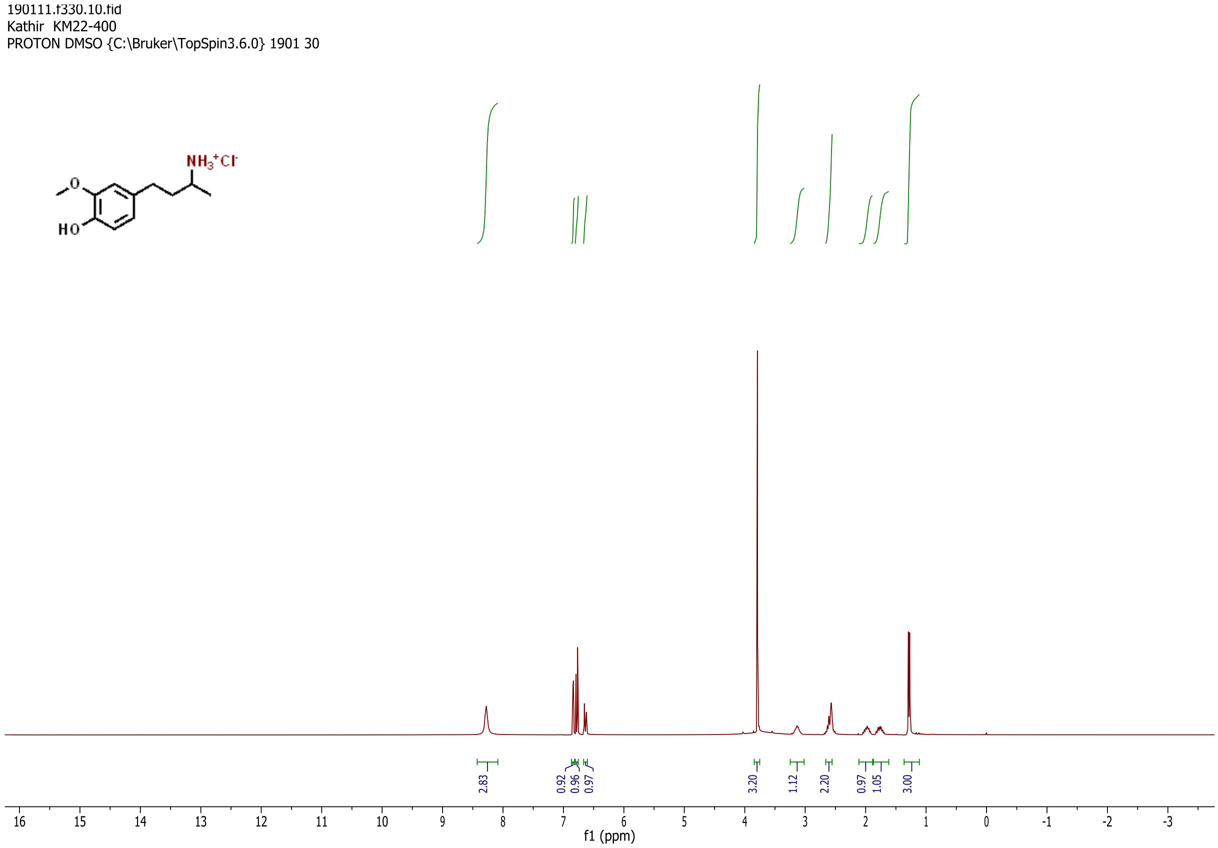

Supplementary Figure 72. ^{1}H NMR (**300 MHz, DMSO-*d*$_6$**)

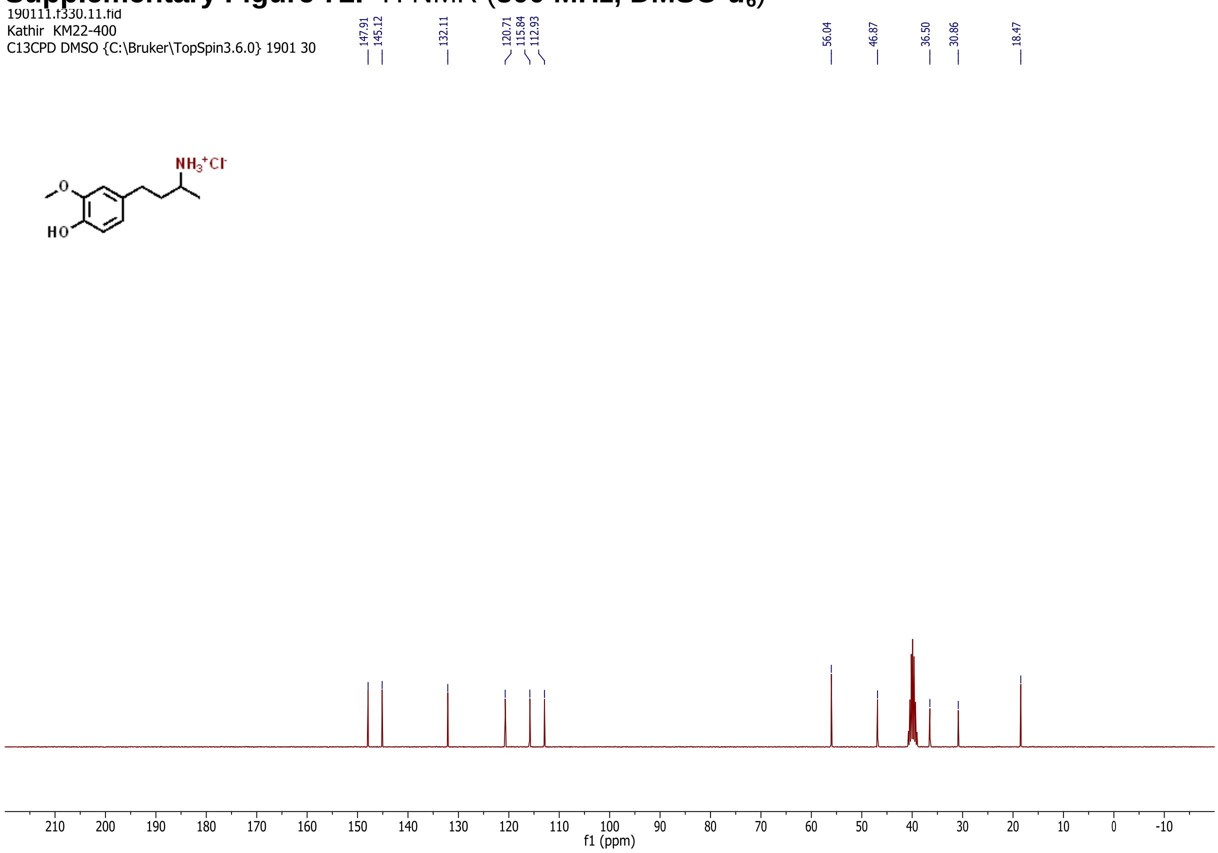

Supplementary Figure 73. ^{13}C NMR (**75 MHz, DMSO-*d*$_6$**)

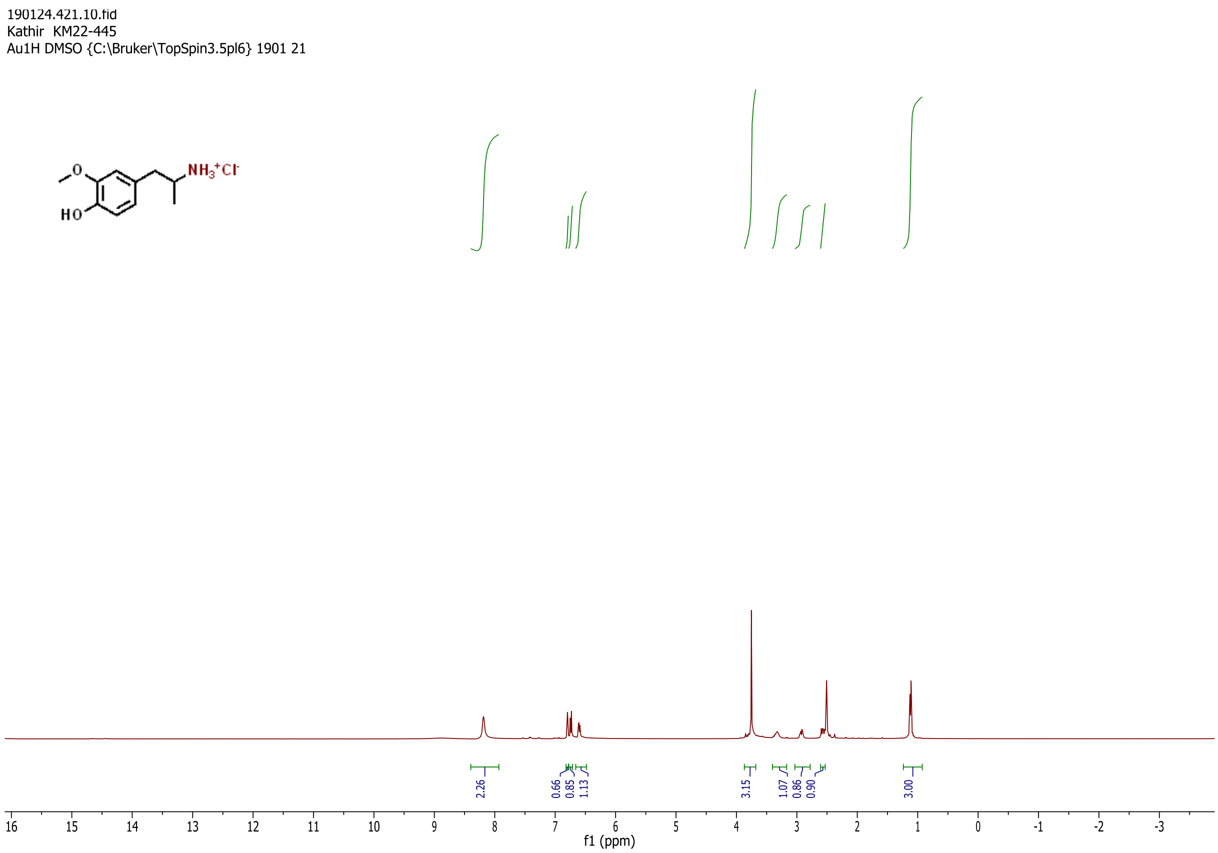

Supplementary Figure 74. ^{1}H NMR (**400 MHz, DMSO-*d*$_6$**)

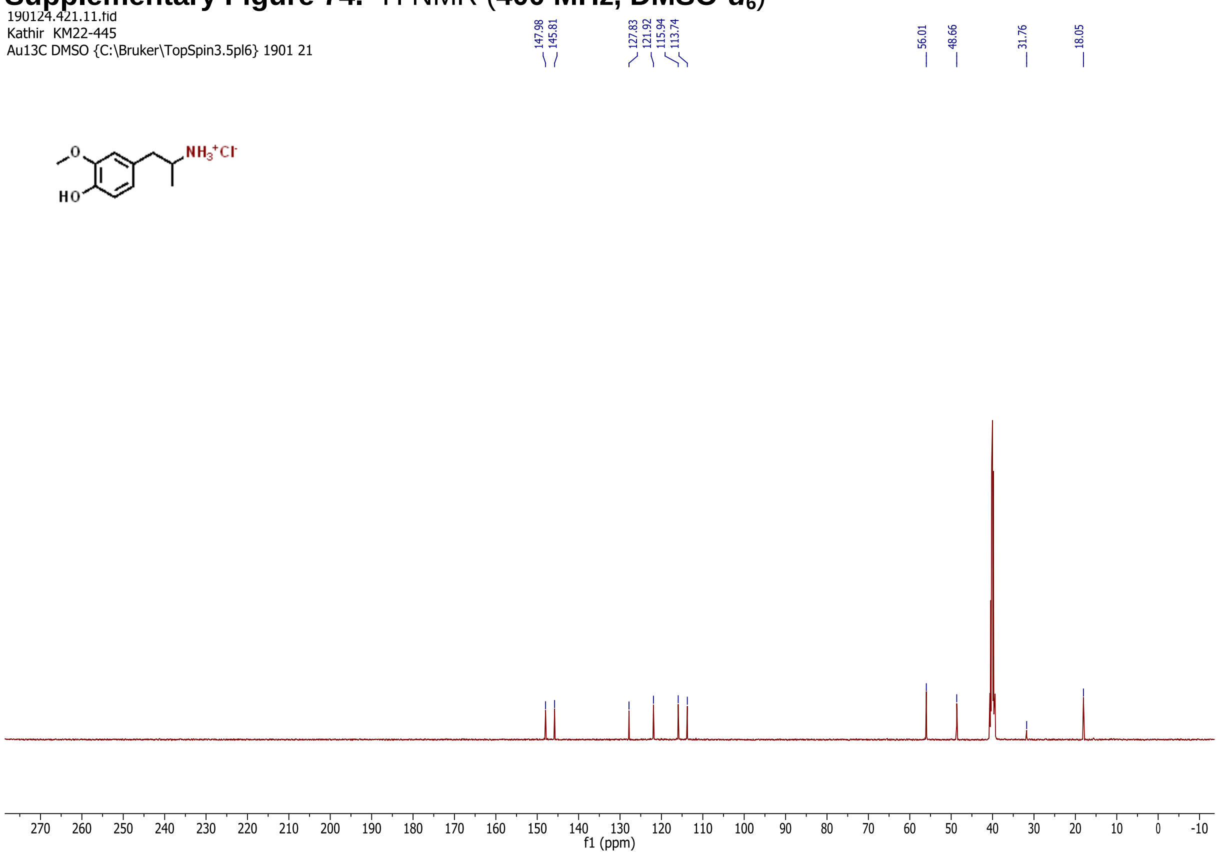

Supplementary Figure 75. ^{13}C NMR (**101 MHz, DMSO-*d*$_6$**)

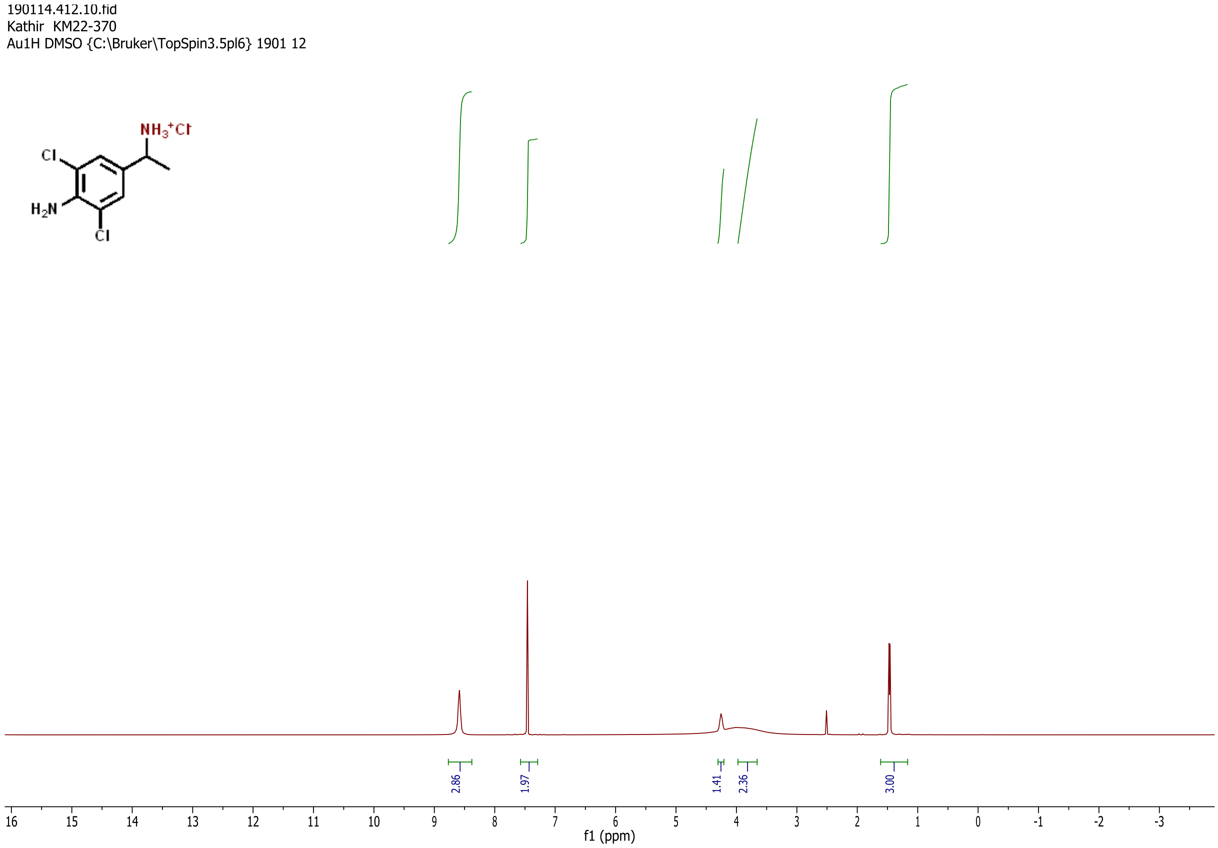

Supplementary Figure 76. ^{1}H NMR (**400 MHz, DMSO-d_6**)

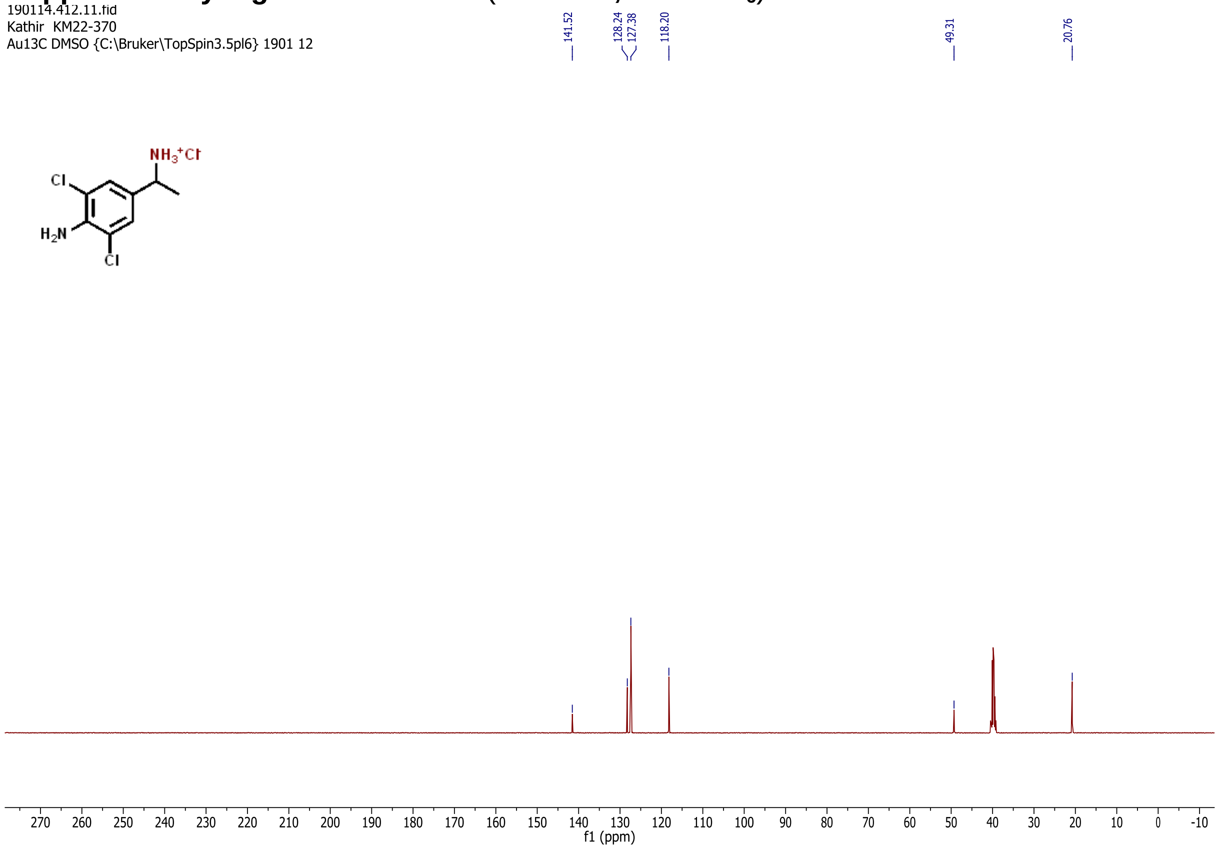

Supplementary Figure 77. ^{13}C NMR (**101 MHz, DMSO-d_6**)

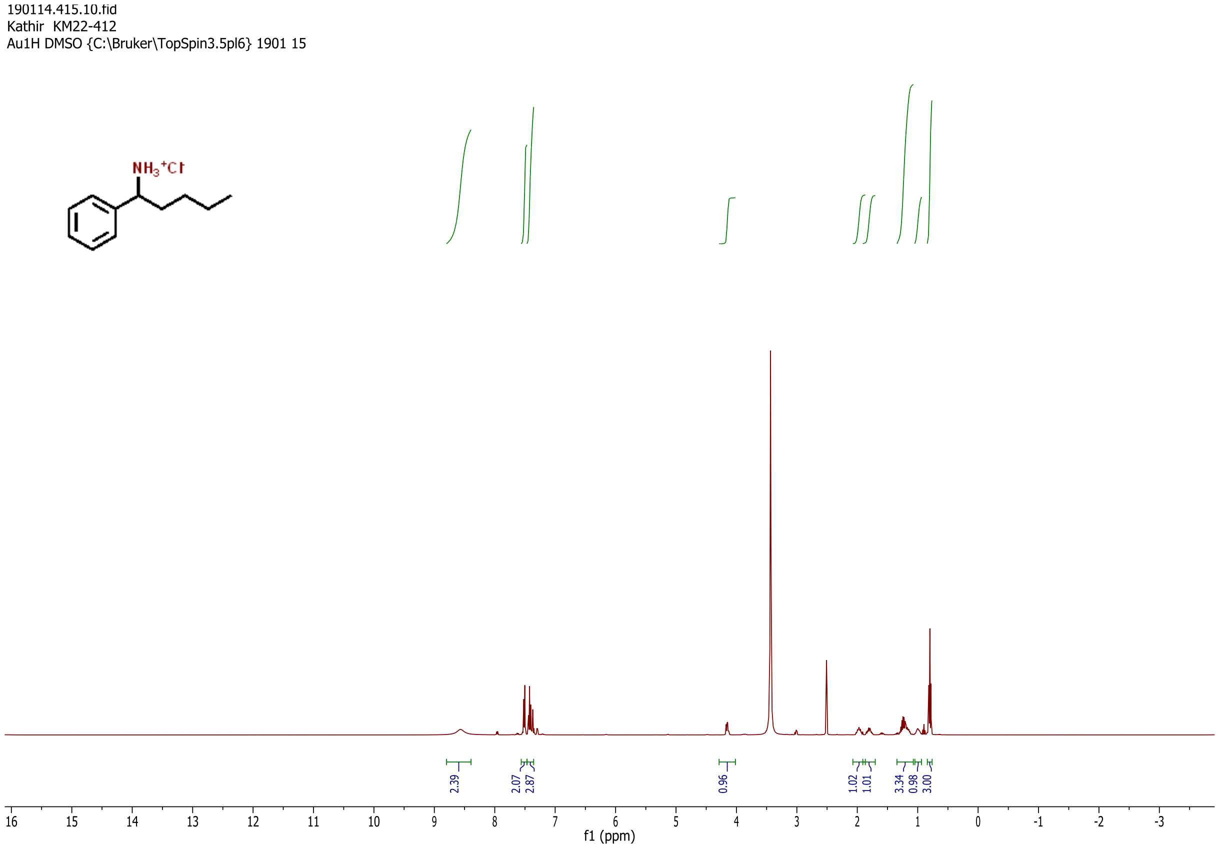

Supplementary Figure 78. ^{1}H NMR (**400 MHz, DMSO-*d*$_6$**)

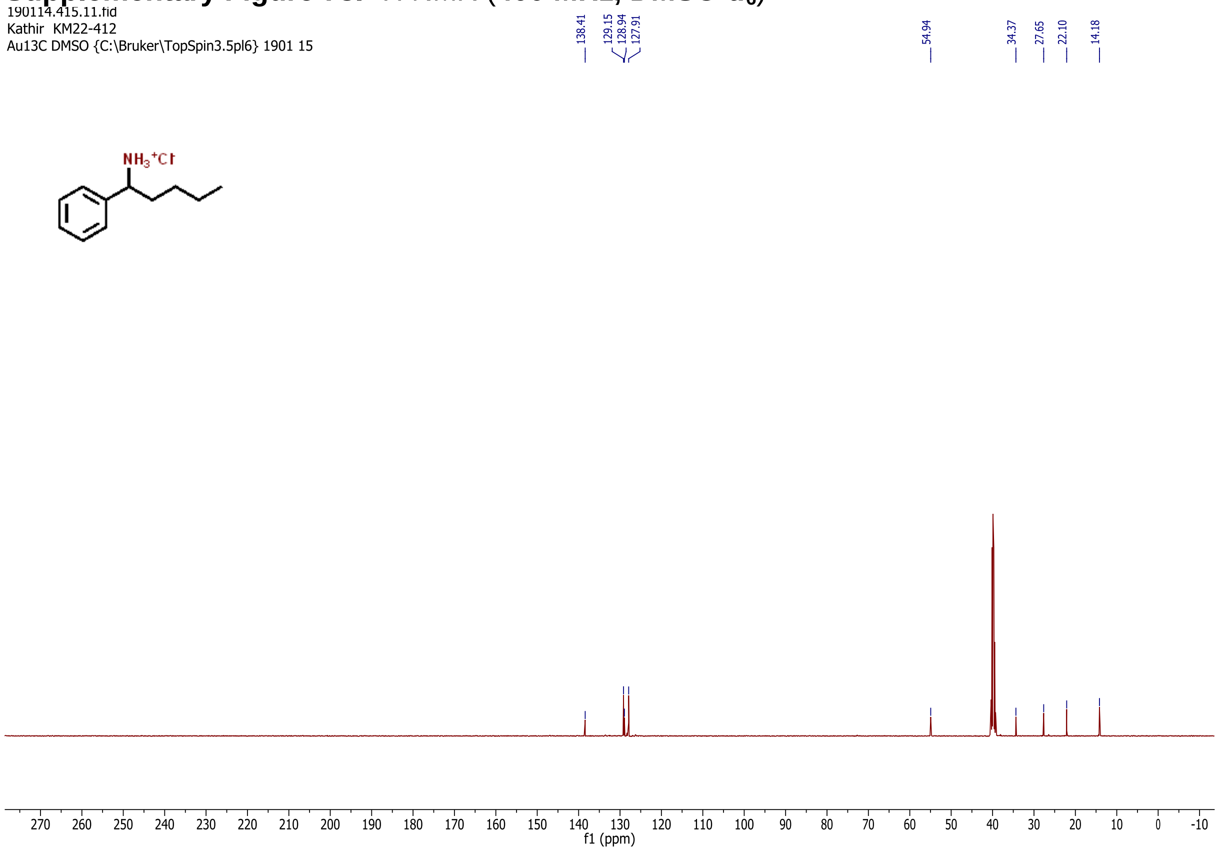

Supplementary Figure 79. ^{13}C NMR (**101 MHz, DMSO-*d*$_6$**)

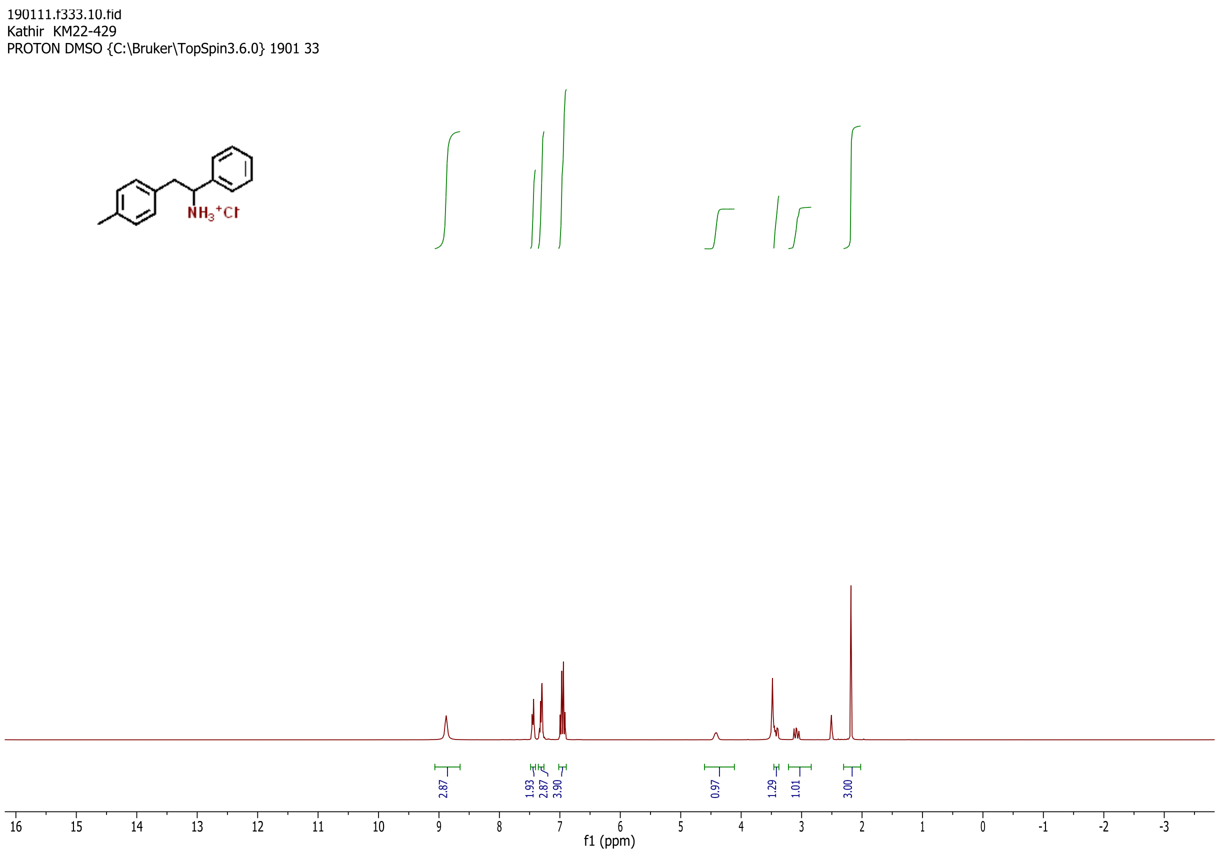

Supplementary Figure 80. ^{1}H NMR (**300 MHz, DMSO-d_6**)

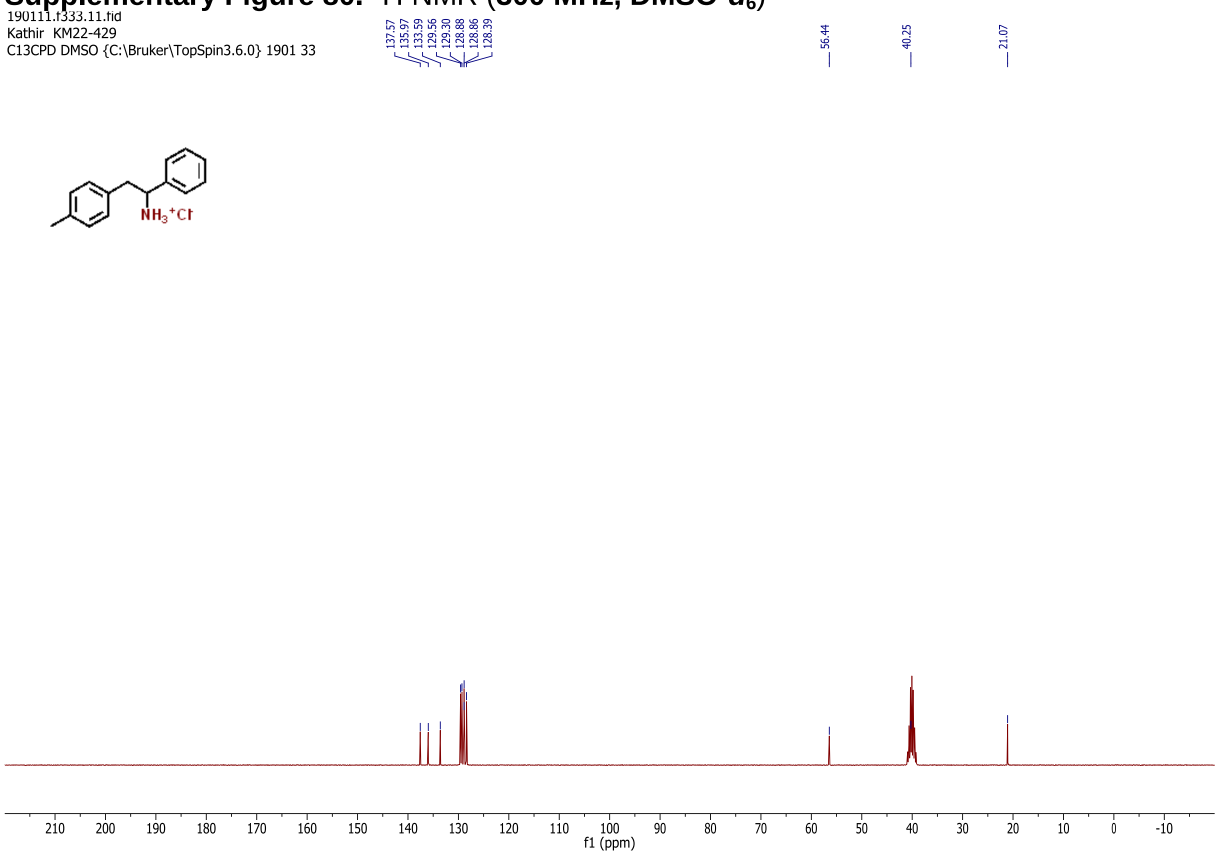

Supplementary Figure 81. ^{13}C NMR (**75 MHz, DMSO-d_6**)

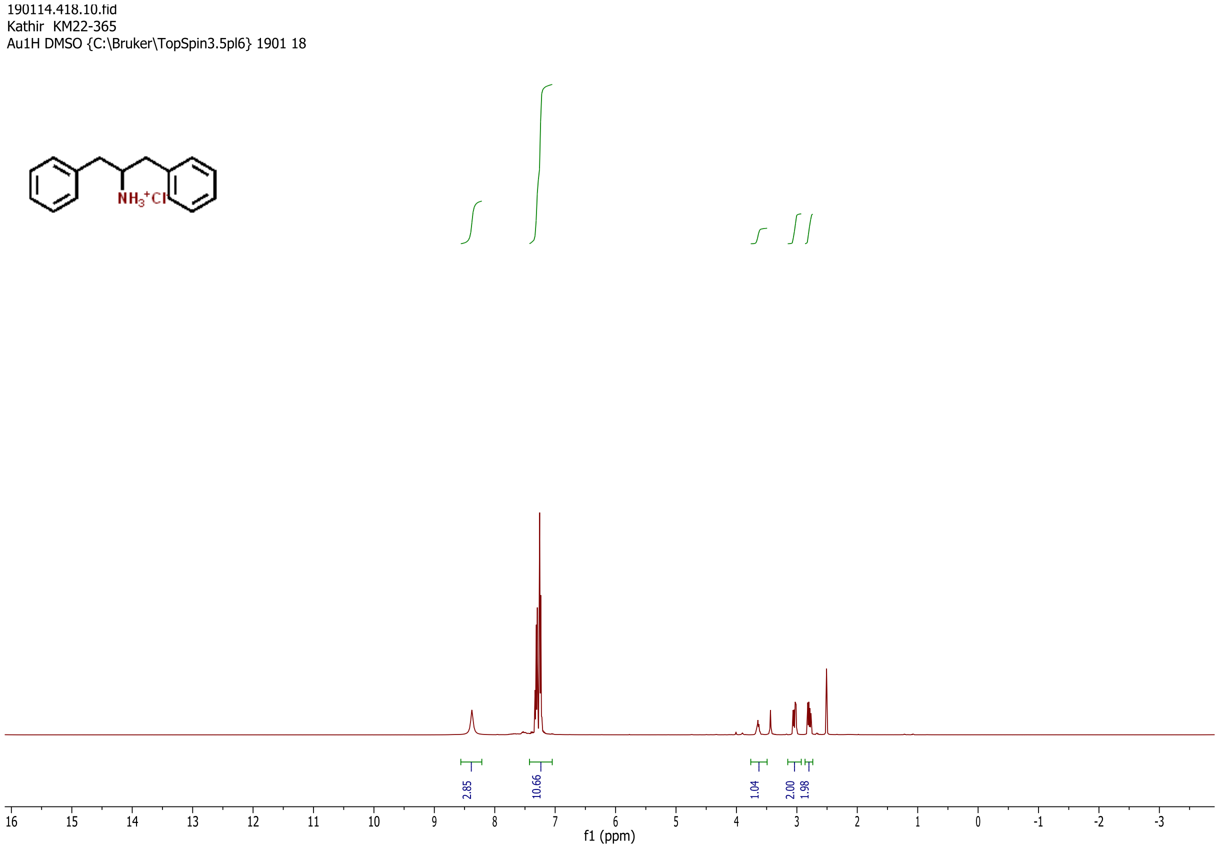

Supplementary Figure 82. ^{1}H NMR (**400 MHz, DMSO-d_6**)

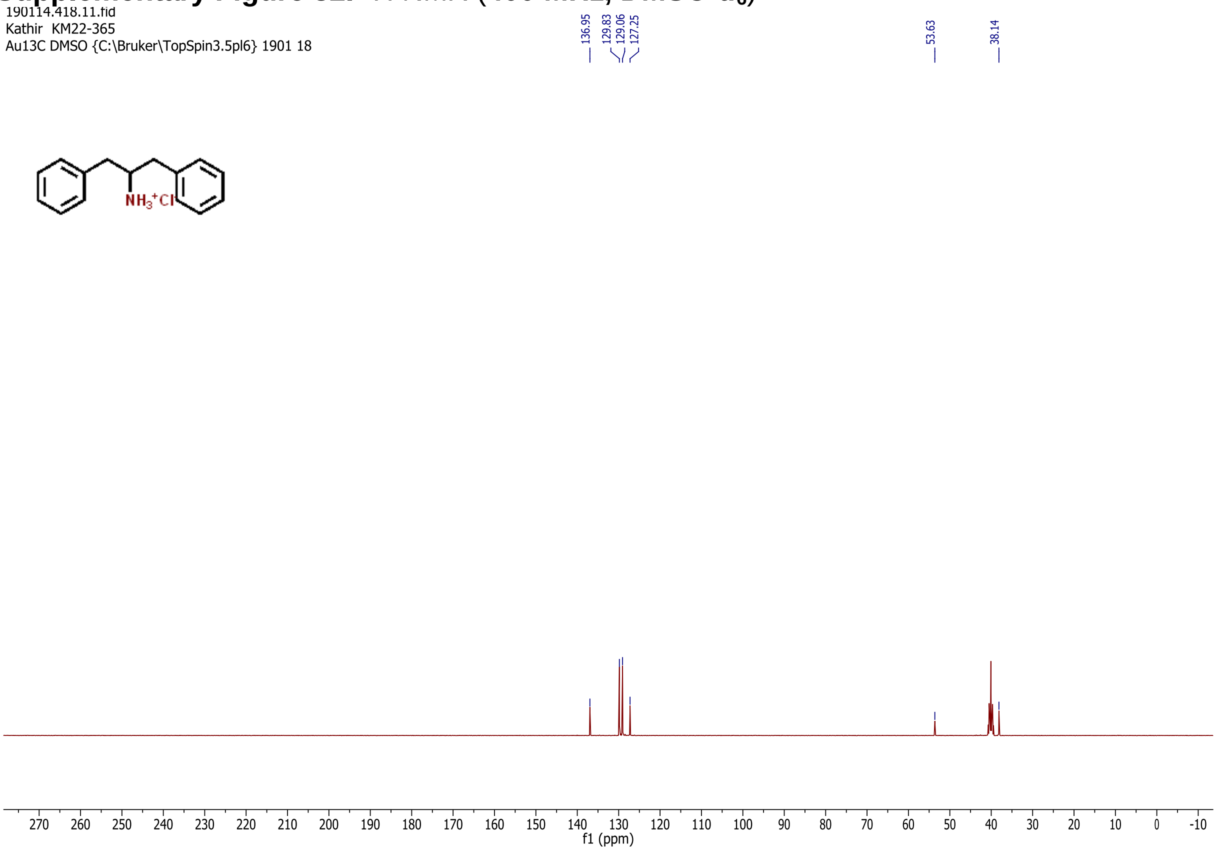

Supplementary Figure 83. ^{13}C NMR (**101 MHz, DMSO-d_6**)

190114.416.10.fid
Kathir KM22-399
Au1H DMSO {C:\Bruker\TopSpin3.5pl6} 1901 16

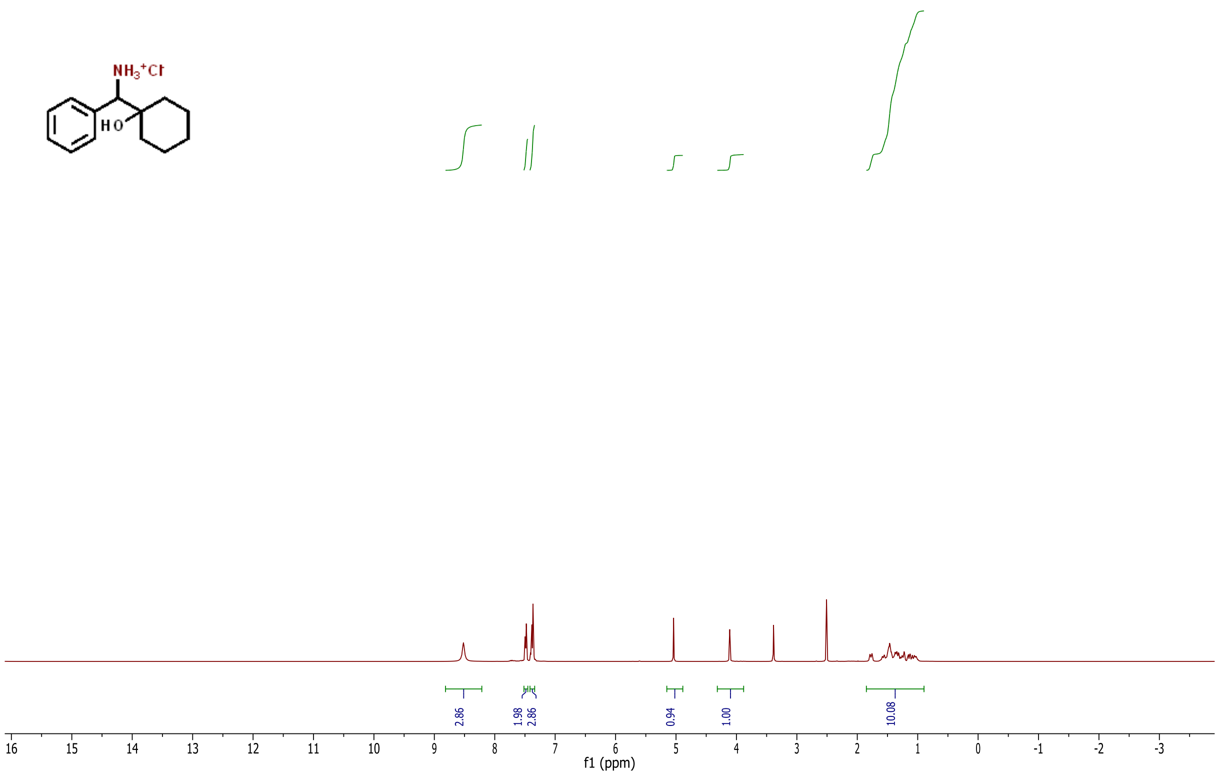

Supplementary Figure 84. ^{1}H NMR (**400 MHz, DMSO-d_6**)

190114.416.11.fid
Kathir KM22-399
Au13C DMSO {C:\Bruker\TopSpin3.5pl6} 1901 16

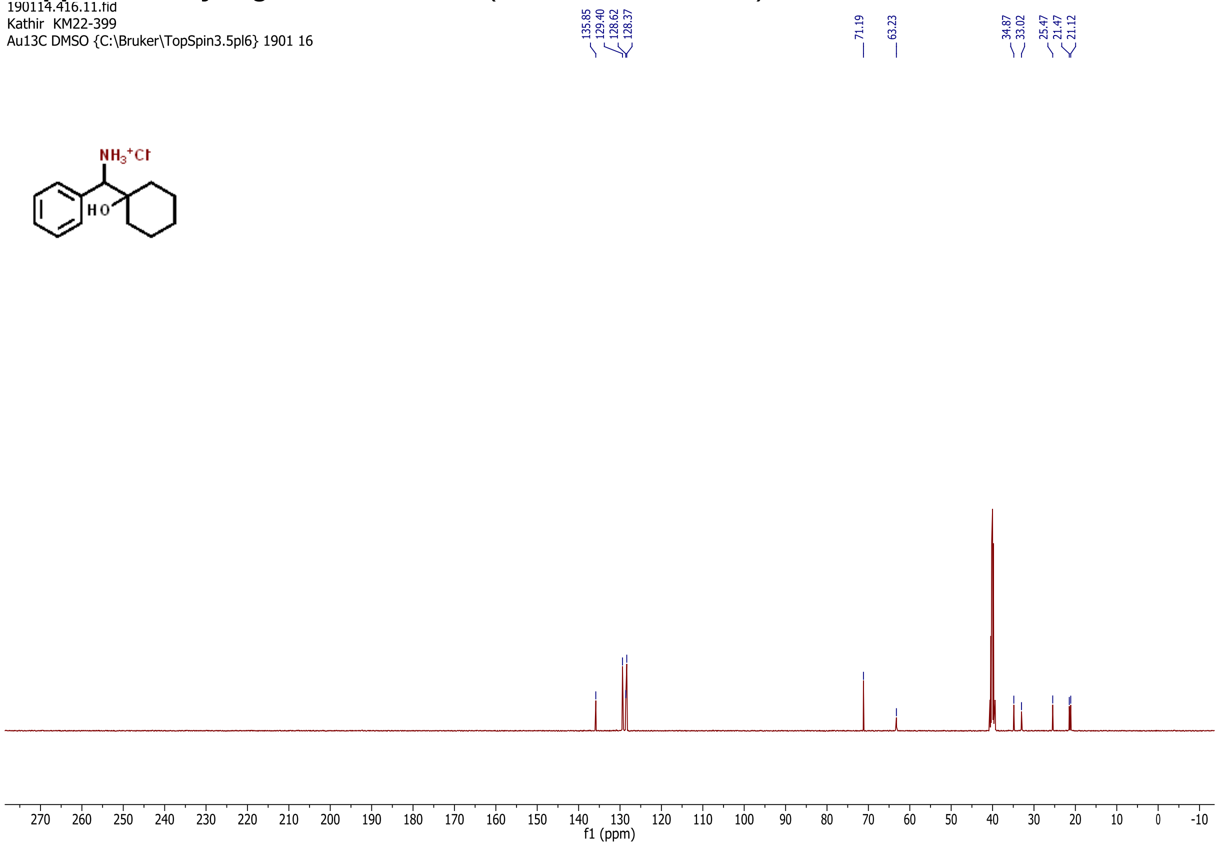

Supplementary Figure 85. ^{13}C NMR (**101 MHz, DMSO-d_6**)

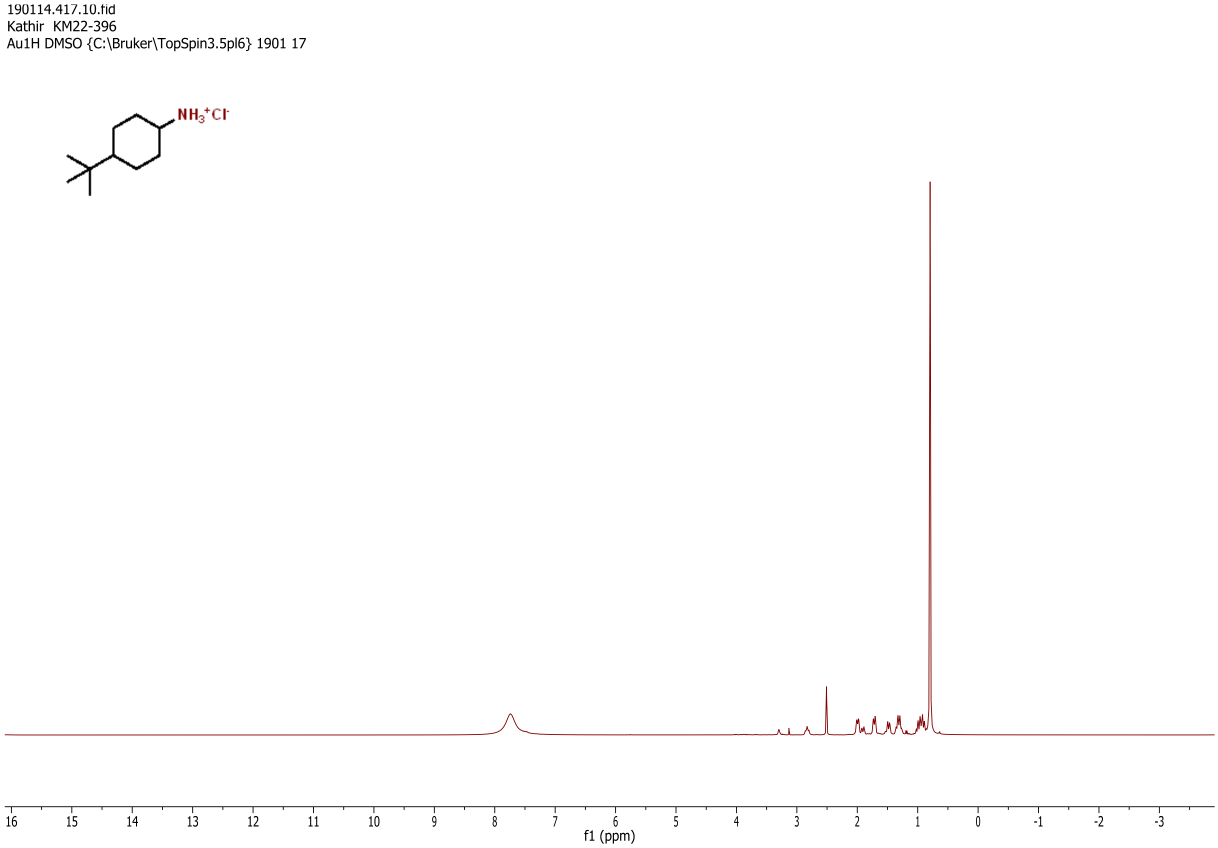

Supplementary Figure 86. ^{1}H NMR (**400 MHz, DMSO-*d*$_6$**)

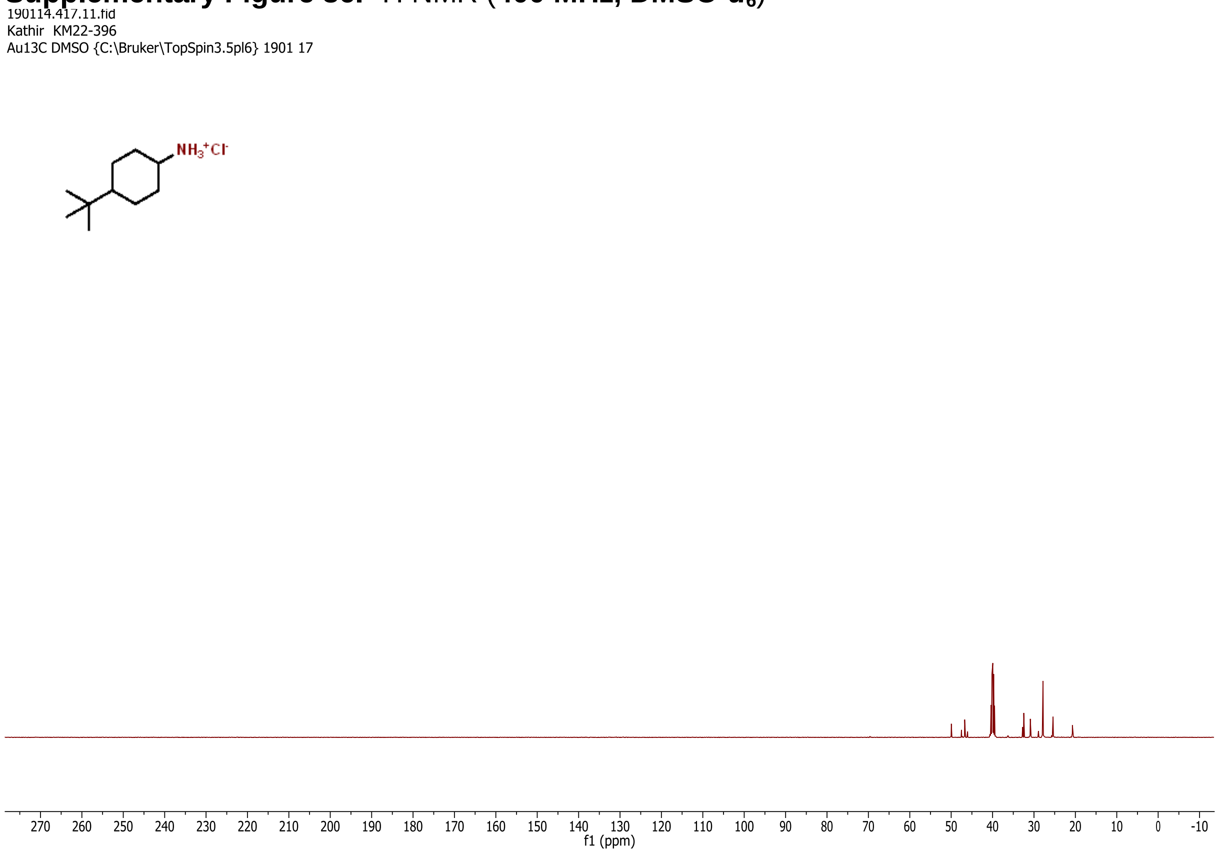

Supplementary Figure 87. ^{13}C NMR (**101 MHz, DMSO-*d*$_6$**)

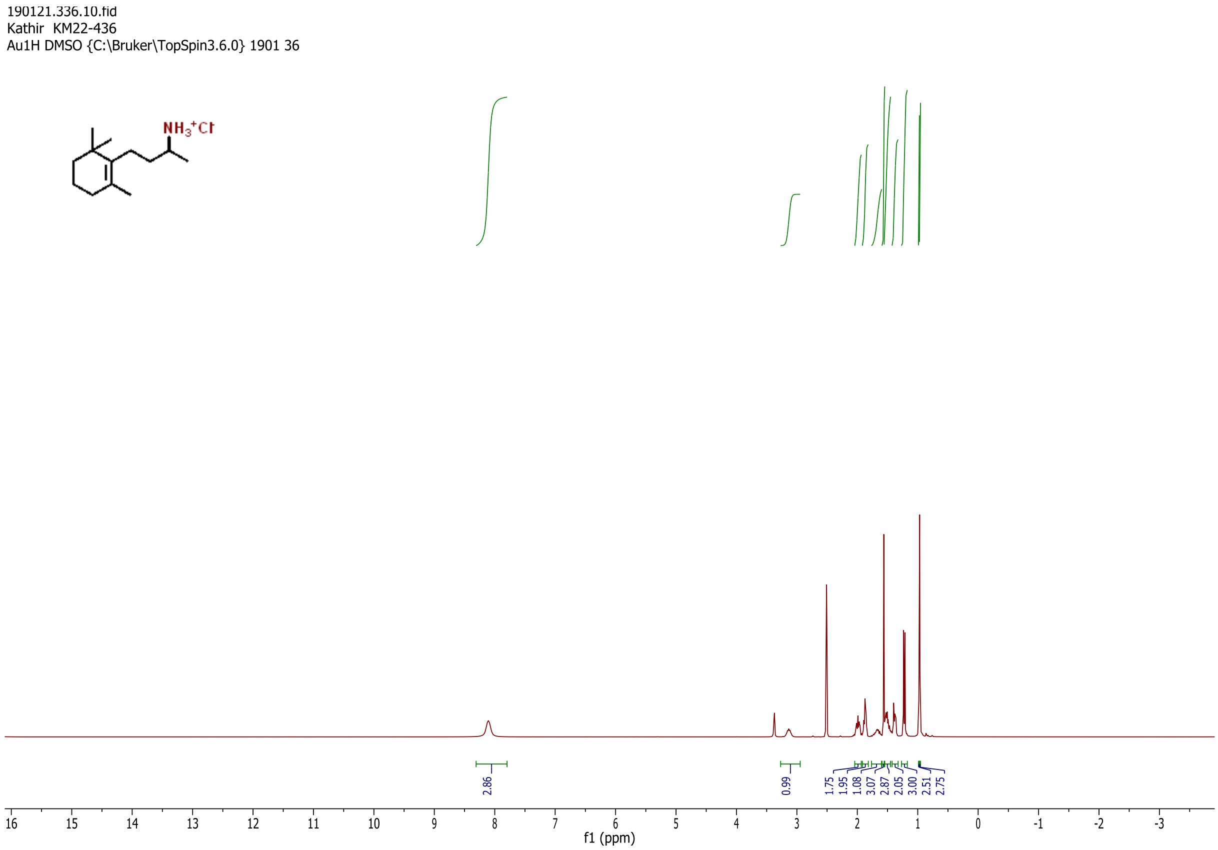

Supplementary Figure 88. ^{1}H NMR (**300 MHz, DMSO-d_6**)

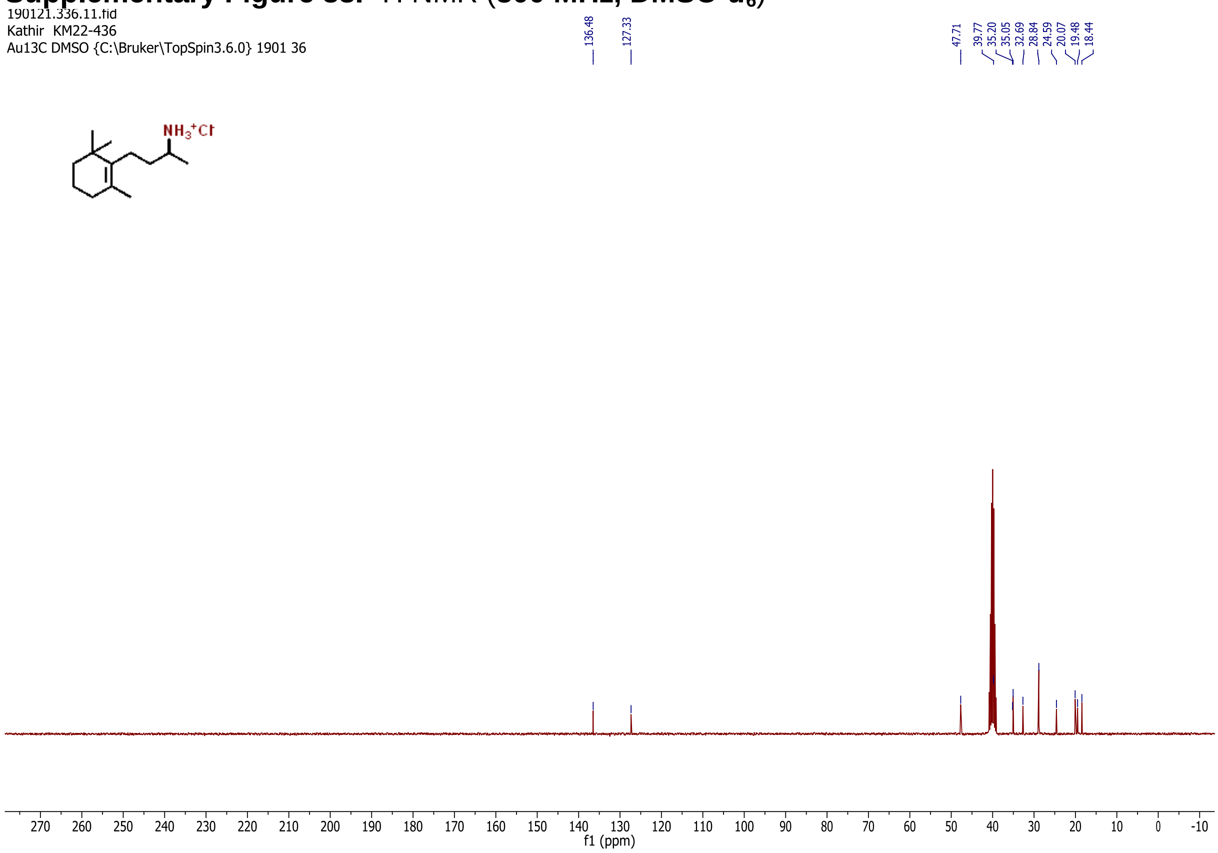

Supplementary Figure 89. ^{13}C NMR (**75 MHz, DMSO-d_6**)

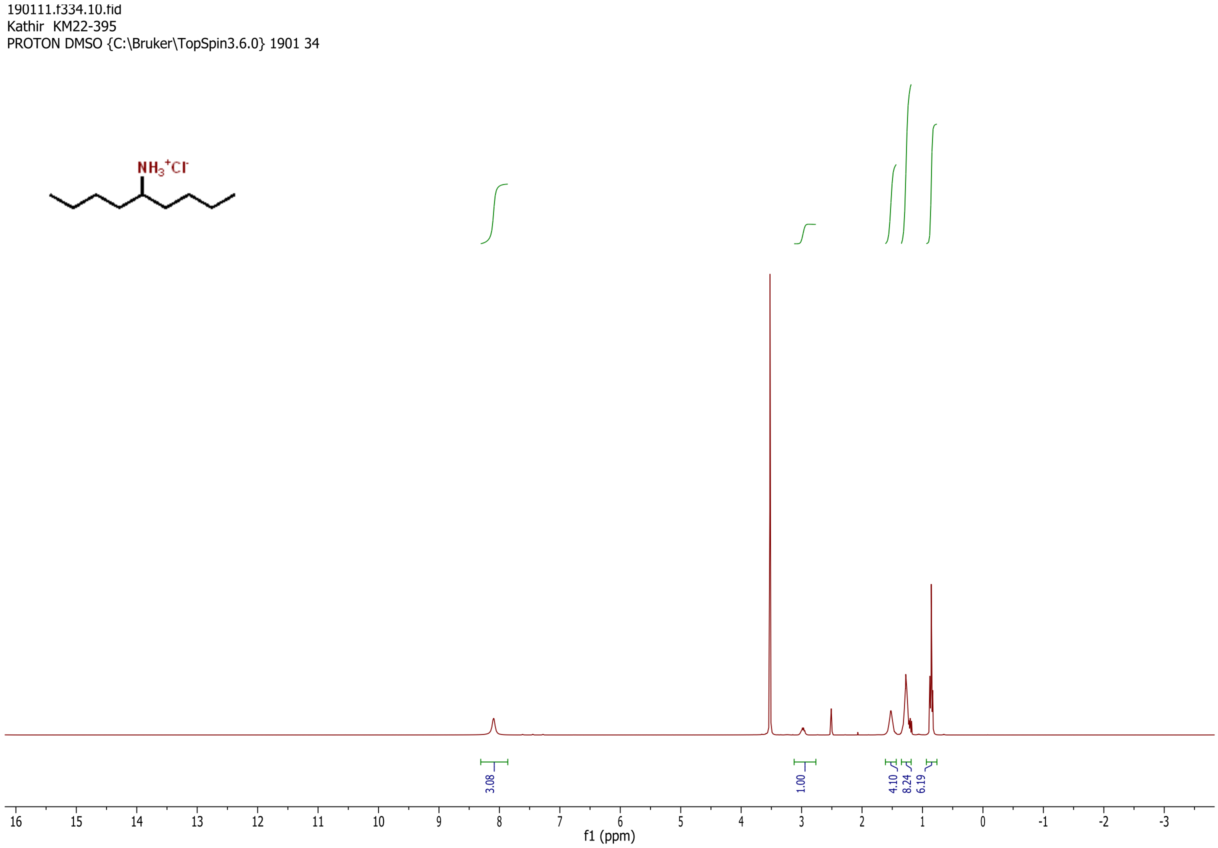

Supplementary Figure 90. ^{1}H NMR (**300 MHz, DMSO-*d*$_6$**)

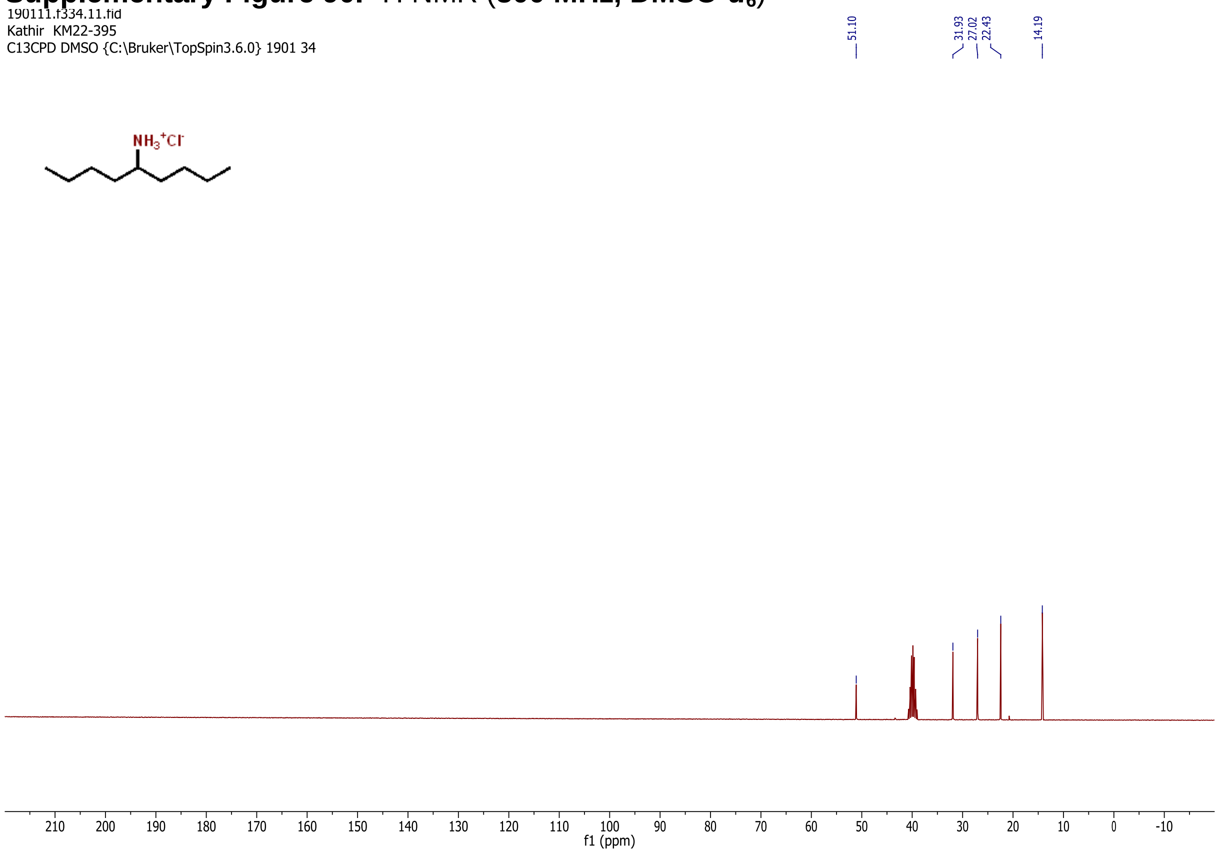

Supplementary Figure 91. ^{13}C NMR (**75 MHz, DMSO-*d*$_6$**)

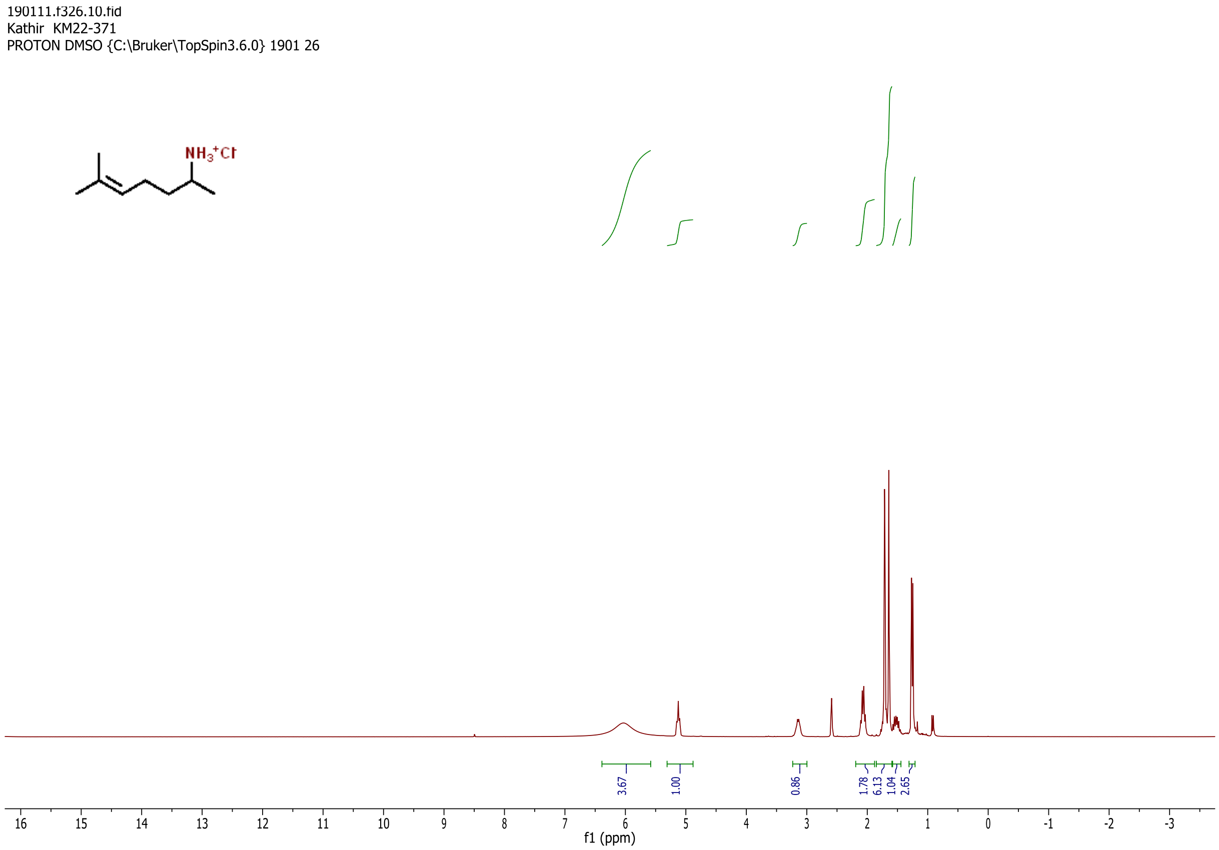

Supplementary Figure 92. ^{1}H NMR (**300 MHz, DMSO-*d*$_6$**)

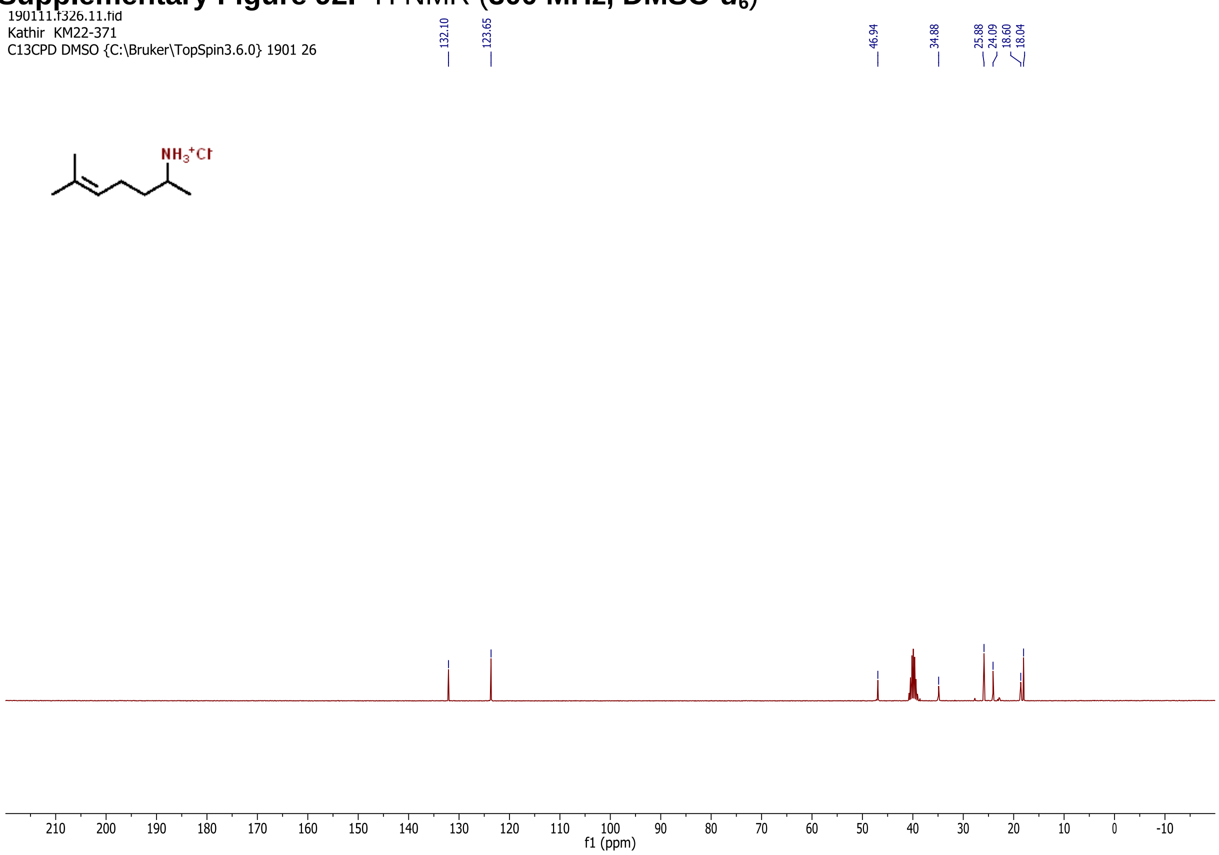

Supplementary Figure 93. ^{13}C NMR (**75 MHz, DMSO-*d*$_6$**)

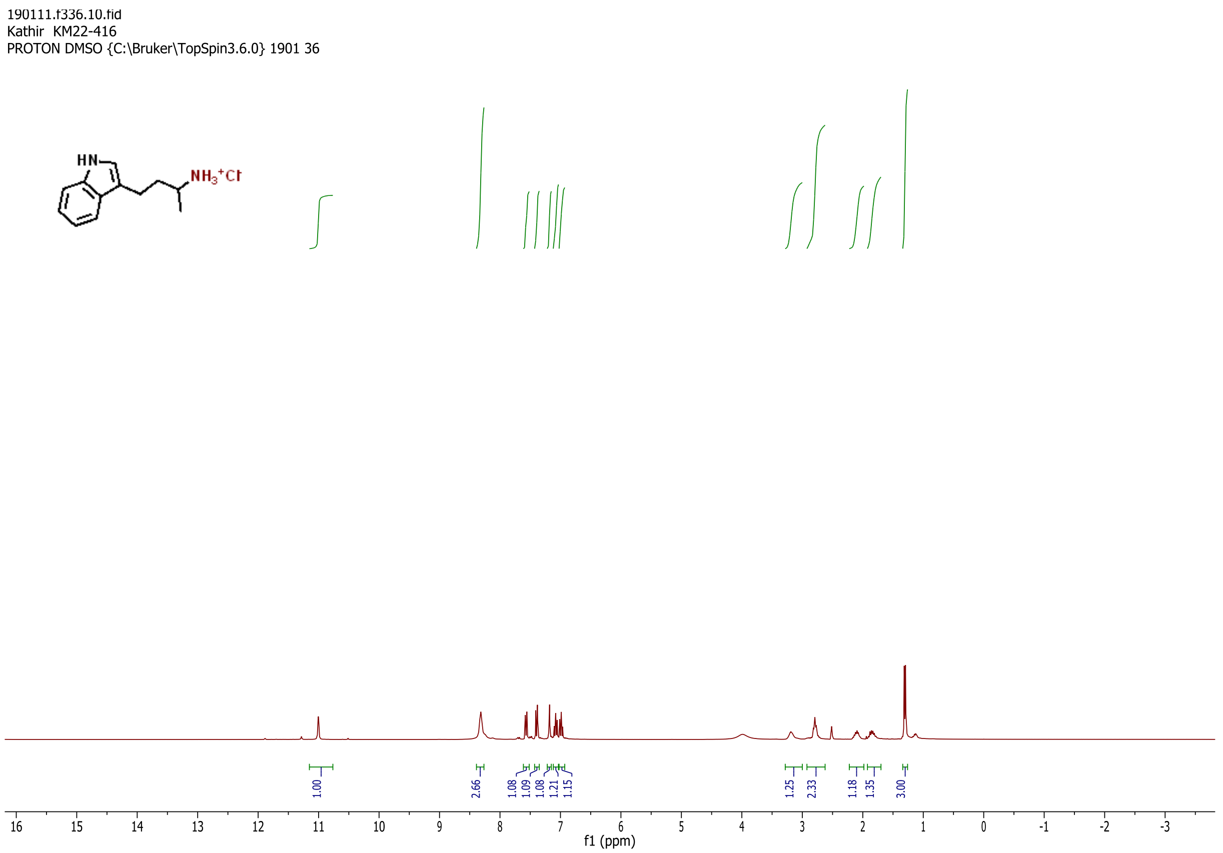

Supplementary Figure 94. ^{1}H NMR (**300 MHz, DMSO-*d*$_6$**)

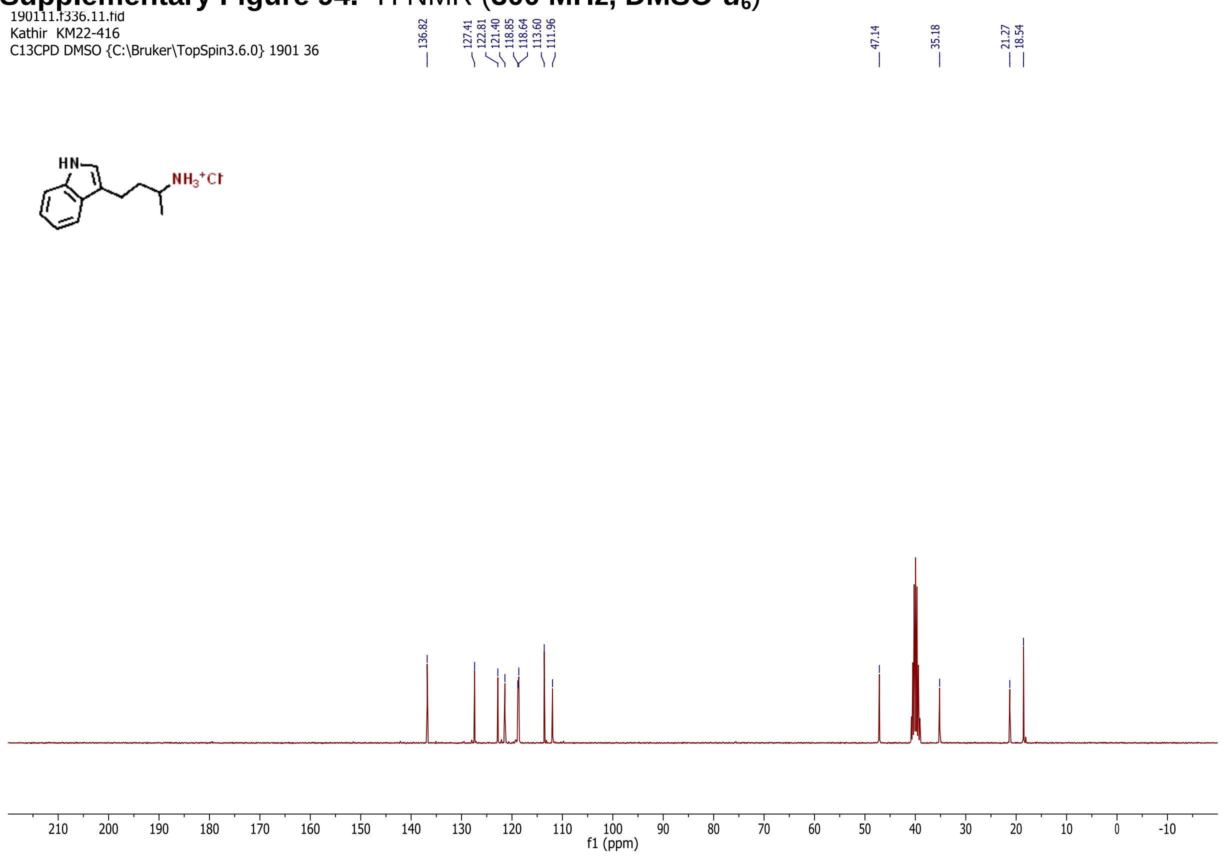

Supplementary Figure 95. ^{13}C NMR (**75 MHz, DMSO-*d*$_6$**)

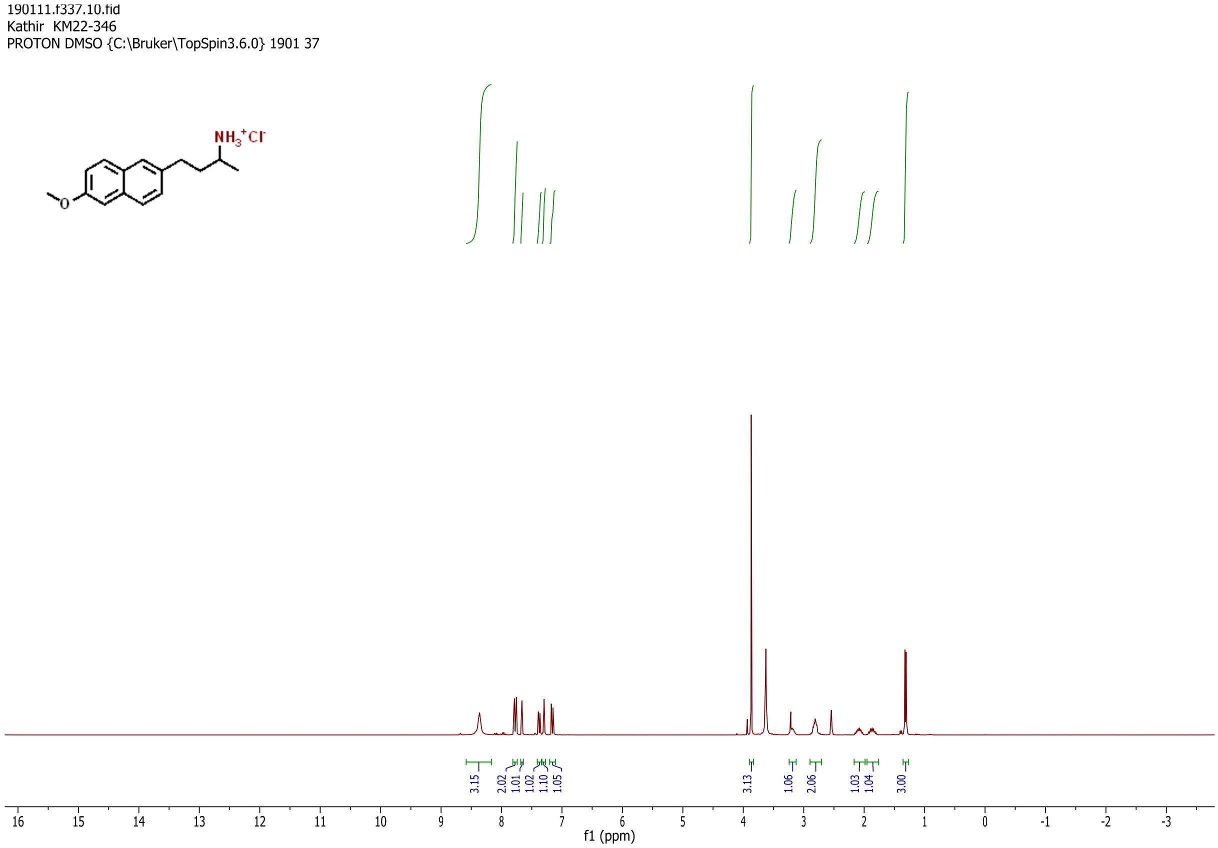

Supplementary Figure 96. ^{1}H NMR (**300 MHz, DMSO-*d*$_6$**)

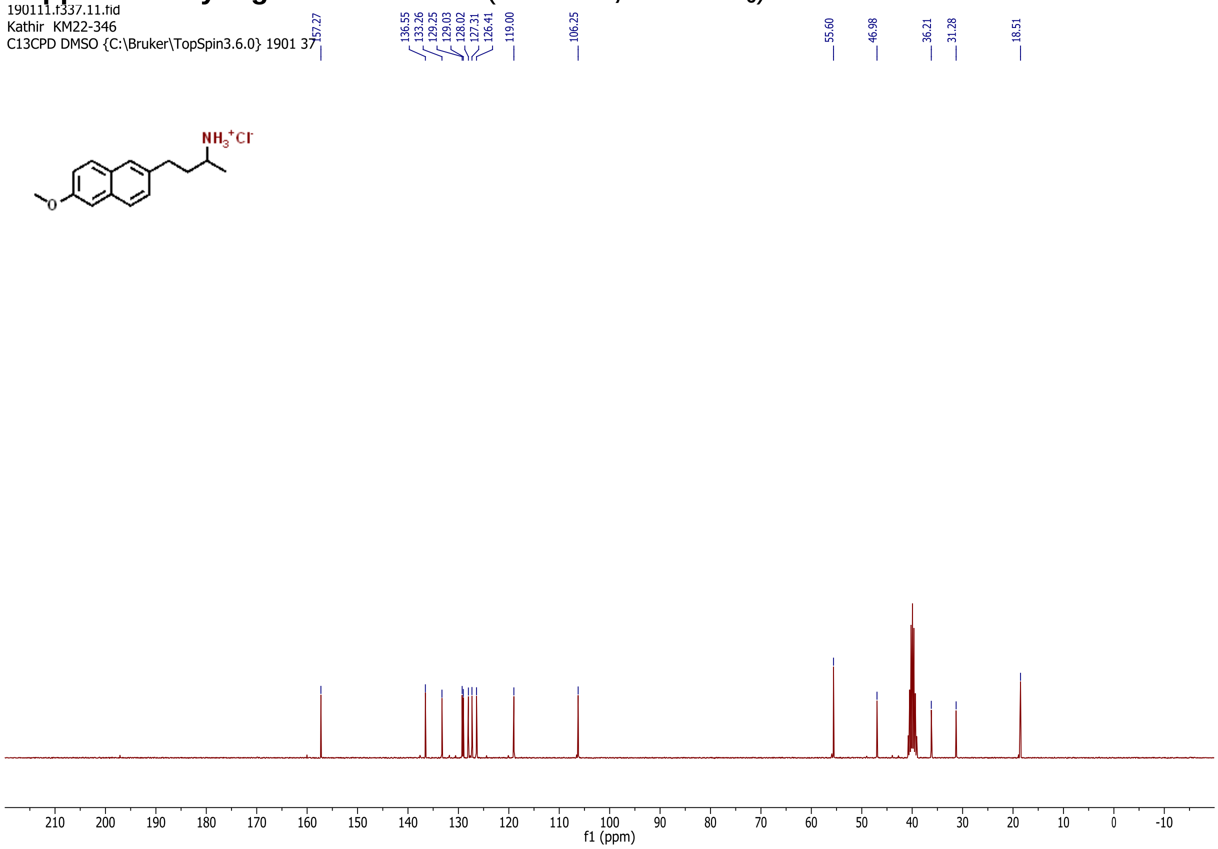

Supplementary Figure 97. ^{13}C NMR (**75 MHz, DMSO-*d*$_6$**)

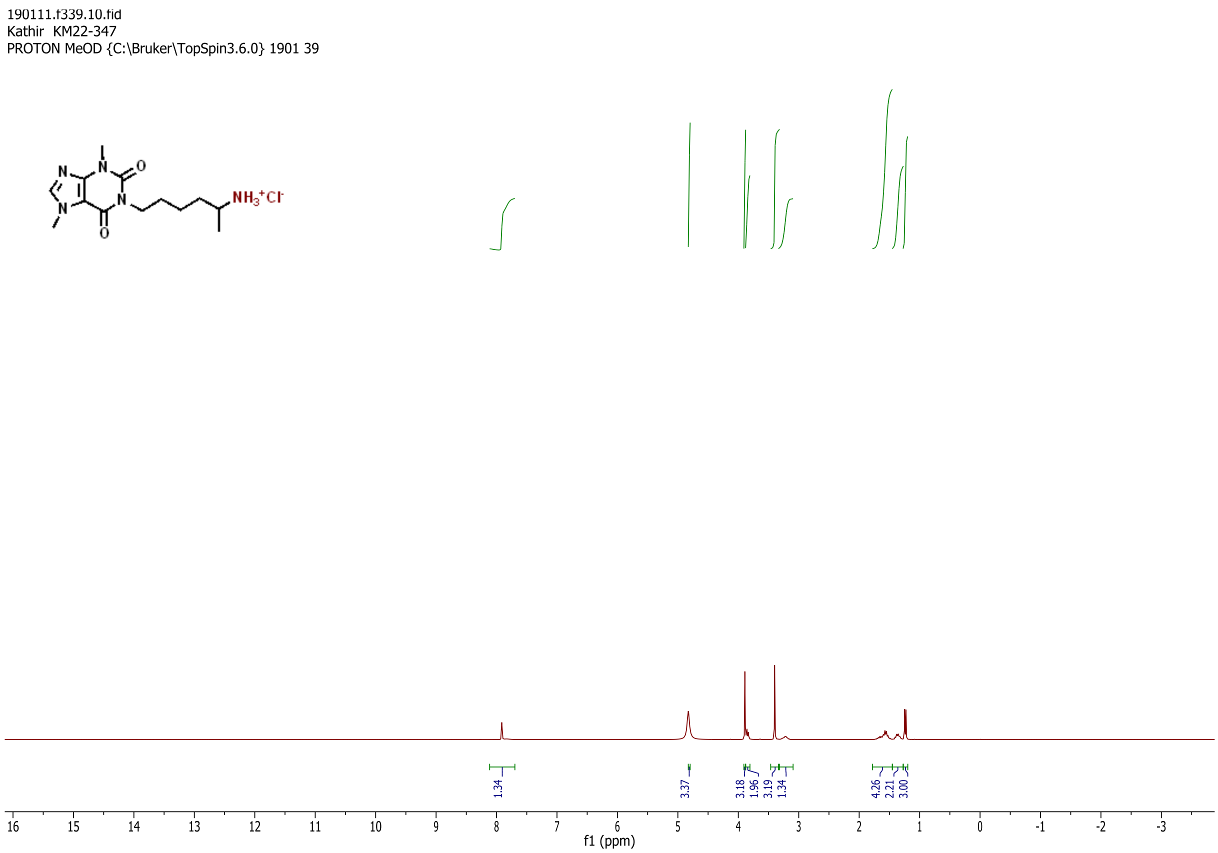

Supplementary Figure 98. ^{1}H NMR (**300 MHz, Methanol-*d*$_4$**)

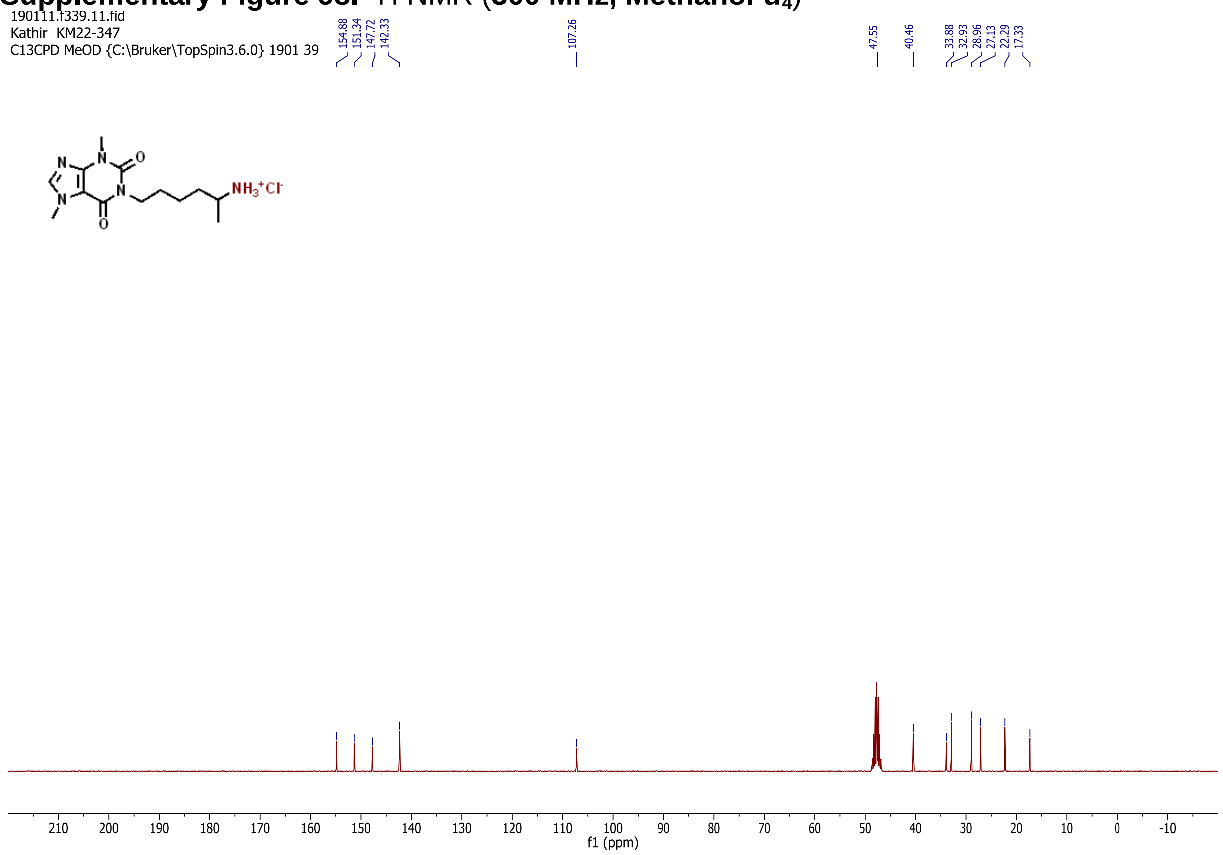

Supplementary Figure 99. ^{13}C NMR (**75 MHz, Methanol-*d*$_4$**)

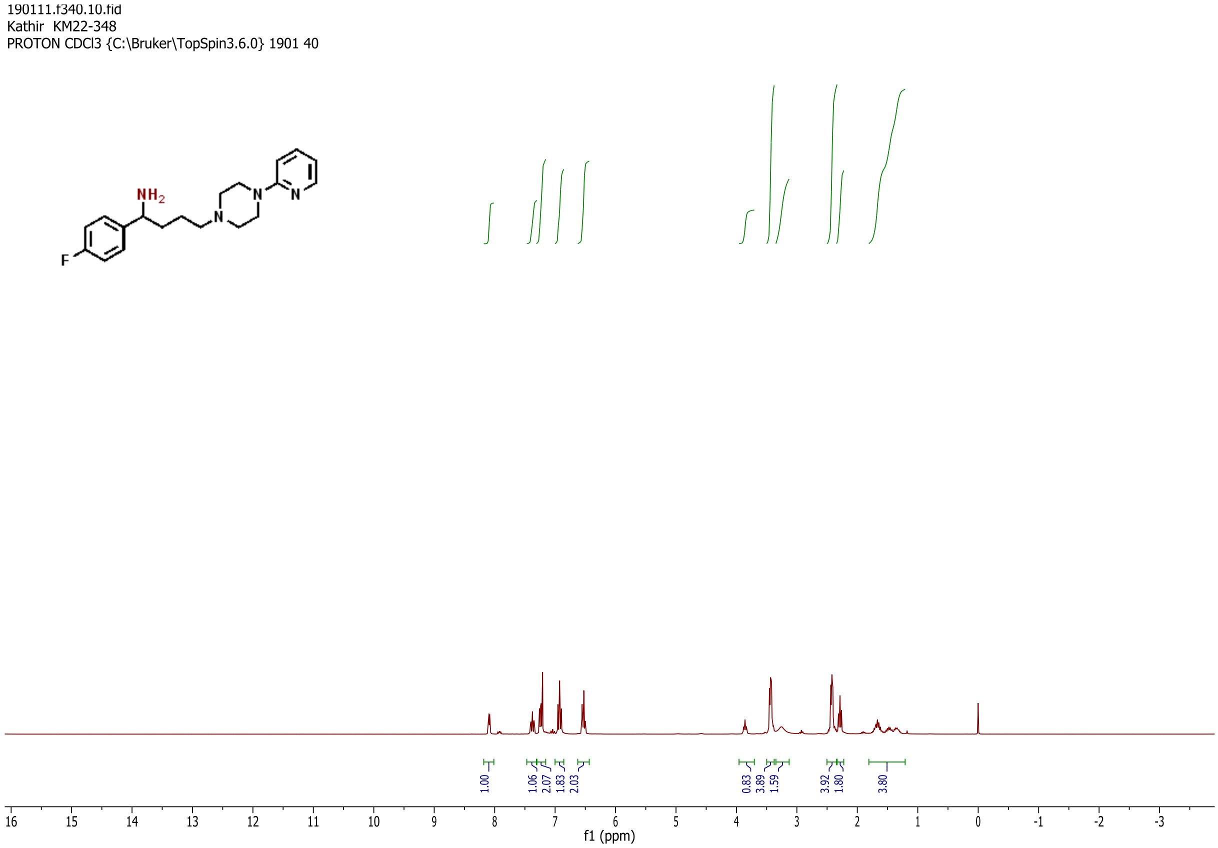

Supplementary Figure 100. ^{1}H NMR (**300 MHz, Chloroform-*d***)

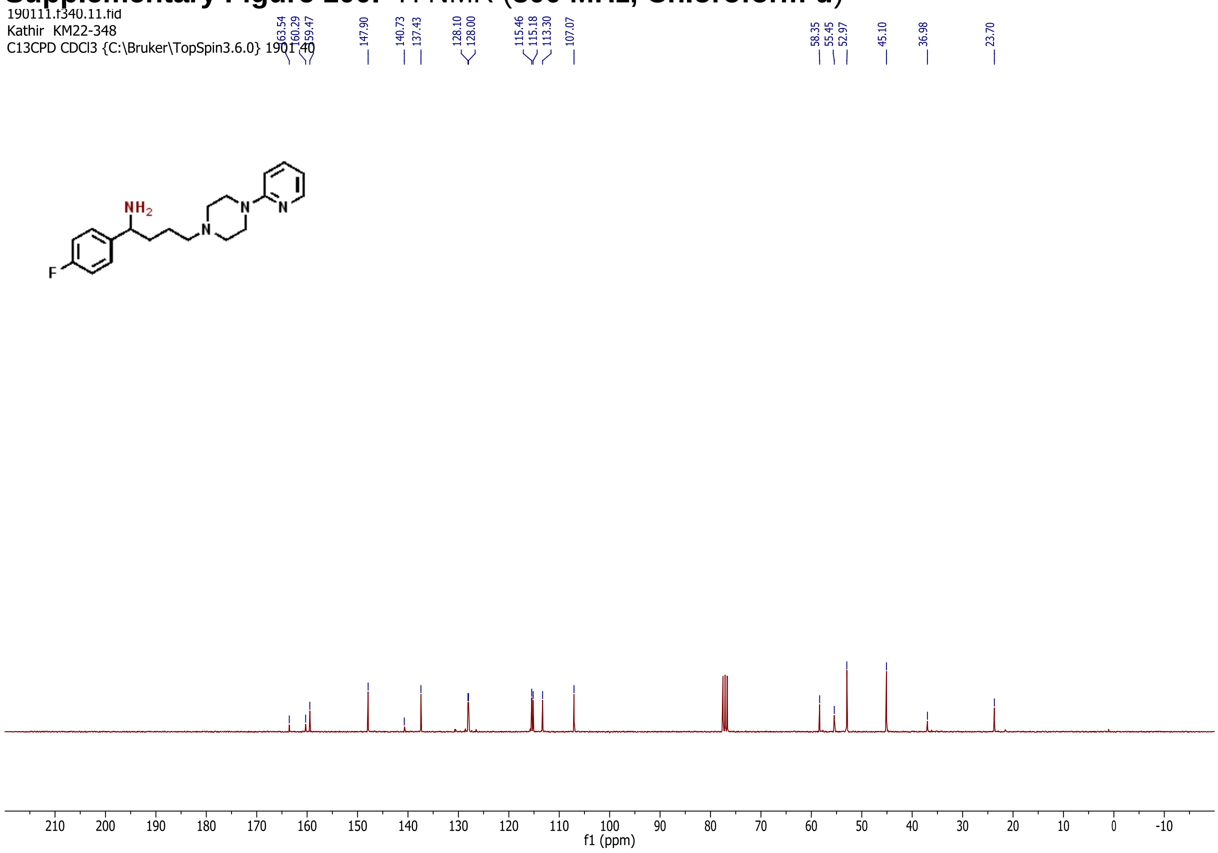

Supplementary Figure 101. ^{13}C NMR (**75 MHz, Chloroform-*d***)

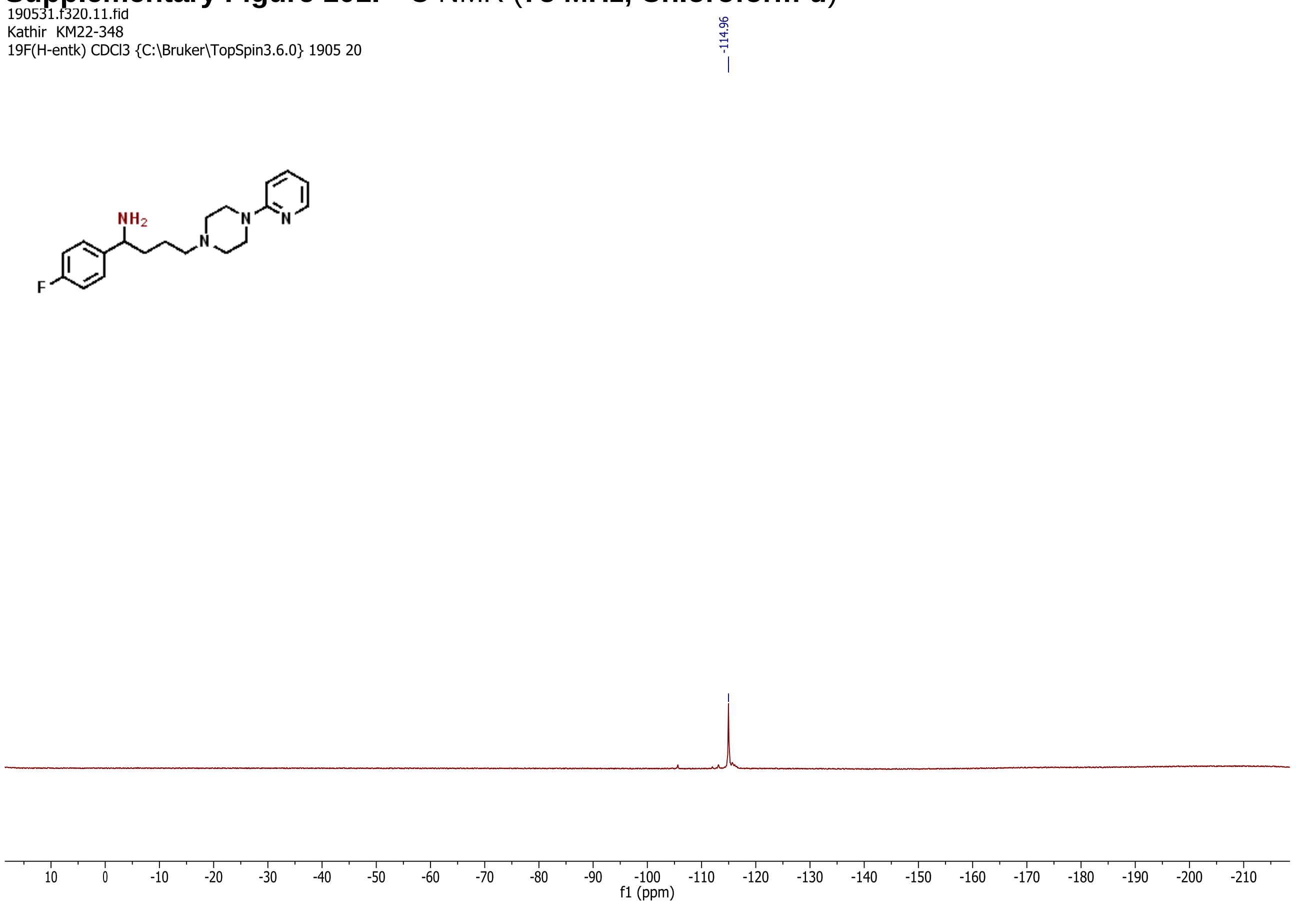

Supplementary Figure 102. ^{19}F NMR **(282 MHz, Chloroform-*d*)**

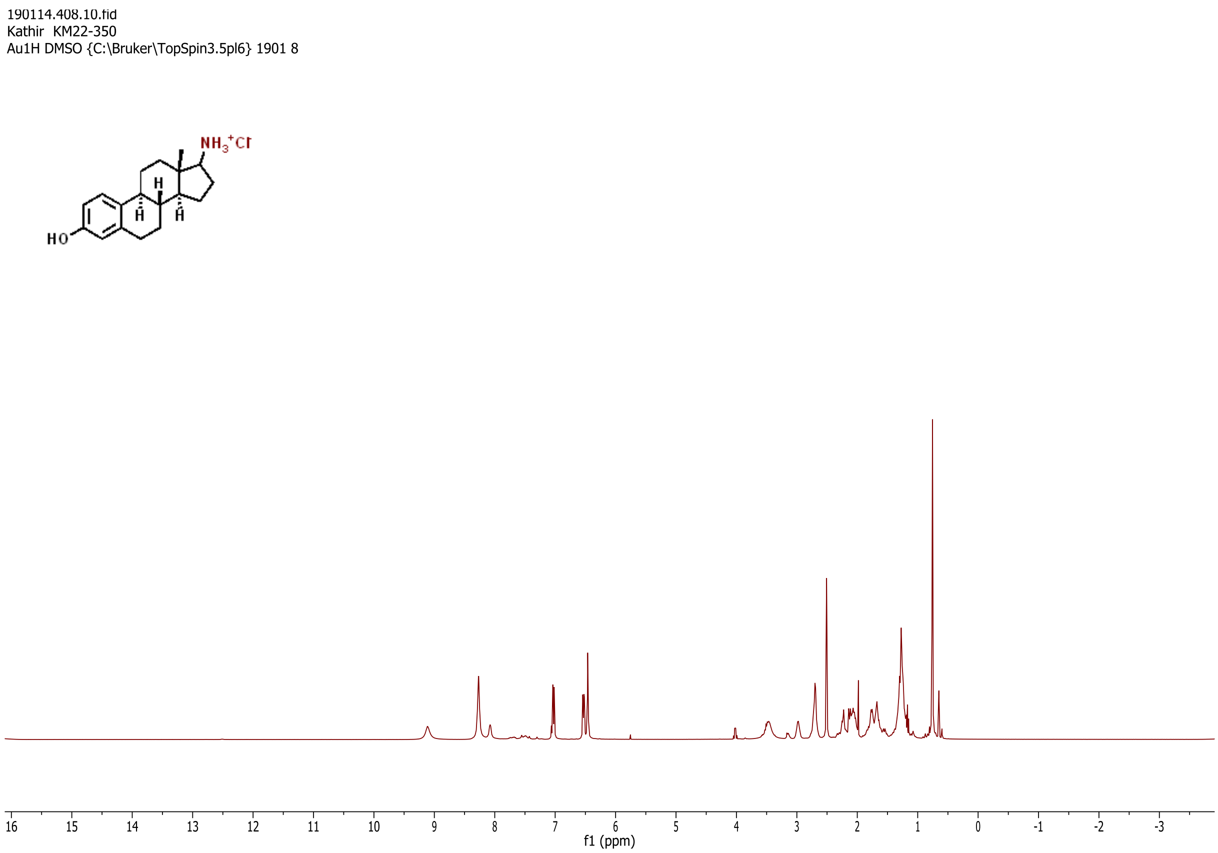

Supplementary Figure 103. ^{1}H NMR (**400 MHz, DMSO-*d*$_6$**)

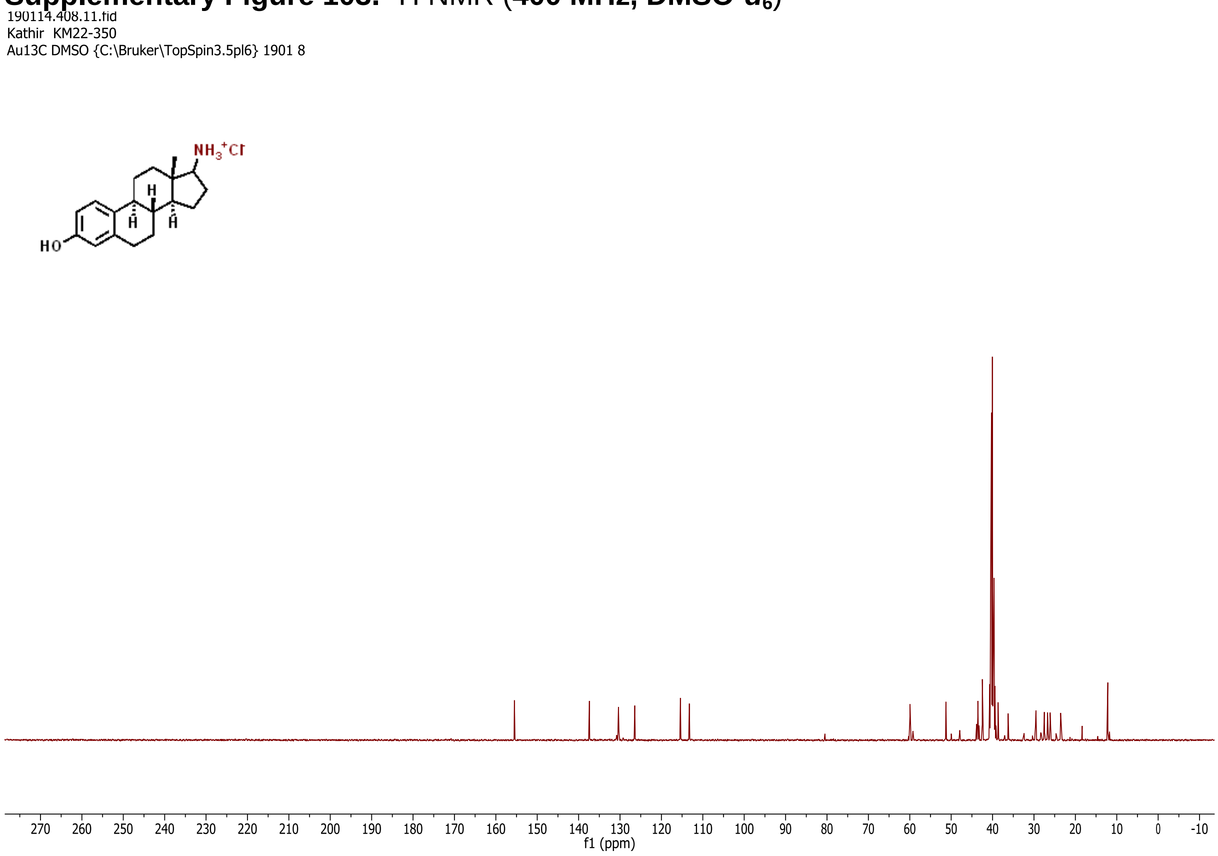

Supplementary Figure 104. ^{13}C NMR (**101 MHz, DMSO-*d*$_6$**)

ESI-TOF Accurate Mass Report Page 1

Results file: E:\Projects\1901.PRO\SampleDB\1901.rpt
Last modified: Monday, January 21, 2019 14:51:00

Sample Summary:

Sample	File	Sample Name	User	Target	Formula	Expected Mass	Observed Mass	Error PPM	Error mDa
187	19012120	KM22-350	Kathir	271.1936	C18H25NO	272.2014	272.2013	-0.4	-0.1

ESI-TOF Accurate Mass Report Page 2

File:19012120
Vial:1:D,2
Description:MeOH/0.1%HCOOH in H2O 9:1

Sample Name:KM22-350
Date:21-Jan-2019

UserName:Kathir
Time:14:47:51

Sample Report:

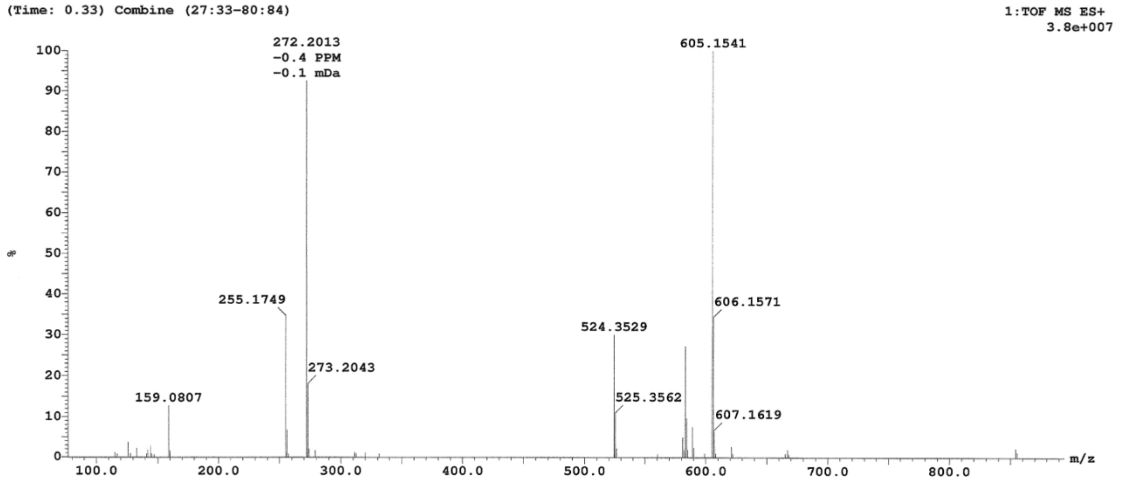

Supplementary Figure 105. HRMS

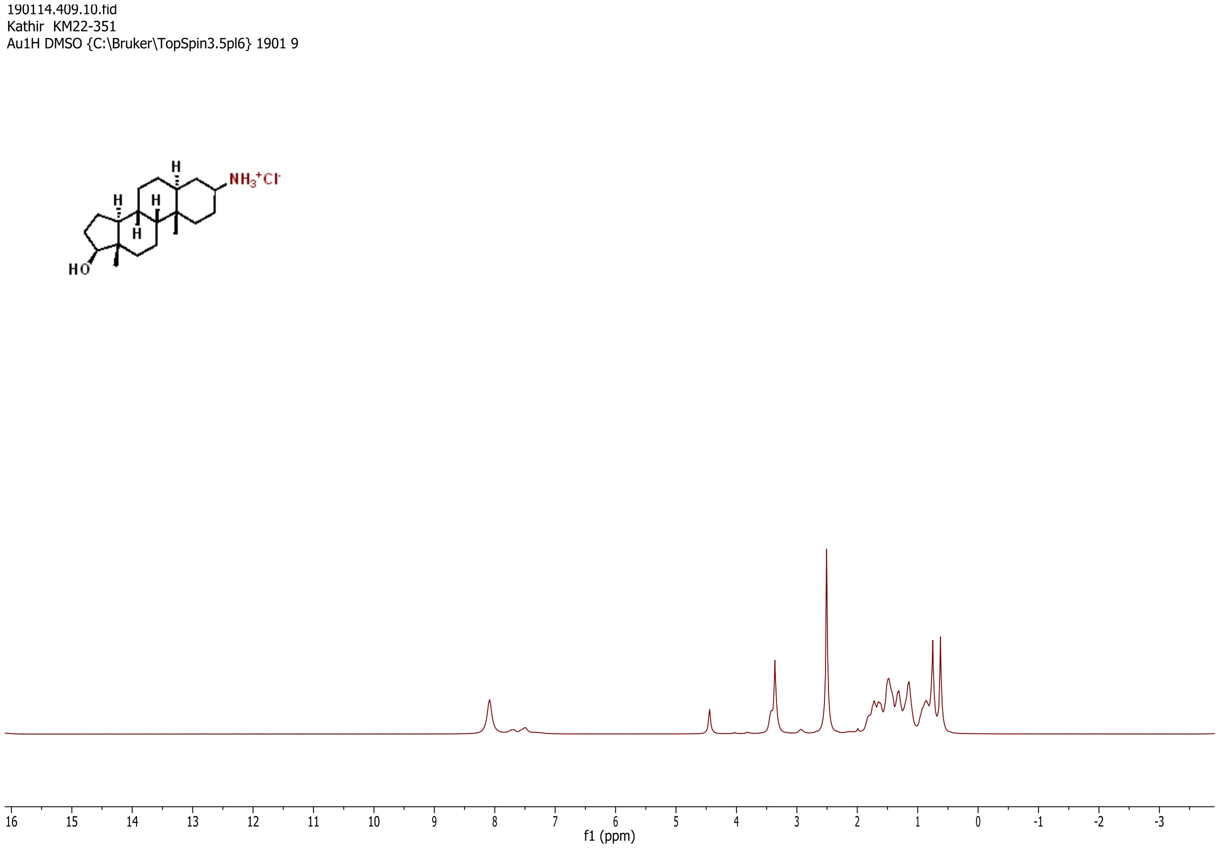

Supplementary Figure 106. ^{1}H NMR (**400 MHz, DMSO-*d*$_6$**)

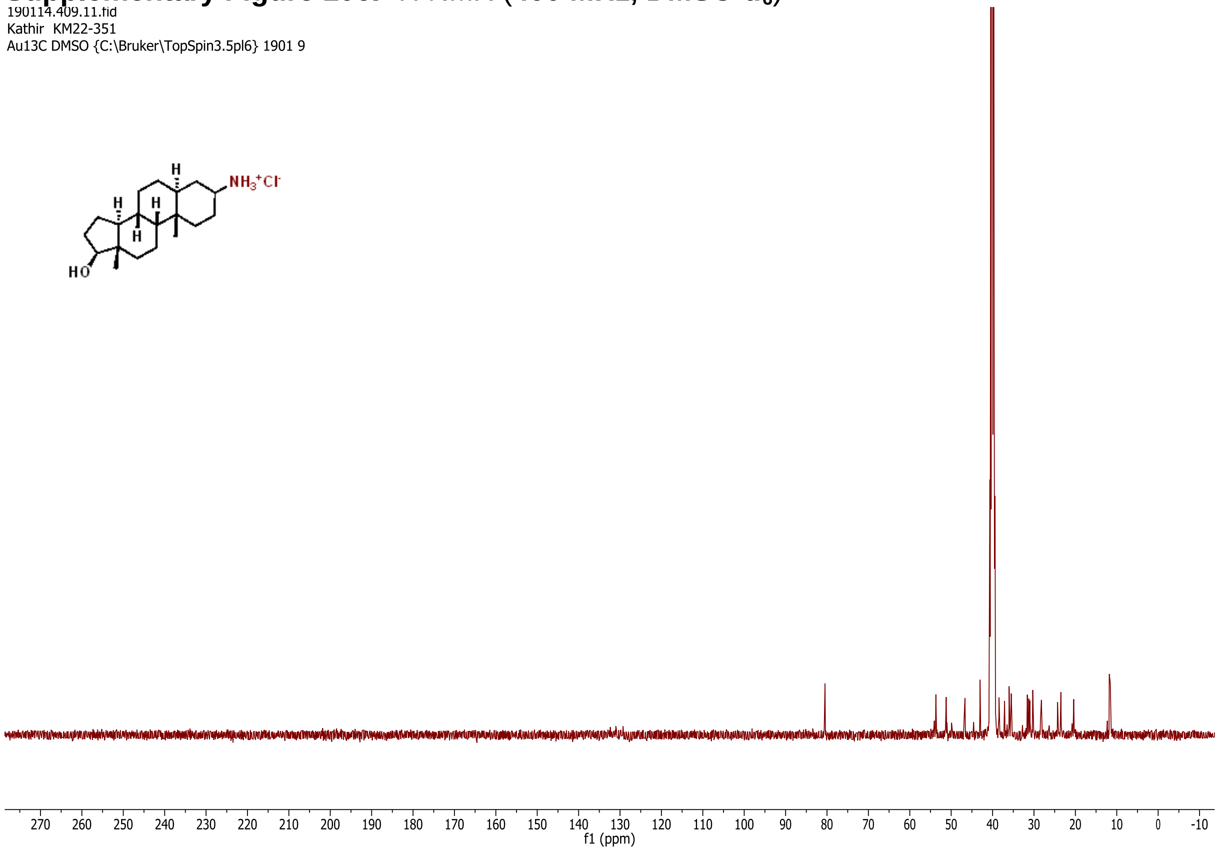

Supplementary Figure 107. ^{13}C NMR (**101 MHz, DMSO-*d*$_6$**)

ESI-TOF Accurate Mass Report Page 1

Results file: E:\Projects\1901.PRO\SampleDB\1901.rpt
Last modified: Monday, January 21, 2019 14:40:22

Sample Summary:

Sample	File	Sample Name	User	Target	Formula	Expected Mass	Observed Mass	Error PPM	Error mDa
188	19012117	KM22-351	Kathir	291.2562	C19H33NO	292.2640	292.2646	2.1	0.6

ESI-TOF Accurate Mass Report Page 2

File:19012117
Vial:1:D,3
Description:MeOH/0.1%HCOOH in H2O 9:1

Sample Name:KM22-351
Date:21-Jan-2019

UserName:Kathir
Time:14:37:48

Sample Report:

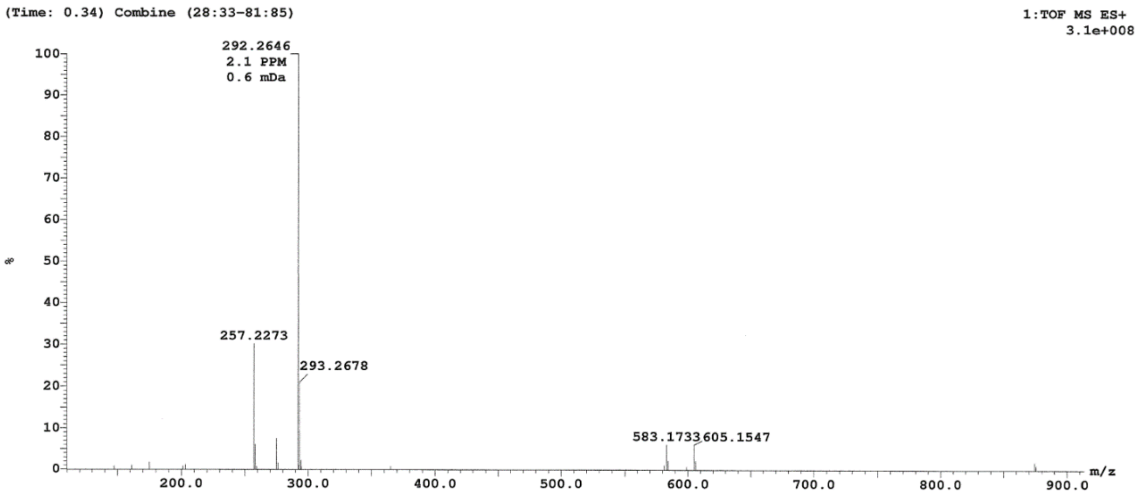

Supplementary Figure 108. HRMS

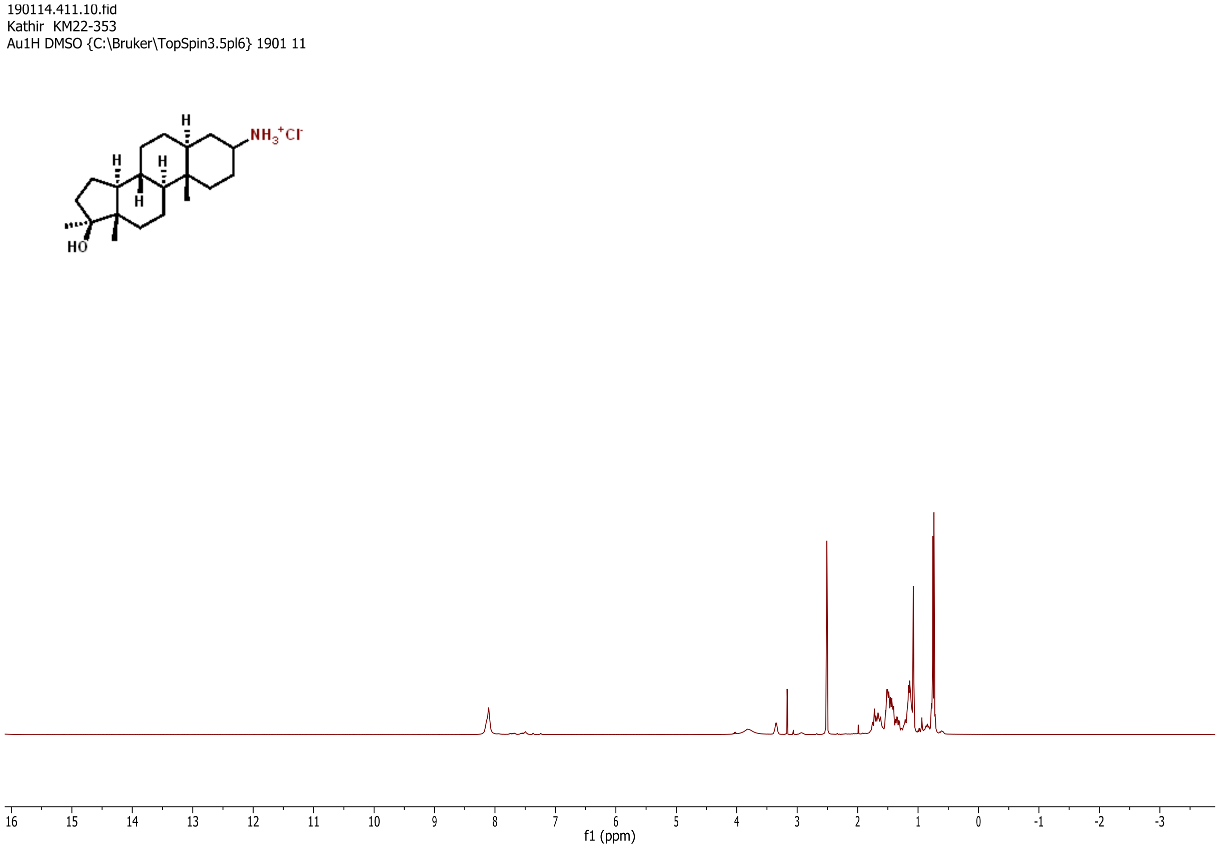

Supplementary Figure 109. ^{1}H NMR (**400 MHz, DMSO-*d*$_6$**)

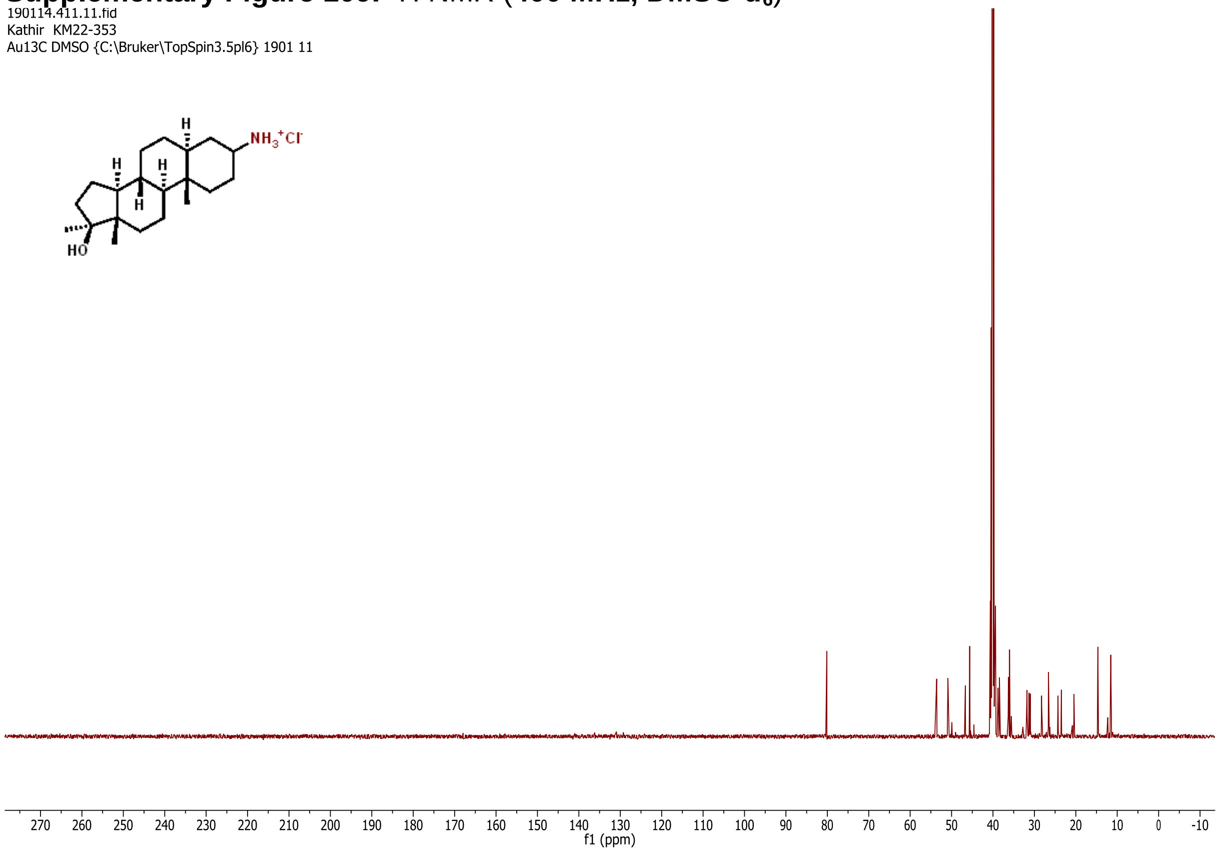

Supplementary Figure 110. ^{13}C NMR (**101 MHz, DMSO-*d*$_6$**)

ESI-TOF Accurate Mass Report Page 1

Results file: E:\Projects\1901.PRO\SampleDB\1901.rpt
Last modified: Monday, January 21, 2019 14:45:30

Sample Summary:

Sample	File	Sample Name	User	Target	Formula	Expected Mass	Observed Mass	Error PPM	Error mDa
190	19012119	KM22-353	Kathir	305.2719	C20H35NO	306.2797	306.2792	-1.6	-0.5

ESI-TOF Accurate Mass Report Page 2

File:19012119
Vial:1:D,5
Description:MeOH/0.1%HCOOH in H2O 9:1

Sample Name:KM22-353
Date:21-Jan-2019

UserName:Kathir
Time:14:42:58

Sample Report:

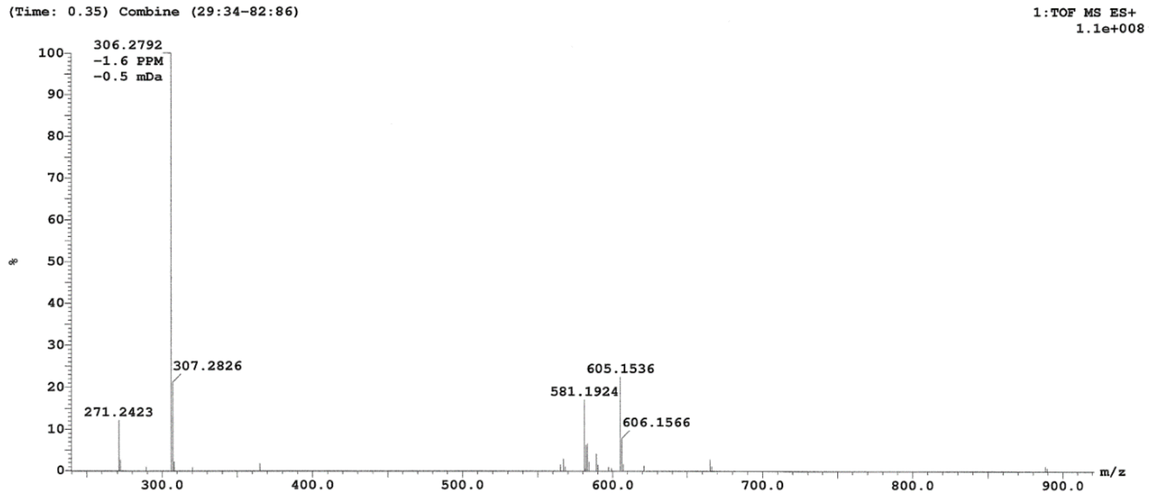

Supplementary Figure 111. HRMS

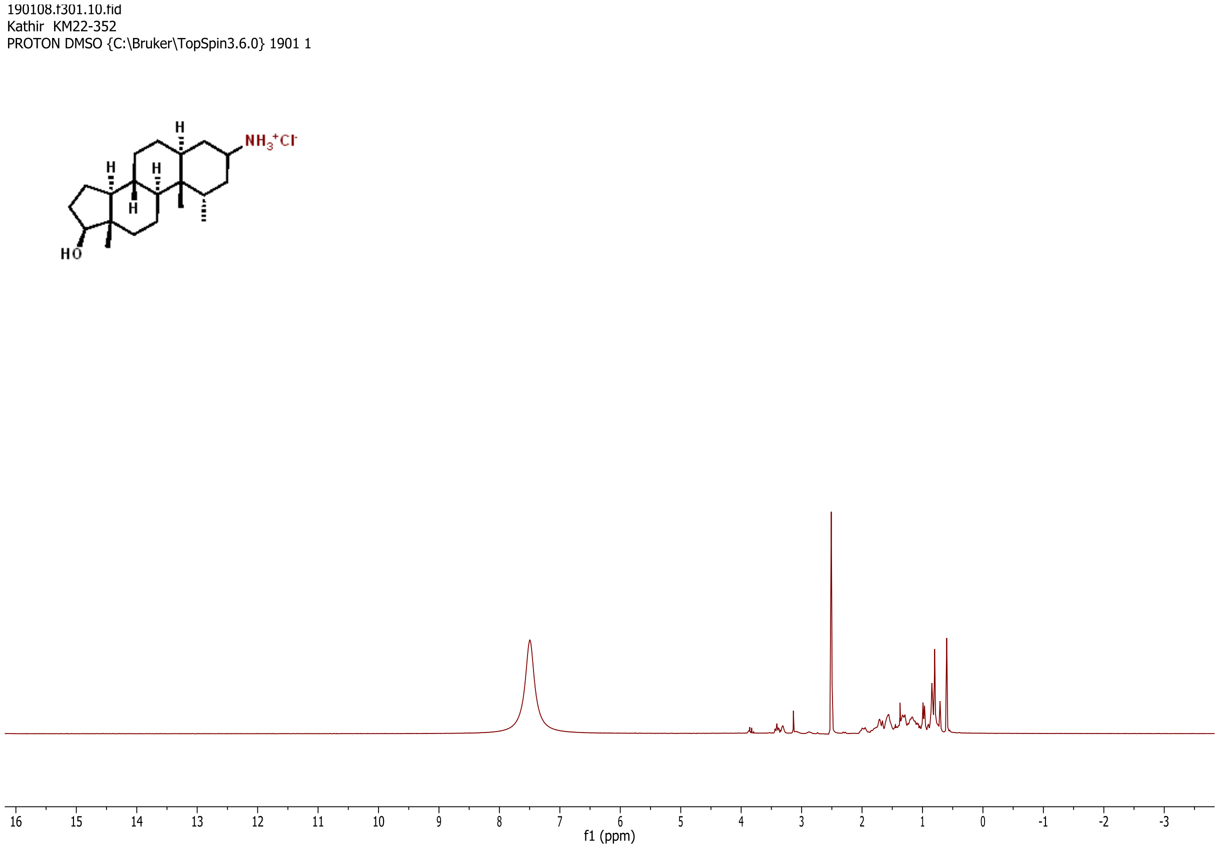

Supplementary Figure 112. ^{1}H NMR (**300 MHz, DMSO-*d*$_6$**)

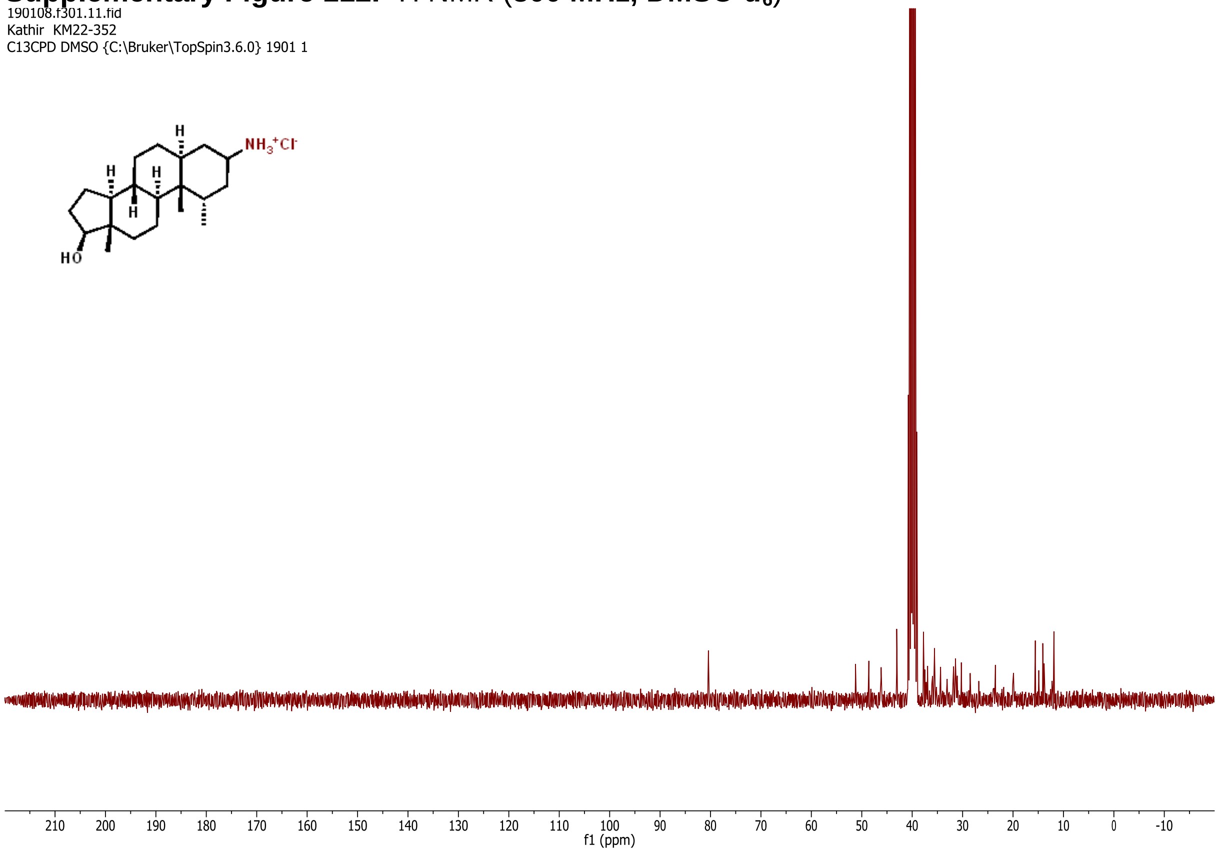

Supplementary Figure 113. ^{13}C NMR (**75 MHz, DMSO-*d*$_6$**)

ESI-TOF Accurate Mass Report Page 1

Results file: E:\Projects\1901.PRO\SampleDB\1901.rpt
Last modified: Monday, January 21, 2019 14:42:56

Sample Summary:

Sample	File	Sample Name	User	Target	Formula	Expected Mass	Observed Mass	Error PPM	Error mDa
189	19012118	KM22-352	Kathir	305.2719	C20H35NO	306.2797	306.2800	1.0	0.3

ESI-TOF Accurate Mass Report Page 2

File:19012118
Vial:1:D,4
Description:MeOH/0.1%HCOOH in H2O 9:1

Sample Name:KM22-352
Date:21-Jan-2019

UserName:Kathir
Time:14:40:24

Sample Report:

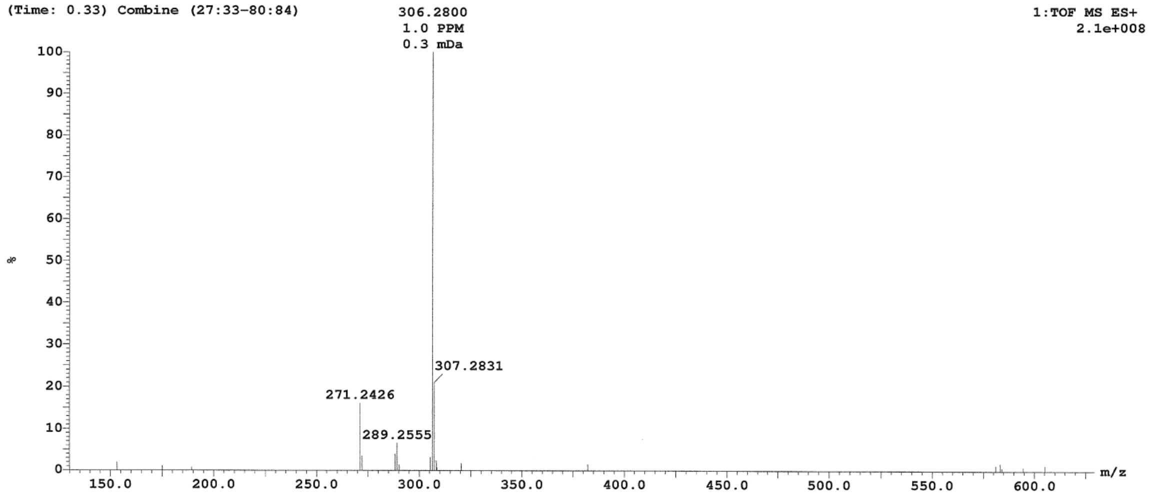

Supplementary Figure 114. HRMS
