# Supplementary material for: Homogeneous cobalt-catalyzed reductive amination for synthesis of functionalized primary amines
Source: Nat Commun. 2019 Nov 29;10:5443. doi: 10.1038/s41467-019-13351-7 (PMC6884468; doi:10.1038/s41467-019-13351-7)
Supplement: Supplementary file 3 — Description of Additional Supplementary Files [file 41467_2019_13351_MOESM3_ESM.pdf]

**Description of Additional Supplementary Files**

File Name: Supplementary Data 1
Description: The Cartesian Coordinates (xyz) for all Optimized structures at B3PW91/TZVP level in gas phase
