# Supplementary material for: Homogeneous cobalt-catalyzed reductive amination for synthesis of functionalized primary amines
Source: Nat Commun. 2019 Nov 29;10:5443. doi: 10.1038/s41467-019-13351-7 (PMC6884468; doi:10.1038/s41467-019-13351-7)
Supplement: Supplementary file 4 — Supplementary Data 1 [file 41467_2019_13351_MOESM4_ESM.pdf]

**H_2**<br>H 0.00000000 0.00000000 0.37259400<br>H 0.00000000 0.00000000 -0.37259400<br>**PhCHNH**<br>C 0.60881300 -2.15990600 0.00000000<br>C 1.63284300 -1.22193000 0.00000000<br>C 1.32784700 0.13275000 0.00000000<br>C 0.00000000 0.56067300 0.00000000<br>C -1.02456500 -0.38999700 0.00000000<br>C -0.71970200 -1.74038600 0.00000000<br>H 0.84298700 -3.21878300 0.00000000<br>H 2.66767800 -1.54536400 0.00000000<br>H 2.12636500 0.86873400 0.00000000<br>H -2.05154700 -0.04408400 0.00000000<br>H -1.51818800 -2.47415200 0.00000000<br>C -0.29502900 2.00066800 0.00000000<br>H 0.59285700 2.64826900 0.00000000<br>N -1.48034500 2.45694800 0.00000000<br>H -1.47898300 3.47551000 0.00000000	**$PhCH_2NH_2$**<br>C 1.40262200 1.33054000 0.05477200<br>C 0.04457500 1.06595800 0.17152600<br>C -0.42755300 -0.24552500 0.13636900<br>C 0.48952300 -1.28283500 -0.01856800<br>C 1.84986600 -1.02096000 -0.13005100<br>C 2.31104700 0.28845200 -0.09277800<br>H 1.75524800 2.35610600 0.08191500<br>H -0.67042400 1.87371900 0.27722900<br>H 0.13426400 -2.30846100 -0.05845300<br>H 2.54892600 -1.84124300 -0.25242300<br>H 3.37139800 0.49647700 -0.18440900<br>C -1.89869000 -0.54405000 0.30514400<br>H -2.08609600 -1.57134400 -0.04310700<br>H -2.13583100 -0.53605300 1.37552900<br>N -2.73979900 0.46299800 -0.33099700<br>H -2.65306800 0.42038500 -1.33959400<br>H -3.71416400 0.31995300 -0.09818200
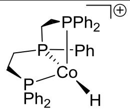<br>***fac*-I**<br>C 2.05060000 0.11528800 -1.98390200<br>H 1.40529800 0.81868300 -2.51685400<br>H 3.05272300 0.23887600 -2.40211100<br>C 1.54993300 -1.31817500 -2.13045000<br>H 2.30641600 -2.02614100 -1.78435800<br>H 1.32927600 -1.56435800 -3.17202000<br>C -1.46512800 -1.35964900 -2.09634600<br>H -1.26603100 -1.57954900 -3.14768300<br>H -2.20279500 -2.08474700 -1.74374000<br>C -2.00981200 0.05779000 -1.92883500<br>H -3.01731300 0.13009800 -2.34499500<br>H -1.37913900 0.78624600 -2.44549500<br>C 2.39465900 2.45654700 -0.28780800	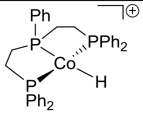<br>***mer*-I**<br>C -0.74434100 -1.53546800 2.67921500<br>H -0.41042300 -1.95780400 3.62865800<br>H -1.77112400 -1.19196300 2.83396800<br>C -0.70156900 -2.57299900 1.55107000<br>H -1.38827700 -3.39966400 1.74415800<br>H 0.30358500 -2.98580600 1.42903400<br>C -0.70156900 -2.57299900 -1.55107000<br>H 0.30358500 -2.98580600 -1.42903400<br>H -1.38827700 -3.39966400 -1.74415800<br>C -0.74434100 -1.53546800 -2.67921500<br>H -1.77112400 -1.19196300 -2.83396800<br>H -0.41042300 -1.95780400 -3.62865800<br>C 1.97359100 -0.36771100 2.62323800

C	3.68604600	2.87697000	-0.61275200
H	4.47822400	2.15179700	-0.76413400
C	3.96754000	4.23098800	-0.72086800
H	4.97250600	4.55382800	-0.96735900
C	2.96566400	5.17094500	-0.50492700
H	3.18968700	6.22840400	-0.58645500
C	1.68232000	4.75580700	-0.17566200
H	0.90209800	5.48656100	0.00310000
C	1.39551400	3.40099400	-0.06258500
H	0.39941900	3.07622800	0.21507200
C	3.40568000	-0.11912300	0.64694500
C	4.43414800	-0.78372300	-0.02075500
H	4.43741200	-0.85940000	-1.10199500
C	5.47946900	-1.35460900	0.69553500
H	6.27527300	-1.86710100	0.16721000
C	5.50741800	-1.26478800	2.08069300
H	6.32551000	-1.70802700	2.63655600
C	4.48582100	-0.60373100	2.75302200
H	4.50568900	-0.52843900	3.83416300
C	3.43721300	-0.03837400	2.04270400
H	2.64542600	0.48070000	2.57340200
C	0.10703800	-3.44548700	-0.76242000
C	-0.32826600	-4.36770400	-1.71653400
H	-0.73783800	-4.03398500	-2.66352800
C	-0.23941900	-5.72922500	-1.46137100
H	-0.58012200	-6.43915200	-2.20626600
C	0.28510300	-6.18141600	-0.25619700
H	0.35256900	-7.24530000	-0.06037300
C	0.72208400	-5.27017500	0.69628400
H	1.13044500	-5.61953700	1.63755700
C	0.63026800	-3.90721500	0.44632700
H	0.97224500	-3.20078900	1.19604700
C	-3.38525800	-0.44484200	0.60020800
C	-3.09848800	-1.56267100	1.38347400
H	-2.06673100	-1.83641600	1.58327900
C	-4.12667400	-2.33082600	1.91482200
H	-3.89465600	-3.19692000	2.52370900
C	-5.44809900	-1.98033900	1.67297000
H	-6.25178600	-2.57499900	2.09166800
C	-5.74140700	-0.86101500	0.90136300
H	-6.77291700	-0.58105800	0.72086200
C	-4.71636300	-0.09310300	0.36841600
H	-4.95500200	0.78917900	-0.21530100
C	-2.54760900	2.26614300	-0.04530000
C	-3.06524700	2.94507400	-1.14878200
H	-3.14290700	2.46056500	-2.11462500
C	-3.49282100	4.26177400	-1.02153300
H	-3.89129700	4.78207800	-1.88491900
C	-3.41438500	4.90495600	0.20591400
H	-3.75279900	5.93002300	0.30396100
C	-2.89988200	4.23280700	1.30925200
H	-2.83551900	4.73182900	2.26934500
C	-2.46190800	2.92299200	1.18450500
C	2.37490200	-1.51741300	3.30342300
H	1.65431000	-2.26635300	3.60886200
C	3.71571500	-1.71842500	3.60882200
H	4.01680100	-2.61496000	4.13857200
C	4.66361000	-0.77269400	3.24343100
H	5.70798400	-0.92876900	3.48784600
C	4.27030400	0.37461300	2.56401800
H	5.00721800	1.11581900	2.27684600
C	2.93545600	0.57331900	2.24569200
H	2.63303700	1.46359700	1.70570600
C	-2.95886700	-1.52704500	0.00000000
C	-3.75746200	-2.67391200	0.00000000
H	-3.30612800	-3.66103700	0.00000000
C	-5.13981700	-2.56146800	0.00000000
H	-5.75358300	-3.45486000	0.00000000
C	-5.73679900	-1.30503200	0.00000000
H	-6.81731400	-1.21996400	0.00000000
C	-4.95053300	-0.16113700	0.00000000
H	-5.41410400	0.81848300	0.00000000
C	-3.56544200	-0.27112400	0.00000000
H	-2.95633600	0.62679400	0.00000000
C	1.97359100	-0.36771100	-2.62323800
C	2.37490200	-1.51741300	-3.30342300
H	1.65431000	-2.26635300	-3.60886200
C	3.71571500	-1.71842500	-3.60882200
H	4.01680100	-2.61496000	-4.13857200
C	4.66361000	-0.77269400	-3.24343100
H	5.70798400	-0.92876900	-3.48784600
C	4.27030400	0.37461300	-2.56401800
H	5.00721800	1.11581900	-2.27684600
C	2.93545600	0.57331900	-2.24569200
H	2.63303700	1.46359700	-1.70570600
Co	0.02187000	0.23920600	0.00000000
P	0.22073300	-0.00143300	2.20393900
P	-1.13137600	-1.64962100	0.00000000
P	0.22073300	-0.00143300	-2.20393900
H	0.79539500	1.54015300	0.00000000
C	-0.32585000	1.28552000	-3.38327800
C	-0.29232900	1.07034500	-4.76266200
C	-0.78563500	2.50288300	-2.88479800
C	-0.72745600	2.06188600	-5.62915000
H	0.08592400	0.13754700	-5.16674600
C	-1.21831100	3.49436900	-3.75696900
H	-0.78485300	2.67783900	-1.81440500
C	-1.19228000	3.27311900	-5.12706800
H	-0.69833500	1.89260600	-6.69937800
H	-1.57078600	4.44159900	-3.36520100
H	-1.52802200	4.04699800	-5.80774400
C	-0.32585000	1.28552000	3.38327800
C	-0.29232900	1.07034500	4.76266200
C	-0.78563500	2.50288300	2.88479800
C	-0.72745600	2.06188600	5.62915000
H	0.08592400	0.13754700	5.16674600

H	-2.04640000	2.40857100	2.04419500
Co	0.03633700	-0.07602400	0.58112700
P	1.98683800	0.67354400	-0.20683500
P	0.05589900	-1.63687400	-1.04911000
P	-1.99973100	0.52070700	-0.12179900
H	0.01233400	1.08694100	1.50538400

C	-1.21831100	3.49436900	3.75696900
H	-0.78485300	2.67783900	1.81440500
C	-1.19228000	3.27311900	5.12706800
H	-0.69833500	1.89260600	6.69937800
H	-1.57078600	4.44159900	3.36520100
H	-1.52802200	4.04699800	5.80774400

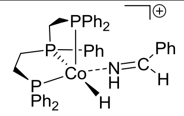

***fac*-II**

C	0.01176400	1.58525600	-2.58014100
H	-1.03552900	1.36032900	-2.79618400
H	0.27524100	2.50770100	-3.10246700
C	0.92162000	0.43939600	-3.00974100
H	1.96636600	0.75779500	-2.98007300
H	0.70823500	0.12478200	-4.03407700
C	-0.54894500	-2.08629900	-2.56779900
H	-0.43692800	-2.14169200	-3.65368200
H	-0.36407000	-3.08963300	-2.17618700
C	-1.94013900	-1.59466300	-2.17942700
H	-2.69914300	-2.34342600	-2.41585100
H	-2.20426400	-0.69447100	-2.73982200
C	-1.01914100	3.12652200	-0.28859800
C	-1.80656900	3.78364200	-1.23266600
H	-1.75955600	3.51316800	-2.28042500
C	-2.66692700	4.80346700	-0.84081000
H	-3.27699200	5.30498200	-1.58329500
C	-2.73953500	5.18185700	0.49192900
H	-3.40554500	5.98198100	0.79403400
C	-1.95422400	4.53273100	1.43890700
H	-2.00606500	4.82472000	2.48169800
C	-1.10492800	3.50699400	1.05282300
H	-0.50442400	2.99717500	1.79864900
C	1.80528700	2.65537500	-0.60603400
C	1.91867000	4.03778600	-0.76936100
H	1.03165600	4.64405800	-0.91174400
C	3.16583000	4.64636500	-0.74120100
H	3.24142800	5.72059800	-0.86618100
C	4.31292000	3.88448800	-0.55064000
H	5.28527200	4.36315900	-0.52879400
C	4.20929100	2.51004100	-0.38518800
H	5.09894800	1.91022600	-0.23121400
C	2.96119900	1.89962600	-0.40848800
H	2.89092100	0.82635700	-0.26664100
C	2.31914300	-1.97248600	-2.18717700
C	2.65237700	-2.37032000	-3.48491200
H	2.01010200	-2.11880100	-4.32211800
C	3.81342000	-3.09249800	-3.71961300
H	4.06402100	-3.39642400	-4.72953600
C	4.65391300	-3.42475200	-2.66271200
H	5.56058700	-3.98892600	-2.84861100

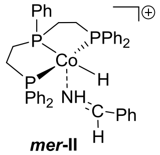

***mer*-II**

C	0.95307100	-2.68289500	1.71443600
H	0.49942800	-3.62096200	2.03892900
H	2.02885500	-2.85859300	1.63830000
C	0.68870300	-1.55251300	2.71879300
H	1.21941300	-1.72776100	3.65690600
H	-0.37710300	-1.46233700	2.94481600
C	0.70482500	1.57139100	2.71310700
H	-0.36060900	1.49218100	2.94527500
H	1.24145500	1.74609700	3.64795300
C	0.97485000	2.69463000	1.70224200
H	2.05191200	2.85912200	1.61922200
H	0.53285200	3.63881800	2.02512200
C	-1.15621100	-3.16829700	-0.30210100
C	-1.97052400	-3.58036100	0.75136600
H	-1.68838500	-3.38447800	1.77969700
C	-3.15653100	-4.26026000	0.50001400
H	-3.77647700	-4.58391900	1.32838700
C	-3.54141700	-4.53101100	-0.80574500
H	-4.46231900	-5.06829700	-1.00081600
C	-2.73901300	-4.11405000	-1.86164800
H	-3.03156900	-4.32631400	-2.88377600
C	-1.55661900	-3.43196700	-1.61331900
H	-0.93492600	-3.11692100	-2.44407000
C	3.05510600	-0.00303000	2.02539100
C	3.66721200	-0.00637300	3.28192800
H	3.06802700	-0.00442100	4.18688700
C	5.05016200	-0.01211800	3.38605900
H	5.51784500	-0.01474100	4.36396800
C	5.83491000	-0.01446200	2.23733100
H	6.91562200	-0.01893100	2.32092800
C	5.23469000	-0.01104200	0.98599300
H	5.84396900	-0.01283500	0.08965100
C	3.84902300	-0.00535600	0.87962000
H	3.38245100	-0.00279100	-0.09925200
C	-1.15155800	3.17432800	-0.29760800
C	-1.95144500	3.59926700	0.76173700
H	-1.65351600	3.41956200	1.78857800
C	-3.14274700	4.27287700	0.51816100
H	-3.75146000	4.60673600	1.35081400
C	-3.54682100	4.52501500	-0.78544700
H	-4.47189100	5.05717500	-0.97471600

C	4.33088700	-3.03253600	-1.37107100	C	-2.75810300	4.09619300	-1.84693500
H	4.98475500	-3.28888700	-0.54528200	H	-3.06577200	4.29391500	-2.86752900
C	3.16822500	-2.30884300	-1.13360700	C	-1.57094000	3.41983700	-1.60638000
H	2.91691700	-2.00454900	-0.12426600	H	-0.96095300	3.09390400	-2.44159700
C	-2.35266400	-2.67527400	0.51643300	Co	0.27098000	0.00445200	-0.11452200
C	-2.63541100	-2.58859600	1.88401300	P	0.39958800	-2.22297900	-0.03217100
H	-2.65527700	-1.61952500	2.37128100	P	1.22820500	0.00497500	1.86359800
C	-2.90972600	-3.72996800	2.62242500	P	0.40740600	2.23176300	-0.03881700
H	-3.14273800	-3.64360500	3.67772600	H	-0.16613800	0.00350900	-1.57519300
C	-2.89192600	-4.97957800	2.01190600	C	1.60776700	3.09252600	-1.12824000
H	-3.10792300	-5.87125600	2.58895200	C	1.96459900	4.42582000	-0.91268800
C	-2.60211400	-5.07824900	0.65875800	C	2.16308000	2.39994700	-2.20212800
H	-2.59100500	-6.04757900	0.17329200	C	2.87492200	5.04791800	-1.75395600
C	-2.33437500	-3.93356000	-0.08560000	H	1.52561100	4.98750200	-0.09522100
H	-2.12746400	-4.04083800	-1.14368100	C	3.07150700	3.02762900	-3.04685300
C	-3.58802800	-0.20462900	-0.21326900	H	1.86627300	1.37194900	-2.38066400
C	-3.66921800	0.87245300	0.66637900	C	3.43043500	4.34913700	-2.82103600
H	-2.78321100	1.19514300	1.20002700	H	3.14790900	6.08272500	-1.58132200
C	-4.87693300	1.53437100	0.85462500	H	3.49605100	2.48385600	-3.88314900
H	-4.92946500	2.37023300	1.54245500	H	4.13894000	4.83967300	-3.47867900
C	-6.00795600	1.13002800	0.15981200	C	1.60823000	-3.08763100	-1.10955600
H	-6.94957400	1.64720100	0.30504500	C	1.96468000	-4.41946600	-0.88446400
C	-5.93391800	0.05598900	-0.72079200	C	2.17032900	-2.40046900	-2.18340000
H	-6.81702500	-0.26690300	-1.26041100	C	2.88125600	-5.04542000	-1.71597500
C	-4.73284000	-0.61332200	-0.90263200	H	1.52047700	-4.97707600	-0.06702200
H	-4.69989000	-1.46319300	-1.57489500	C	3.08512500	-3.03199700	-3.01837500
Co	0.09835500	-0.22519400	0.18246900	H	1.87428600	-1.37361100	-2.36965500
P	0.16974400	1.80286900	-0.73535900	C	3.44362800	-4.35193900	-2.78292200
P	0.79284400	-1.01327900	-1.83755000	H	3.15386400	-6.07904000	-1.53577800
P	-1.98949100	-1.09788900	-0.38173400	H	3.51503200	-2.49239500	-3.85463400
H	-0.46630900	0.50895300	1.34389600	H	4.15715100	-4.84537300	-3.43292400
H	0.08050500	-2.53305100	1.17761600	N	-1.71964700	0.01164700	0.58812400
C	1.31179800	-1.91510500	2.47433500	C	-4.15978600	-0.01132800	0.23235400
N	0.60941500	-1.69970200	1.42155100	C	-4.58210200	0.04525800	1.56540500
C	2.17923800	-0.97989300	3.17123100	C	-5.12372300	-0.06037300	-0.77895600
C	2.93826400	-1.47548900	4.23986200	C	-5.93042800	0.05120400	1.87428500
C	2.27686100	0.37560900	2.84021400	H	-3.85972800	0.08524500	2.37457600
C	3.78202800	-0.64026900	4.95349700	C	-6.47575100	-0.05379500	-0.46975500
H	2.86158700	-2.52410700	4.50958300	H	-4.80443700	-0.10468700	-1.81483900
C	3.11699400	1.20937100	3.55793700	C	-6.88066100	0.00171600	0.85702300
H	1.67032700	0.77283800	2.03493100	H	-6.24823600	0.09475900	2.90956900
C	3.87256900	0.70409200	4.61228300	H	-7.21299400	-0.09210900	-1.26302800
H	4.36594300	-1.03445300	5.77676900	H	-7.93630600	0.00678100	1.10309300
H	3.18578500	2.25878900	3.29634400	C	-2.75282200	-0.02328300	-0.16485700
H	4.52988000	1.36102100	5.17049600	H	-1.97092100	0.05283100	1.57491700
H	1.27530200	-2.91204500	2.91943400	H	-2.55605600	-0.06840100	-1.23507800

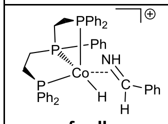

***fac*-IIπ**

C	1.82798000	-1.97325200	-2.07158900	
H	1.90984300	-1.24661100	-2.88392600	
H	2.64949100	-2.68228200	-2.19433700	
C	0.48037800	-2.68422600	-2.12145400	
H	0.48709300	-3.59783700	-1.52318100	
H	0.22607000	-2.98210500	-3.14204400	
C	-1.41813600	-0.51310300	-2.81018500	
H	-1.23195900	-0.98700000	-3.77662700	
H	-2.49871500	-0.38913900	-2.70942600	
C	-0.72676700	0.84258500	-2.71732900	
H	-1.21750500	1.57301000	-3.36444900	
H	0.32349500	0.77472800	-3.01203800	
C	3.40182300	0.18346100	-0.91881800	
C	4.53328900	-0.25580400	-1.61375300	
H	4.62227200	-1.29095200	-1.92399800	
C	5.56506600	0.62565900	-1.89782900	
H	6.43777600	0.27652600	-2.43783700	
C	5.48528100	1.95082300	-1.48162700	
H	6.29591900	2.63657900	-1.70006100	
C	4.37024300	2.39106600	-0.78352700	
H	4.30380000	3.42151500	-0.45470800	
C	3.32952300	1.51145100	-0.50503300	
H	2.45761700	1.86231100	0.03394300	
C	2.86823200	-2.13113400	0.68298500	
C	3.69899700	-1.60319600	1.67563200	
H	3.92216100	-0.54196900	1.68869700	
C	4.27413100	-2.43290200	2.62822600	
H	4.92559200	-2.00976100	3.38441300	
C	4.02463200	-3.79980100	2.60963400	
H	4.47668700	-4.44630600	3.35268100	
C	3.20123800	-4.33442300	1.62827000	
H	3.00895600	-5.40091800	1.60001700	
C	2.62198200	-3.50600800	0.67399600	
H	1.99227500	-3.95220300	-0.08655300	
C	-2.27852300	-2.83667300	-1.26327500	
C	-2.87399900	-3.39479100	-2.39812200	
H	-2.54285400	-3.11627600	-3.39270300	
C	-3.90295800	-4.31553800	-2.26379300	
H	-4.36156800	-4.74331900	-3.14794900	
C	-4.34349300	-4.68855200	-0.99865200	
H	-5.14730200	-5.40864600	-0.89652200	
C	-3.75372300	-4.13844700	0.13111200	
H	-4.09580000	-4.42840900	1.11816400	
C	-2.72422700	-3.21262300	0.00412100	
H	-2.25962400	-2.78019300	0.88399800	
C	-2.54185700	1.81285500	-0.69069800	
C	-3.08557200	2.98565200	-1.22009100	
H	-2.45065900	3.70011400	-1.73212400	
C	-4.44005400	3.25022800	-1.08066500	
H	-4.85447600	4.16370800	-1.49170500	
C	-5.26299500	2.35024800	-0.41183600	
H	-6.32086800	2.56065900	-0.30386400	
C	-4.72736500	1.18661900	0.12212000	

H	-5.36386200	0.48545600	0.64940500
C	-3.37017900	0.92039300	-0.01301600
H	-2.95428700	0.01588000	0.41730600
C	0.06063500	3.03102600	-0.90520800
C	0.55176400	3.65595900	-2.05032700
H	0.48630000	3.17448900	-3.01846700
C	1.13813300	4.91402100	-1.96416700
H	1.51714400	5.39106500	-2.86072200
C	1.23236400	5.55773700	-0.73850300
H	1.68335900	6.54137100	-0.67465300
C	0.74336100	4.93911100	0.40759200
H	0.81087300	5.43956500	1.36680700
C	0.16452800	3.68151900	0.32693000
H	-0.21484000	3.20595400	1.22573600
Co	-0.05515300	-0.35985000	0.24824200
P	2.04269900	-0.97985100	-0.50074900
P	-0.89518600	-1.64360700	-1.41255000
P	-0.76544800	1.39479700	-0.94134600
H	0.62806000	0.70061100	1.02734300
H	0.29643100	-2.37261300	1.65227600
C	0.13862500	-0.61652900	2.44600700
H	1.19866600	-0.62249300	2.71009900
C	-0.68741100	0.32974700	3.22629100
C	-0.07926900	1.42406600	3.84599000
C	-2.04938400	0.10438200	3.42710600
C	-0.82272000	2.28972000	4.63303500
H	0.98503400	1.59294000	3.71226300
C	-2.79270400	0.97279200	4.21262600
H	-2.50709400	-0.77077200	2.98249500
C	-2.18405200	2.06847800	4.81352800
H	-0.34067800	3.13231900	5.11555600
H	-3.84904300	0.78733600	4.37029700
H	-2.76571200	2.74097600	5.43351800
N	-0.39868800	-1.64529000	1.80939800

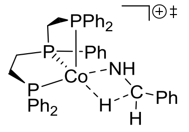

***fac*-TS1**

C	1.81040700	-1.99525700	-2.06702300
H	1.90076300	-1.27181800	-2.88130400
H	2.62459200	-2.71320900	-2.18663600
C	0.45590000	-2.69384800	-2.11857600
H	0.45200100	-3.60472900	-1.51619200
H	0.20245700	-2.99374200	-3.13882900
C	-1.41664500	-0.50310000	-2.81929500
H	-1.23414200	-0.97942900	-3.78525900
H	-2.49559700	-0.36316300	-2.72176000
C	-0.70547400	0.84268000	-2.72338200
H	-1.18160200	1.57974200	-3.37381600
H	0.34510800	0.75827400	-3.01263000

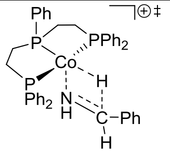

***mer*-TS1**

C	1.63068500	-2.16952200	-1.85005200
H	2.61888300	-2.32648400	-2.28334700
H	1.25597400	-3.13909800	-1.51467800
C	0.66125500	-1.56929200	-2.87350900
H	0.42431400	-2.28481800	-3.66348800
H	1.06023600	-0.65796900	-3.32399700
C	-2.12657600	-0.18272200	-2.81132300
H	-1.65498500	0.67299600	-3.29985400
H	-2.59448800	-0.79847200	-3.58221100
C	-3.14821000	0.24857500	-1.75331200
H	-3.72040000	-0.61939300	-1.41836900
H	-3.85949300	0.97359500	-2.15204400

C	3.40414300	0.14964400	-0.91602100	C	3.49018300	-0.43507200	-0.47825200
C	4.53017000	-0.29400600	-1.61673700	C	3.95833600	-0.02440600	-1.72744000
H	4.60848700	-1.32697300	-1.93691100	H	3.29984100	-0.01976100	-2.58791100
C	5.57016600	0.58009500	-1.89376900	C	5.27308300	0.39097200	-1.88039600
H	6.43863900	0.22781800	-2.43855200	H	5.62966800	0.69549300	-2.85788300
C	5.50431000	1.90171400	-1.46400300	C	6.12752900	0.41725200	-0.78538200
H	6.32161100	2.58137500	-1.67658600	H	7.15656200	0.73582300	-0.90609600
C	4.39467700	2.34630700	-0.76002700	C	5.65808700	0.03790500	0.46480500
H	4.33913400	3.37406600	-0.42097100	H	6.31786700	0.06261900	1.32441000
C	3.34550300	1.47434500	-0.48931600	C	4.34522500	-0.38627000	0.62125900
H	2.47701600	1.82884100	0.05303700	H	3.99826800	-0.69777500	1.59904500
C	2.84873000	-2.15651100	0.68777900	C	-1.61217600	-2.69614700	-1.47106700
C	3.68844700	-1.63489400	1.67617900	C	-2.08041000	-3.52994500	-2.49117600
H	3.92613200	-0.57678400	1.68346900	H	-1.98028500	-3.23949900	-3.53219000
C	4.25459300	-2.46746700	2.63175300	C	-2.67773900	-4.74332300	-2.18387500
H	4.91297000	-2.04917100	3.38463900	H	-3.03790000	-5.38418800	-2.98045100
C	3.98735400	-3.83101100	2.62023400	C	-2.81306300	-5.13644200	-0.85635700
H	4.43239600	-4.47981100	3.36550700	H	-3.28005600	-6.08519900	-0.61831400
C	3.15495900	-4.35944900	1.64307100	C	-2.34949200	-4.31511500	0.16147300
H	2.94855500	-5.42344000	1.62040800	H	-2.45154700	-4.61950400	1.19654000
C	2.58432700	-3.52809600	0.68624000	C	-1.75034400	-3.09868000	-0.14406100
H	1.94645600	-3.96953800	-0.07025800	H	-1.38578300	-2.46424100	0.65566200
C	-2.30829900	-2.81562700	-1.26453100	C	-2.56796800	2.75241200	-0.34573700
C	-2.92430300	-3.35823100	-2.39582200	C	-2.59409500	3.39048200	-1.58716100
H	-2.60301300	-3.07547100	-3.39245800	H	-2.53074600	2.81933700	-2.50696900
C	-3.96130000	-4.26892200	-2.25474100	C	-2.72592400	4.77162700	-1.66551000
H	-4.43626300	-4.68488500	-3.13588500	H	-2.75168700	5.25474400	-2.63547400
C	-4.38891100	-4.64700600	-0.98668900	C	-2.83221900	5.52783600	-0.50628200
H	-5.19913200	-5.35911600	-0.87939900	H	-2.93859400	6.60453900	-0.56812600
C	-3.77840800	-4.11218400	0.13949500	C	-2.80618300	4.89978500	0.73340800
H	-4.11080100	-4.40602600	1.12869100	H	-2.89497700	5.48506200	1.64137200
C	-2.74093200	-3.19636100	0.00606900	C	-2.66871600	3.52144900	0.81623300
H	-2.26112200	-2.77466400	0.88320200	H	-2.65631400	3.04242700	1.78888300
C	-2.51724300	1.83635600	-0.70600600	P	1.77683100	-1.08688500	-0.31119900
C	-3.04672800	3.00963400	-1.24869000	P	-0.81522200	-1.09343200	-1.88124400
H	-2.40265300	3.71162300	-1.76661300	P	-2.30356500	0.93553100	-0.21645400
C	-4.39827900	3.29112200	-1.11449500	C	-3.38788100	0.43170700	1.17229100
H	-4.80162600	4.20485200	-1.53586700	C	-4.77511600	0.58461100	1.10665500
C	-5.23245900	2.40771100	-0.43742100	C	-2.81045700	-0.09988900	2.32353400
H	-6.28798300	2.63141900	-0.33327400	C	-5.56842000	0.19870400	2.17667500
C	-4.71102600	1.24386300	0.10984000	H	-5.24026300	1.01553700	0.22682000
H	-5.35618300	0.55589800	0.64390400	C	-3.60750500	-0.47996200	3.39698500
C	-3.35680000	0.96048300	-0.02050400	H	-1.73239500	-0.20907400	2.37922000
H	-2.95249500	0.05541100	0.41980500	C	-4.98567600	-0.33389000	3.32224800
C	0.09996800	3.02296700	-0.90842600	H	-6.64423500	0.31854700	2.12006400
C	0.62626300	3.63079700	-2.04712800	H	-3.15124300	-0.88873300	4.29125400
H	0.57583200	3.14202500	-3.01251900	H	-5.60900800	-0.63053300	4.15796300
C	1.22820800	4.88122500	-1.95811900	C	1.91121000	-2.28155500	1.07831600
H	1.63372200	5.34533600	-2.84986000	C	2.60214400	-3.48973300	0.95918800
C	1.30441900	5.53398000	-0.73596700	C	1.32631700	-1.94980500	2.29992300
H	1.76781600	6.51169600	-0.67010900	C	2.68939900	-4.35394800	2.04098800
C	0.78086000	4.93227000	0.40382100	H	3.08566300	-3.75862400	0.02668900
H	0.83370800	5.44002400	1.36011400	C	1.42358000	-2.81207100	3.38560900

C	0.18543900	3.68262400	0.32035500
H	-0.22280900	3.22183000	1.21421600
Co	-0.07934300	-0.37613800	0.24676700
P	2.03347100	-1.00039900	-0.49847000
P	-0.91305800	-1.63913600	-1.42002300
P	-0.74499100	1.39635700	-0.94795500
H	0.58687000	0.63002700	1.12600700
H	0.29751700	-2.35279200	1.68292500
C	0.14602100	-0.55940000	2.41530000
H	1.19937100	-0.58034300	2.70560600
C	-0.68868100	0.36964400	3.21774200
C	-0.09523100	1.47637900	3.82835100
C	-2.04082500	0.11348000	3.43961000
C	-0.84464800	2.32339300	4.63021300
H	0.96293400	1.66986000	3.67853200
C	-2.79066200	0.96352900	4.23991800
H	-2.48532400	-0.77090400	2.99993700
C	-2.19740100	2.07129700	4.83320000
H	-0.37348400	3.17560800	5.10660100
H	-3.83983700	0.75368300	4.41425400
H	-2.78349300	2.72932600	5.46442400
N	-0.39417800	-1.61633400	1.80564200

H	0.80377300	-1.00394300	2.40134200
C	2.10029000	-4.01707200	3.25498900
H	3.22585300	-5.29039500	1.93911400
H	0.97252000	-2.54124500	4.33352100
H	2.17557800	-4.69254400	4.09944100
Co	-0.12859500	0.22192000	-0.27480100
H	0.29690300	1.18070200	0.84636100
H	-0.18050600	2.12321900	-1.95486600
C	0.71814200	2.25172400	-0.25340300
H	-0.07329900	2.93643400	0.06509600
C	2.02592700	2.62037800	0.37361000
C	2.15392100	2.73412600	1.75820400
C	3.07351100	3.03905600	-0.44109100
C	3.31099500	3.25019800	2.31935500
H	1.33870700	2.41608500	2.40173100
C	4.23258800	3.55972300	0.12127800
H	2.95827400	2.96904800	-1.51548300
C	4.35467800	3.66770100	1.49946900
H	3.39884300	3.33487300	3.39670800
H	5.03985000	3.88962800	-0.52258700
H	5.25686000	4.08071500	1.93625800
N	0.65452700	1.75312600	-1.50524500

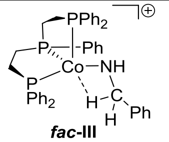

***fac*-III**

C	1.84055700	-1.81277000	-2.13368000
H	1.99189100	-1.02617300	-2.87726000
H	2.63355500	-2.54763200	-2.28902000
C	0.46854600	-2.45887900	-2.30744600
H	0.39410000	-3.39327200	-1.74693000
H	0.27665500	-2.70347900	-3.35572000
C	-1.26231200	-0.13328200	-2.98101700
H	-1.04060600	-0.53873700	-3.97086200
H	-2.33682600	0.05928100	-2.93726100
C	-0.49014600	1.15905600	-2.72725200
H	-0.88819700	1.97398700	-3.33562600
H	0.56891200	1.04160600	-2.97007800
C	3.52300800	0.09703800	-0.75048000
C	4.67077900	-0.39791100	-1.37760000
H	4.70943600	-1.42468000	-1.72417600
C	5.78132100	0.41476000	-1.54574300
H	6.66626800	0.02286200	-2.03411100
C	5.76488500	1.72522100	-1.07806200
H	6.63730900	2.35603700	-1.20473200
C	4.63418900	2.22008000	-0.44511800
H	4.61713600	3.23862500	-0.07549400
C	3.51517100	1.40954600	-0.28386000
H	2.63292200	1.80480900	0.20575100
C	2.73882100	-2.27187600	0.63357800
C	3.46033800	-1.86978600	1.76172300

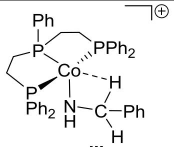

***mer*-III**

C	1.57872400	-2.21478700	-1.86265600
H	2.56261600	-2.37292200	-2.30537100
H	1.19418100	-3.18650700	-1.54526300
C	0.61226800	-1.57829900	-2.86562200
H	0.35199700	-2.27456300	-3.66553000
H	1.02578300	-0.66741300	-3.30333200
C	-2.13110800	-0.13866700	-2.79728800
H	-1.63095900	0.70265200	-3.28147700
H	-2.60720400	-0.74487800	-3.57082800
C	-3.14889000	0.32280400	-1.74982500
H	-3.75063400	-0.52671400	-1.41939400
H	-3.83295200	1.07066100	-2.15398700
C	3.46144100	-0.51612700	-0.46981500
C	3.91429700	-0.05953100	-1.70866400
H	3.24433500	-0.01958800	-2.55900800
C	5.22674300	0.36287100	-1.86021300
H	5.57206600	0.70411400	-2.82956600
C	6.09249700	0.35391000	-0.77384000
H	7.11891100	0.68101700	-0.89382100
C	5.63768900	-0.07015900	0.46719600
H	6.30601100	-0.07226700	1.32051400
C	4.32775200	-0.50388200	0.62200200
H	3.99189500	-0.85067900	1.59182300
C	-1.68070500	-2.66860600	-1.45786300

H	3.67062900	-0.81812900	1.92557500	C	-2.16774900	-3.48562500	-2.48282100
C	3.94753500	-2.80853100	2.66011000	H	-2.06152900	-3.19187900	-3.52230000
H	4.51576200	-2.48071500	3.52317800	C	-2.79196400	-4.68701200	-2.18206800
C	3.71566800	-4.16296700	2.45149900	H	-3.16668100	-5.31506400	-2.98211100
H	4.09810600	-4.89570700	3.15241000	C	-2.93573300	-5.08439900	-0.85672100
C	3.00007000	-4.57272900	1.33526200	H	-3.42405000	-6.02365700	-0.62388400
H	2.82261300	-5.62766600	1.15946200	C	-2.45340200	-4.27957200	0.16557600
C	2.51040300	-3.63404400	0.43370200	H	-2.56200200	-4.58769800	1.19886600
H	1.96543300	-3.98406300	-0.43465700	C	-1.82709000	-3.07527100	-0.13326200
C	-2.36077300	-2.51361600	-1.66279200	H	-1.44708200	-2.45317900	0.66878100
C	-2.96760100	-2.89969500	-2.86113000	C	-2.49074000	2.80769000	-0.33992400
H	-2.60866300	-2.52419300	-3.81332600	C	-2.45571500	3.44621600	-1.58102000
C	-4.04527400	-3.77321700	-2.84334300	H	-2.38335500	2.87256000	-2.49841500
H	-4.51413200	-4.06872400	-3.77489900	C	-2.53578600	4.83108200	-1.66158200
C	-4.52169300	-4.26757700	-1.63413100	H	-2.51484700	5.31500000	-2.63127500
H	-5.36389900	-4.94996700	-1.62362700	C	-2.64901300	5.59081400	-0.50510000
C	-3.92023300	-3.88608800	-0.44237000	H	-2.71419900	6.67068000	-0.56892300
H	-4.29276900	-4.26965100	0.50071500	C	-2.68238000	4.96229000	0.73407300
C	-2.84264400	-3.00836700	-0.45075100	H	-2.77624800	5.55037100	1.63971300
H	-2.36840400	-2.69892500	0.47542800	C	-2.59787600	3.57977900	0.81931900
C	-2.35942100	2.07577900	-0.71316800	H	-2.63284200	3.10113100	1.79155700
C	-2.84603900	3.23039900	-1.33185900	P	1.74845200	-1.16566700	-0.30671200
H	-2.18078900	3.86721500	-1.90515500	P	-0.84888900	-1.08284000	-1.86260900
C	-4.18072600	3.58167100	-1.19961500	P	-2.29643300	0.98294500	-0.20868500
H	-4.55163200	4.47924200	-1.68118600	C	-3.41593600	0.52195000	1.16858900
C	-5.03953300	2.78966600	-0.44367100	C	-4.79242000	0.75193900	1.10046300
H	-6.08144500	3.06948900	-0.33825200	C	-2.87462800	-0.05775100	2.31396500
C	-4.56022500	1.64760100	0.18155300	C	-5.61043800	0.39442900	2.16177200
H	-5.22343100	1.03379200	0.77970600	H	-5.22899500	1.22048700	0.22517600
C	-3.22340200	1.29041800	0.04750600	C	-3.69535100	-0.40859700	3.37946400
H	-2.85492000	0.40005500	0.54685500	H	-1.80474800	-0.23029100	2.37127600
C	0.31296500	3.13799300	-0.69113600	C	-5.06301000	-0.18566300	3.30175600
C	1.00628700	3.77033900	-1.72188600	H	-6.67783800	0.57376800	2.10295100
H	1.04398700	3.33765800	-2.71409700	H	-3.26615400	-0.85481000	4.26922300
C	1.66149300	4.97446500	-1.48852100	H	-5.70544000	-0.45948900	4.13075000
H	2.19420000	5.46001800	-2.29817700	C	1.87863700	-2.38680600	1.05981100
C	1.62959400	5.55436400	-0.22821700	C	2.56960900	-3.59266500	0.91944600
H	2.13597200	6.49611000	-0.05043200	C	1.28834800	-2.08061100	2.28545200
C	0.94124900	4.92724600	0.80511900	C	2.65199600	-4.47880300	1.98380900
H	0.90833200	5.37918700	1.78984100	H	3.05676400	-3.84246900	-0.01650200
C	0.28850800	3.72526400	0.57692400	C	1.38082600	-2.96420400	3.35416500
H	-0.25749000	3.25140700	1.38663300	H	0.76292800	-1.13829700	2.40630300
Co	-0.16815400	-0.37116400	0.16143800	C	2.05802200	-4.16642000	3.20192600
P	2.04819800	-0.96936800	-0.47765400	H	3.18835100	-5.41330600	1.86505700
P	-0.91187200	-1.39912700	-1.65316900	H	0.92541300	-2.71273500	4.30530100
P	-0.60285900	1.56483500	-0.91715900	H	2.12944000	-4.85886400	4.03283100
H	0.29442100	0.30505500	1.63468600	Co	-0.13031100	0.20707400	-0.28641400
H	0.10988400	-2.47680200	1.48607400	H	0.42314900	1.35170500	0.72961700
C	-0.00842100	-0.65108900	2.32334000	H	-0.01549500	2.14411800	-1.94930300
H	0.96736900	-0.85631900	2.77895800	C	0.78185600	2.18372600	-0.15314200
C	-0.96092100	-0.04097300	3.32894900	H	0.04410300	2.96527200	0.06406600
C	-0.56244500	1.06537100	4.07904300	C	2.11591400	2.60704500	0.41744700
C	-2.21704800	-0.59443500	3.54780200	C	2.31275400	2.67103600	1.79572900

C	-1.41134500	1.61377200	5.02916400
H	0.42410300	1.49579700	3.92906600
C	-3.06738400	-0.04542700	4.50066400
H	-2.50916000	-1.46587600	2.97478100
C	-2.66963400	1.05999300	5.24027000
H	-1.09043800	2.47049600	5.61096000
H	-4.04164700	-0.48960800	4.67194600
H	-3.33196300	1.48473200	5.98581300
N	-0.50991700	-1.67307200	1.49114300

C	3.10423600	3.08803500	-0.43347400
C	3.47908900	3.21036300	2.31771600
H	1.54422400	2.30166000	2.46988300
C	4.27219600	3.63037800	0.08865800
H	2.94124100	3.03654400	-1.50308800
C	4.46201000	3.69614100	1.46248500
H	3.62076900	3.25719300	3.39169600
H	5.03563400	4.00752800	-0.58250500
H	5.37160400	4.12496200	1.86746200
N	0.71735300	1.65200800	-1.44417100

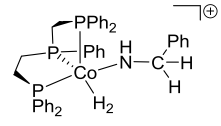

***fac*-IV**

C	1.59193900	-1.97414800	-2.07254100
H	2.27354800	-1.18952300	-2.40800000
H	2.03006300	-2.93698600	-2.34318300
C	0.21256000	-1.80049700	-2.69341500
H	-0.40058300	-2.68603400	-2.50959100
H	0.26182000	-1.65758000	-3.77542500
C	-0.22731000	1.13980800	-2.86610100
H	-0.15636200	0.89875100	-3.93001200
H	-1.06333400	1.83123000	-2.74108000
C	1.06443600	1.77443400	-2.36037800
H	1.20100600	2.76624200	-2.79705600
H	1.93412000	1.18313600	-2.65539500
C	3.12555000	-1.97650800	0.43912000
C	4.26396100	-1.83135000	-0.35271200
H	4.18237200	-1.63087600	-1.41384800
C	5.52944500	-1.95248300	0.20955200
H	6.40648100	-1.84633200	-0.41848100
C	5.67018300	-2.21121000	1.56562600
H	6.65786700	-2.30960300	2.00107800
C	4.53949100	-2.35231900	2.36294100
H	4.64223300	-2.56274300	3.42134400
C	3.27549400	-2.23662900	1.80429600
H	2.40192100	-2.37404500	2.43324900
C	0.66334800	-3.43215700	0.26320700
C	1.31359500	-4.63840100	-0.01139700
H	2.28408800	-4.64120500	-0.49647500
C	0.73277400	-5.84274700	0.35551800
H	1.24273200	-6.77461600	0.13955300
C	-0.49659900	-5.85492200	1.00746900
H	-0.94572700	-6.79782500	1.29771700
C	-1.14239600	-4.66011500	1.29160600
H	-2.09606300	-4.66603700	1.80710800
C	-0.56542500	-3.45005000	0.92050900
H	-1.07060300	-2.51360700	1.14107700
C	-2.44717700	-0.69493400	-2.32806900
C	-3.09956300	-0.03483800	-3.37164500
H	-2.59055000	0.71851000	-3.96018200

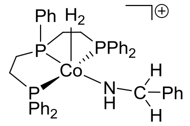

***mer*-IV**

C	-2.78515000	-1.88741900	1.16129300
H	-3.71600100	-1.77614600	1.71963800
H	-2.96075300	-2.58479400	0.33809400
C	-1.65776900	-2.40322300	2.04581900
H	-1.85455400	-3.41812600	2.39691400
H	-1.51385600	-1.76344900	2.92063800
C	1.34545300	-2.57105600	2.11339200
H	1.23149700	-1.91632100	2.98112700
H	1.40850400	-3.60114600	2.46991200
C	2.56776900	-2.18519900	1.28899800
H	2.71207500	-2.90716800	0.48131100
H	3.47678200	-2.16041800	1.89201100
C	-2.99787200	0.95692700	1.61161100
C	-2.23918900	1.30509700	2.72992300
H	-1.21748600	0.95400800	2.81213600
C	-2.77004500	2.13979400	3.70384100
H	-2.17363500	2.40636500	4.56910400
C	-4.05615000	2.64590600	3.56227800
H	-4.46748100	3.30428700	4.31878000
C	-4.80928500	2.31584000	2.44292100
H	-5.80870600	2.71783200	2.32171200
C	-4.28641900	1.47251300	1.47105300
H	-4.88218900	1.22876400	0.59958200
C	-0.18480400	-3.84564200	-0.00570300
C	-0.31073600	-5.09498600	0.61070700
H	-0.37638800	-5.17446800	1.69108000
C	-0.35377200	-6.25314400	-0.15078000
H	-0.45262700	-7.21567800	0.33788300
C	-0.27106900	-6.17907700	-1.53728200
H	-0.30608600	-7.08502800	-2.13126800
C	-0.14406200	-4.94459900	-2.15730700
H	-0.07923000	-4.88154700	-3.23742100
C	-0.10080700	-3.78252100	-1.39530500
H	-0.00026200	-2.82565600	-1.89118800
C	3.05199500	0.65368700	1.69943000
C	2.33916700	1.04567900	2.83490100
H	1.30457800	0.74494100	2.94985200

C	-4.41755100	-0.34496900	-3.67909100	C	2.93745800	1.85042100	3.79473900
H	-4.91364700	0.17188600	-4.49247500	H	2.37639600	2.15102700	4.67221900
C	-5.09617200	-1.31303500	-2.95060300	C	4.24752000	2.27987500	3.62441000
H	-6.12561400	-1.55110100	-3.19189500	H	4.71262900	2.91348100	4.37081700
C	-4.45416500	-1.97325300	-1.91075100	C	4.95752800	1.90400100	2.49124600
H	-4.98138200	-2.72447600	-1.33452500	H	5.97725600	2.24360500	2.35064600
C	-3.13791800	-1.66595700	-1.59756800	C	4.36627900	1.09246000	1.53198000
H	-2.65075500	-2.17486900	-0.77394200	H	4.92944100	0.80681200	0.65170200
C	0.21891700	3.39271500	-0.05487000	Co	0.01740400	-0.24094800	0.18297000
C	0.30718700	3.83491900	1.26799000	P	-2.29450300	-0.24364700	0.40401400
H	0.91192800	3.28929200	1.98522900	P	-0.12462400	-2.31372800	1.01645400
C	-0.35053200	4.98602900	1.67379100	P	2.26685300	-0.51187400	0.50933600
H	-0.26383500	5.32080200	2.70113900	H	-0.36222400	-0.58994000	-1.56581800
C	-1.11797100	5.70827400	0.76670800	C	3.35702600	-0.46727800	-0.96650700
H	-1.63322200	6.60745800	1.08381600	C	4.37725300	-1.39675800	-1.17589200
C	-1.21432500	5.27620600	-0.54805100	C	3.16701500	0.55931800	-1.89485500
H	-1.80380700	5.83803100	-1.26376900	C	5.18769700	-1.30481600	-2.30037600
C	-0.54914000	4.12539500	-0.95759000	H	4.55881400	-2.19422000	-0.46579900
H	-0.62911900	3.82295600	-1.99468800	C	3.98603900	0.65277700	-3.01143800
C	2.86753400	2.36075500	-0.16912200	H	2.38364400	1.29421400	-1.74149400
C	3.41052300	3.49978000	-0.77117500	C	4.99386700	-0.28142900	-3.21871100
H	2.81323100	4.09989600	-1.44879100	H	5.97586600	-2.03292700	-2.45416500
C	4.71012000	3.88907900	-0.48669300	H	3.83387100	1.45765700	-3.72114300
H	5.12332000	4.77295900	-0.95916900	H	5.62968000	-0.21004700	-4.09366100
C	5.47692900	3.15605400	0.41395900	C	-3.37913900	-0.13344900	-1.07883300
H	6.48968300	3.46809600	0.64216500	C	-4.57783800	-0.84674200	-1.17027500
C	4.94078800	2.03236100	1.02526000	C	-3.00288100	0.69166800	-2.13992700
H	5.53110100	1.46199400	1.73274800	C	-5.37752500	-0.73621600	-2.30003100
C	3.64076200	1.63380800	0.73260400	H	-4.90388200	-1.49162700	-0.36309400
H	3.23195800	0.75096000	1.20945500	C	-3.80867600	0.80672800	-3.26504900
Co	-0.01387500	-0.11041400	0.19841700	H	-2.08048400	1.25772000	-2.09261300
P	1.42031100	-1.82490700	-0.22934600	C	-4.99523300	0.09058300	-3.34883900
P	-0.69580000	-0.37867900	-1.90180000	H	-6.30352300	-1.29680500	-2.35796100
P	1.12868900	1.85091900	-0.49974800	H	-3.50461100	1.45674700	-4.07732500
N	-1.72170600	0.28523100	0.75735200	H	-5.62203600	0.17702500	-4.22887200
H	-2.47393300	0.34847300	0.08032100	N	0.22331200	1.58776400	0.36065800
H	0.35000800	-0.41666500	1.79527100	H	1.16332300	1.92329400	0.53510400
H	0.58054300	0.35426800	1.68528700	H	0.41943000	-0.57506200	-1.55866200
C	-2.09979200	1.07017500	1.92953200	C	-0.59638700	2.73586300	0.02061200
H	-1.42930700	0.82082500	2.75844000	H	-1.62062500	2.40970200	-0.17848300
H	-1.98103000	2.14663100	1.74770900	H	-0.67060800	3.41050200	0.88527800
C	-3.52694800	0.79894100	2.33772500	C	-0.10512200	3.55568400	-1.15700700
C	-3.85300800	-0.34690600	3.06398700	C	0.18184900	2.96288700	-2.38753800
C	-4.55042500	1.66982300	1.96912700	C	0.04815600	4.93546000	-1.03877800
C	-5.16848800	-0.61432400	3.41568000	C	0.59787200	3.72618300	-3.47001000
H	-3.06462900	-1.02904200	3.36674300	H	0.08382900	1.88805900	-2.49578000
C	-5.87019200	1.40340300	2.31482600	C	0.46751400	5.70492900	-2.11820800
H	-4.31037900	2.57214400	1.41480600	H	-0.16538200	5.41540500	-0.08860100
C	-6.18187500	0.26055500	3.03924400	C	0.74250900	5.10301900	-3.33838200
H	-5.40517700	-1.50145600	3.99285100	H	0.80658800	3.24819700	-4.42130700
H	-6.65448100	2.09445400	2.02653300	H	0.58117100	6.77718400	-2.00374300
H	-7.20927700	0.05559400	3.31798500	H	1.06806600	5.70125700	-4.18172000

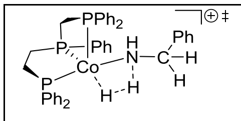

***fac*-TS2**

C	-1.74056500	-1.95925500	1.99552200
H	-2.41623600	-1.15512300	2.29559500
H	-2.23923200	-2.90707300	2.20769100
C	-0.41117800	-1.86163900	2.73421800
H	0.16751300	-2.77738400	2.59205200
H	-0.54797900	-1.72852700	3.80987100
C	0.24373400	1.03740000	3.03176200
H	0.14638200	0.78146300	4.09006000
H	1.11633500	1.68600400	2.92937800
C	-1.00655100	1.74401800	2.51367500
H	-1.09971600	2.73354600	2.96598100
H	-1.90735600	1.18822700	2.78442800
C	-3.08371600	-1.82733100	-0.61994200
C	-4.27382200	-1.78332800	0.10403700
H	-4.26721600	-1.70319600	1.18406400
C	-5.49647000	-1.84934100	-0.55454700
H	-6.41575300	-1.81966600	0.01900900
C	-5.54054600	-1.95569900	-1.93714100
H	-6.49494600	-2.01230000	-2.44781800
C	-4.35644300	-1.99507100	-2.66591900
H	-4.38458900	-2.08311400	-3.74600900
C	-3.13538000	-1.92995600	-2.01256800
H	-2.21777000	-1.97330700	-2.59036200
C	-0.70506000	-3.39295000	-0.31846200
C	-1.38326200	-4.58541200	-0.05307600
H	-2.35244600	-4.56944500	0.43428900
C	-0.83465400	-5.80087000	-0.43344700
H	-1.36751300	-6.72128400	-0.22375900
C	0.39045500	-5.83888900	-1.09196700
H	0.81368300	-6.79006800	-1.39376400
C	1.06388100	-4.65810600	-1.37062500
H	2.01232500	-4.68180500	-1.89515400
C	0.51863700	-3.43844400	-0.98403500
H	1.04748600	-2.51794800	-1.21074500
C	2.34612400	-0.94576800	2.48967700
C	3.08327900	-0.27600300	3.46705400
H	2.65929100	0.55885000	4.01092600
C	4.37733800	-0.68148400	3.76828300
H	4.93875900	-0.15612800	4.53226200
C	4.94875200	-1.75409900	3.09761500
H	5.95923200	-2.06651300	3.33399800
C	4.22155500	-2.42693100	2.12322600
H	4.66227600	-3.26472700	1.59552700
C	2.92949200	-2.02425800	1.81780600
H	2.37222400	-2.55960900	1.05565700
C	-0.13668800	3.41939600	0.26088000
C	-0.29593000	3.96255200	-1.01659700

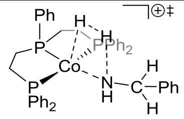

***mer*-TS2**

C	0.03197000	2.73652500	1.62059200
H	0.72274900	3.55703400	1.82066700
H	-0.97851300	3.10615300	1.81031000
C	0.32771700	1.52295400	2.50607600
H	0.08564300	1.72128000	3.55203200
H	1.36762600	1.20285400	2.41408700
C	-0.23577700	-1.50821100	2.53452300
H	0.84719700	-1.60152400	2.43732600
H	-0.50416800	-1.58809500	3.58976200
C	-0.97259000	-2.55030200	1.68929600
H	-2.04709400	-2.49923100	1.87979700
H	-0.65630500	-3.56392600	1.93592300
C	1.63163400	2.99685500	-0.85566900
C	2.76390900	3.14694400	-0.05371700
H	2.74670800	2.86478800	0.99257100
C	3.93921400	3.65639200	-0.59168500
H	4.81202400	3.76868600	0.04072100
C	3.99557600	4.01463700	-1.93152200
H	4.91258400	4.41425000	-2.34874300
C	2.87317000	3.86026300	-2.73651200
H	2.91067400	4.14253400	-3.78243300
C	1.69746500	3.35072700	-2.20484700
H	0.82484900	3.24858800	-2.84024000
C	-2.39223900	0.46321100	2.35342600
C	-2.73496100	0.40286400	3.70790100
H	-1.99085100	0.14781200	4.45560000
C	-4.03369900	0.67233400	4.11291900
H	-4.29173100	0.62230100	5.16450700
C	-5.00160200	1.00760600	3.17177300
H	-6.01600400	1.21761200	3.49073900
C	-4.66830400	1.07328700	1.82628200
H	-5.42024200	1.33519100	1.09111200
C	-3.36821900	0.80109000	1.41755600
H	-3.11382800	0.85273700	0.36523400
C	0.29585100	-3.50882200	-0.85180800
C	0.97181900	-4.45578100	-0.08606600
H	0.90731500	-4.45293300	0.99510900
C	1.75803900	-5.42287200	-0.70171500
H	2.28012500	-6.15356300	-0.09485600
C	1.87550400	-5.45230300	-2.08377900
H	2.48857200	-6.20780900	-2.56116600
C	1.20382700	-4.50967500	-2.85407900
H	1.28901100	-4.52846800	-3.93449200
C	0.42219000	-3.54161600	-2.24282800
H	-0.10104300	-2.81494800	-2.85572100
Co	-0.08838600	-0.01038600	-0.28370500
P	0.07785700	2.26904900	-0.19517500

H	-0.95433300	3.48316500	-1.73324000	P	-0.66980800	0.13401600	1.81867000
C	0.36330200	5.12893400	-1.37381300	P	-0.76906800	-2.18805200	-0.13998800
H	0.22234900	5.54318300	-2.36561200	H	0.39624300	-0.13470200	-1.83539600
C	1.19807800	5.76745800	-0.46370500	C	-2.41287800	-2.58068000	-0.86289200
H	1.71248900	6.67947000	-0.74328100	C	-3.02109600	-3.81814900	-0.63859800
C	1.36292300	5.23538100	0.80703200	C	-3.05239200	-1.64171600	-1.66776700
H	2.00488700	5.73168200	1.52604400	C	-4.25762200	-4.09783900	-1.20032200
C	0.70049000	4.06721700	1.16763300	H	-2.52669200	-4.57261100	-0.03622600
H	0.83847300	3.68279600	2.17093700	C	-4.28859900	-1.92592800	-2.23601200
C	-2.75973700	2.30616500	0.26104700	H	-2.57734500	-0.68433000	-1.85516500
C	-3.44592900	3.26615800	1.01043800	C	-4.89275200	-3.15257000	-1.99953000
H	-2.96999300	3.74886000	1.85675400	H	-4.72476900	-5.05943700	-1.02121600
C	-4.73911400	3.62876600	0.66567800	H	-4.77731500	-1.19086500	-2.86521600
H	-5.26450500	4.37267500	1.25375400	H	-5.85647400	-3.37758600	-2.44164200
C	-5.35504100	3.05089700	-0.43992100	C	-1.23767800	3.27398600	-0.98555900
H	-6.36307500	3.34249000	-0.71192100	C	-1.44323100	4.61188400	-0.64122200
C	-4.67519600	2.10750500	-1.19638400	C	-2.02673600	2.69587400	-1.97860400
H	-5.14866000	1.65682300	-2.06068200	C	-2.43372700	5.34942900	-1.27318500
C	-3.38317900	1.73283500	-0.84489800	H	-0.82614600	5.08750300	0.11299900
H	-2.86039600	0.98854600	-1.43378800	C	-3.01231500	3.43917400	-2.61730300
Co	0.06994200	-0.09778600	-0.07340800	H	-1.86026600	1.66162600	-2.26250900
P	-1.42736600	-1.76822500	0.17318000	C	-3.21897900	4.76470400	-2.26113500
P	0.63292900	-0.48152400	2.03806400	H	-2.58900700	6.38665900	-0.99935400
P	-1.02125300	1.84729900	0.64890000	H	-3.61644700	2.98326500	-3.39335400
N	1.82189400	0.31741700	-0.74407300	H	-3.98830200	5.34637500	-2.75577600
H	2.56816200	-0.17522700	-0.26925500	N	1.90170100	-0.16214500	0.31633700
H	0.63485000	-0.06401200	-1.55976200	H	2.45781000	0.61078800	-0.03696500
H	-0.31064300	0.07104800	-1.63142300	H	1.10130200	-0.19214300	-1.31010300
C	2.33859100	1.42754900	-1.53813200	C	2.74921300	-1.35180800	0.24886000
H	1.54436400	1.77687400	-2.20664200	H	2.32327700	-2.13476800	0.88759400
H	2.58833600	2.28252000	-0.89769500	H	2.81946100	-1.80136700	-0.75522000
C	3.55459600	1.03997800	-2.34372500	C	4.15029300	-1.04983200	0.72285000
C	3.43152600	0.23505700	-3.47733900	C	5.19485200	-0.90913900	-0.18846200
C	4.82528600	1.46033100	-1.95836200	C	4.42288900	-0.86635100	2.07941100
C	4.55013900	-0.13823300	-4.20776100	C	6.47953300	-0.59358100	0.24057800
H	2.44722200	-0.09264500	-3.79808900	H	5.00155500	-1.05697400	-1.24668200
C	5.94999100	1.08627600	-2.68569300	C	5.70326400	-0.55464600	2.51394500
H	4.93543100	2.09527000	-1.08448500	H	3.62107700	-0.97679100	2.80310100
C	5.81451400	0.28572200	-3.81133100	C	6.73671600	-0.41508600	1.59300900
H	4.43792200	-0.75348200	-5.09367500	H	7.28151700	-0.49427500	-0.48261700
H	6.93176400	1.42650300	-2.37577900	H	5.90078200	-0.42817300	3.57291000
H	6.68890900	-0.00153500	-4.38413900	H	7.73876300	-0.17629400	1.93100200

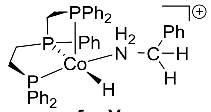

***fac*-V**

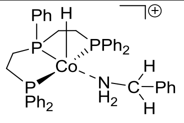

***mer*-V**

C	2.25730500	1.60106100	1.75290000	C	0.91734300	-2.62615100	1.73622100
H	2.66013600	0.65821000	2.13155500	H	0.51573900	-3.59256800	2.04305300
H	3.00786500	2.37522700	1.92584900	H	2.00551400	-2.72552900	1.74326700
C	0.94528300	1.95400400	2.44203200	C	0.49999200	-1.51085100	2.70575700
H	0.67784600	2.99121400	2.22855300	H	0.96506300	-1.64929100	3.68387800

H	1.01730500	1.85827300	3.52802900	H	-0.58273800	-1.47994300	2.84064200
C	-0.59189200	-0.51941500	2.98804500	C	0.44025800	1.64053700	2.64733600
H	-0.47674300	-0.17585800	4.01940700	H	-0.64320000	1.57991600	2.77166300
H	-1.60477800	-0.91642100	2.88686800	H	0.88690200	1.82941800	3.62554500
C	0.43217200	-1.59894900	2.64683600	C	0.83007300	2.73366900	1.64245400
H	0.21962800	-2.52187300	3.19051000	H	1.91511500	2.86301100	1.64500900
H	1.43686700	-1.28701000	2.94306900	H	0.40070700	3.69928200	1.91318800
C	3.55650100	0.88621800	-0.78529400	C	-1.01844000	-3.18744500	-0.47374000
C	4.70846700	0.72314900	-0.01801600	C	-1.68293100	-4.02651400	0.41937100
H	4.68183800	0.83733700	1.05866000	H	-1.34826200	-4.12888500	1.44460300
C	5.91821700	0.40981400	-0.62793700	C	-2.78921800	-4.76095500	0.00441300
H	6.80767900	0.28528700	-0.02094900	H	-3.29258800	-5.41467600	0.70758400
C	5.98806100	0.26145000	-2.00547200	C	-3.24269900	-4.66275900	-1.30349000
H	6.93348000	0.02371100	-2.47944300	H	-4.09923700	-5.24287300	-1.62698600
C	4.84150400	0.42139500	-2.77682400	C	-2.59256900	-3.81891500	-2.19773900
H	4.89016000	0.30869100	-3.85395500	H	-2.94163800	-3.73686700	-3.22075700
C	3.63267800	0.72761400	-2.17101800	C	-1.49264000	-3.08082000	-1.78535900
H	2.74217200	0.84694400	-2.77904800	H	-0.98783200	-2.42625600	-2.48862000
C	1.69913600	3.06289400	-0.70484000	C	2.84442200	0.10113800	2.13933800
C	2.63731700	4.06810100	-0.46223200	C	3.38047800	0.15979800	3.42909700
H	3.53609600	3.85437800	0.10623200	H	2.72896500	0.18394900	4.29701500
C	2.44197600	5.34531400	-0.96708900	C	4.75468700	0.18830000	3.61541600
H	3.17596800	6.11907900	-0.77291900	H	5.16224100	0.23410900	4.61883100
C	1.31432500	5.63073600	-1.72982000	C	5.60727500	0.15789800	2.51662600
H	1.16807600	6.62786200	-2.12892900	H	6.68088500	0.18008800	2.66422400
C	0.38321300	4.63428100	-1.98799700	C	5.08330900	0.09908300	1.23291000
H	-0.48996800	4.84898000	-2.59376500	H	5.74519800	0.07489100	0.37502900
C	0.57571800	3.35658100	-1.47494400	C	3.70660800	0.07108900	1.04441500
H	-0.14697700	2.57416400	-1.68514300	H	3.30158700	0.02546100	0.03933800
C	-1.92595200	1.97107400	2.22626700	C	-1.16340300	3.12613200	-0.53055000
C	-2.86661100	1.63908700	3.20118900	C	-1.90265800	3.86075800	0.39565600
H	-2.77323100	0.72745900	3.77830800	H	-1.57774500	3.94756000	1.42566100
C	-3.94028000	2.48384600	3.45962700	C	-3.07202200	4.50650000	0.00799100
H	-4.66176700	2.21406900	4.22237200	H	-3.63354100	5.08109100	0.73585900
C	-4.08638700	3.66792100	2.75112500	C	-3.51484300	4.42177300	-1.30402200
H	-4.92306900	4.32542200	2.95659800	H	-4.42373800	4.92961200	-1.60493500
C	-3.15353000	4.00783800	1.77801500	C	-2.78888400	3.68230000	-2.23135100
H	-3.25897100	4.93258400	1.22220500	H	-3.13039800	3.61110200	-3.25764400
C	-2.08524300	3.16358000	1.51308200	C	-1.62540000	3.03253400	-1.84755700
H	-1.36723300	3.44042000	0.74690000	H	-1.06579400	2.45374200	-2.57475800
C	-0.77377400	-3.14430500	0.44662300	Co	0.30042400	0.01023600	-0.19784600
C	-0.79636400	-3.69760600	-0.83853700	P	0.48837100	-2.20805900	-0.05165100
H	-0.04227500	-3.41023900	-1.56485200	P	1.02710400	0.06044600	1.87305200
C	-1.74973300	-4.64470700	-1.18475300	P	0.40268800	2.24070500	-0.12485800
H	-1.74247000	-5.07551900	-2.17958800	H	0.05124800	-0.01518300	-1.70354700
C	-2.70426500	-5.04700600	-0.25688100	C	1.65645100	3.12340400	-1.13440900
H	-3.44492600	-5.79163400	-0.52455700	C	1.94525300	4.47253900	-0.91672200
C	-2.69530600	-4.49883600	1.01810900	C	2.32396000	2.43267600	-2.14349900
H	-3.43036300	-4.81331800	1.75049800	C	2.89876600	5.11434700	-1.69256100
C	-1.73801700	-3.55188700	1.36789500	H	1.42033100	5.03160900	-0.14958500
H	-1.74590200	-3.15565400	2.37650900	C	3.27624300	3.08010200	-2.92215500
C	2.06364600	-2.85859500	0.58359400	H	2.08171200	1.39101100	-2.32483100
C	2.42729400	-3.86802800	1.47951400	C	3.56636100	4.41821500	-2.69509600

H	1.81167200	-4.08752600	2.34470100
C	3.57592900	-4.61430600	1.26149200
H	3.85237500	-5.39384300	1.96236900
C	4.36430800	-4.37150300	0.14136000
H	5.25811900	-4.96073900	-0.02924400
C	4.00281000	-3.37825700	-0.75737900
H	4.61274100	-3.18567600	-1.63223700
C	2.85837000	-2.62084900	-0.53572900
H	2.58279700	-1.83765100	-1.23192900
Co	0.09099800	0.12833500	-0.24213900
P	1.93218100	1.34766000	-0.06166400
P	-0.47791600	0.91521100	1.80694900
P	0.52440000	-1.87707400	0.80338300
N	-1.69291300	-0.27266400	-1.19970100
H	-1.62272400	0.17662300	-2.10966600
H	-1.65130900	-1.26805000	-1.40913500
H	0.71898900	-0.33576600	-1.51229800
C	-3.03935600	0.02841100	-0.64031500
H	-3.16383200	-0.58746600	0.25193300
H	-3.04308200	1.07019500	-0.32021800
C	-4.15226700	-0.22452800	-1.62153100
C	-4.68169600	-1.50482500	-1.78568200
C	-4.64615300	0.81382100	-2.41075500
C	-5.67680300	-1.74236500	-2.72414300
H	-4.31861000	-2.32160000	-1.16908700
C	-5.64139000	0.57850100	-3.35066200
H	-4.25855500	1.81993200	-2.27990200
C	-6.15725500	-0.70127400	-3.50963900
H	-6.08503500	-2.74022300	-2.83795700
H	-6.02057200	1.39604700	-3.95317500
H	-6.93766700	-0.88612700	-4.23877500

H	3.11826100	6.16169500	-1.51952200
H	3.78917500	2.53851200	-3.70885700
H	4.30869100	4.92345800	-3.30231500
C	1.80208900	-3.06113900	-1.01262800
C	2.19687200	-4.36527800	-0.70649200
C	2.39973500	-2.39693300	-2.08180500
C	3.18505900	-4.98799400	-1.45415900
H	1.72916600	-4.90596600	0.10917000
C	3.38653400	-3.02572300	-2.83234100
H	2.07705800	-1.39171200	-2.33128900
C	3.78215600	-4.31818600	-2.51706700
H	3.48666400	-6.00052900	-1.21147900
H	3.84466000	-2.50494400	-3.66556700
H	4.55181500	-4.80860700	-3.10199300
N	-1.92098200	-0.02510400	0.04764300
H	-2.21589300	0.87897900	-0.31340000
H	-2.15163200	-0.68811100	-0.68700200
C	-2.74315000	-0.34337200	1.23601700
H	-2.50199700	-1.36674800	1.53182800
H	-2.43315700	0.31527000	2.05029600
C	-4.22892000	-0.20855900	1.01873300
C	-4.92933400	-1.18131800	0.30407300
C	-4.92380800	0.89557400	1.50768800
C	-6.29165600	-1.04665900	0.07653100
H	-4.40625200	-2.05638100	-0.07006400
C	-6.28852800	1.03124500	1.28428300
H	-4.39485400	1.65543300	2.07465400
C	-6.97456400	0.06080200	0.56653600
H	-6.82547600	-1.81187400	-0.47565700
H	-6.81744400	1.89282200	1.67579900
H	-8.03983400	0.16285700	0.39414100

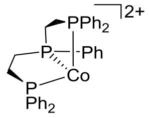

***fac*-L**

C	-0.17673400	2.06941100	2.08119900
H	-0.91207600	2.57196300	1.44978400
H	-0.25212400	2.50510700	3.07967400
C	1.23512000	2.22420600	1.52263600
H	1.97399000	1.87278000	2.24650400
H	1.46403200	3.26937200	1.30104700
C	1.23512000	2.22420600	-1.52263600
H	1.46403200	3.26937200	-1.30104700
H	1.97399000	1.87278000	-2.24650400
C	-0.17673400	2.06941100	-2.08119900
H	-0.25212400	2.50510700	-3.07967400
H	-0.91207600	2.57196300	-1.44978400
C	-2.35625900	0.08082700	2.58020200
C	-3.31135300	1.02822000	2.19664300
H	-3.02667200	1.92893800	1.66556700
C	-4.64437800	0.83724800	2.52691700
H	-5.37916500	1.58459000	2.25170900

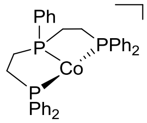

***mer*-L**

P	2.24841500	-0.23614300	-0.02259200
P	-2.20525400	0.63004000	-0.10669700
C	2.50052900	-1.29527900	-1.53423900
C	-2.73175800	0.02932300	-1.78563100
H	2.40362900	-2.34418400	-1.24669400
H	-3.22114800	-0.93188600	-1.60912300
H	3.51018300	-1.15029900	-1.92185400
H	-3.48406900	0.69186600	-2.21833100
C	1.44731900	-0.92406900	-2.57856400
C	-1.52090400	-0.14388300	-2.70451500
H	1.46493700	-1.60666000	-3.43116500
H	-1.77488500	-0.72032000	-3.59694300
H	1.59788900	0.09294300	-2.95127600
H	-1.11949200	0.81790500	-3.03397900
Co	-0.01809300	0.05904400	0.16309800
C	3.10869600	-1.00720400	1.38462000
C	3.73654700	-0.19368200	2.33280500

C	-5.03709700	-0.30501300	3.21603900
H	-6.07988000	-0.44946900	3.47310300
C	-4.09346800	-1.25777500	3.58269100
H	-4.39821700	-2.14494500	4.12504900
C	-2.75678000	-1.07238900	3.26496000
H	-2.02609700	-1.81118100	3.57410000
C	3.25326800	0.69177000	0.00000000
C	4.24538400	1.67820900	0.00000000
H	3.98940800	2.73253200	0.00000000
C	5.58174600	1.30737700	0.00000000
H	6.35026700	2.07129800	0.00000000
C	5.93373700	-0.03808700	0.00000000
H	6.97961400	-0.32173300	0.00000000
C	4.95013400	-1.01843700	0.00000000
H	5.22685800	-2.06608800	0.00000000
C	3.61002800	-0.65631000	0.00000000
H	2.84877400	-1.42922900	0.00000000
C	-2.35625900	0.08082700	-2.58020200
C	-3.31135300	1.02822000	-2.19664300
H	-3.02667200	1.92893800	-1.66556700
C	-4.64437800	0.83724800	-2.52691700
H	-5.37916500	1.58459000	-2.25170900
C	-5.03709700	-0.30501300	-3.21603900
H	-6.07988000	-0.44946900	-3.47310300
C	-4.09346800	-1.25777500	-3.58269100
H	-4.39821700	-2.14494500	-4.12504900
C	-2.75678000	-1.07238900	-3.26496000
H	-2.02609700	-1.81118100	-3.57410000
Co	-0.02771100	-0.40998200	0.00000000
P	-0.60315500	0.26533900	2.13454400
P	1.50513300	1.18429700	0.00000000
P	-0.60315500	0.26533900	-2.13454400
C	0.39221900	-0.47734700	-3.46338900
C	0.33653700	0.03952800	-4.76177800
C	1.19749300	-1.58449400	-3.19164800
C	1.09196200	-0.54365400	-5.76745200
H	-0.30381400	0.88260300	-4.99750900
C	1.94711400	-2.16795200	-4.20440300
H	1.23525000	-2.00525200	-2.19108700
C	1.89612500	-1.64497800	-5.48967200
H	1.04822200	-0.14297500	-6.77333900
H	2.56611600	-3.03152500	-3.99214900
H	2.47999500	-2.09954000	-6.28145100
C	0.39221900	-0.47734700	3.46338900
C	0.33653700	0.03952800	4.76177800
C	1.19749300	-1.58449400	3.19164800
C	1.09196200	-0.54365400	5.76745200
H	-0.30381400	0.88260300	4.99750900
C	1.94711400	-2.16795200	4.20440300
H	1.23525000	-2.00525200	2.19108700
C	1.89612500	-1.64497800	5.48967200
H	1.04822200	-0.14297500	6.77333900
H	2.56611600	-3.03152500	3.99214900
C	3.07105500	-2.39275200	1.57363000
C	4.34258100	-0.76668500	3.44166400
H	3.77447400	0.88091800	2.19711900
C	3.68455600	-2.95584700	2.68275200
H	2.58513300	-3.04486500	0.85643400
C	4.31855600	-2.14475700	3.61746500
H	4.84016600	-0.13430700	4.16738900
H	3.67202000	-4.03115300	2.81621100
H	4.79859700	-2.58889400	4.48151400
C	3.07803500	1.34223600	-0.40090700
C	2.34905100	2.53199300	-0.41470100
C	4.44432400	1.36179700	-0.70038000
C	2.97641300	3.73047200	-0.73133800
H	1.28919800	2.53871500	-0.17359400
C	5.06480900	2.56118800	-1.01355200
H	5.03044800	0.44938500	-0.67446200
C	4.33175000	3.74401200	-1.03129800
H	2.40812900	4.65276100	-0.73945200
H	6.12397600	2.57478500	-1.24222600
H	4.82228900	4.67884600	-1.27609300
C	-1.98974800	2.43973800	-0.16506900
C	-1.52974400	3.05774500	1.00778700
C	-2.21349600	3.21006300	-1.30804700
C	-1.32255300	4.42903700	1.03689600
H	-1.38815200	2.47937400	1.91689900
C	-2.00014200	4.58199000	-1.27097200
H	-2.58134500	2.76098000	-2.22307500
C	-1.55475400	5.19075900	-0.10330300
H	-0.99035100	4.90476800	1.95217300
H	-2.19419400	5.17889400	-2.15443700
H	-1.40237700	6.26346000	-0.07760700
C	-3.52548500	0.25209800	1.08193400
C	-3.56099900	-1.01353900	1.67456400
C	-4.51851700	1.19108500	1.37201900
C	-4.59158800	-1.33908500	2.54379400
H	-2.79347700	-1.74891600	1.45642400
C	-5.54409800	0.85536500	2.24405200
H	-4.49638000	2.17706100	0.92296600
C	-5.58083300	-0.40502700	2.82925300
H	-4.62098200	-2.31976800	3.00362000
H	-6.31680800	1.58138600	2.46761900
H	-6.38295000	-0.65982200	3.51194600
P	-0.19462300	-0.98569900	-1.73371500
C	-0.65101600	-2.73327700	-1.52699700
C	-0.97394400	-3.50629200	-2.64693200
C	-0.64903500	-3.31591700	-0.25797300
C	-1.29481000	-4.84663500	-2.48919100
H	-0.97640800	-3.07768200	-3.64372200
C	-0.96887200	-4.65858700	-0.10812300
H	-0.39213600	-2.72865500	0.61934300
C	-1.29318300	-5.42164600	-1.22286300
H	-1.54653800	-5.44399500	-3.35754000
H	-0.96619400	-5.10909100	0.87743300

H	2.47999500	-2.09954000	6.28145100

H	-1.54521500	-6.46911800	-1.10594400

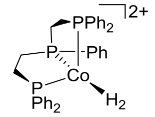

***fac*-I'**

C	-1.98229000	0.49349300	2.09911000
H	-1.28196400	1.21210000	2.53203600
H	-2.95751600	0.70601900	2.54272400
C	-1.54918200	-0.94407100	2.34971100
H	-2.35291500	-1.64374800	2.11212800
H	-1.27434000	-1.10834200	3.39425400
C	1.45217000	-1.37949700	2.24312900
H	1.24422500	-1.58653900	3.29493200
H	2.08477200	-2.18418400	1.86240600
C	2.15221300	-0.03830900	2.06304200
H	3.16543900	-0.06175500	2.47035400
H	1.60773900	0.76287300	2.56761700
C	-2.32994800	2.62932000	0.08297900
C	-3.58018200	3.09570900	-0.32708900
H	-4.37800000	2.40237800	-0.56401400
C	-3.80291400	4.46236400	-0.42917200
H	-4.77470800	4.82420700	-0.74357300
C	-2.78685600	5.36136300	-0.13169400
H	-2.96656800	6.42682500	-0.21441600
C	-1.53771500	4.89776900	0.26768100
H	-0.74423500	5.60010400	0.49360200
C	-1.30432900	3.53524700	0.37044100
H	-0.32145600	3.18575100	0.67176600
C	-3.41295500	-0.06556900	-0.44660800
C	-4.51004400	-0.46863200	0.31776000
H	-4.55871800	-0.26205600	1.38041900
C	-5.57194100	-1.12808400	-0.28741300
H	-6.42281300	-1.43469900	0.30955200
C	-5.54930300	-1.38373800	-1.65297100
H	-6.38339000	-1.89282500	-2.12157200
C	-4.46264500	-0.97950900	-2.42039700
H	-4.44881300	-1.16879300	-3.48732000
C	-3.39447000	-0.32820100	-1.82092000
H	-2.56083000	-0.00337400	-2.43612900
C	-0.39068200	-3.15675100	0.74252200
C	0.23066900	-4.22582700	1.39331200
H	0.90964900	-4.06380700	2.22168100
C	-0.03702800	-5.52668200	0.99053800
H	0.43847400	-6.35429700	1.50346800
C	-0.91138500	-5.76770100	-0.06233000
H	-1.11371700	-6.78570000	-0.37398600
C	-1.53023800	-4.70662300	-0.71301600
H	-2.21368100	-4.89506700	-1.53240000
C	-1.27348900	-3.40328200	-0.31459000
H	-1.77528100	-2.58475700	-0.82126100
C	3.36744200	-0.81636900	-0.51527300
C	2.89446700	-1.79114700	-1.39551100

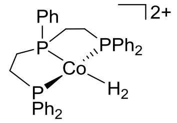

***mer*-I'**

P	2.28223200	0.13667000	-0.04170600
P	-2.25210000	0.27120800	-0.06844800
C	2.69375200	-0.74047100	-1.63281400
C	-2.68188200	-0.44569100	-1.73222000
H	2.80232400	-1.80610600	-1.42145900
H	-2.93980400	-1.49036200	-1.54217700
H	3.65291100	-0.37878800	-2.00682900
H	-3.57694600	0.03530800	-2.13187500
C	1.57120500	-0.50164400	-2.63909300
C	-1.49836700	-0.34946900	-2.69329900
H	1.68749100	-1.12505300	-3.52826100
H	-1.65063300	-0.96887900	-3.57981900
H	1.53408300	0.54366000	-2.95729800
H	-1.32205300	0.67603600	-3.02684400
Co	0.00502200	0.05691900	0.22846100
C	3.34234500	-0.54628700	1.27157800
C	3.88581500	0.30621400	2.23611000
C	3.55083100	-1.92578400	1.36422400
C	4.64920500	-0.21911800	3.26916700
H	3.73418400	1.37763300	2.17394300
C	4.31919400	-2.44015600	2.39852000
H	3.13257800	-2.60798500	0.63235300
C	4.86750900	-1.58898100	3.35089300
H	5.08027300	0.44550100	4.00855300
H	4.49379200	-3.50790400	2.45891900
H	5.46960800	-1.99402700	4.15568400
C	2.74818300	1.87597500	-0.32328000
C	1.79090100	2.88550500	-0.21242200
C	4.06054900	2.20128300	-0.68169500
C	2.13714400	4.20717200	-0.46278900
H	0.76849400	2.65581700	0.07666000
C	4.40127600	3.52292600	-0.92498000
H	4.82292900	1.43307300	-0.75456500
C	3.44100200	4.52446000	-0.81860600
H	1.39097200	4.98760900	-0.37432400
H	5.42006700	3.77372600	-1.19606500
H	3.71379400	5.55572000	-1.00975700
C	-2.54443600	2.07389800	-0.15374200
C	-2.45191000	2.80869300	1.03490000
C	-2.85192100	2.73220900	-1.34624500
C	-2.68094700	4.17564000	1.02810300
H	-2.24793900	2.31155500	1.97822000
C	-3.07336300	4.10389300	-1.34643000
H	-2.94929900	2.18962400	-2.27873800
C	-2.98709000	4.82558900	-0.16337000
H	-2.62985100	4.73425500	1.95535300
H	-3.32584600	4.60580400	-2.27307500

H	1.83705500	-1.85032100	-1.63987200
C	3.77585000	-2.69669400	-1.97087700
H	3.40741600	-3.44950000	-2.65752300
C	5.13008500	-2.62746200	-1.67112600
H	5.81980400	-3.32994800	-2.12424400
C	5.60711800	-1.65449400	-0.79780000
H	6.66548300	-1.59938700	-0.57176900
C	4.73288900	-0.74665900	-0.22026100
H	5.11773000	0.01969100	0.44387000
C	2.87814600	2.02885100	0.05034100
C	2.86566900	2.96009100	1.09210200
H	2.48950000	2.70267800	2.07497300
C	3.37657600	4.23506900	0.88583000
H	3.38581200	4.94730100	1.70257100
C	3.88238100	4.59273500	-0.35729000
H	4.28245300	5.58772700	-0.51315200
C	3.88710000	3.67123000	-1.39938800
H	4.29050000	3.94589200	-2.36686700
C	3.38537300	2.39444800	-1.20136700
H	3.41746300	1.67728300	-2.01506200
Co	0.02853300	0.01838500	-0.43584100
P	-2.01253300	0.84740200	0.27996500
P	-0.11864800	-1.43357300	1.25407200
P	2.20292100	0.35160000	0.25172800
H	0.55641100	0.99457600	-1.67218200
H	-0.24078800	1.15156400	-1.62221700

H	-3.17128200	5.89353600	-0.16587700
C	-3.41817600	-0.41656900	1.14387500
C	-2.99983700	-1.42446500	2.01384500
C	-4.74360200	0.02686900	1.17115100
C	-3.90171700	-1.98801300	2.90585600
H	-1.97155000	-1.77084700	2.00407600
C	-5.63859800	-0.54344400	2.06382000
H	-5.07605300	0.82106300	0.51199400
C	-5.21949200	-1.54897300	2.92859300
H	-3.57593400	-2.76625500	3.58576000
H	-6.66582900	-0.19932000	2.08759700
H	-5.92264100	-1.98760300	3.62687000
P	-0.00358500	-0.89705200	-1.76006600
C	-0.08767400	-2.70872500	-1.60450000
C	-0.19817600	-3.50289400	-2.75004900
C	-0.02786600	-3.31146700	-0.34681800
C	-0.25267900	-4.88353100	-2.62974000
H	-0.24211100	-3.05660000	-3.73812200
C	-0.08099400	-4.69412100	-0.23261900
H	0.06464100	-2.70770400	0.55194400
C	-0.19513400	-5.47827700	-1.37353800
H	-0.34003400	-5.49734600	-3.51845200
H	-0.03588200	-5.15941700	0.74493900
H	-0.23910400	-6.55747300	-1.28532600
H	-0.39590900	0.61803100	1.73089800
H	0.42648200	0.65104100	1.71565300

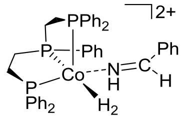

***fac*-II'**

C	-2.39754700	1.51308100	-1.81366500
H	-2.70080700	0.54499400	-2.21723000
H	-3.21705300	2.21276700	-1.98803100
C	-1.10891900	2.00449000	-2.45716300
H	-0.92987600	3.05225400	-2.20613500
H	-1.13816800	1.93253100	-3.54679000
C	0.64603100	-0.37685300	-2.94461700
H	0.52776200	-0.04806100	-3.98045400
H	1.69268300	-0.65454900	-2.80647700
C	-0.26354900	-1.55837900	-2.63001800
H	0.06037600	-2.44332600	-3.18150400
H	-1.29273800	-1.35836600	-2.93580600
C	-3.64684400	0.66865700	0.73555800
C	-4.69176400	0.17127300	-0.04380300
H	-4.61385900	0.11946300	-1.12269100
C	-5.86650000	-0.25569300	0.56301700
H	-6.68014200	-0.62718400	-0.04876300
C	-6.00016100	-0.20189300	1.94375500
H	-6.92035400	-0.53041900	2.41262100
C	-4.95721300	0.28458700	2.72526400

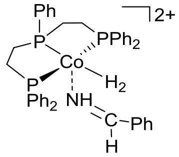

***mer*-II'**

C	2.37655400	1.68230000	-1.93363000
H	2.43883300	2.66299600	-2.40679100
H	3.38784900	1.38357800	-1.65124700
C	1.76941700	0.64341400	-2.87469100
H	2.47078000	0.35879600	-3.66183200
H	0.86243500	1.01688900	-3.35686500
C	0.34406000	-2.07288500	-2.74752100
H	-0.47487200	-1.57390500	-3.27257900
H	0.96424400	-2.56955900	-3.49683900
C	-0.16720000	-3.05713900	-1.69852100
H	0.65931200	-3.65537800	-1.30871200
H	-0.90197400	-3.74921300	-2.11245700
C	0.60468500	3.46781800	-0.39867600
C	-0.01175600	3.90233200	-1.57464800
H	-0.01597400	3.28115000	-2.46341500
C	-0.61433200	5.15074800	-1.62451100
H	-1.07643800	5.48975700	-2.54433100
C	-0.61678300	5.96907200	-0.50060400
H	-1.08218700	6.94673700	-0.54370000
C	-0.01190000	5.53804800	0.67282300

H	-5.06286100	0.33740400	3.80252300	H	-0.00047600	6.17957600	1.54625700
C	-3.78563700	0.72061000	2.12593600	C	0.59672700	4.29089300	0.72908500
H	-2.99340200	1.12918200	2.74506300	H	1.08625100	3.97524600	1.64293200
C	-1.93788800	2.99942800	0.67281800	C	2.88432000	-1.61542700	-1.37631700
C	-2.91601900	3.96156600	0.40637800	C	3.73361800	-2.06803200	-2.39237700
H	-3.77690100	3.72143200	-0.20817300	H	3.47557900	-1.93595700	-3.43819600
C	-2.80564700	5.23240900	0.95031200	C	4.92534500	-2.69784100	-2.06833200
H	-3.56673500	5.97467300	0.73994800	H	5.58114300	-3.04711400	-2.85713600
C	-1.73053600	5.55064100	1.77434100	C	5.27688700	-2.88151500	-0.73472200
H	-1.65450000	6.54244900	2.20447600	H	6.20905800	-3.37507800	-0.48593300
C	-0.76259300	4.59596900	2.05478200	C	4.43707900	-2.43486000	0.27621900
H	0.06719100	4.83746900	2.70889900	H	4.71168800	-2.57805800	1.31444800
C	-0.86518900	3.32385600	1.50395400	C	3.24236400	-1.80168300	-0.04176500
H	-0.10824100	2.58051900	1.73899000	H	2.59377000	-1.45297300	0.75351600
C	1.78739400	2.17425300	-2.00564000	C	-2.70539900	-2.25923000	-0.41238300
C	2.80139600	1.94127300	-2.93874400	C	-3.30251800	-2.39543200	-1.66668600
H	2.78519400	1.07004500	-3.58134000	H	-2.70430800	-2.46387100	-2.56841600
C	3.84332500	2.85024200	-3.07386600	C	-4.68459300	-2.48043100	-1.77506400
H	4.61858700	2.66930000	-3.80912700	H	-5.14066400	-2.60922800	-2.74990300
C	3.88648600	3.98913100	-2.28045200	C	-5.47787000	-2.42119900	-0.63600100
H	4.69809300	4.69818200	-2.39467000	H	-6.55522500	-2.50196800	-0.72156300
C	2.88410200	4.22377400	-1.34574900	C	-4.88794800	-2.27484700	0.61391500
H	2.91233700	5.11454700	-0.72927500	H	-5.50327900	-2.23959700	1.50523200
C	1.83956800	3.32197400	-1.20549100	C	-3.50787800	-2.18939800	0.72841200
H	1.05941400	3.52401400	-0.47854800	H	-3.05913700	-2.10283900	1.71193600
C	1.07468000	-3.05556900	-0.46335400	P	1.40630400	1.82276400	-0.33162900
C	1.03667700	-3.81006000	0.71307900	P	1.31593700	-0.80201200	-1.82155900
H	0.17578500	-3.75563200	1.37200000	P	-0.88708100	-2.16045500	-0.22020600
C	2.07313300	-4.67900000	1.02316500	C	-0.50247200	-3.21077900	1.22339200
H	2.02127000	-5.27624200	1.92632000	C	-0.86876900	-4.56038400	1.21899700
C	3.16238000	-4.80133600	0.16762900	C	0.15131200	-2.67882600	2.33367900
H	3.96619300	-5.48816100	0.40505400	C	-0.57136700	-5.36135500	2.31083300
C	3.20725400	-4.05501300	-1.00144400	H	-1.39738100	-4.98815500	0.37423400
H	4.04550300	-4.15853400	-1.68070900	C	0.44118000	-3.48388500	3.42836700
C	2.17101100	-3.18298800	-1.31515200	H	0.43372300	-1.63172500	2.35402700
H	2.22522800	-2.63223300	-2.24635700	C	0.08254000	-4.82465900	3.41535400
C	-1.78718600	-3.02279200	-0.62319700	H	-0.85705700	-6.40667400	2.30351200
C	-1.94063300	-4.11434100	-1.48506700	H	0.94324500	-3.06376200	4.29197500
H	-1.22435900	-4.30095700	-2.27730000	H	0.30711400	-5.45381500	4.26866400
C	-3.00385000	-4.98739200	-1.31945000	C	2.66001300	1.95476100	0.99206700
H	-3.11825200	-5.82949600	-1.99194700	C	3.81470200	2.71840600	0.79687300
C	-3.91146000	-4.79316300	-0.28278800	C	2.45057500	1.33294800	2.22317700
H	-4.73545400	-5.48434800	-0.14910500	C	4.74519400	2.84462600	1.81764900
C	-3.75760600	-3.72000300	0.58285000	H	3.99054600	3.23099100	-0.14233100
H	-4.45958600	-3.56971200	1.39428300	C	3.38178800	1.46768400	3.24536300
C	-2.70032200	-2.83299300	0.41223900	H	1.55418800	0.74782100	2.39894000
H	-2.60091600	-1.99086500	1.08714100	C	4.53062300	2.21995700	3.04150100
Co	-0.08704700	0.23005600	0.21932000	H	5.63733000	3.43920100	1.65974500
P	-2.09841700	1.30098200	0.00092100	H	3.20878300	0.98762800	4.20149200
P	0.35493700	1.05783000	-1.81262400	H	5.25806700	2.32553800	3.83779800
P	-0.33502600	-1.92297900	-0.80475000	Co	0.03515200	-0.03451900	-0.18693100
H	-0.50660600	-0.72110800	1.49869800	H	-0.48045700	0.73225000	1.19086600
H	-0.53407600	0.03440500	1.80727000	H	-1.38902100	0.43964800	-2.35440300

H	2.43552800	0.85163900	0.20295000
C	2.46239500	-0.46822900	1.61083600
H	1.88022800	-1.19724800	2.17208200
C	3.85589600	-0.36607400	1.98424600
C	4.30270000	-1.17581100	3.03861000
C	4.76296500	0.50045700	1.35384800
C	5.62146100	-1.12056400	3.45740900
H	3.60768800	-1.84881600	3.52958500
C	6.07742900	0.55078500	1.77349300
H	4.45037200	1.13892200	0.53392300
C	6.50823500	-0.25815600	2.82536900
H	5.95904500	-1.74662800	4.27435000
H	6.77674500	1.21966300	1.28645200
H	7.54128400	-0.21204700	3.15016100
N	1.83592900	0.19516500	0.69963000

C	-2.72031500	1.24908900	-1.31543200
H	-3.34736200	1.21940400	-2.21015900
C	-3.38015300	1.84246900	-0.16687600
C	-4.78259500	1.85424500	-0.18211300
C	-2.70801800	2.42818700	0.91296600
C	-5.49453300	2.38487200	0.88065300
H	-5.31386300	1.43532000	-1.03025800
C	-3.42266800	2.97708400	1.96289500
H	-1.63074200	2.52089400	0.89032300
C	-4.81490500	2.94291400	1.95664200
H	-6.57775300	2.38018600	0.86377500
H	-2.89919100	3.45196100	2.78435800
H	-5.36956100	3.37459800	2.78176200
N	-1.53367800	0.75094400	-1.39830800
H	-0.90785300	0.03731000	1.17754100

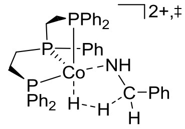

***fac*-TS1'**

C	2.20174200	-1.68489500	-2.07331500
H	2.19466600	-0.94951100	-2.88177800
H	3.11794400	-2.26922100	-2.18914700
C	0.96420600	-2.56304700	-2.11419200
H	1.05969000	-3.43671800	-1.46673600
H	0.77325200	-2.94004400	-3.12237400
C	-1.17416100	-0.62683200	-2.92692700
H	-0.80465200	-1.02174700	-3.87570100
H	-2.25982100	-0.74187000	-2.92418700
C	-0.80266600	0.84098600	-2.76206600
H	-1.40887500	1.46928900	-3.41849200
H	0.24927400	1.02304600	-2.99590500
C	3.51684100	0.63592400	-0.91137100
C	4.82702600	0.26230400	-1.23399000
H	5.13219600	-0.77751500	-1.19333500
C	5.75511600	1.22814800	-1.59001900
H	6.76808100	0.93353400	-1.83773200
C	5.38999900	2.57000000	-1.62169300
H	6.12017700	3.32274800	-1.89487400
C	4.09437400	2.94579000	-1.29607900
H	3.80983700	3.99100800	-1.31098600
C	3.15764100	1.98163800	-0.94292700
H	2.15125200	2.30103900	-0.69103200
C	3.15823400	-1.70199800	0.75987200
C	3.80241300	-1.04895600	1.81700300
H	3.85702000	0.03426000	1.84079200
C	4.42340100	-1.78582300	2.81542400
H	4.93513100	-1.27321200	3.62158800
C	4.40185100	-3.17540500	2.77517900
H	4.89314900	-3.74828000	3.55275200
C	3.76488600	-3.82927700	1.72825000
H	3.76057600	-4.91209900	1.68498500

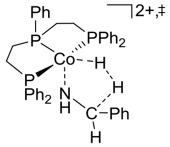

***mer*-TS1'**

C	1.43999500	-2.34980900	-1.84641400
H	2.41508800	-2.62624600	-2.24852500
H	0.93997500	-3.26532200	-1.52373500
C	0.58688900	-1.62808900	-2.88969900
H	0.27342300	-2.30645400	-3.68636500
H	1.10894900	-0.78070300	-3.33958500
C	-2.02466600	0.06163200	-2.91261000
H	-1.45935800	0.87711800	-3.36871400
H	-2.50829400	-0.50112400	-3.71342800
C	-3.04423100	0.55397400	-1.88645700
H	-3.73435400	-0.25190200	-1.63027000
H	-3.63682300	1.38616900	-2.26790200
C	3.41692200	-0.72582800	-0.45102100
C	3.96924300	-0.45592400	-1.70598700
H	3.36529900	-0.49690300	-2.60489200
C	5.31734100	-0.15201500	-1.81968200
H	5.74602800	0.03190500	-2.79795300
C	6.11636900	-0.09436900	-0.68381500
H	7.17269600	0.12978700	-0.77591800
C	5.56241800	-0.32946100	0.56815300
H	6.18371100	-0.28660400	1.45489000
C	4.21592900	-0.64132100	0.69041200
H	3.80435100	-0.85390500	1.66964100
C	-1.81637100	-2.45959000	-1.46479300
C	-2.44618300	-3.20908200	-2.46585300
H	-2.38489100	-2.91606600	-3.50896400
C	-3.15879700	-4.34997700	-2.13030700
H	-3.64529700	-4.92790300	-2.90725300
C	-3.24697100	-4.75096400	-0.80094400
H	-3.80575200	-5.64296900	-0.54286500
C	-2.62023900	-4.01233900	0.19314200
H	-2.68781700	-4.32593000	1.22800600

C	3.13761400	-3.09914300	0.72630600
H	2.66621200	-3.63450900	-0.08877600
C	-1.77976600	-2.99626500	-1.29536000
C	-2.06124000	-3.51962600	-0.03253300
H	-1.59934600	-3.08967100	0.84770000
C	-2.94677600	-4.58319000	0.09031600
H	-3.16551800	-4.98760400	1.07179300
C	-3.55025500	-5.12660300	-1.03609800
H	-4.23943900	-5.95692600	-0.93565300
C	-3.27115500	-4.60640600	-2.29474400
H	-3.73990500	-5.02904200	-3.17563800
C	-2.38935300	-3.54419700	-2.42936900
H	-2.18412400	-3.15720100	-3.42131100
C	-2.87391300	1.26828600	-0.71467400
C	-3.66202500	2.34941500	-1.12289600
H	-3.20578700	3.24066700	-1.53801600
C	-5.04047200	2.28543500	-0.98738800
H	-5.64967700	3.12343200	-1.30518400
C	-5.63841400	1.15486900	-0.44067500
H	-6.71612200	1.11221300	-0.33435600
C	-4.85705000	0.08488200	-0.02512600
H	-5.32188900	-0.79316800	0.40740400
C	-3.47577100	0.14068600	-0.15647800
H	-2.87470700	-0.69407400	0.18505200
C	-0.59425700	3.07939600	-0.82749200
C	-0.19521400	3.84693900	-1.92282300
H	-0.13401700	3.41931100	-2.91619200
C	0.12120800	5.18943600	-1.74913300
H	0.42175800	5.78382500	-2.60404800
C	0.03992800	5.76847400	-0.49006000
H	0.28044700	6.81710600	-0.35962300
C	-0.35718800	5.00548800	0.60356500
H	-0.42756100	5.45898000	1.58523000
C	-0.67244500	3.66538700	0.43985800
H	-0.99741200	3.08394200	1.29715000
Co	0.12621600	-0.26148000	0.15913300
P	2.32022800	-0.68448200	-0.50950200
P	-0.54553800	-1.66902000	-1.51040100
P	-1.07028600	1.32710000	-0.99572700
H	0.48329300	0.65304900	1.34442800
H	0.70293800	-2.17862500	1.59442100
C	0.24137900	-0.43674300	2.37819800
H	1.26461800	-0.35628900	2.76033100
C	-0.76818600	0.27127100	3.20021900
C	-0.37167100	1.39019800	3.93977500
C	-2.07517600	-0.20412700	3.31165800
C	-1.27477000	2.03016100	4.77304700
H	0.65151600	1.74969700	3.87818500
C	-2.97456600	0.43834000	4.14838900
H	-2.36501400	-1.09681100	2.77217300
C	-2.57907200	1.55635300	4.87489900
H	-0.96106500	2.88827800	5.35558800
H	-3.98385100	0.05742300	4.25016200
C	-1.90474300	-2.86742000	-0.13474900
H	-1.41610800	-2.29662600	0.64528200
C	-2.35375600	2.88489100	-0.21194900
C	-2.30639700	3.65189700	-1.38086600
H	-2.25931700	3.18467400	-2.35969300
C	-2.36777300	5.03756700	-1.30900300
H	-2.34703800	5.62465700	-2.21963400
C	-2.47129200	5.66675900	-0.07442000
H	-2.52579100	6.74766700	-0.02100400
C	-2.52011300	4.90943600	1.09006200
H	-2.61751700	5.39821800	2.05235500
C	-2.45593000	3.52436200	1.02707900
H	-2.51781500	2.94339800	1.94036100
P	1.68485600	-1.30223600	-0.30830500
P	-0.85780100	-0.98607400	-1.95118300
P	-2.22785600	1.05842900	-0.27120000
C	-3.33914300	0.47129700	1.05414600
C	-4.71728700	0.65201000	0.89079800
C	-2.85431200	-0.07975900	2.23774300
C	-5.59061500	0.26970500	1.89731000
H	-5.11817600	1.10261600	-0.01031500
C	-3.73361000	-0.45436700	3.24596200
H	-1.78934300	-0.22112400	2.38272500
C	-5.10048600	-0.28297500	3.07545600
H	-6.65673100	0.41084500	1.76408800
H	-3.34936000	-0.87924100	4.16581000
H	-5.78626700	-0.57605100	3.86172100
C	1.68763200	-2.45551400	1.10951600
C	2.23449100	-3.73321300	0.95758400
C	1.22674300	-2.04980500	2.36172300
C	2.29746100	-4.59420100	2.04344100
H	2.62567500	-4.06339500	0.00227400
C	1.30194000	-2.91267600	3.44761600
H	0.82028900	-1.05454700	2.49984500
C	1.83243500	-4.18599900	3.28825000
H	2.71952000	-5.58439600	1.91799600
H	0.94908700	-2.58837300	4.41966900
H	1.88938000	-4.86005000	4.13492300
Co	-0.02377100	0.33863800	-0.35392600
H	0.50470800	1.14367600	0.84267000
H	-0.17815700	2.38634200	-1.77712900
C	0.86766900	2.32773100	-0.10375000
H	0.12609700	2.98897000	0.35607100
C	2.24747900	2.60197400	0.35439200
C	2.47840800	2.88874600	1.70378900
C	3.27613900	2.76276100	-0.57322200
C	3.72590900	3.31917300	2.12106600
H	1.67400900	2.79254900	2.42747400
C	4.51939100	3.21166500	-0.15266400
H	3.08166600	2.57670600	-1.62205400
C	4.74749500	3.48555500	1.19031600
H	3.90023200	3.54571000	3.16624900
H	5.30961100	3.36344700	-0.87818400

H	-3.28285100	2.04914200	5.53550200
N	-0.08054500	-1.52903900	1.64307200
H	0.94474100	0.94777600	0.02502900

H	5.71771200	3.84567600	1.51238400
N	0.58606300	1.86344800	-1.35563300
H	-0.32419500	0.04793200	1.06720300

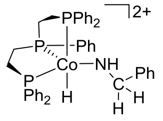

***fac*-III'**

C	0.90831400	-2.00227500	-2.54987500
H	1.67130100	-1.35482100	-2.98612600
H	1.17872100	-3.02284700	-2.82886400
C	-0.48364400	-1.62720100	-3.04260100
H	-1.20010300	-2.42120000	-2.82187400
H	-0.49950300	-1.46826700	-4.12335600
C	-0.35596100	1.34687400	-3.02526500
H	-0.29350600	1.16716500	-4.10112700
H	-1.02853400	2.19383200	-2.88083200
C	1.02625800	1.66722500	-2.46060000
H	1.35741100	2.64950800	-2.80489800
H	1.76564300	0.93596200	-2.79285700
C	2.70887500	-2.24780300	-0.21771700
C	2.92018700	-2.93347300	0.98080800
H	2.08428600	-3.21047100	1.61201000
C	4.20664000	-3.29487000	1.35320500
H	4.36431900	-3.84003800	2.27639200
C	5.28574900	-2.97697400	0.53739200
H	6.28787100	-3.27272200	0.82508000
C	5.07916200	-2.29091000	-0.65297100
H	5.91814100	-2.04979700	-1.29490100
C	3.79666700	-1.92039500	-1.03070200
H	3.66156000	-1.38945900	-1.96532800
C	-0.12224300	-3.06269700	-0.00655700
C	-0.41405500	-4.24887800	-0.68717800
H	0.05124100	-4.47737300	-1.63868900
C	-1.28697500	-5.17118700	-0.13049400
H	-1.49439800	-6.09663000	-0.65502500
C	-1.88668800	-4.91772900	1.09982900
H	-2.56646400	-5.64456100	1.52901000
C	-1.60293000	-3.74309100	1.78223800
H	-2.05679900	-3.54636000	2.74629800
C	-0.71964300	-2.81877600	1.23673900
H	-0.47646500	-1.92308100	1.80000300
C	-2.91297000	-0.06683600	-2.49255300
C	-3.48591700	0.76769800	-3.45408400
H	-2.87673200	1.43435200	-4.05272900
C	-4.85809500	0.73681200	-3.66695200
H	-5.29828700	1.38129800	-4.41873400
C	-5.66203700	-0.12062400	-2.92674500
H	-6.73163000	-0.14249200	-3.09935800
C	-5.09592500	-0.95471000	-1.96935500
H	-5.72208900	-1.62757200	-1.39535500
C	-3.72688000	-0.92676900	-1.74725100
H	-3.29470100	-1.59103200	-1.00423600

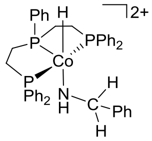

***mer*-III'**

C	-3.27371700	0.53158900	1.59561800
H	-3.68631400	1.29869800	2.25266400
H	-4.06755400	0.20064300	0.92219400
C	-2.72543200	-0.65009700	2.39197200
H	-3.53214200	-1.25465000	2.81259200
H	-2.08728200	-0.32446900	3.21692400
C	-0.75105200	-2.95777500	2.08671800
H	-0.33071100	-2.52214200	2.99623100
H	-1.39894200	-3.78542800	2.37928800
C	0.33973700	-3.41481700	1.12195700
H	-0.09412000	-3.92840800	0.25982700
H	1.04559600	-4.11021600	1.58014100
C	-1.24847600	2.66240800	1.50000600
C	-0.62211400	2.36893800	2.71714100
H	-0.55424000	1.34791100	3.07884400
C	-0.08457500	3.38806600	3.48982700
H	0.38774700	3.15485500	4.43692600
C	-0.15685100	4.70542400	3.05137900
H	0.25822200	5.50186300	3.65776300
C	-0.76417400	5.00014200	1.83733500
H	-0.82364700	6.02646700	1.49429400
C	-1.30463800	3.98502200	1.05807500
H	-1.77911200	4.22946100	0.11558600
C	-2.91614700	-2.51146800	0.15154300
C	-3.68771000	-3.53197800	0.71922000
H	-3.55658500	-3.82409500	1.75569500
C	-4.64444500	-4.18724400	-0.04114900
H	-5.23497000	-4.97866400	0.40511900
C	-4.84574900	-3.82847800	-1.36941500
H	-5.59364400	-4.34290200	-1.96133000
C	-4.09245500	-2.80893900	-1.93486700
H	-4.25348900	-2.52248200	-2.96745400
C	-3.13048100	-2.15071700	-1.17903300
H	-2.55894700	-1.34793900	-1.63002200
C	2.40748200	-1.39255100	1.73471700
C	2.56985800	-2.09209500	2.93477400
H	2.00226800	-2.99039300	3.14343800
C	3.49234900	-1.65558200	3.87584600
H	3.61863100	-2.20907600	4.79891000
C	4.26081100	-0.52497900	3.62899300
H	4.98552600	-0.19262900	4.36306900
C	4.10778700	0.17204900	2.43604000
H	4.71661900	1.04538800	2.23466700
C	3.18099200	-0.25068300	1.49551000

C 0.46868300 3.30018300 -0.08404600
C 1.32604300 4.18572200 0.57318100
H 2.33063400 3.88360300 0.84009200
C 0.89545000 5.47113700 0.87776800
H 1.56670600 6.15236000 1.38724500
C -0.38228300 5.88298000 0.52639100
H -0.71205400 6.88847500 0.76011900
C -1.24254600 5.00247800 -0.12249000
H -2.24263300 5.32091900 -0.39299000
C -0.82548800 3.71444300 -0.41810700
H -1.52088600 3.03033100 -0.89038200
C 2.82983500 1.58126400 -0.13436400
C 3.80640400 2.04064600 -1.02442000
H 3.54588800 2.38226000 -2.01862800
C 5.13653500 2.08948100 -0.63047000
H 5.88698300 2.45110300 -1.32369300
C 5.50138200 1.69055300 0.64919700
H 6.54003600 1.73699300 0.95516500
C 4.53444900 1.23623900 1.53793100
H 4.81608200 0.92475800 2.53672200
C 3.20455600 1.17741700 1.14865300
H 2.46030400 0.81622700 1.84918900
Co -0.21897300 -0.04091300 -0.04346100
P 1.01198000 -1.80635400 -0.70687400
P -1.12104700 -0.11176700 -2.17320200
P 1.06931800 1.64968100 -0.59552300
N -1.51439900 0.74914800 0.93217100
H -2.45703900 0.40588400 0.73318600
H 0.68136500 -0.02354900 1.11194300
C -1.55829200 1.65017300 2.06810100
H -0.55075100 1.99888200 2.28865300
H -2.16773500 2.52444800 1.81121200
C -2.16950400 0.92929900 3.25004600
C -1.35824700 0.20708500 4.12554700
C -3.54709100 0.96555400 3.46493100
C -1.91541300 -0.45228300 5.21282800
H -0.28300000 0.18976800 3.97779200
C -4.10425500 0.30015100 4.54845600
H -4.18639300 1.54226400 2.80310300
C -3.28917000 -0.41019200 5.42241200
H -1.27709800 -0.98777800 5.90621100
H -5.17287200 0.35018200 4.72172700
H -3.72314100 -0.91777100 6.27596900

H 3.08342300 0.29457900 0.56379800
P -1.94706800 1.28843000 0.50798000
P -1.70600900 -1.64056900 1.21130800
P 1.23623000 -1.93942500 0.44610800
C 2.17988100 -2.50523500 -1.00336200
C 3.55257600 -2.74577100 -0.92180400
C 1.49628800 -2.75663900 -2.19732600
C 4.23017400 -3.24099200 -2.02795100
H 4.09220300 -2.55504100 -0.00218600
C 2.18184000 -3.25288300 -3.29598600
H 0.42792200 -2.57781300 -2.27264500
C 3.54884600 -3.49412700 -3.21150100
H 5.29473100 -3.43213000 -1.96175100
H 1.64983500 -3.45353300 -4.21850800
H 4.08308600 -3.88232600 -4.07075600
C -2.83008800 2.00151500 -0.91508800
C -3.97841400 2.77467100 -0.71147000
C -2.37920500 1.76149500 -2.21333300
C -4.65714100 3.30307000 -1.79959000
H -4.34455000 2.97601000 0.28920400
C -3.06302600 2.29575100 -3.29787700
H -1.49785300 1.15238000 -2.37697300
C -4.19992600 3.06588000 -3.09147100
H -5.54499300 3.90312000 -1.63832000
H -2.70929200 2.10883200 -4.30507500
H -4.73338200 3.48220700 -3.93796500
Co -0.26196200 -0.27205200 0.17265800
N 0.84474800 0.71507300 -0.88771200
H 1.27958400 0.22843300 -1.67114500
H -0.67631000 -1.00350500 -0.96978200
C 1.19945700 2.12643300 -0.92087400
H 0.41683100 2.64203700 -1.50036800
H 1.14844500 2.53659200 0.08876500
C 2.54612400 2.38518000 -1.54740900
C 2.75677100 2.12708100 -2.90241800
C 3.58969900 2.90343400 -0.78392800
C 3.99487900 2.37029200 -3.48011300
H 1.94413700 1.75943600 -3.52337000
C 4.82711900 3.15555800 -1.36432700
H 3.42900500 3.13474200 0.26449500
C 5.03239300 2.88435800 -2.71055200
H 4.14646200 2.17673200 -4.53558400
H 5.62911200 3.57320700 -0.76668400
H 5.99566500 3.08577300 -3.16420300

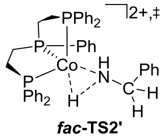

***fac*-TS2'**

C -0.04151900 -3.65290900 -0.26858100
H 0.38427200 -3.87603300 -1.25056600
H 0.17841100 -4.50902700 0.37182200
C -1.54936500 -3.40916900 -0.35693400

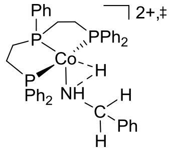

***mer*-TS2'**

C -3.06841800 -0.30949700 -1.77740900
H -3.41942000 -1.02899600 -2.51930600
H -3.92740100 -0.01524700 -1.17085000

H	-2.04624100	-3.71735600	0.56381300
H	-1.98829100	-4.01152800	-1.15515200
C	-1.96685400	-1.36473600	-2.46298500
H	-2.30479200	-2.26722700	-2.97661800
H	-2.69012000	-0.57631100	-2.68158500
C	-0.57528200	-0.95716900	-2.93043600
H	-0.59606000	-0.60672000	-3.96517300
H	0.12709200	-1.79129400	-2.86953500
C	2.65948300	-2.52494600	0.16900400
C	3.13696300	-3.81536500	0.42615500
H	2.46418900	-4.61906900	0.70091900
C	4.49663900	-4.07386600	0.34717300
H	4.86556100	-5.07241300	0.54949700
C	5.38360100	-3.05477200	0.01680800
H	6.44567600	-3.26255800	-0.04141100
C	4.91202100	-1.77354000	-0.23792900
H	5.60367100	-0.98104700	-0.49722400
C	3.55166800	-1.50487500	-0.16651500
H	3.18594000	-0.50729600	-0.37842000
C	0.50619800	-1.77954200	2.06896300
C	-0.79792300	-1.86081400	2.59192000
H	-1.59907800	-2.32411100	2.03496600
C	-1.06723200	-1.40692300	3.88091900
H	-2.07189600	-1.49162400	4.27787800
C	-0.04989000	-0.87771500	4.65807800
H	-0.25867100	-0.53559900	5.66513300
C	1.25276300	-0.80946100	4.15716900
H	2.05059600	-0.41792800	4.77693500
C	1.53006800	-1.24479000	2.87822600
H	2.54359200	-1.19205400	2.49888200
C	-3.74021400	-1.48646200	-0.11988000
C	-4.72054100	-2.27789400	-0.72738200
H	-4.46413800	-2.98047300	-1.51297900
C	-6.04402100	-2.16833300	-0.32749400
H	-6.80111800	-2.78254400	-0.80072200
C	-6.39890300	-1.27382100	0.67670100
H	-7.43438800	-1.19244900	0.98582700
C	-5.42956400	-0.48538700	1.28240600
H	-5.70771600	0.21216700	2.06385400
C	-4.10195900	-0.58917900	0.88608800
H	-3.34717900	0.03003500	1.35888600
C	-0.95141100	1.85694600	-2.32589100
C	-0.53291100	2.69424000	-3.36475800
H	0.43387100	2.55281900	-3.83203500
C	-1.36180400	3.71682200	-3.80545200
H	-1.03162400	4.36327900	-4.61009600
C	-2.60671500	3.91124200	-3.22032500
H	-3.24922700	4.71166500	-3.56815800
C	-3.02659700	3.08173600	-2.18697200
H	-3.99673600	3.23310000	-1.72820400
C	-2.20299200	2.05951400	-1.73791600
H	-2.53010500	1.43140600	-0.91736600
C	1.75889800	0.77545500	-2.42792600
C	-2.44527700	0.91680300	-2.43892800
H	-3.20766100	1.54914800	-2.89906100
H	-1.73158400	0.64273500	-3.22011200
C	-0.48075700	3.16698800	-1.89543600
H	-0.05757800	2.78721500	-2.82878500
H	-1.09406100	4.03893600	-2.12940100
C	0.61505800	3.50465100	-0.88919800
H	0.19484700	3.96134200	0.01070800
H	1.34550500	4.20983800	-1.28856300
C	-1.20044300	-2.58130500	-1.53206600
C	-0.71676700	-2.37548400	-2.82846600
H	-0.77796300	-1.40042200	-3.29923900
C	-0.16966700	-3.42855600	-3.54715600
H	0.18488700	-3.26316400	-4.55782400
C	-0.08787400	-4.69363100	-2.97696300
H	0.33040900	-5.51778400	-3.54279800
C	-0.55564400	-4.90241000	-1.68587400
H	-0.50469100	-5.88953800	-1.24133700
C	-1.10516800	-3.85259000	-0.96102000
H	-1.48796200	-4.03972800	0.03530900
C	-2.75444900	2.68857700	-0.10471300
C	-3.64235000	3.57083200	-0.73127100
H	-3.60589100	3.73491300	-1.80341000
C	-4.58778100	4.25344500	0.01858000
H	-5.27287300	4.93464100	-0.47218400
C	-4.65494300	4.06529800	1.39503500
H	-5.39448600	4.60127700	1.97824100
C	-3.77651900	3.19194500	2.02133700
H	-3.82966000	3.04439100	3.09356000
C	-2.82878600	2.50346300	1.27480100
H	-2.15141100	1.81841700	1.77044800
C	2.47613400	1.38357300	-1.75724700
C	2.97369000	2.28680200	-2.70222700
H	2.75257600	3.34528100	-2.63726200
C	3.78406500	1.83582900	-3.73408500
H	4.17241400	2.54214400	-4.45847400
C	4.10533200	0.48659800	-3.83288600
H	4.74241800	0.14081800	-4.63858800
C	3.61715900	-0.41504600	-2.89587300
H	3.87375700	-1.46537300	-2.96648000
C	2.80161800	0.02653400	-1.86305200
H	2.43968600	-0.68412100	-1.12818400
P	-1.88955700	-1.16711700	-0.59128900
P	-1.51395700	1.83157600	-1.13436400
P	1.43535700	1.93016200	-0.36019700
C	2.50984200	2.31271300	1.05884400
C	3.89206600	2.12773800	0.99242400
C	1.92658600	2.80533200	2.23239300
C	4.68258100	2.45044400	2.08772200
H	4.35312200	1.74722200	0.08911000
C	2.72501500	3.12777100	3.31911100
H	0.85288400	2.95190300	2.29979300
C	4.10298200	2.95069800	3.24643400

C	2.38205400	0.01185800	-3.41686000
H	1.88489900	-0.83745200	-3.86812500
C	3.65810800	0.34743200	-3.85360300
H	4.13025300	-0.24352200	-4.62958500
C	4.32012200	1.43853100	-3.30757600
H	5.31160300	1.70168000	-3.65727300
C	3.70543500	2.20172600	-2.31977300
H	4.21498300	3.06104100	-1.89970500
C	2.43350700	1.87107000	-1.87928000
H	1.95908400	2.48766300	-1.12403000
Co	-0.24618700	-0.28417300	0.22158400
P	0.89075900	-2.16298400	0.31800500
P	-1.99171600	-1.61157200	-0.62148300
P	0.06713400	0.41069100	-1.85374400
N	-0.54351400	1.26655300	1.17598600
H	-0.58662800	1.03152700	2.17078500
H	0.86715400	0.62995500	0.64248800
C	-0.41086200	2.71638700	1.03910900
H	0.02656300	2.94291200	0.06639200
H	-1.42915300	3.12356800	1.01035500
C	0.35510500	3.34689900	2.17212200
C	1.74904000	3.28764900	2.21147700
C	-0.32480000	3.98435300	3.20878100
C	2.44874900	3.85682200	3.26608200
H	2.29723500	2.80960800	1.40495200
C	0.37445000	4.55362400	4.26581200
H	-1.40785600	4.05580000	3.18222300
C	1.76134400	4.48939300	4.29608700
H	3.53200400	3.82015300	3.28057900
H	-0.16381200	5.05893200	5.05918600
H	2.30801000	4.94290800	5.11456900

H	5.75625800	2.31544900	2.03128400
H	2.27395200	3.52165200	4.22229000
H	4.72557200	3.20632400	4.09585800
C	-2.98547000	-1.85424600	0.69474200
C	-3.97886600	-2.77176400	0.33424100
C	-2.89590700	-1.42020600	2.01661900
C	-4.86037200	-3.24935500	1.29227700
H	-4.06461900	-3.12194500	-0.68851300
C	-3.78045500	-1.90395600	2.97194700
H	-2.13735200	-0.70209300	2.30411400
C	-4.76115700	-2.81790200	2.61074800
H	-5.62699100	-3.96087300	1.00876100
H	-3.70432700	-1.56669800	3.99905800
H	-5.45087700	-3.19545700	3.35651300
Co	-0.16679700	0.33369500	-0.11550900
N	0.78238700	-0.58326200	1.12058400
H	1.51817800	-0.05980000	1.59714900
H	-0.43121100	0.72432000	1.27572700
C	0.59977700	-1.85427100	1.81871100
H	0.13470100	-1.67607800	2.79450100
H	-0.06238400	-2.49303200	1.23773000
C	1.95404700	-2.49245100	2.00736200
C	2.66362100	-2.30146800	3.19282500
C	2.51716400	-3.25951300	0.98625400
C	3.91510400	-2.87843600	3.35951000
H	2.22709300	-1.72270400	4.00115300
C	3.76732900	-3.83814000	1.15590900
H	1.96334600	-3.42599400	0.06784800
C	4.46817500	-3.64557200	2.34085900
H	4.45305300	-2.74271700	4.29040800
H	4.19238600	-4.44978600	0.36851900
H	5.44083500	-4.10408000	2.47579600

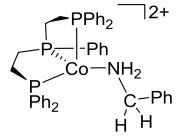

***fac*-IV'**

C	1.89473000	-2.56628100	-1.69285800
H	2.57858300	-2.01215800	-2.34092400
H	2.34697000	-3.55123800	-1.55147900
C	0.49725900	-2.64756100	-2.29555200
H	-0.02919100	-3.53509500	-1.94061700
H	0.53664300	-2.72331100	-3.38448700
C	-0.24709900	0.17996200	-2.96647800
H	-0.05732200	-0.20643100	-3.97095200
H	-1.17580600	0.75273900	-3.01507000
C	0.90836400	1.08633100	-2.52697500
H	0.94319300	1.97845000	-3.15608900
H	1.86939100	0.58238400	-2.65279900
C	3.67486700	-1.49494300	0.36582400
C	4.18746500	-2.14265800	1.49234400
H	3.54105800	-2.71697400	2.14452500

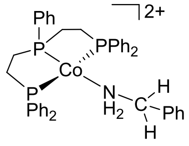

***mer*-IV'**

C	-3.24386000	-0.59553200	1.60686900
H	-3.88859200	-0.05528000	2.30199600
H	-3.88325900	-1.06553900	0.85629700
C	-2.41306400	-1.65404000	2.32872900
H	-3.02850600	-2.50535600	2.62798100
H	-1.93914400	-1.25730700	3.22974000
C	0.19556000	-3.22482700	1.98930100
H	0.50910700	-2.72544200	2.91014100
H	-0.20715600	-4.20319900	2.25771900
C	1.35351500	-3.34473300	1.00123300
H	1.06356100	-3.94745900	0.13788600
H	2.22825100	-3.81344900	1.45460500
C	-1.98932900	2.05939700	1.80322600
C	-1.43447600	1.85079200	3.07088500
H	-1.12861600	0.85932500	3.38913500

C	5.54636900	-2.07209900	1.76792800
H	5.94297000	-2.58266100	2.63759500
C	6.39549900	-1.36583500	0.92584700
H	7.45686400	-1.32229000	1.14036400
C	5.88699300	-0.71967800	-0.19549900
H	6.54839600	-0.16888000	-0.85352800
C	4.53068700	-0.77618500	-0.47432200
H	4.15010100	-0.25377500	-1.34523700
C	1.04262900	-2.57480000	1.20871600
C	0.51911800	-3.86511000	1.06118100
H	0.72643300	-4.44664000	0.17080300
C	-0.23228800	-4.42740700	2.08047900
H	-0.60951500	-5.43801900	1.97426400
C	-0.49375900	-3.70854700	3.24621900
H	-1.07687100	-4.16170900	4.03945900
C	0.01659000	-2.42866800	3.40598200
H	-0.15447400	-1.88396000	4.32740900
C	0.78259000	-1.85750900	2.39214200
H	1.26052700	-0.89534100	2.56685200
C	-2.26212000	-1.83075300	-2.21510800
C	-2.95359900	-1.44116900	-3.36322400
H	-2.53100100	-0.72507600	-4.05771100
C	-4.20212900	-1.98462900	-3.64119500
H	-4.73133400	-1.67923400	-4.53621300
C	-4.76624900	-2.91957900	-2.78385300
H	-5.73830500	-3.34291900	-3.00762300
C	-4.08173000	-3.31401600	-1.63960900
H	-4.51891700	-4.04601200	-0.97046000
C	-2.83932700	-2.76881800	-1.35253300
H	-2.31606200	-3.07982100	-0.45287100
C	-0.29037200	3.01307300	-0.61262800
C	-0.31949200	3.71957000	0.59449500
H	0.32491200	3.42817100	1.41826100
C	-1.12871400	4.83802800	0.73197700
H	-1.12774700	5.39117100	1.66413700
C	-1.92177900	5.26080400	-0.32959100
H	-2.54634900	6.14009200	-0.22464500
C	-1.89902600	4.56373900	-1.53004900
H	-2.50483700	4.89772300	-2.36431100
C	-1.08897900	3.44316100	-1.67322900
H	-1.07498600	2.93246900	-2.62833500
C	2.47148500	2.29080600	-0.33071100
C	3.10938800	3.15725700	-1.22324200
H	2.67658600	3.37698000	-2.19261600
C	4.30187900	3.76723400	-0.86299400
H	4.79365000	4.43746900	-1.55835000
C	4.85463500	3.53602000	0.39239900
H	5.77942100	4.02594400	0.67401400
C	4.22043700	2.68372000	1.28616800
H	4.64926000	2.50399200	2.26496000
C	3.03494600	2.05777400	0.92342100
H	2.55133300	1.38331600	1.62228600
Co	0.14375100	-0.35401900	0.23130000
C	-1.28466900	2.91094400	3.95363200
H	-0.87088500	2.73696700	4.94019400
C	-1.67422000	4.19104100	3.57649600
H	-1.56240600	5.01801300	4.26777200
C	-2.21273600	4.40655800	2.31485600
H	-2.52086500	5.40238000	2.01821700
C	-2.36886500	3.34856900	1.42772600
H	-2.79916200	3.53244200	0.45078800
C	-1.86995800	-3.24789900	-0.08800000
C	-2.44706000	-4.46384100	0.29140500
H	-2.43684000	-4.78781500	1.32703800
C	-3.04663100	-5.27338400	-0.66194800
H	-3.49410600	-6.21421800	-0.36379600
C	-3.07422600	-4.87914500	-1.99574900
H	-3.54400600	-5.51513600	-2.73684600
C	-2.50424500	-3.67213200	-2.37858300
H	-2.53051700	-3.36380700	-3.41709700
C	-1.90382900	-2.85765600	-1.42721000
H	-1.47136600	-1.90993300	-1.73351500
C	2.89780600	-0.96719400	1.69384100
C	4.09694500	-1.61706100	2.00416100
H	4.40707800	-2.49764000	1.45196900
C	4.90887600	-1.12837200	3.01617700
H	5.83845600	-1.63364000	3.25068200
C	4.53273300	0.00597600	3.72874400
H	5.17054000	0.38342600	4.51943600
C	3.34244300	0.65452600	3.42888900
H	3.04904500	1.53724900	3.98479500
C	2.52675900	0.17046200	2.41318700
H	1.59542900	0.68592200	2.19335100
P	-2.17126600	0.62827800	0.66925200
P	-1.08214300	-2.17605500	1.16134400
P	1.79388700	-1.63709000	0.40146300
C	2.76762600	-1.81976700	-1.13177400
C	3.93486200	-1.07975900	-1.33741000
C	2.28732300	-2.64917800	-2.15130100
C	4.62143500	-1.18680600	-2.54034700
H	4.32746300	-0.43968200	-0.55573500
C	2.98319900	-2.75507600	-3.34636200
H	1.37902500	-3.22749900	-2.01896300
C	4.14994500	-2.02434000	-3.54260400
H	5.53275700	-0.61955200	-2.68883600
H	2.61710700	-3.41376200	-4.12519500
H	4.69360700	-2.11270900	-4.47581800
C	-3.22282400	1.15722700	-0.72583000
C	-4.52344600	1.61908500	-0.49857400
C	-2.75303200	1.05025000	-2.03497400
C	-5.33104000	1.97341500	-1.56923000
H	-4.91012200	1.71159400	0.51026700
C	-3.56442300	1.40784000	-3.10408700
H	-1.75131400	0.68039100	-2.22757000
C	-4.85269700	1.87014200	-2.87103000
H	-6.33732700	2.33182400	-1.38672600

P	1.92107100	-1.64761500	-0.08907500
P	-0.59089400	-1.21928400	-1.79241400
P	0.84067900	1.57184500	-0.73216100
N	-1.51875900	0.29428400	1.14256000
H	-1.75611300	-0.43403500	1.81775200
H	-1.27622600	1.10247400	1.71575900
C	-2.79860200	0.64769900	0.43652900
H	-2.58881600	1.51834500	-0.18382600
H	-3.04880700	-0.18788800	-0.21508600
C	-3.91053700	0.91950900	1.40956600
C	-4.09003100	2.19321200	1.95033800
C	-4.75801000	-0.11375300	1.81149700
C	-5.09314900	2.42687000	2.88133300
H	-3.45559100	3.01269400	1.62632500
C	-5.76212300	0.11965200	2.74168600
H	-4.64858300	-1.10315600	1.37707700
C	-5.92831000	1.38988800	3.27966600
H	-5.23283100	3.42160100	3.28833900
H	-6.42400300	-0.68574900	3.03813700
H	-6.71645100	1.57480400	4.00010000

H	-3.19295700	1.32296900	-4.11867000
H	-5.48729100	2.14890600	-3.70407700
Co	-0.12354100	-0.38122700	0.27135800
N	0.86340500	1.11236600	-0.69653600
H	1.83977100	1.09756300	-0.40357800
H	0.92040500	0.82904100	-1.67605800
C	0.45269000	2.55847100	-0.68223400
H	-0.56194900	2.61147200	-1.07625700
H	0.42207900	2.87532000	0.35979700
C	1.38902600	3.41649000	-1.48273300
C	1.19204400	3.58859100	-2.85364300
C	2.48527300	4.02736400	-0.87284000
C	2.08025200	4.34784100	-3.60282000
H	0.32578600	3.14683000	-3.33815900
C	3.37385800	4.78763400	-1.62137600
H	2.63601700	3.92732500	0.19823400
C	3.17341500	4.94599700	-2.98725800
H	1.91274200	4.48597900	-4.66460500
H	4.21551900	5.26880000	-1.13695900
H	3.86153300	5.54704500	-3.57001700

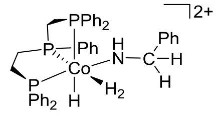

fac-**V'**

C	-1.13502900	1.51537700	-2.71552900
H	-1.71011500	0.69104500	-3.13894500
H	-1.53750500	2.44227100	-3.12733000
C	0.33910900	1.35010200	-3.04652500
H	0.91090100	2.24584500	-2.79337500
H	0.50153800	1.14268800	-4.10695900
C	0.34968900	-1.61547600	-2.73108900
H	0.16457600	-1.49194000	-3.80097600
H	1.12427500	-2.37600300	-2.62046700
C	-0.90957500	-2.06372200	-1.99380100
H	-1.13980200	-3.10168500	-2.24235700
H	-1.77482600	-1.45352000	-2.26042800
C	-3.13533000	1.10712900	-0.58286400
C	-3.99506100	0.67362500	-1.59356800
H	-3.64961900	0.53295600	-2.61025800
C	-5.33954300	0.45333300	-1.31852700
H	-6.00336000	0.13697300	-2.11469400
C	-5.83326600	0.65531700	-0.03769100
H	-6.88433100	0.49309000	0.17034100
C	-4.98035600	1.08045200	0.97498100
H	-5.36398300	1.24991100	1.97411600
C	-3.63993500	1.30540400	0.70606400
H	-2.99625000	1.67048200	1.49957000
C	-1.27829100	3.29135700	-0.39015300
C	-2.28806800	4.13822700	-0.86368900
H	-3.11255100	3.74328200	-1.44705400
C	-2.25463000	5.49303400	-0.57516500

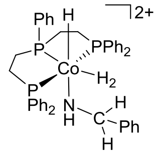

mer-**V'**

C	1.02845200	-2.63315000	1.68026900
H	0.67258600	-3.62775900	1.95222600
H	2.12025000	-2.66272900	1.70173300
C	0.51064500	-1.55585200	2.63185100
H	0.94842800	-1.65911900	3.62659300
H	-0.57662500	-1.58671100	2.73297000
C	0.24545400	1.52963200	2.66124100
H	-0.83037100	1.37567100	2.77299000
H	0.66487300	1.68612700	3.65703000
C	0.56356500	2.70096200	1.73291100
H	1.62773700	2.94205300	1.78137200
H	0.01247500	3.60022200	2.01150600
C	-0.86441700	-3.30079900	-0.51121600
C	-1.69287000	-3.85239800	0.46668800
H	-1.49687100	-3.69540200	1.52141700
C	-2.77365800	-4.64455400	0.09823100
H	-3.40146600	-5.08627500	0.86314500
C	-3.04349700	-4.87752800	-1.24387100
H	-3.88447800	-5.49906700	-1.52775400
C	-2.22624700	-4.32302500	-2.22273700
H	-2.42612500	-4.51379900	-3.27065000
C	-1.14134600	-3.53724200	-1.86148100
H	-0.49123400	-3.14085300	-2.63473900
C	2.78534800	0.20028900	2.15533900
C	3.21128000	0.18510000	3.49027800
H	2.50157800	0.08338500	4.30510600
C	4.55899700	0.30060900	3.79195000

H	-3.03946700	6.14009200	-0.94893900
C	-1.22417400	6.01786600	0.19777400
H	-1.20374400	7.07687600	0.42682100
C	-0.22723000	5.18254000	0.67978900
H	0.57376300	5.58523100	1.28841800
C	-0.25082600	3.82354700	0.38683500
H	0.53946000	3.19165300	0.77395400
C	2.83375000	0.00541400	-2.31090600
C	3.48935500	-0.96392600	-3.07458900
H	2.95198400	-1.79822200	-3.50753600
C	4.85311400	-0.85346100	-3.31320200
H	5.35308800	-1.60398600	-3.91399500
C	5.57079200	0.21613700	-2.79443700
H	6.63397700	0.29912300	-2.98712800
C	4.92436000	1.18495300	-2.03482800
H	5.48027600	2.02508800	-1.63565600
C	3.56283600	1.08249500	-1.79051600
H	3.07025200	1.85147200	-1.20357900
C	0.43131500	-3.31000400	0.27037100
C	-0.10613700	-4.45858800	0.85601400
H	-1.15506700	-4.50186400	1.11952700
C	0.70311600	-5.55943000	1.10753200
H	0.27434400	-6.44256800	1.56650600
C	2.05005300	-5.53181700	0.77357600
H	2.67731100	-6.39313300	0.97110700
C	2.59248600	-4.39434000	0.18770700
H	3.64381600	-4.36671000	-0.07477600
C	1.79003900	-3.28880500	-0.05770900
H	2.23791800	-2.40737800	-0.50130900
C	-2.24916900	-2.27549800	0.64824400
C	-3.32304700	-2.83266300	-0.04603600
H	-3.27188400	-3.00195400	-1.11453200
C	-4.47686200	-3.19814700	0.63666700
H	-5.30455300	-3.63684600	0.09182600
C	-4.56462600	-3.01719400	2.01021900
H	-5.46206000	-3.31460000	2.53995600
C	-3.49790700	-2.46103300	2.70749700
H	-3.56068600	-2.32403700	3.78068100
C	-2.34776800	-2.08736700	2.02958200
H	-1.51524800	-1.67504100	2.59171300
Co	0.29215600	0.21893200	0.02721700
P	-1.37532500	1.51923700	-0.87202500
P	1.03366900	-0.02805800	-2.03385100
P	-0.65959300	-1.88992200	-0.16064200
H	0.94895500	1.51596900	-0.13655500
N	1.89895000	-0.30691800	0.85119300
H	2.77300600	-0.15429100	0.34887800
H	-0.07971800	0.93165200	1.43788500
H	-0.50192200	0.22215000	1.45534800
C	2.15997000	-0.62266600	2.24428100
H	1.23355500	-0.92545900	2.73167500
H	2.85134500	-1.47168700	2.28416500
C	2.76941700	0.58004500	2.93720500
H	4.88113500	0.28652300	4.82648300
C	5.49263700	0.43371500	2.76963100
H	6.54598800	0.52321300	3.00798100
C	5.07562900	0.45199900	1.44628900
H	5.80160900	0.55677300	0.64884500
C	3.72598400	0.33575500	1.13672100
H	3.41260300	0.35220700	0.10063400
C	-1.35996300	3.09590800	-0.48462800
C	-2.32344700	3.37812100	0.48496300
H	-2.16145500	3.12870600	1.52747500
C	-3.50365600	4.01705900	0.12788900
H	-4.23873900	4.25094600	0.88883900
C	-3.73593500	4.36335600	-1.19717400
H	-4.65259000	4.87232900	-1.47132500
C	-2.78650700	4.06952300	-2.16918100
H	-2.96010000	4.34865400	-3.20188100
C	-1.60203100	3.43682800	-1.81803700
H	-0.85378100	3.24679000	-2.58001300
P	0.57716300	-2.25371400	-0.09363600
P	0.99127700	0.04727800	1.85311400
P	0.22010000	2.27587400	-0.05804900
C	1.48099200	3.16833400	-1.03121900
C	1.66777900	4.53420900	-0.79150000
C	2.22802000	2.53345900	-2.02097500
C	2.60679100	5.24307500	-1.52471000
H	1.07816700	5.05296000	-0.04342400
C	3.15992300	3.25202500	-2.76011100
H	2.08541000	1.47831200	-2.22028000
C	3.35224300	4.60354500	-2.50973500
H	2.75173400	6.29959300	-1.33225200
H	3.73436600	2.75557800	-3.53337700
H	4.08048100	5.16311700	-3.08502300
C	1.93414100	-2.93963600	-1.10527300
C	2.21578300	-4.30606200	-0.99355800
C	2.67960000	-2.15170100	-1.97939700
C	3.24414500	-4.86355200	-1.73686600
H	1.62915800	-4.94169300	-0.33922000
C	3.70240600	-2.71894700	-2.72984600
H	2.46367500	-1.09502200	-2.08056100
C	3.98718200	-4.07162200	-2.60635200
H	3.46103000	-5.92122400	-1.64423000
H	4.27456200	-2.10368300	-3.41429300
H	4.78500200	-4.51337700	-3.19178700
Co	0.18598000	-0.00530800	-0.17620400
H	-0.17152200	0.37592600	-1.72882400
N	-1.66782100	-0.09429000	0.33511000
H	-1.99436300	0.07139100	1.28594100
H	-0.17510400	-0.44403700	-1.71326600
C	-2.81118700	-0.30225200	-0.52644300
H	-2.78692500	0.46408800	-1.31390900
H	-2.64776000	-1.25692300	-1.05156400
C	-4.14291800	-0.29374500	0.17255600
C	-5.17780500	0.51230600	-0.29592500

C	1.97645100	1.42056400	3.71728900
C	4.12847600	0.86064900	2.79339500
C	2.53614900	2.52139500	4.35273600
H	0.92577800	1.19316600	3.86934900
C	4.68600800	1.96342200	3.42497200
H	4.76420100	0.19757500	2.21402900
C	3.88965100	2.79717200	4.20194800
H	1.91926100	3.15457800	4.97988000
H	5.74667800	2.16311000	3.32702000
H	4.32847000	3.65052800	4.70553000

C	-4.36684300	-1.10365200	1.28721100
C	-6.41297800	0.51157800	0.33934300
H	-5.02080500	1.14239000	-1.16506700
C	-5.59689900	-1.09476000	1.93107700
H	-3.58664800	-1.77161600	1.63972900
C	-6.62301700	-0.28684800	1.45636000
H	-7.21377700	1.13647400	-0.03856600
H	-5.76217300	-1.73024100	2.79345100
H	-7.58704900	-0.28665500	1.95119900
H	1.58164000	0.10417000	-0.56774900

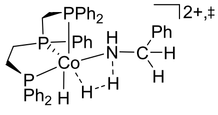

***fac*-TS3'**

C	1.49750900	-1.44520800	-2.73172600
H	1.81642300	-0.52048300	-3.21474100
H	2.16044900	-2.24144200	-3.07532600
C	0.04719900	-1.74184900	-3.07789000
H	-0.24318800	-2.74726600	-2.76440500
H	-0.13560100	-1.66667800	-4.15236900
C	-0.91749300	1.08753400	-3.00115500
H	-0.72892000	0.95321700	-4.06900400
H	-1.88675900	1.57902100	-2.89640900
C	0.16065400	1.94361500	-2.34314600
H	0.06790900	2.98137100	-2.66989900
H	1.16528500	1.59862600	-2.59723500
C	3.24796700	-0.27582400	-0.65794700
C	3.98650500	0.25157300	-1.71861600
H	3.65718000	0.15123500	-2.74511700
C	5.19592600	0.89124500	-1.47394400
H	5.77104500	1.28336000	-2.30458500
C	5.67279100	1.01145700	-0.17667800
H	6.62107400	1.50200600	0.00901300
C	4.93895800	0.49076500	0.88399300
H	5.31324400	0.57424500	1.89739100
C	3.73373700	-0.14918600	0.64676800
H	3.18612200	-0.57406200	1.48171600
C	2.14299700	-2.90472600	-0.25451800
C	3.33845500	-3.48009000	-0.70070800
H	3.99708300	-2.94058100	-1.37272800
C	3.70330300	-4.74545800	-0.26944600
H	4.62797100	-5.18662300	-0.62247200
C	2.89166100	-5.44172000	0.62023000
H	3.18296800	-6.42868200	0.95996400
C	1.71419300	-4.86881100	1.07888700
H	1.08566300	-5.40407400	1.78078000
C	1.33872000	-3.60365600	0.64276100
H	0.41883900	-3.16749300	1.01304500
C	-2.75573300	-1.19216400	-2.39421000
C	-3.66179300	-0.57228900	-3.25798700
H	-3.39631300	0.32452600	-3.80397500

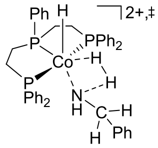

***mer*-TS3'**

C	2.07868800	2.15032400	-1.76869500
H	2.05394500	3.15845000	-2.18323100
H	3.10918400	1.94623300	-1.47010800
C	1.60969000	1.11013700	-2.78387500
H	2.31517500	1.01494100	-3.61233100
H	0.62547600	1.34766200	-3.19454600
C	0.70256700	-1.84732500	-2.76904100
H	-0.23521700	-1.48642900	-3.19771400
H	1.34591900	-2.18285000	-3.58510300
C	0.47979100	-2.95768500	-1.74328000
H	1.43647600	-3.38300800	-1.43180800
H	-0.12178800	-3.77140800	-2.15067900
C	0.02199900	3.59315800	-0.23439600
C	-0.35546000	4.19483600	-1.43751000
H	-0.04367900	3.78621800	-2.39195800
C	-1.13827900	5.34138300	-1.42739000
H	-1.41453700	5.81140100	-2.36399000
C	-1.56031900	5.88783400	-0.22142700
H	-2.16794000	6.78503400	-0.21640800
C	-1.19307000	5.28935400	0.97804900
H	-1.50974900	5.72101300	1.92025900
C	-0.40707100	4.14558900	0.97571000
H	-0.10292600	3.70555900	1.91894000
C	3.19573200	-0.98721400	-1.51693900
C	3.99781200	-1.27924000	-2.62803800
H	3.60753000	-1.20113500	-3.63777700
C	5.31448800	-1.67395500	-2.45004700
H	5.92947000	-1.89894700	-3.31341800
C	5.84259400	-1.77997800	-1.16766500
H	6.87230800	-2.08900600	-1.03129900
C	5.05255000	-1.49027400	-0.06417200
H	5.46315400	-1.57113800	0.93522200
C	3.73180600	-1.09433900	-0.23515400
H	3.12861500	-0.86769900	0.63557900
C	-2.09110000	-2.73834300	-0.31431100
C	-2.72032600	-2.88724600	-1.55460200
H	-2.16699100	-2.77532600	-2.48032100

C	-4.92492400	-1.11924200	-3.44376200
H	-5.62122500	-0.63835200	-4.12058800
C	-5.29128600	-2.27893200	-2.77385800
H	-6.27737900	-2.70242600	-2.92427000
C	-4.39152500	-2.90139800	-1.91535100
H	-4.67341700	-3.81121300	-1.39853100
C	-3.13016600	-2.36000500	-1.72158600
H	-2.43184900	-2.86112500	-1.05862700
C	-1.53436200	2.83241800	-0.17404200
C	-1.43049600	4.18836000	0.14682000
H	-0.45958600	4.64805900	0.28442700
C	-2.57659700	4.95804700	0.29611300
H	-2.48582700	6.00800100	0.54879900
C	-3.83136900	4.38842700	0.12355000
H	-4.72281200	4.99324700	0.24165200
C	-3.94184400	3.04017200	-0.19471400
H	-4.91968800	2.59095500	-0.32521000
C	-2.80033500	2.26282000	-0.33781000
H	-2.90530500	1.20613500	-0.55824000
C	1.34549800	2.81163800	0.23435500
C	2.19549900	3.60077800	-0.54127300
H	2.10818400	3.62505800	-1.62044200
C	3.16534100	4.38607200	0.07018000
H	3.81778200	5.00098000	-0.53859300
C	3.28943800	4.39445700	1.45263700
H	4.03938700	5.01734300	1.92598500
C	2.44679700	3.60789200	2.23046400
H	2.53876000	3.61528000	3.31027100
C	1.48434100	2.81556600	1.62463400
H	0.82763500	2.21413400	2.24423200
Co	-0.23965500	-0.43085200	-0.09525300
P	1.71373800	-1.24184200	-0.89536400
P	-1.06071100	-0.57185100	-2.17309400
P	-0.01776700	1.84221700	-0.49388100
H	-0.45067700	-1.86937000	-0.32040300
N	-1.67190800	-0.49974300	1.16914100
H	-2.16646100	-1.38874600	1.18865300
H	-0.30487700	-0.67477600	1.46029300
H	0.66424700	-0.30395900	1.15125700
C	-2.07880500	0.30898700	2.32756700
H	-1.42433000	1.17814900	2.39563700
H	-3.08182400	0.68690100	2.09831800
C	-2.09031800	-0.49136700	3.60352500
C	-0.97263400	-0.50705500	4.43830500
C	-3.20892000	-1.25164600	3.94645900
C	-0.97277200	-1.27251700	5.59690500
H	-0.10637700	0.10414700	4.20209100
C	-3.20739300	-2.01926000	5.10288200
H	-4.09777800	-1.22451500	3.32270700
C	-2.08823400	-2.03215800	5.92730000
H	-0.10759300	-1.26668600	6.24959000
H	-4.08575500	-2.59467700	5.37075600
H	-2.09149800	-2.62297300	6.83572600
C	-4.06793300	-3.21768200	-1.61720300
H	-4.54370600	-3.35274100	-2.58148700
C	-4.79864700	-3.38549100	-0.44777900
H	-5.84792600	-3.65113900	-0.49862800
C	-4.18206000	-3.22354600	0.78747900
H	-4.74908200	-3.36153900	1.70043000
C	-2.83610200	-2.89582900	0.85878200
H	-2.36032600	-2.79625500	1.82813500
P	1.05949000	2.08157500	-0.19569900
P	1.47752700	-0.46120300	-1.83415300
P	-0.31903900	-2.29292600	-0.18774500
C	0.32713300	-3.30637800	1.18418500
C	0.26193200	-4.69987300	1.07239400
C	0.85748600	-2.73038800	2.33673500
C	0.73828400	-5.49915600	2.09972700
H	-0.16953500	-5.16726100	0.19398500
C	1.32611900	-3.53771800	3.36611500
H	0.90336100	-1.65330200	2.43786600
C	1.26994500	-4.91933000	3.24701600
H	0.68833000	-6.57774700	2.00791400
H	1.73236600	-3.08594100	4.26343700
H	1.63611300	-5.54803400	4.05018000
C	2.24234700	2.41227300	1.16156100
C	3.19379100	3.42311000	0.99137200
C	2.17462500	1.73131000	2.37587800
C	4.07557300	3.72720000	2.01719800
H	3.24651600	3.98648100	0.06640600
C	3.05396900	2.04757000	3.40459100
H	1.43287800	0.95748200	2.52998200
C	4.00700400	3.04043800	3.22461600
H	4.81306200	4.50867900	1.87656600
H	2.99145600	1.51901800	4.34866600
H	4.69379000	3.28550600	4.02627300
Co	0.07542800	-0.01763000	-0.16628200
H	-0.42587200	0.14929700	1.28025100
N	-1.74330400	0.35842300	-0.73763200
H	-2.33793600	-0.47233300	-0.77831200
H	-1.26479700	0.30667700	0.61719300
C	-2.59710700	1.53883700	-0.89651400
H	-2.05750400	2.41830200	-0.55373100
H	-2.74777300	1.66361200	-1.97733300
C	-3.92757300	1.40185000	-0.20444000
C	-4.03079200	1.64758100	1.16593800
C	-5.06738200	1.03366500	-0.91669300
C	-5.25263700	1.52342500	1.81249300
H	-3.15930700	1.97050200	1.72825800
C	-6.29128700	0.91348200	-0.27079100
H	-5.00679800	0.86284600	-1.98717900
C	-6.38438300	1.15535700	1.09414500
H	-5.32729800	1.73022700	2.87384400
H	-7.17622100	0.64569600	-0.83632900
H	-7.34133300	1.07184100	1.59589600
H	1.22366600	-0.35041500	0.65125200

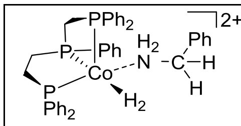

***fac*-VI'**

C	-2.16471300	1.72912200	-1.92905500
H	-2.61806600	0.80659100	-2.29631000
H	-2.86254200	2.54304300	-2.13413300
C	-0.81417300	1.98226200	-2.58242500
H	-0.46113400	2.99115800	-2.35874100
H	-0.85607200	1.88692500	-3.66985600
C	0.43941200	-0.70213800	-2.97138100
H	0.34369100	-0.40496700	-4.01894800
H	1.41785500	-1.17276600	-2.85567900
C	-0.66307700	-1.67340400	-2.56218600
H	-0.55106700	-2.62049400	-3.09364100
H	-1.65157900	-1.28987700	-2.82407500
C	-3.52448400	1.16661400	0.64093200
C	-4.60956100	0.73199300	-0.12120800
H	-4.52529200	0.59082500	-1.19163200
C	-5.83310700	0.48952300	0.49061900
H	-6.67637400	0.16711300	-0.10873100
C	-5.97703800	0.66462300	1.86028900
H	-6.93484800	0.48000100	2.33254800
C	-4.89540900	1.08914800	2.62511300
H	-5.00845300	1.23822400	3.69255200
C	-3.67390700	1.34202900	2.02012700
H	-2.84860100	1.70677100	2.62323200
C	-1.51074400	3.23914900	0.48930400
C	-2.37816900	4.29915900	0.20918700
H	-3.29064600	4.13475100	-0.35352800
C	-2.08971500	5.57352000	0.67321600
H	-2.76569600	6.39129900	0.45250300
C	-0.94530000	5.80049900	1.43115200
H	-0.72936500	6.79666200	1.79916400
C	-0.08669300	4.75024300	1.72623100
H	0.79644800	4.92313400	2.33022000
C	-0.36870000	3.47336200	1.25515400
H	0.30434600	2.65597200	1.49959800
C	2.07799000	1.63052900	-2.18044600
C	3.05402700	1.10769900	-3.03281700
H	2.89181400	0.18625200	-3.57750500
C	4.25160700	1.78693500	-3.21646500
H	4.99917500	1.38091900	-3.88761100
C	4.48682000	2.98317400	-2.55228400
H	5.42100600	3.51133900	-2.70262600
C	3.52179000	3.50626800	-1.69846100
H	3.70111600	4.44304800	-1.18406700
C	2.32413700	2.83416400	-1.50825900
H	1.57711100	3.25799500	-0.84454200
C	0.61310200	-3.20150900	-0.38124100
C	0.68878400	-3.70080500	0.92427100

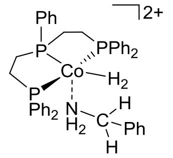

***mer*-VI'**

C	-1.01647300	3.29287600	1.24807800
H	-0.57365200	4.12794500	1.79312000
H	-1.68168400	3.70036400	0.48280800
C	-1.77030900	2.35955100	2.18313800
H	-2.63813300	2.84222600	2.63549500
H	-1.13005000	2.02515200	3.00336900
C	-2.72361200	-0.46665800	2.41306100
H	-1.99287400	-0.44877200	3.22599700
H	-3.70315400	-0.24676500	2.84323300
C	-2.72218300	-1.80516900	1.68414000
H	-3.60429200	-1.89249900	1.04597200
H	-2.73965500	-2.64339400	2.38297500
C	1.76571000	2.44042700	1.48194300
C	1.79039000	1.61499200	2.61054800
H	0.99799200	0.89291700	2.78517300
C	2.83762300	1.69842100	3.51715800
H	2.84672600	1.06120100	4.39379700
C	3.87675900	2.59538900	3.29701800
H	4.69602600	2.65965100	4.00337900
C	3.86772500	3.40253100	2.16718000
H	4.68162800	4.09522700	1.98768300
C	2.81805600	3.32809500	1.25970300
H	2.82753100	3.95955300	0.38002600
C	-3.73179000	1.26529500	0.24642500
C	-4.54961000	2.34208100	0.59876000
H	-4.29970100	2.99005600	1.42950600
C	-5.71244800	2.59863000	-0.11462400
H	-6.33830800	3.43746400	0.16630900
C	-6.07411800	1.78385300	-1.17942400
H	-6.98349300	1.98634000	-1.73284200
C	-5.26939500	0.70791400	-1.53295000
H	-5.54905700	0.06826500	-2.36175800
C	-4.10080700	0.45279300	-0.82987200
H	-3.48506500	-0.38898700	-1.12632900
C	0.00642600	-2.93947500	1.46026800
C	0.24039700	-2.70038900	2.81791100
H	-0.34772900	-1.97178100	3.36441300
C	1.21039000	-3.42331600	3.49749500
H	1.36876200	-3.24954900	4.55550300
C	1.96791400	-4.37693300	2.82669100
H	2.71928700	-4.94588300	3.36156400
C	1.75012500	-4.61000400	1.47527700
H	2.32881800	-5.36208500	0.95198800
C	0.77543800	-3.89480000	0.78967500
H	0.59002500	-4.11443800	-0.25581900
P	0.32171500	2.34352800	0.35213100
P	-2.23430900	0.85868500	1.21559500

H	-0.01000900	-3.36022200	1.68255700	P	-1.24836600	-1.96266100	0.54338900
C	1.61680000	-4.67848200	1.25114500	C	-1.80838900	-3.05872300	-0.81007200
H	1.64718600	-5.07603500	2.25914900	C	-2.46954400	-4.25057600	-0.49288600
C	2.49177900	-5.16128300	0.28356300	C	-1.58216100	-2.73664100	-2.14814800
H	3.21163800	-5.93057300	0.53697800	C	-2.90301200	-5.09429200	-1.50409000
C	2.42925300	-4.66700600	-1.01159000	H	-2.63918000	-4.53184400	0.54044800
H	3.09932500	-5.04981100	-1.77259500	C	-2.01409900	-3.58788000	-3.15814700
C	1.49397900	-3.69298800	-1.34574000	H	-1.06698000	-1.82197100	-2.41896700
H	1.44849000	-3.35271400	-2.37350400	C	-2.67656100	-4.76428300	-2.83636200
C	-2.24040700	-2.85068600	-0.38854500	H	-3.41641600	-6.01465300	-1.25161400
C	-2.57263900	-3.97675700	-1.14890600	H	-1.83273900	-3.33110100	-4.19519700
H	-1.93500100	-4.30599700	-1.96175400	H	-3.01534800	-5.42805900	-3.62311600
C	-3.71735800	-4.70010400	-0.85351000	C	0.67511600	3.37840600	-1.11480400
H	-3.97164300	-5.57043800	-1.44707800	C	0.69762100	4.77300000	-1.00114500
C	-4.52836700	-4.31998100	0.21135300	C	0.89224500	2.79572700	-2.36422200
H	-5.41655500	-4.89504200	0.44582000	C	0.94184200	5.56056900	-2.11702100
C	-4.19705400	-3.21068800	0.97596800	H	0.52888800	5.25618600	-0.04573500
H	-4.82431500	-2.91596200	1.80869200	C	1.13684800	3.58740700	-3.47849800
C	-3.05721400	-2.47394900	0.67595400	H	0.86845500	1.71838900	-2.48018500
H	-2.81760500	-1.60220600	1.27351500	C	1.16244700	4.96996800	-3.35569400
Co	-0.07726900	0.19360800	0.17241800	H	0.95787800	6.63962000	-2.01809300
P	-1.90259700	1.55013100	-0.10486300	H	1.30236900	3.12462000	-4.44431700
P	0.46726600	0.81673700	-1.91111000	H	1.35074400	5.58859100	-4.22523400
P	-0.68773700	-1.94265100	-0.72337500	Co	-0.41935900	0.15635200	0.08318000
H	-0.56957800	-0.59180800	1.53131800	H	-0.94487500	0.79599900	-1.53396900
H	-0.58562100	0.19559900	1.75094600	N	1.32213600	-0.66826900	-0.64547900
H	2.49919800	0.32284900	0.12244500	H	1.44250900	-1.55367800	-0.15160100
C	2.26117400	-0.10927300	2.11074100	H	1.14886400	-0.95598400	-1.60759500
H	1.93662300	0.87711400	2.44703000	C	2.67773800	-0.01606800	-0.63525900
C	3.73928200	-0.27033300	2.31925600	H	2.58948100	0.94550300	-1.13966600
C	4.56647600	0.84991000	2.39777700	H	2.93341400	0.17740700	0.40512900
C	4.30578300	-1.54210200	2.41730500	C	3.71467100	-0.87359900	-1.30234200
C	5.93664500	0.70285500	2.56781300	C	3.90475300	-0.80099000	-2.68338700
H	4.13718700	1.84622300	2.34742000	C	4.48316700	-1.76970300	-0.55888100
C	5.67526200	-1.68950900	2.58558700	C	4.83737800	-1.61499700	-3.31115500
H	3.67207900	-2.42325700	2.38126800	H	3.33958200	-0.08429600	-3.27289800
C	6.49202200	-0.56712200	2.65985300	C	5.41819500	-2.58262800	-1.18562500
H	6.56994800	1.57910100	2.64229400	H	4.36605300	-1.81853200	0.51955900
H	6.10615600	-2.67995200	2.67414500	C	5.59378800	-2.50848600	-2.56205800
H	7.56006900	-0.68274900	2.80217900	H	4.98678300	-1.54213100	-4.38207900
N	1.86066600	-0.25408800	0.66929900	H	6.02161500	-3.26513300	-0.59843900
H	2.06724900	-1.21315000	0.39003200	H	6.33013800	-3.13668800	-3.04941000
H	1.70020300	-0.85491000	2.67645100	H	-1.26857200	0.08476100	-1.52365200
