# Supplementary material for: Homogeneous cobalt-catalyzed reductive amination for synthesis of functionalized primary amines
Source: Nat Commun. 2019 Nov 29;10:5443. doi: 10.1038/s41467-019-13351-7 (PMC6884468; doi:10.1038/s41467-019-13351-7)
Supplement: Supplementary file 1 — Peer Review File [file 41467_2019_13351_MOESM1_ESM.pdf]

Reviewers' comments:

Reviewer #1 (Remarks to the Author):

In this paper, Murugesan et. al. report a homogenous cobalt-catalyzed reductive amination for the synthesis of primary amines. 24 combinations of metal salts and ligands were screened and the combination of $Co(BF_4)_2 \cdot 6H_2O$ and linear Triphos was found to be the only system active for primary amine formation. . The substrate scope is very wide and diverse and even includes commercially available drugs. The yields of products are very good in most cases. This is a notable example of an abundant metal catalyst having superior performance to precious metal catalysts. It is a breakthrough article and likely should be published in a high profile journal like Nature Communications.
Criticisms of the work are that no direct information is provided about why this particular system is so selective and how it works. For example why does the product amine not add to the aldehyde or ketone and eventually lead to the formation of secondary amines as is usually the case in amination reactions?
No direct support is provided for the hydride and amide intermediates proposed in Figure 1; on the other hand such evidence is difficult to produce for a system under a pressure of dangerous gases like H_2 and NH_3. At least computational (Density Functional Theory) data should be provided to demonstrate that these steps have reasonable energies. Alternative mechanisms can be proposed with different oxidation states of cobalt. The authors have chosen Co(II) but cobalt phosphine complexes in different oxidation states might be good hydrogenation catalysts although Co(II) is a popular choice for the active catalyst (e.g. Friedfeld, M. R.; Zhong, H.; Ruck, R. T.; Shevlin, M.; Chirik, P. J. Science 2018, 360, 888-893).

Other comments:

Introduction:
- Are enzyme-catalyzed reductive aminations an alternative to noble metal catalysis? Sharma, M.; Mangas-Sanchez, J.; France, S. P.; Aleku, G. A.; Montgomery, S. L.; Ramsden, J. I.; Turner, N. J.; Grogan, G. ACS Catalysis 2018, 8, 11534-11541

Results and Discussion:

- Line 71 there is a mention that, "the presence of strongly coordinating anions (e.g. halides) is inferior for hydrogen catalysis". In view of the high activity of Wilkinson's Rh catalyst, and Noyori's Ru catalyst, this statement is not correct for 2nd row transition metals. Do you mean first row TM?
- Line 91 is written as if it is surprising the ligand coordinates in a 2:1 ratio to the metal. Square pyramidal Co(II) complexes are common in the crystallographic database. Probably the authors mean that, starting with a 1:1 ratio, it was surprising to find a 2:1 ratio in the isolated complex.
- The active complex A should have its solution magnetic susceptibility measured via Evan's method as part of the characterization. Also a paramagnetic 1H NMR spectrum of the complex should be shown in the SI.
- Fig. 1. BF4 to BF(subscript 4) and complex A should be dicationic (only one positive sign shown).
- Phosphorous to phosphorus
- " we cannot exclude a direct hydrogenation to give II." Do you mean a direct hydride addition without prior coordination?
- "produce liner primary" – liner to linear
- "In order to proof the" – proof to prove

Supporting information
- The English is poor in the preparation of the cobalt complex. Many articles (a, the) are missing. String instead of stirring.

- Why is the preparation of complex A done with a ratio of 1:1 Co to triphos when 1:2 might be used to get a better yield? This needs to be explained.
- The spectra should be labeled as 1H NMR or 13C{1H} NMR with the frequency of the spectrometer and the solvent (DMSO-d6). Where 19F is present, that spectrum should be presented since it would be a good indicator of purity.

Reviewer #2 (Remarks to the Author):

The manuscript reported by Jagadesh and co-workers titled "Homogeneous cobalt-catalyzed reductive amination for the synthesis of functionalized primary amines" is quite interesting and useful methodology to access variety of primary amines. Although there are several positive points in the manuscript, I am afraid that this manuscript is suitable for nature communications due to the following reasons.
1) The author earlier published heterogenous cobalt-nanocatalysts for reductive amination of carbonyl derivatives citing heterogenous system scores over homogeneous catalytic system (see ref 52). In addition, recently the same author also published the reductive amination of carbonyl derivatives (aldehyde and ketone) using Ru-based homogenous catalytic system.
2) The presented manuscript is not much novel except using Co/linear triphos catalyst and the scope of the carbonyl derivatives including "life-science molecules" are the same as reported earlier.
3) The manuscript can be considered as improve catalytic system if so, the author has to compare the cobalt-nanoparticle, Ru-based catalytic system with the currently developed catalyst and show the efficience of this catalyst over the reported catalytic system.
4) There are some mistakes in the schemes. For eg, in Fig. 1, complex A is dicationic but the chemdraw structure shows mono cationic, same wit the structure of comple B.
5) Is it possible to isolate or detect the Co-H species by 1H NMR (Complex A + H2 (5 bar) pressure)? What will be efficiency of the werner complex as precatalyst along with linear phosphine for mentioned reaction under the optimized conditions.
6) Its better to do some DFT calculations in order to find out whether it operates via inner sphere or outer sphere mechanism? The mechanism, which operates with Ru, may or may not be the same as 3d transition metals may vary due to different spin states.

Reviewer #3 (Remarks to the Author):

Reductive amination with ammonia using hydrogen to access primary amines is an important reaction. In this manuscript, Beller, Jagadeesh and coworkers identified an efficient homogeneous cobalt catalyst for the reductive amination of aldehydes and ketones to amines, although the nickel-based catalytic systems have already been reported by the same group. Isolation of an active pre-catalyst was made and the oxidation of catalytic species was pointed out as a potential deactivation pathway. Overall, this is a nice demonstration of cobalt catalysis. Publication is recommended after the following issues are addressed.

1. Could the authors comment on why phosphine L7 offered the best activity? Have they tried to methylate the NH of L6 and examined its reactivity?
2. DFT calculations (even some preliminary work) could be included to provide some insights and/or support the proposed mechanism.
3. Have the authors tried to isolate catalyst intermediate I? This will allow a closer look at the reactivity of the Co-H moiety.
4. What is the best TON one can achieve under O2 free condition?

Reply to the comments of Reviewers

Reply to the comments of Reviewer-1

In this paper, Murugesan et. al. report a homogenous cobalt-catalyzed reductive amination for the synthesis of primary amines. 24 combinations of metal salts and ligands were screened and the combination of Co(BF4)2·6H2O and linear Triphos was found to be the only system active for primary amine formation. . The substrate scope is very wide and diverse and even includes commercially available drugs. The yields of products are very good in most cases. This is a notable example of an abundant metal catalyst having superior performance to precious metal catalysts. It is a breakthrough article and likely should be published in a high profile journal like Nature Communications.

Reply: We are thankful to the Reviewer for highlighting the importance of our work and recommending it for the publication in Nature Communication.

Criticisms of the work are that no direct information is provided about why this particular system is so selective and how it works. For example why does the product amine not add to the aldehyde or ketone and eventually lead to the formation of secondary amines as is usually the case in amination reactions?

Reply: As mentioned by the reviewer, in case of reductive amination reaction of carbonyl compounds with ammonia, side reactions such as over-alkylation (to produce secondary imine or amine) or reduction to the corresponding alcohols are likely occur. Hence, highly active and selective catalysts are required for this reaction to produce primary amine.

In reductive amination reaction with ammonia, initially the carbonyl compound **1** undergoes condensation with ammonia to form the corresponding primary imine **1$^{/}$**. Subsequently, the intermediate imine is hydrogenated in presence of catalyst to give the primary amine **2** **(**See **Scheme A**, below**)**. Intermediate **1$^{/}$**, however, was never detected in the reaction mixture, due to its high reactivity/instability. Instead, secondary imine **4** was determined in case of less active catalysts, which is formed *via* condensation of the product **2** with either the starting aldehyde/ketone (releasing water) or *via* condensation of **2** and **a** (releasing NH_3). Due to rapid hydrogenation under optimized conditions in presence of Co-triphos catalyst, the stationary concentration of **1$^{/}$** is low, preventing side reactions of this reactive intermediate. When the hydrogenation does not proceed quickly (in case of less active catalysts), the accumulation of **1$^{/}$** can likely undergo side reaction to produce **4**. The secondary imine **4**, in presence of catalyst can undergoes hydrogenation to produce secondary amine **5**. In the present case, Co-triphos system is very active to hydrogenate primary imine **1$^{/}$** to produce primary amine **2**. Hence, under optimized conditions by using Co-triphos, there is no formation of secondary imine or imine was occurred.

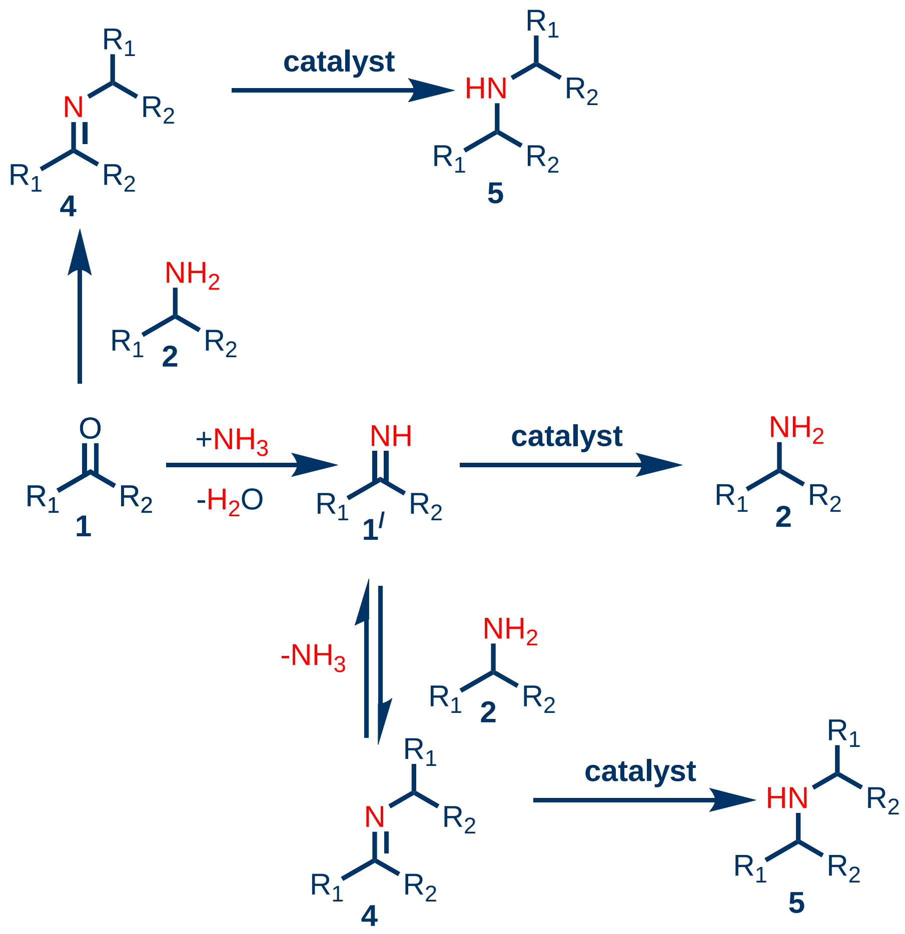

Scheme A. Reaction pathway for the reductive amination of carbonyl compounds with ammonia in presence of hydrogen.

In case of homogeneous catalyzed reductive aminations, the deactivation of catalysts by the formation of stable Werner-type ammine complexes in presence of ammonia is likely occurs. As shown in Table S3 (Entry 5), the Werner complex is not active for the reaction to produce primary amine. In order to avoid the formation of Werner complex, strongly coordinating ligands for the formation of corresponding stable metal complex is essential. Compared to mono and bi-dentate phosphine ligands or PNP ligand (L6), the triphos based tri-dentate phosphine ligands (L7 and L8) are found to be promising due their strong coordination to the metal center. Hence, Co-triphos system constitutes highly active and selective catalyst system for the reductive amination of carbonyl compounds with ammonia to produce primary amines.

No direct support is provided for the hydride and amide intermediates proposed in Figure 1; on the other hand such evidence is difficult to produce for a system under a pressure of dangerous gases like H2 and NH3. At least computational (Density Functional Theory) data should be provided to demonstrate that these steps have reasonable energies. Alternative mechanisms can be proposed with different oxidation states of cobalt.

Reply: We thank the reviewer for this interesting suggestion. We also agree that it is quite difficult to to produce evidence for the formation of hydride and amide intermediates during the reaction under pressurized conditions. Also, it is difficult to isolate them. However, as suggested by the reviewer now in the revised manuscript we have provided DFT studies to support our hypothesis. An inner-sphere mechanism on the basis of the mono-cationic $[triphos\text{-}CoH]^+$ complex as active catalyst has been proposed and verified with density functional theory computation on the doublet state potential free energy surface and H_2 metathesis is found as the rate-determining step.

The authors have chosen Co(II) but cobalt phosphine complexes in different oxidation states might be good hydrogenation catalysts although Co(II) is a popular choice for the active catalyst (e.g. Friedfeld, M. R.; Zhong, H.; Ruck, R. T.; Shevlin, M.; Chirik, P. J. Science 2018, 360, 888-893).

Reply: As per the suggestion of reviewer, we tested different Co slats and found that Co(II) ($Co(BF_4)_2 \cdot 6H_2O$) works better compared to other (Please see the table below). These results have also been included in the revised supporting information.

Table S3. Reductive amination of 4-methylbenzaldehyde with different cobalt salt.

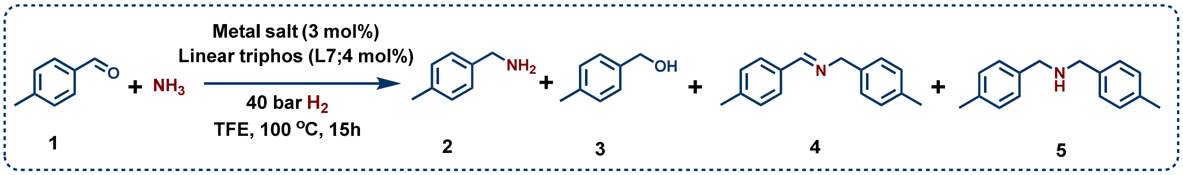

Entry	Cobalt salt	Ligand	Conv. (%)	Yield of 2 (%)	Yield of 3 (%)	Yield of 4 (%)	Yield of 5 (%)
1	$Co_2(CO)_8$	L7	90	68	-	20	-
2	$Co(PPh_3)_3Cl$	L7	95	88	-	5	-
3	$Co(BF_4)_2 \cdot 6H_2O$	L7	>99	96	-	2	-
4	$Co(acac)_3$	L7	70	-	-	68	-
5	$Co[(NH_3)6]Cl_3$ Werner complex	L7	10	-	-	8	-

Reaction conditions: 0.5 mmol 4-methylbenzaldehyde, 3 mol% Cobalt salt, 4 mol% linear triphos (**L7**), 5-7 bar NH_3, 40 bar H_2, 2 mL trifluoroethanol (TFE), 100 °C, 15 h, GC yields using n-hexadecane as standard.

Other comments:

Introduction:

- Are enzyme-catalyzed reductive aminations an alternative to noble metal catalysis? Sharma, M.; Mangas-Sanchez, J.; France, S. P.; Aleku, G. A.; Montgomery, S. L.; Ramsden, J. I.; Turner, N. J.; Grogan, G. ACS Catalysis 2018, 8, 11534-11541

Reply: Enzyme based catalysts have also been used for the reductive amination to produce primary amines; especially for asymmetric reductive aminations. However, these catalysts are less explored / applicable for the preparation of more functionalized and structurally diverse primary amines.

Results and Discussion:

- Line 71 there is a mention that, “the presence of strongly coordinating anions (e.g. halides) is inferior for hydrogen catalysis”. In view of the high activity of Wilkinson’s Rh catalyst, and Noyori’s Ru catalyst, this statement is not correct for 2nd row transition metals. Do you mean first row TM?

Reply: Thanks for rightly pointing out this statement. Yes, we meant for first row transition metals.

- Line 91 is written as if it is surprising the ligand coordinates in a 2:1 ratio to the metal. Square pyramidal Co(II) complexes are common in the crystallographic database. Probably the authors mean that, starting with a 1:1 ratio, it was surprising to find a 2:1 ratio in the isolated complex.

Reply: Yes, we were surprised too. During optimization, we observed that *in situ* generated L7-Co complex with both 1:1 and 2:1 ratio of ligand to cobalt exhibited similar activity and selectivity. Hence, we thought there could be a formation of 1:1 complex. Hence, we started with 1:1 ratio of ligand to metal ratio and obtained 2:1 ratio in the isolated complex with 48-50% yield.

- The active complex A should have its solution magnetic susceptibility measured via Evan’s method as part of the characterization. Also a paramagnetic 1H NMR spectrum of the complex should be shown in the SI.

Reply: With respect the suggestion of reviewer, the magnetic susceptibility of active complex **A** has been measured and its paramagnetic ^{1}H NMR spectrum are given in the revised SI. Please also see below for spectrum and magnetic susceptibility data.

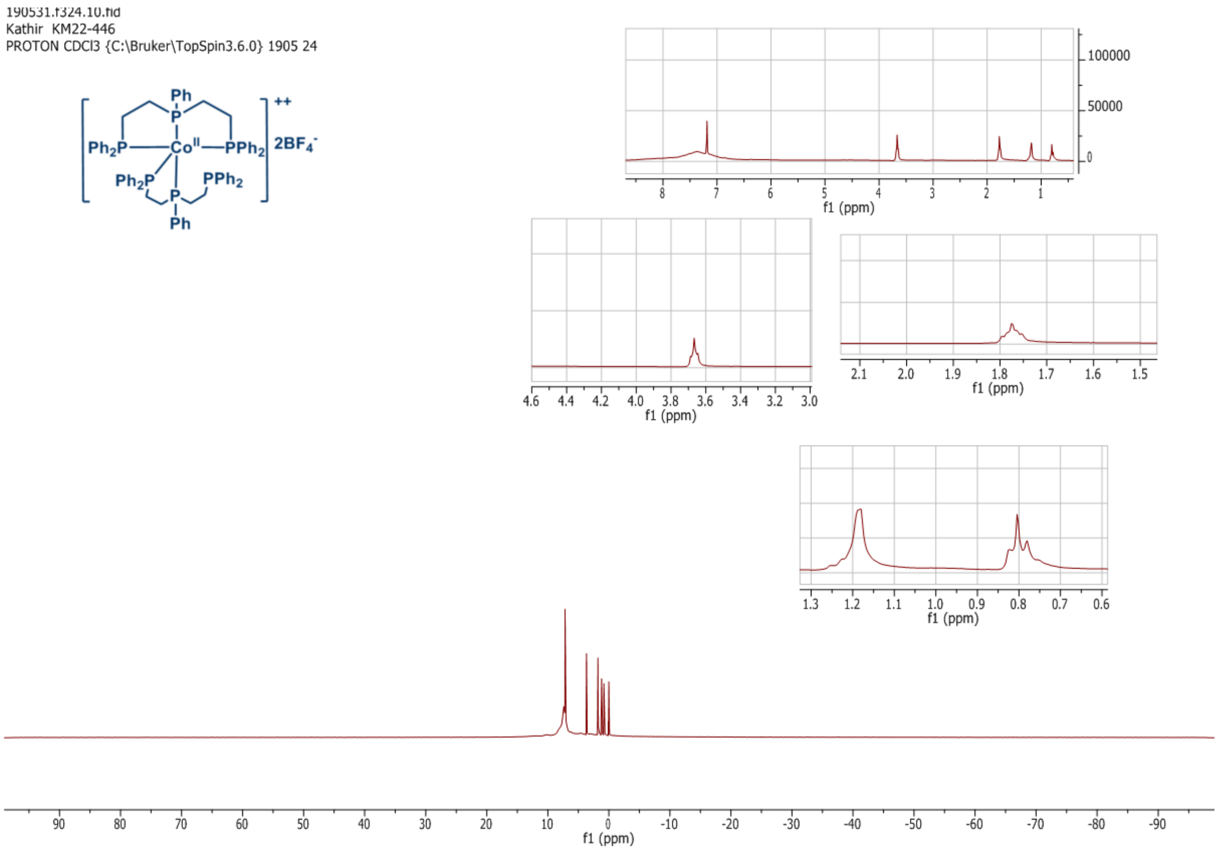

^{1}H NMR (**300 MHz, Chloroform-*d***) of paramagnetic complex-A

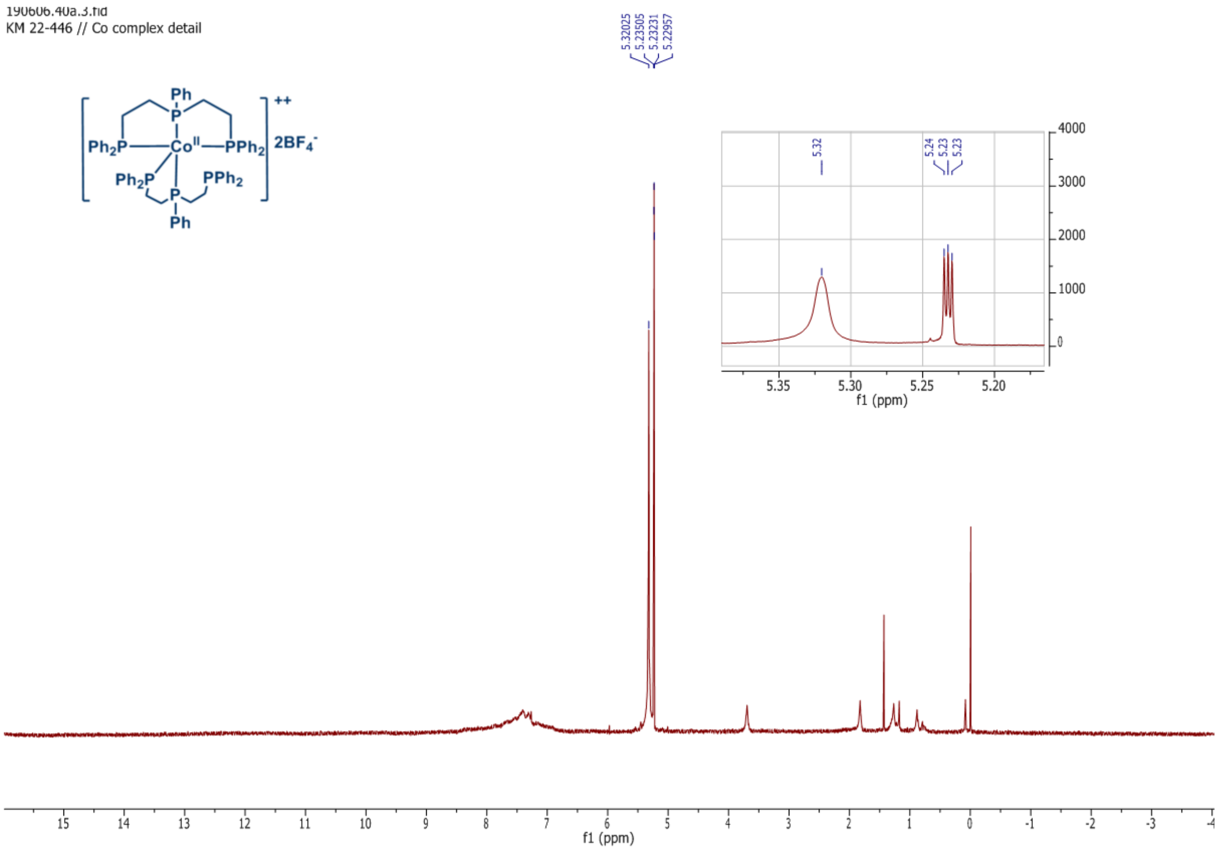

^{1}H NMR (**400 MHz, Dichloromethane-*d*$_2$**) of complex-A by The Evans Method

Paramagnetic susceptibility measurement preliminary results:

Entry	Measurement	Data
1	Observed paramagnetic shift	0,088 ppm (^{1}H)
2	Mass susceptibility χ_m	$8{,}92 \cdot 10^{-10}$ m^3/kg
3	Molar susceptibility χ_M	$1{,}16 \cdot 10^{-9}$ m^3/mol
4	Number of unpaired electron	1

- Fig. 1. BF4 to BF(subscript 4) and complex A should be dicationic (only one positive sign shown).

Reply: Thanks for rightly pointing out this mistake. These errors have been corrected now.

- Phosphorous to phosphorus

Reply: This error is corrected now.

- “we cannot exclude a direct hydrogenation to give II.” Do you mean a direct hydride addition without prior coordination?

Reply: Yes, we mean direct hydride addition without prior coordination. However, based on DFT studies, we omitted this statement and suitable correction has been made.

- “produce liner primary” – liner to linear

Reply: This error is corrected now.

- “In order to proof the” – proof to prove

Reply: This error is corrected now.

Supporting information

- The English is poor in the preparation of the cobalt complex. Many articles (a, the) are missing. String instead of stirring.

Reply: Thanks for finding out these mistakes. In the revised SI, these mistakes have been rectified.

- Why is the preparation of complex A done with a ratio of 1:1 Co to triphos when 1:2 might be used to get a better yield? This needs to be explained.

Reply: During the optimization of catalytic reductive amination reaction, we observed that the *in situ* generated L7-Co complex with both 1:1 and 1:2 ratio of ligand to cobalt gave similar activity and selectively. Hence we believed that there might be the possibility of the formation of 1:1 complex. Accordingly, we performed the experiment to prepare complex **A** with 1:1 ratio. After

isolation and analysis, we observed the formation of 1:2 complexes with 48-50% yield. The isolated complex **A**, also exhibited similar activity and selectivity to that of 1:1 *in situ* complex. After having these results and since, all the catalytic reactions were carried out by using *in situ* complex with 1:1 ratio, we were not further focused to investigate the formation of complex with different ratios of Co to ligand.

- The spectra should be labeled as 1H NMR or 13C{1H} NMR with the frequency of the spectrometer and the solvent (DMSO-d6). Where 19F is present, that spectrum should be presented since it would be a good indicator of purity.

Reply: Thanks for this nice suggestion. All of these details have been included in the revised supporting information.

Reply to the comments of Reviewer-2

Reviewer #2 (Remarks to the Author):

The manuscript reported by Jagadesh and co-workers titled “Homogeneous cobalt-catalyzed reductive amination for the synthesis of functionalized primary amines” is quite interesting and useful methodology to access variety of primary amines. Although there are several positive points in the manuscript, I am afraid that this manuscript is suitable for nature communications due to the following reasons.

1) The author earlier published heterogenous cobalt-nanocatalysts for reductive amination of carbonyl derivatives citing heterogenous system scores over homogeneous catalytic system (see ref 52). In addition, recently the same author also published the reductive amination of carbonyl derivatives (aldehyde and ketone) using Ru-based homogenous catalytic system.

   Reply: We thank the reviewer for mentioning our previous reductive amination works using heterogeneous Co- and homogeneous Ru-based catalysts. As we discussed in the introduction of this manuscript, the development of base metal homogeneous catalysts is not explored and remains as challenging for the reductive amination to prepare primary amines. Typically, reductive amination are carried out using heterogeneous catalysts and comparatively homogeneous catalysts are less explored due to common problems associated with this reaction (Pleas see introduction). The development of related homogeneous non-noble metal catalysts remains interesting because of the inherent advantage regarding activity - in principle all the individual metal centers can be active here. Furthermore, compared to homogeneous catalysts the upscaling of advanced heterogeneous materials possess additional challenges.

2) The presented manuscript is not much novel except using Co/linear triphos catalyst and the scope of the carbonyl derivatives including "life-science molecules" are the same as reported earlier.

Reply: As we discussed in introduction and vide supra, our aim is to demonstrate the possibility of challenging reductive amination reaction by using simple base metal homogeneous catalyst. Accordingly, for the first time we report such a simple in situ Co-catalyst, which is compatible with the known precious metal-based homogenous catalysts as well as state-of-the-art heterogeneous catalysts. In fact, the Co-triphos catalyst systems works under mild reaction conditions to that of previously reported Ru complexes and Co-nanocatalyst. Please see Section S6 of the revised supporting information on the comparison of reactivity of different catalysts.

3) The manuscript can be considered as improve catalytic system if so, the author has to compare the cobalt-nanoparticle, Ru-based catalytic system with the currently developed catalyst and show the efficiency of this catalyst over the reported catalytic system.

Reply: We have compared the reactivity of this Co-triphos catalyst with previously reported cobalt-nanoparticles as well as Ru-based homogeneous catalysts. Interestingly, this Co-triphos system works under mild conditions and showed better activity and selectivity compared to previously reported catalyst systems under similar experimental conditions. These results have been included in the revised supporting information (S6). Please also see below:

S6. Comparison reactivity and selectivity of Co-triphos system with previously reported Co-nanoparticles and Ru-based complexes for the reductive amination to prepare primary amines

A) This work with Co-triphos catalyst

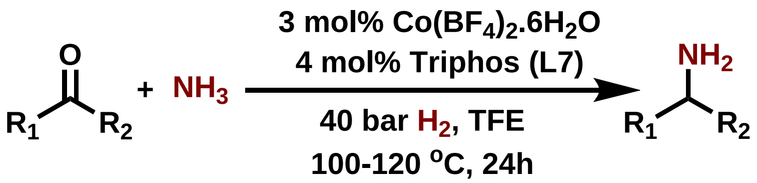

B) Using previously reported Co-nanoparticles (ref: *Science,* 2017, 358, 326-332)

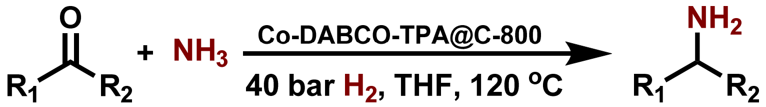

B) Using Ru- and Rh-based homogeneous catalysts

1	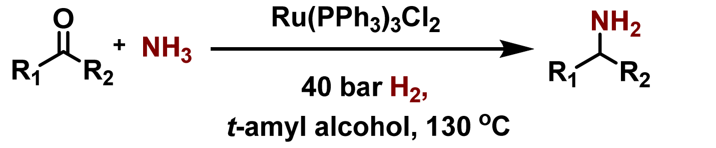	***Nature Commun.*, 2018, *9*, 4123**
2	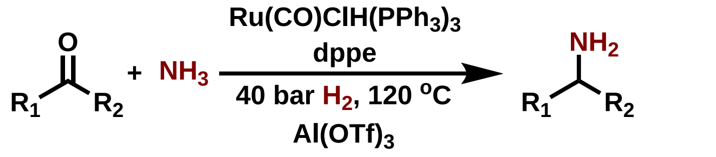	***Adv. Synth. Catal*. 2016, *358*, 358**
3	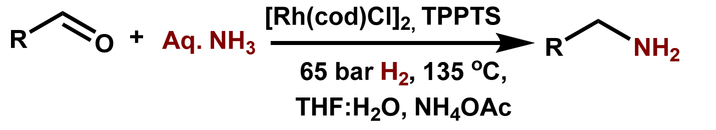	***Org. Lett.*, 2002, *12*, 2055**

Table S5. Comparison of cobalt triphos system with previously reported Ru-homogeneous catalysts under our standard reaction conditions.

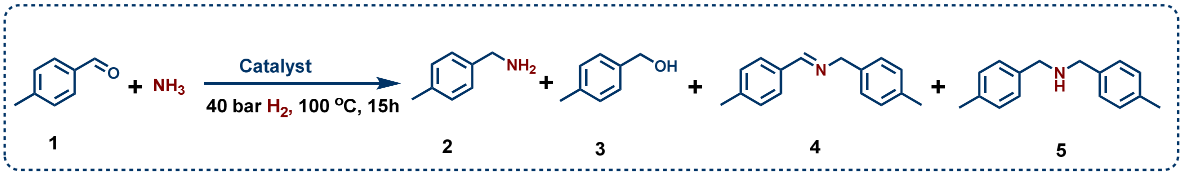

Entry	Catalyst	Conv. (%)	Yield of 2 (%)	Yield of 3 (%)	Yield of 4 (%)	Yield of 5 (%)
1[A]	Co-triphos(2019) This work	>99	96	-	2	-
2[B]	Ru (2018) *Nature Commun.*, **2018**, 9, 4123	72	-	-	70	-
3[C]	Ru (2016) *Adv. Synth. Catal.* **2016**, *358,* 358.	>99	30	-	68	-

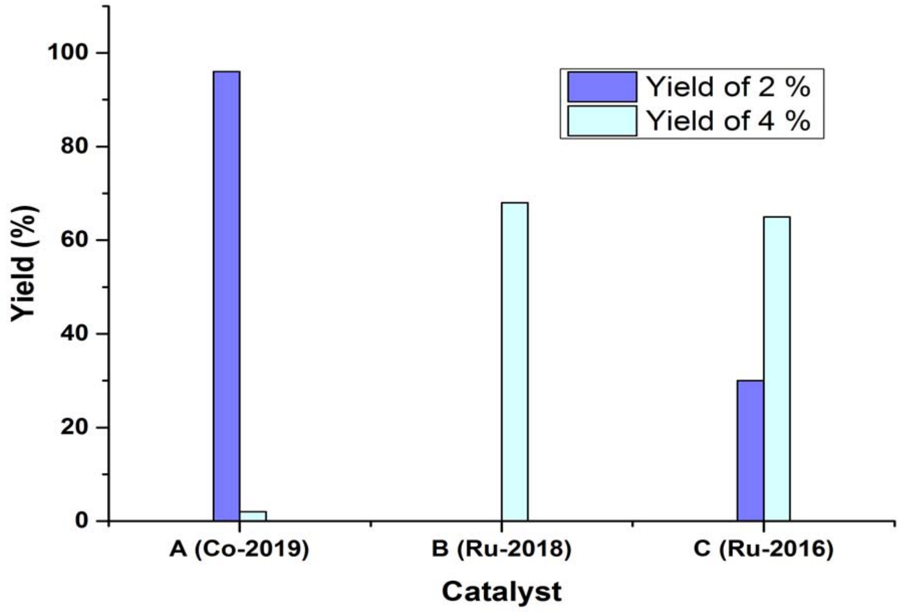

Reaction conditions: [A]0.5 mmol 4-methyl benzaldehyde, 3 mol% $Co(BF_4)_2 \cdot 6H_2O$, 4 mol% triphos (L7), 5-7 bar NH_3, 40 bar H_2, 2 mL trifluoroethanol (TFE), 100 ^oC, 15 h. [B]0.5 mmol 4-methyl benzaldehyde, 2 mol% $RuCl_2(PPh_3)_3$, 5-7 bar NH_3, 40 bar H_2, 2 mL *t*-amyl alcohol, 100 ^oC, 15 h. [C]0.5 mmol 4-methyl benzaldehyde, 1 mol% $Ru(Co)ClH(PPh_3)_3$, 1.1 mol% dppe (L4), $Al(OTf)_3$10 mol%, 5-7 bar NH_3, 40 bar H_2, 2 mL Toluene, 100 ^oC, 15 h GC yields using n-hexadecane as standard. Products 3 and 5 are not detected

4) There are some mistakes in the schemes. For eg, in Fig. 1, complex A is dicationic but the chemdraw structure shows mono cationic, same with the structure of complex B.

   Reply: We thank the reviewer for finding out these mistakes. These mistakes have been corrected now

5) Is it possible to isolate or detect the Co-H species by 1H NMR (Complex A + H2 (5 bar) pressure)? What will be efficiency of the Werner complex as precatalyst along with linear phosphine for mentioned reaction under the optimized conditions?

   Reply: The complex **A** is paramagnetic and hence it is quite difficult to measure Co-H species by ^1H NMR. Further to prove the paramagnetic nature, we performed the Evan's method and details have been given in the revised SI. As suggested, we tested Werner complex under our standard reaction conditions and observed 10% of conversion to yield only secondary imine as sole product without the formation of primary amine, a desired product. (Please see Table S3 in the SI)

6) Its better to do some DFT calculations in order to find out whether it operates via inner sphere or outer sphere mechanism? The mechanism, which operates with Ru, may or may not be the same as 3d transition metals may vary due to different spin states.

   Reply: With respect to reviewer suggestion we carried out DFT calculation. Yes, this catalyst also operates via an inner-sphere mechanism. Please see revised manuscript and SI for DFT studies.

Reply to the comments of Reviewer-3

Reductive amination with ammonia using hydrogen to access primary amines is an important reaction. In this manuscript, Beller, Jagadeesh and coworkers identified an efficient homogeneous cobalt catalyst for the reductive amination of aldehydes and ketones to amines, although the nickel-based catalytic systems have already been reported by the same group. Isolation of an active pre-catalyst was made and the oxidation of catalytic species was pointed out as a potential deactivation pathway. Overall, this is a nice demonstration of cobalt catalysis. Publication is recommended after the following issues are addressed.

Reply: We thank the reviewer for recommending our paper to publish in Nature communications.

1. Could the authors comment on why phosphine L7 offered the best activity? Have they tried to methylate the NH of L6 and examined its reactivity?

Reply: In case of homogeneous catalyzed reductive aminations, the deactivation of catalysts by the formation of stable Werner-type ammine complexes in presence of ammonia is likely occurs. As shown in Table S3 (Entry 5), the Werner complex is inactive for the reductive amination to produce primary amines. In order to avoid the formation of Werner complex, strongly coordinating ligands for the formation of corresponding stable metal complex is essential. Compared to mono and bi-dentate phosphine or PNP (L6), ligands the triphos based tri-dentate phosphine ligands (L7 and L8) are found to be promising due their strong coordination to the metal center. Hence, Co-triphos system constitutes highly active and selective catalyst system for the reductive amination of carbonyl compounds with ammonia to produce primary amines. We tested N-methylated L6 ligand and found that this ligand is also not active (observed <5& of conversion).

DFT calculations (even some preliminary work) could be included to provide some insights and/or support the proposed mechanism.

Reply: We have performed DFT studies and these data have been included in the revised manuscript and supporting information.

2. Have the authors tried to isolate catalyst intermediate **I**? This will allow a closer look at the reactivity of the Co-H moiety.

   Reply: We tried to isolate catalyst intermdiates, but unfortunately we could not able to isolate them. The complex **A** is paramagnetic and hence it is quite difficult identify Co-H species by ^{1}HMR. Further to prove the paramagnetic nature, we performed the Evan’s method and details have been given in the revised SI.

3. What is the best TON one can achieve under O2 free condition?

   Reply: The maximum turn over number is 32.66 for 2-methyl benzaldehyde as a substrate with 3 mol% of catalyst.

REVIEWERS' COMMENTS:

Reviewer #1 (Remarks to the Author):

My concerns have been addressed. The new discussion of the DFT results have some minor errors:
Fig. 1. ++ is unconventional Please use 2+ or +2.
Beta hydride addition or beta-migration is better than "hydride insertion" since the H is not inserting between atoms in a bond

in [the] gas phase
in [a] solution

Reviewer #2 (Remarks to the Author):

The author almost address all the concerns of the reviewers. The manuscript is now substantially improved to be published in nature communications. However, i would like the author to change the structure of amine into ammonium salt as the product they isolate is not primary amine, it's in salt form (see supporting information). To show amine in the manuscript and ammonium salt in the supporting information is some what misleading the readers and it should not be done. Otherwise, i would like to see this nice manuscript online soon.

Reviewer #3 (Remarks to the Author):

The revised manuscript has addressed most of the issues and has included more studies to analyze the observed reactivity with a more specific and improved mechanistic understanding. Publication is thus recommended.

1. A proposed mechanism can only be disproved or supported, but never verified. Thus, "verified" in the abstract should be revised to "supported".

2. This point is not absolutely critical, but since the authors have conducted DFT calculations to address a plausible mechanism, why didn't they also compute different reaction pathways to support the observed product selectivity?

Reply to the comments of Reviewers

Reply to the comments of Reviewer-1

Reviewer #1 (Remarks to the Author):

My concerns have been addressed. The new discussion of the DFT

Results have some minor errors:

Reply: We thank the reviewer for recommending our paper for its publication in Nature Communications and also for finding out some minor errors in the manuscript.

Fig. 1. ++ is unconventional Please use 2+ or +2.

Reply: Thanks for this suggestion. Now this correction has been made in the revised manuscript.

Beta hydride addition or beta-migration is better than “hydride insertion” since the H is not inserting between atoms in a bond

Reply: Hydride insertion is now changed to beta hydride addition

in [the] gas phase

Reply: This mistake has been corrected now.

in [a] solution

Reply: This mistake has been corrected now.

Reply to the comments of Reviewer-2

Reviewer #2 (Remarks to the Author):

The author almost addresses all the concerns of the reviewers. The manuscript is now substantially improved to be published in nature communications.

Reply: We thank the reviewer for recommending our revised manuscript to be published in Nature Communications.

However, I would like the author to change the structure of amine into ammonium salt as the product they isolate is not primary amine, it's in salt form (see supporting information). To show amine in the manuscript and ammonium salt in the supporting information is some what misleading the readers and it should not be done. Otherwise, i would like to see this nice manuscript online soon.

Reply: We isolated the amines in free form. For measuring NMR, we converted the isolated free amines to HCl salts. Initially we observed some of the amines are not stable to measure NMR and also free amine peaks in some cases are not clearly visible in ^{1}H NMR spectra. For this reason we converted free amines into HCl salts after they have been isolated and measured NMR and HRMS. These details have already been included at the foot note of Figs.3-5 and also in the experimental procedure. Since we have isolated the amines in free from and hence we retain the structure of amines in the manuscript as free amines and in NMR spectra as slat form. We hope this is acceptable to reviewer now.

Reply to the comments of Reviewer-3

Reviewer #3 (Remarks to the Author):

The revised manuscript has addressed most of the issues and has included more studies to analyze the observed reactivity with a more specific and improved mechanistic understanding. Publication is thus recommended.

Reply: We are thankful to the reviewer for recommending our revised manuscript to be published in Nature Communications.

A proposed mechanism can only be disproved or supported, but never verified. Thus, “verified” in the abstract should be revised to “supported”.

Reply: We thank the reviewer for this nice observation and suggestion. Accordingly, we have changed the word verified to supported.

This point is not absolutely critical, but since the authors have conducted DFT calculations to address a plausible mechanism, why didn’t they also compute different reaction pathways to support the observed product selectivity?

Reply: We appreciate the reviewer for this interesting question and suggestion. In case of most active catalyst system under optimized conditions, we have not observed any other side product except minor amount of secondary imine (<3%). In addition to the direct hydrogenation of primary imine to primary amine, there might be possibility for the conversion of secondary imine in presence of catalyst, ammonia and hydrogen to the desired product primary amine. This secondary imine forms by the reaction of primary amine with carbonyl compound or with primary imine. However, in the presence of active catalyst we observed the formation of secondary imine in < 3%. The formation of primary amine is likely and exclusively occurs by the hydrogenation of primary imine. Hence, we considered mainly computing DFT calculation for the hydrogenation of primary imine to primary amine and not for other pathways of product selectivity.
